# Unique behaviour of dinitrogen-bridged dimolybdenum complexes bearing pincer ligand towards catalytic formation of ammonia

**DOI:** 10.1038/ncomms4737

**Published:** 2014-04-28

**Authors:** Hiromasa Tanaka, Kazuya Arashiba, Shogo Kuriyama, Akira Sasada, Kazunari Nakajima, Kazunari Yoshizawa, Yoshiaki Nishibayashi

**Affiliations:** 1 Institute for Materials Chemistry and Engineering and International Research Center for Molecular Systems, Kyushu University, Nishi-ku, Fukuoka 819-0395, Japan; 2 Elements Strategy Initiative for Catalysts and Batteries (ESICB), Kyoto University, Nishikyo-ku, Kyoto 615-8520, Japan; 3 Institute of Engineering Innovation, School of Engineering, The University of Tokyo, Yayoi, Bunkyo-ku, Tokyo 113-8656, Japan; 4 These authors contributed equally to this work

## Abstract

It is vital to design effective nitrogen fixation systems that operate under mild conditions, and to this end we recently reported an example of the catalytic formation of ammonia using a dinitrogen-bridged dimolybdenum complex bearing a pincer ligand, where up to twenty three equivalents of ammonia were produced based on the catalyst. Here we study the origin of the catalytic behaviour of the dinitrogen-bridged dimolybdenum complex bearing the pincer ligand with density functional theory calculations, based on stoichiometric and catalytic formation of ammonia from molecular dinitrogen under ambient conditions. Comparison of di- and mono-molybdenum systems shows that the dinitrogen-bridged dimolybdenum core structure plays a critical role in the protonation of the coordinated molecular dinitrogen in the catalytic cycle.

## Abstract (web summary)

Research into molecular catalysts for conversion of nitrogen into ammonia under mild conditions is ongoing. Here, the authors synthesize a dimolybdenum–nitride complex and verify the role of the dimetallic core in the protonation of the coordinated dinitrogen.

## Graphical abstract

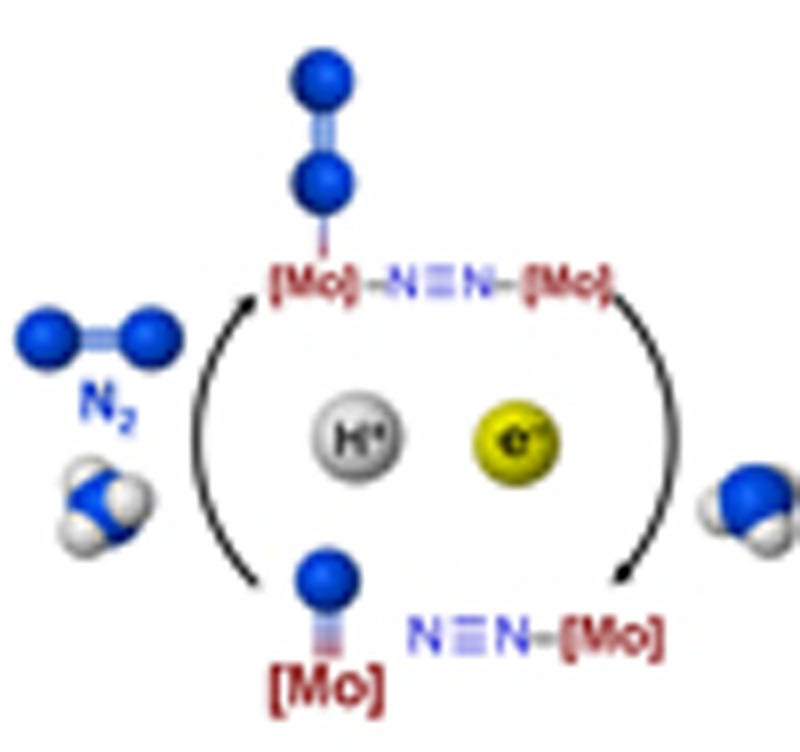

Nitrogen is an essential element for human beings. To supply the increasing demand of nitrogenous fertilizer, the Haber-Bosch process has long been used industrially to form ammonia from molecular dinitrogen and dihydrogen gasses^1^. The production of ammonia by the Haber-Bosch process requires drastic reaction conditions such as high temperature and high pressure because of the extreme chemical inertness of molecular dinitrogen, although molecular dinitrogen is readily available in plenty from the atmosphere^1^. From a viewpoint of energy, the production of ammonia from molecular dinitrogen and molecular dihydorgen is considered to be the most economical process; however, an enormous amount of energy (over 90% of the total energy of the Haber-Bosch process) was consumed for the production of molecular dihydrogen from fossil fuels. As a result, the development of the alternative to the energy-consuming Haber-Bosch process without the use of molecular dihydrogen has therefore been awaited for a long period of time^1^.

Since the discovery of the first example of a transition metal–dinitrogen complex, [Ru(N_2_)(NH_3_)_5_]^2+^ in 1965 (ref. 2), a variety of transition metal–dinitrogen complexes have been prepared, and the reactivity of the coordinated dinitrogen ligand has been studied extensively to exploit a novel catalytic reaction system of molecular dinitrogen by using transition metal–dinitrogen complexes under mild reaction conditions^3^,^4^,^5^,^6^,^7^,^8^,^9^. Among a variety of transition metal–dinitrogen complexes known to date, molybdenum–dinitrogen complexes have intriguing reactivities because the coordinated dinitrogen on the molybdenum atom is easily converted into ammonia by the protonation with inorganic acids such as sulphuric acid, where only a stoichiometric amount of ammonia is produced based on the molybdenum atom^10^,^11^,^12^.

In sharp contrast to the stoichiometric transformations, there are only a few examples of catalytic transformations by using transition metal–dinitrogen complexes as catalysts^13^,^14^,^15^,^16^,^17^,^18^,^19^,^20^,^21^. In 2003, Schrock and co-worker found the first example of the catalytic conversion of molecular dinitrogen into ammonia by using molybdenum–dinitrogen complex bearing a triamidoamine as the supporting ligand under ambient conditions, where less than 8 equiv of ammonia were produced based on the molybdenum atom^22^,^23^,^24^,^25^,^26^. Results of the theoretical study on the reaction pathway also support that the catalytic reaction proceeds via some reactive intermediates such as mononuclear hydrazide, -hydrazidium and -nitride complexes^22^,^23^,^24^,^25^,^26^. Quite recently, Peters and co-workers have reported the first successful example of the iron-catalysed direct transformation from molecular dinitrogen into ammonia at −78 °C, where up to 7 equiv of ammonia were produced based on the iron atom of iron–dinitrogen complex bearing a tris(phosphine)borane ligand^27^. Although they have clarified some elementary steps of the catalytic reaction, the whole catalytic cycle has not yet been clarified until now.

As an extensive study on the development of novel nitrogen fixation systems under ambient reaction conditions^28^,^29^,^30^,^31^,^32^,^33^,^34^,^35^,^36^, we have recently found another successful example of the catalytic conversion of molecular dinitrogen into ammonia by using dinitrogen-bridged dimolybdenum complex bearing a PNP-type pincer ligand [Mo(N_2_)_2_(**PNP**)]_2_(μ-N_2_) (**1**: **PNP**=2,6-bis(di-tert-butylphosphinomethyl)pyridine), where up to 23 equiv of ammonia were produced based on the catalyst (12 equiv of ammonia based on the molybdenum atom)^37^,^38^,^39^,^40^,^41^. In this paper, we postulate a reaction pathway for the catalytic conversion of molecular dinitrogen into ammonia, where mononuclear molybdenum–dinitrogen complexes bearing the PNP-type pincer ligand have been considered to work as key reactive intermediates. To obtain more detailed information on the reaction pathway, we prepare the mononuclear molybdenum–nitride complexes bearing the PNP-type pincer ligand and examine their catalytic reactivity towards the catalytic formation of ammonia from molecular dinitrogen, because transition metal–nitride complexes are considered to work as key reactive intermediates in the conversion of the coordinated dinitrogen into ammonia^42^,^43^,^44^,^45^,^46^,^47^. We also perform a density functional theory (DFT) study on the reaction pathway based on the stoichiometric and catalytic reactivities of the newly isolated molybdenum complexes bearing the PNP-type pincer ligand. The combined experimental and theoretical studies reveal that the dinitrogen-bridged dimolybdenum core structure plays a crucial role to promote the catalytic reaction in the protonation of the coordinated molecular dinitrogen in the catalytic cycle. This result is in sharp contrast to our previous proposals, where only mononuclear molybdenum complexes were proposed to work as key reactive intermediates^37^,^38^,^39^,^40^,^41^. In this article, we propose a new catalytic reaction pathway with the aid of DFT calculations and experimental results.

## Results

### Preparation and reactivity of molybdenum–nitride complexes

**Figure 1:**
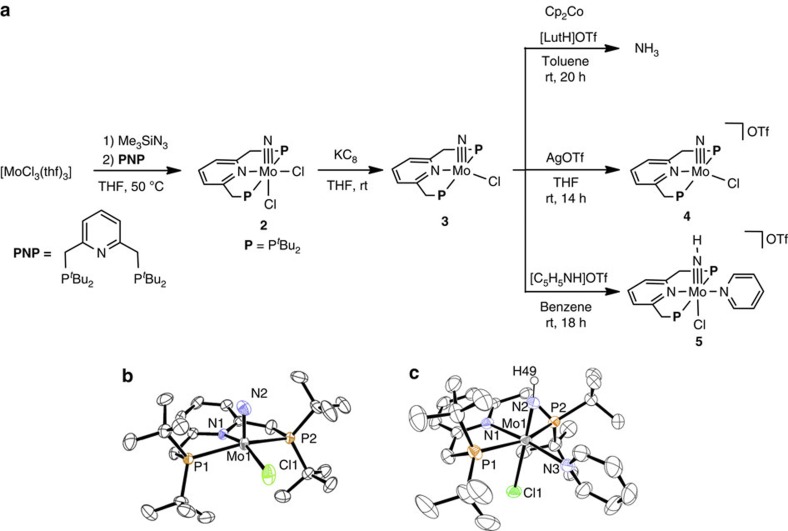
Preparation and reactivity of molybdenum–nitride complexes. (**a**) Preparation and reactivity of 2–5. (**b**) An ORTEP drawing of the cationic part of 4. Thermal ellipsoids are shown at the 50% probability level. Hydrogen atoms are omitted for clarity. (**c**) An ORTEP drawing of the cationic part of 5. Thermal ellipsoids are shown at the 50% probability level. Hydrogen atoms except for H49 are omitted for clarity.

As described in the previous paper, we have already prepared a hydrazide complex bearing the PNP-type pincer ligand [Mo(NNH_2_)F(**PNP**)(C_5_H_5_N)]BF_4_ (C_5_H_5_N=pyridine) by the protonation of **1** with tetrafluoroboric acid; however, this hydrazide complex has no catalytic activity towards the catalytic conversion of molecular dinitrogen into ammonia^37^,^38^,^39^,^40^,^41^. As a next step, we paid our attention to the preparation of molybdenum–nitride complexes^42^,^43^,^44^,^45^,^46^,^47^ bearing the PNP-type pincer ligand. Treatment of [MoCl_3_(thf)_3_] with Me_3_SiN_3_ at 50 °C for 1 h and then the addition of **PNP** at 50 °C for 4 h gave a paramagnetic molybdenum(V) nitride complex [Mo(≡N)Cl_2_(**PNP**)] (2) in 43% yield (Fig. 1a). A preliminary diffraction study of 2 displays the distorted octahedral molybdenum(V) geometry with the *mer*-**PNP** ligand, and the nitride ligand occupied a position *trans* to one of the chloride ligands (see Supplementary Fig. 1, Supplementary Tables 1 and 3 and Supplementary Data 1). Subsequently, reduction of 2 with 1 equiv of KC_8_ in THF at room temperature gave a diamagnetic molybdenum(IV) nitride complex [Mo(≡N)Cl(**PNP**)] (3) in 46% yield (Fig. 1a). The ^1^H NMR of 3 indicates a set of signals for the **PNP** ligand and its preliminary X-ray study also reveals a distorted square-pyramidal geometry with the **PNP** and chloride ligands in the basal plane and the nitride ligand in the apical position (see Supplementary Fig. 2, Supplementary Tables 1 and 4 and Supplementary Data 2). The infrared spectrum exhibits a weak ν_Mo14N_ band at 1,031 cm^−1^ (ν_Mo15N_=1,003 cm^−1^). To confirm the reactivity of the nitride ligand in 3, the stoichiometric reaction of 3 with 4 equiv of Cp_2_Co (Cp=η^5^-C_5_H_5_) and [LutH]OTf (Lut=2,6-lutidine; OTf=OSO_2_CF_3_) was carried out under Ar atmosphere. As a result, ammonia was produced in 83% yield based on the Mo atom in 3 (Fig. 1a).

The reaction of 3 with 1 equiv of AgOTf afforded a paramagnetic molybdenum(V) nitride complex [Mo(≡N)Cl(**PNP**)]OTf (4) in 52% yield (Fig. 1a). The detailed molecular structure of 4 is unambiguously determined by X-ray crystallographic analysis (Fig. 1b, Supplementary Fig. 3, Supplementary Tables 2 and 5 and Supplementary Data 3). The crystal structure of 4 displays a distorted square-pyramidal geometry, which is closely related to that of 3. The nitride ligand resides in the apical position, and the Mo≡N bond length is 1.634(3) Å. The chloride ligand is located *trans* to the nitrogen atom of **PNP**.

Next, the preparation of the molybdenum(IV) imide complex by the protonation of 3 was carried out. Treatment of 3 with 1 equiv of [LutH]OTf in THF gave only unidentified greenish products. When 3 was protonated with 1 equiv of pyridinium trifluoromethanesulphonate [C_5_H_5_NH]OTf as a proton source instead of [LutH]OTf in benzene, a diamagnetic molybdenum(IV) imide complex [Mo(≡NH)Cl(**PNP**)(C_5_H_5_N)]OTf (5) was obtained in 53% yield as green crystals (Fig. 1a). The ^1^H NMR spectrum of 5 exhibits a set of **PNP** and C_5_H_5_N ligands, while the imide proton could not be assigned. The infrared spectrum of 5 reveals the *ν*(N−H) band at 3,126 cm^−1^. The detailed structure of 5 has been established by an X-ray diffraction study (Fig. 1c, Supplementary Fig. 4, Supplementary Tables 2,6 and Supplementary Data 4). The molybdenum centre has a distorted octahedral geometry with **PNP** and C_5_H_5_N in the equatorial plane and mutually *trans* NH and Cl ligands. The Mo–N (imide) bond length is elongated to 1.711(3) Å from that of 4.

With the nitride and imide complexes bearing the **PNP** ligand in hand, we have investigated their catalytic activity towards the reduction of molecular dinitrogen into ammonia. When 2 (0.020 mmol) was used as a catalyst in the presence of excess amounts of CoCp_2_ (0.72 mmol) and [LutH]OTf (0.96 mmol) under an atmospheric pressure of dinitrogen, only a stoichiometric amount of ammonia was formed based on the molybdenum atom in 2 (Table 1, run 2). In contrast, 3 exhibited the catalytic activity to afford 6.6 equiv of ammonia based on the molybdenum atom in 3, which is comparable to that of **1** (Table 1, run 3). Complex 4 also worked as an effective catalyst in contrast to 2, where 7.1 equiv of ammonia were produced based on the molybdenum atom in 4 (Table 1, run 4). On the basis of the results of the stoichiometric and catalytic reactions of newly prepared nitride complexes, we believe that 3 and 4 can be regarded as reactive intermediates in the catalytic reduction of molecular dinitrogen into ammonia. In contrast, no catalytic activity of 2 is considered to be due to the coordination of the second chloro ligand to the molybdenum centre, which may inhibit the generation of the corresponding reactive species. Complex 5 did not work as a catalyst under the same reaction conditions (Table 1, run 5). The pyridine ligand coordinated to the Mo atom in 5 is considered to inhibit the generation of reactive species towards the catalytic reaction. In fact, addition of an excess amount (10 equiv) of pyridine to **1** in the catalytic reduction of molecular dinitrogen in the presence of **1** as a catalyst markedly decreased the catalytic activity. This experimental result supports our proposal on the nature of the pyridine ligand in 5.

### Theoretical calculations

We have investigated a possible reaction pathway catalysed by **1** with DFT calculations. On the basis of the above experimental findings, a mononuclear molybdenum(IV) nitride complex [**Mo**(≡N)(OTf)] (**Mo**=[Mo(**PNP**)]) can be regarded as a key intermediate. This means that the dinuclear complex [**Mo**(N_2_)_2_]_2_(μ-N_2_) **1** must be separated into the corresponding two mononuclear molybdenum complexes at a certain stage in the course of the catalytic reaction. This speculation is reasonable because no dinuclear molybdenum complex except for **1** was experimentally isolated from the catalytic reaction, and the newly prepared mononuclear molybdenum(IV) nitride complex [**Mo**(≡N)Cl] 3 was revealed to be capable of serving as a catalyst towards the catalytic formation of ammonia.

**Figure 2:**
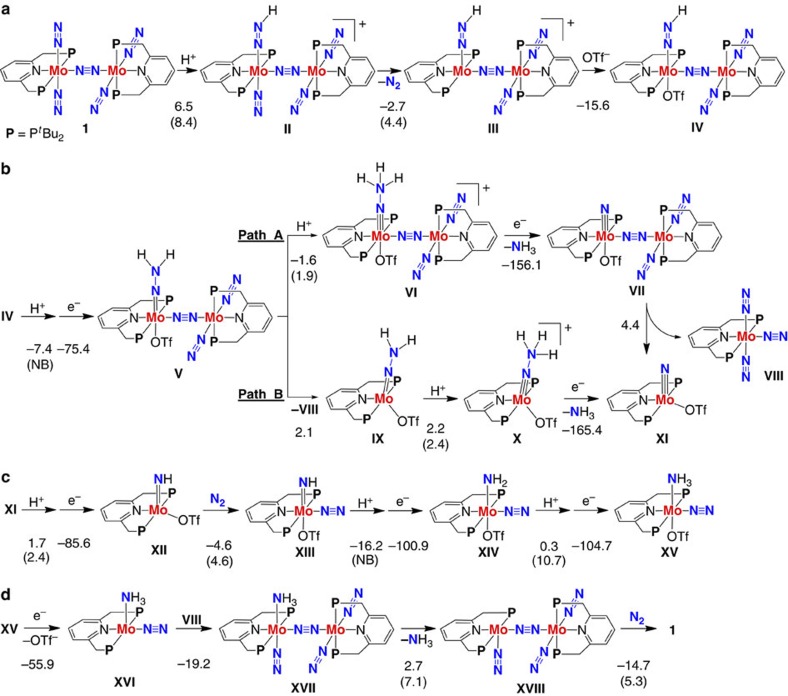
A possible reaction pathway by **1**. (**a**) Protonation of a terminal dinitrogen ligand in **1** followed by exchange of the dinitrogen ligand *trans* to the NNH group for OTf group. Protons and electrons are supplied by lutidinium and cobaltocene, respectively. Energy changes and activation energies (in parenthesis) for individual reaction steps were calculated at the B3LYP*/BS2 level of theory (units in kcal mol^−1^). NB represents that the corresponding reaction has no activation barrier. (**b**) A sequential protonation/reduction of **IV** and separation of bimetallic complexes leading to formation of ammonia and the monometallic nitride complex **XI**. (**c**) A sequential protonation/reduction of **XI** via the six-coordinate imide complex **XIII** to give the ammonia complex **XV**. (**d**) Ligand exchange of ammonia for molecular dinitrogen leading to regeneration of **1**.

In our previous report on the transformation of molecular dinitrogen into ammonia catalysed by **1**, we proposed a reaction pathway that **1** is first separated into the corresponding two mononuclear molybdenum–dinitrogen complexes, and then one of the dinitrogen ligands on the Mo atom leads to ammonia^37^. On the basis of computational results obtained in the present paper, we have newly proposed a reaction pathway involving the separation of dinuclear molybdenum complexes after a sequential protonation/reduction of a terminal dinitrogen ligand as well as regeneration of **1** linked with ligand exchange of ammonia for molecular dinitrogen. Figure 2 shows a plausible mechanism on the transformation of molecular dinitrogen into ammonia catalysed by **1** via a mononuclear molybdenum–nitride complex as a key intermediate. Detailed information on optimized structures of reactant complexes, transition states and product complexes in individual reaction steps is described in Supplementary Fig. 5, Supplementary Tables 7–74 and Supplementary Methods.

### Catalytic reaction pathway catalysed by 1

As shown in Fig. 2a, the transformation of molecular dinitrogen into ammonia starts with protonation of a terminal dinitrogen ligand in **1** to form [**Mo**(N_2_)(NNH)–N≡N–**Mo**(N_2_)_2_]^+^ **II**. The dinitrogen ligand *trans* to the NNH group in **II** is readily replaced by OTf group that is the counter anion of LutH^+^ (**II**→**III**→**IV**). Protonation and one-electron reduction of **IV** afford a hydrazide(2–) complex [**Mo**(OTf)(NNH_2_)–N≡N–**Mo**(N_2_)_2_] **V** (Fig. 2b). After protonation of the hydrazide complex **V**, reduction of [**Mo**(OTf)(NNH_3_)–N≡N–**Mo**(N_2_)_2_]^+^ **VI** induces a spontaneous N–N bond cleavage to generate the first molecule of ammonia together with [**Mo**(OTf)(≡N)–N≡N–**Mo**(N_2_)_2_] **VII** (Path A in Fig. 2b). The dinuclear nitride complex **VII** is readily separated into the corresponding two mononuclear complexes [**Mo**(N_2_)_3_] **VIII** and [**Mo**(OTf)(≡N)] **XI**, the latter of which is a key reactive intermediate in the proposed catalytic mechanism (vide infra). When **V** is separated into the corresponding two mononuclear complexes **VIII** and [**Mo**(OTf)(NNH_2_)] **IX**, the NNH_2_ group in **IX** is protonated and reduced to afford **XI** and ammonia (**IX**→**X**→**XI**; Path B in Fig. 2b).

Figure 2c describes sequential protonation/reduction steps of **XI** resulting in the corresponding ammonia complex **XV**. Protonation/reduction and the coordination of molecular dinitrogen to **XI** result in the formation of a six-coordinate imide complex [**Mo**(OTf)(N_2_)(NH)] **XIII** (**XI**→**XII**→**XIII**). Complex **XIII** is finally converted into the ammonia complex **XV** via two sequential protonation/reduction steps (**XIII**→**XIV**→**XV**).

The proposed catalytic cycle is completed by regeneration of **1** involving exchange of the ammonia ligand for a newly incoming molecular dinitrogen (Fig. 2d). Reduction of **XV** results in a spontaneous elimination of the OTf group. A five-coordinate complex [**Mo**(N_2_)(NH_3_)] **XVI** reacts with complex **VIII**, generated from dinuclear molybdenum complexes **V** or **VII** (vide supra), to afford a dinuclear ammonia complex **XVII**. Finally, ligand exchange of ammonia in **XVII** for molecular dinitrogen leads to the regeneration of **1**.

### Discussion on key steps of the catalytic reaction pathway

**Figure 3:**
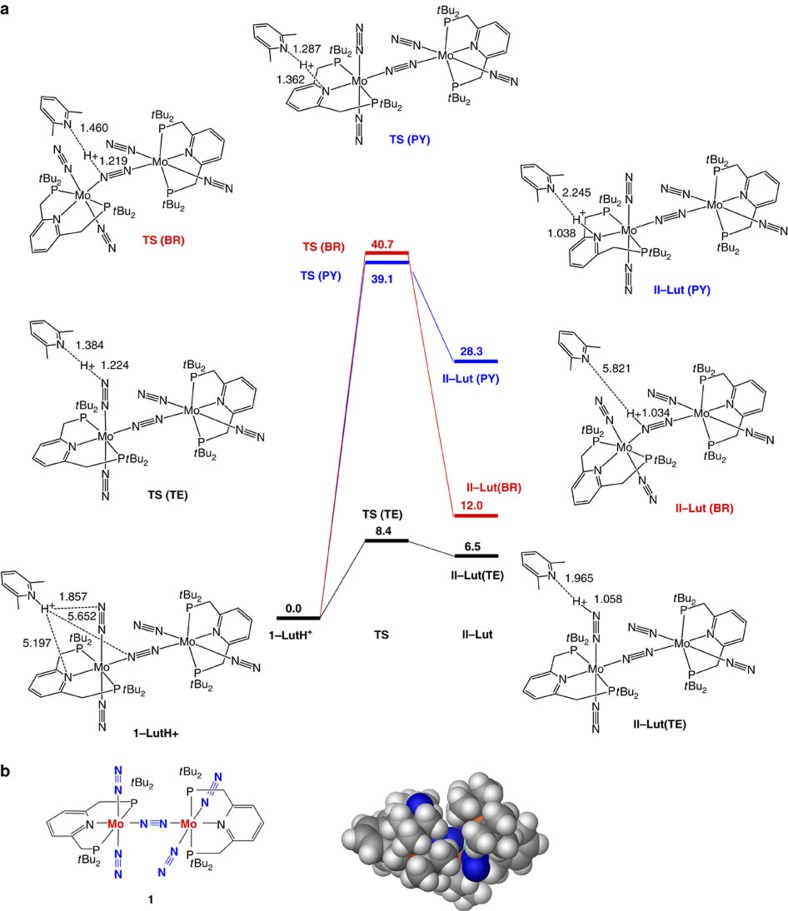
The first protonation step of 1. (**a**) Energy profiles for proton transfer from LutH^+^ to a terminal dinitrogen ligand (**TE**, black), the bridging dinitrogen ligand (**BR**, red) and the pyridine nitrogen atom in the pincer ligand (**PY**, blue) in **1**. Relative energies are given in kcal mol^−1^. (**b**) A space-filling model of **1**.

On the assumption of alternating protonation/reduction steps in the transformation of molecular dinitrogen, one of the dinitrogen ligands in **1** should be protonated at the first step of the catalytic cycle. Since **1** contains four equivalent terminal dinitrogen ligands and one bridging dinitrogen ligand, **1** has at least two reaction sites for the first protonation. Infrared and Raman spectra of **1** indicate that the bridging dinitrogen ligand is more strongly activated and is a better candidate for protonation. However, as shown in Fig. 3a, the protonation of the bridging dinitrogen ligand requires an extremely high activation energy (40.7 kcal mol^−1^), and thus this process does not likely occur at room temperature. In contrast, the activation energy is relatively low (8.4 kcal mol^−1^), although the protonation of a terminal dinitrogen ligand is endothermic by 6.5 kcal mol^−1^. A space-filling model of **1** in Fig. 3b indicates that the bridging dinitrogen ligand is sterically protected by eight *tert*-butyl groups on the phosphorus atoms in the pincer ligands, which make LutH^+^ inaccessible to the bridging dinitrogen ligand without a large distortion around the Mo–N–N–Mo moiety. For the transformation of N_2_ into NH_3_ catalysed by [HIPTN_3_N]Mo(N_2_), where HIPTN_3_N=(3,5-(2,4,6-i-Pr_3_C_6_H_2_)_2_C_6_H_3_N-CH_2_CH_2_)_3_N, the mechanism of the first protonation/reduction step has been thoroughly investigated^48^,^49^,^50^,^51^. Recent infrared and electron-nuclear double resonance studies reported by Schrock and co-workers^48^,^50^ demonstrated that protonation first occurs at an amide nitrogen of the HIPTN_3_N ligand of [HIPTN_3_N]Mo(N_2_). At present, the most probable reaction pathway for the conversion of [HIPTN_3_N]Mo(N_2_) into [HIPTN_3_N]Mo(NNH) involves protonation of an amide nitrogen of HIPTN_3_N. The protonated intermediate undergoes reduction and protonation of the N_2_ ligand, followed by loss of the first proton from the amide nitrogen. For comparison, we examined protonation of the pyridine nitrogen atom of the pincer ligand. As shown in Fig. 3a, the activation energy for the protonation of the pyridine nitrogen atom is calculated to be 39.1 kcal mol^−1^, which is much higher than that of a terminal dinitrogen ligand. In conclusion, the proton transfer from LutH^+^ to **1** should first occur at one of the terminal dinitrogen ligands.

While the detachment of the proton from the NNH group in **II** easily occurs (**II**→**I**; *E*_a_=1.9 kcal mol^−1^), the protonation of **1** markedly prompts elimination of the dinitrogen ligand *trans* to the NNH group. This elimination step is exothermic by 2.7 kcal mol^−1^ (*E*_a_=4.4 kcal mol^−1^). For comparison, the elimination of an axial dinitrogen ligand in **1** is endothermic by 14.7 kcal mol^−1^ (*E*_a_=20.0 kcal mol^−1^). After the elimination of the coordinated dinitrogen ligand, OTf group, which is the counter anion of LutH^+^, will occupy the vacant coordination site of Mo in **III** to cancel electronic charge of the system (**III**+OTf^−^→**IV**; Δ*E*=–15.6 kcal mol^−1^). This mechanism is feasible since OTf group can exist in the vicinity of **III** when a terminal dinitrogen ligand in **1** is protonated. The calculated results strongly suggest that the ligand exchange process should be considered as an important part of the first protonation step.

**Figure 4:**
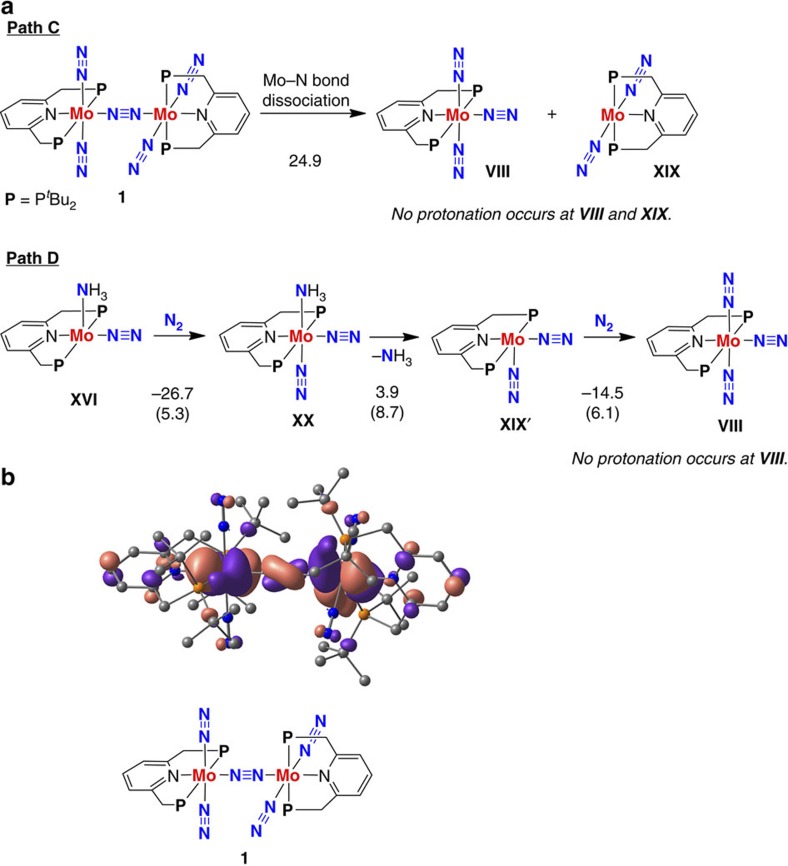
An unacceptable reaction pathway by **1**. (**a**) An unacceptable reaction pathway on the protonation of the dinitrogen ligands in the mononuclear molybdenum–dinitrogen complexes **VIII** and **XIX**, generated from **1**, (Path C) and an unsuitable reaction pathway via mononuclear complexes involving **XX** as key reactive intermediates (Path D). Energy changes and activation energies (in parenthesis) for individual reaction steps were calculated at the B3LYP*/BS2 level of theory (units in kcal mol^−1^). (**b**) Spatial distribution of the HOMO of **1**. Hydrogen atoms are omitted for clarity.

Here we should examine the previous proposed reaction pathway^37^, where **1** is first separated into [**Mo**(N_2_)_3_] **VIII** and [**Mo**(N_2_)_2_] **XIX**, and then a dinitrogen ligand in **VIII** is protonated towards formation of ammonia (Path C in Fig. 4a). The bond dissociation energy (BDE) between an Mo centre and the bridging dinitrogen ligand is calculated to be 24.9 kcal mol^−1^ for **1**, which is much higher than the energy change (+6.5 kcal mol^−1^) for the protonation of a terminal dinitrogen ligand (Figs 2a and 4a). Even if the Mo–NN bond dissociation is supposed, the dinitrogen ligands in **VIII** and **XIX** do not accept a proton from LutH^+^. We were not able to obtain any product complex consisting of a protonated **VIII** and a lutidine molecule, even starting optimization at a H^+^˙˙˙N(Lut) distance of 5 Å. Judging from the calculated results, we have newly found that the dinuclear structure remains in the first protonation step.

In the proposed catalytic mechanism, formation of [**Mo**(OTf)(≡N)] **XI** is regarded as a key reaction step. To figure out in which steps dinuclear molybdenum complexes are separated, we calculated the BDEs between one Mo centre and the bridging dinitrogen ligand for dinuclear molybdenum complexes **1**, **IV**, **V**, **VI** and **VII**. As shown in Table 2, very small BDEs were obtained for the Mo–N_α_ bond of **V** (2.1 kcal mol^−1^) and **VII** (4.4 kcal mol^−1^), and therefore these complexes should be separated into the corresponding mononuclear complexes. The small BDEs calculated for **V** and **VII** are consistent with the isolation of mononuclear molybdenum hydrazide(2−) complex [**Mo**F(NNH_2_)(C_5_H_5_N)]BF_4_ (ref. 37) and mononuclear molybdenum(IV) nitride complex [**Mo**(≡N)Cl] 3.

Towards the formation of **XI**, the NNH ligand in **IV** is first protonated/reduced to give the corresponding hydrazide(2−) complex **V**. The protonation step is exothermic by 7.4 kcal mol^−1^ with no activation barrier. We thus exclude a reaction pathway for the protonation of a terminal dinitrogen ligand bound to the other Mo centre in **IV**. In the reaction pathway via the dinuclear molybdenum–nitride complex **VII** (Path A in Fig. 2b), the third protonation to give **VI** proceeds in an exothermic way with a very low activation barrier (Δ*E*=−1.6 kcal mol^−1^, *E*_a_=1.9 kcal mol^−1^). Reduction of **VI** induces a spontaneous cleavage of the N-NH_3_ bond and leads to formation of ammonia together with the dinuclear molybdenum–nitride complex **VII**. Complex **VII** undergoes the Mo–N_α_ bond dissociation to give two mononuclear complexes **VIII** and **XI**. In the reaction pathway through the mononuclear molybdenum hydrazide(2−) complex **IX** (Path B in Fig. 2b), the generated **IX** is readily protonated (*E*_a_=2.4 kcal mol^−1^) and reduced to afford **XI** and ammonia. Experimentally, the formation of both dinuclear nitride complex bearing the dinitrogen-bridged dimolybdenum core [**Mo**(OTf)(≡N)–N≡N–**Mo**(N_2_)] and mononuclear nitride complex [**Mo**(OTf)(≡N)] was observed by mass spectrometry from the stoichiometric reaction of **1** with 2 equiv of [LutH]OTf in toluene at room temperature. This experimental result supports the proposal of **VII** and **XI** by the DFT calculation.

The isolated imide complex [**Mo**(Cl)(NH)(C_5_H_5_N)]OTf 5 has a six-coordinate structure, in which a pyridine molecule coordinates to the equatorial site of **Mo**. On the basis of this result, we propose the formation of a six-coordinate imide complex **XIII** from **XI**, where the equatorial site of **Mo** is occupied by an incoming dinitrogen molecule. Because the formation of **XIII** involves three steps such as protonation, reduction and coordination of molecular dinitrogen, there are three reaction pathways to be considered. One of them is picked up in Fig. 2c. The protonation step leading to **XII** is found to be slightly endothermic (Δ*E*=+1.7 kcal mol^−1^), followed by a highly exothermic reduction step. The coordination of molecular dinitrogen to give the six-coordinate **XIII** also proceeds in an exothermic way (Δ*E*=−4.6 kcal mol^−1^) with a low activation barrier of 4.6 kcal mol^−1^. Other two reaction pathways from **XI** to **XIII** are shown in Supplementary Fig. 5. The imide complex **XIII** is readily converted to the corresponding amide complex **XIV** via a barrierless protonation. Further protonation of **XIV** leading to an ammonia complex **XV** is almost isoenergetic (Δ*E*=0.3 kcal mol^−1^) with a moderate activation energy (*E*_a_=10.7 kcal mol^−1^).

We discuss the reaction pathway for the exchange of ammonia for molecular dinitrogen in **XV** involving regeneration of dinuclear complex **1**. As shown in Fig. 2d, reduction of **XV** induces a spontaneous elimination of the OTf group to give a five-coordinate complex **XVI** (Δ*E*=−55.9 kcal mol^−1^). The vacant coordination site in **XVI** is attacked by [**Mo**(N_2_)_3_] **VIII** to form the dinuclear molybdenum ammonia complex **XVII**. Experimentally, the formation of an ammonia complex bearing the dinitrogen-bridged dimolybdenum core [**Mo**(NH_3_)–N≡N–**Mo**(N_2_)_2_] was observed by mass spectrometry from a reaction mixture of the catalytic reaction of **1** with excess amounts of CoCp_2_ and [LutH]OTf. This experimental result supports the proposal of **XVII** by the DFT calculation. Elimination of the coordinated ammonia in **XVII** yielding [**Mo**(N_2_)–N≡N–**Mo**(N_2_)_2_] **XVIII** is endothermic by only 2.7 kcal mol^−1^ and requires an activation energy of 7.1 kcal mol^−1^. As the final step towards regeneration of **1**, a dinitrogen molecule coordinates to **XVIII** in an exothermic way (Δ*E*=−14.7 kcal mol^−1^) with a low activation energy of 5.3 kcal mol^−1^.

Next, we examined the reaction pathway involving only mononuclear complexes. In this case, as shown in Path D in Fig. 4a, the reaction pathway starts with the coordination of molecular dinitrogen into **XVI** to give the corresponding mononuclear bis(dinitrogen) complex [**Mo**(NH_3_)(N_2_)_2_] **XX**. The dissociation energy of the Mo–NH_3_ bond in **XX** is 3.9 kcal mol^−1^. The ligand exchange of ammonia for molecular dinitrogen will be attained in thermal equilibrium; however, the final product complex [**Mo**(N_2_)_3_] **VIII** can not be protonated by LutH^+^ (vide supra).

### Synergy of two molybdenum cores for catalytic ability

The calculated results clearly indicate that the mononuclear dinitrogen complex **VIII** does not serve as an active catalytic species, but that the cooperation between two molybdenum cores in dinuclear complexes plays an essential role in exhibiting the catalytic ability of **1**. In this section, we discuss the reason why the present catalytic system requires the formation of dinuclear complexes in terms of the changes in atomic charge of dinitrogen and their protonated complexes at the first protonation step.

Table 3 summarizes differences in atomic charge (Δ*q*) between dinitrogen and their protonated complexes obtained for dinuclear (**1** and **II**) and mononuclear (**VIII** and **XXI**) molybdenum complexes. The atomic charges were calculated with the natural population analysis (NPA)^52^. In the mononuclear system, the NPA charges of the molybdenum centre, the axial dinitrogen ligand, the equatorial dinitrogen ligand and the pincer ligand are increased by 0.38, 0.17, 0.09 and 0.29 after the protonation, respectively. The value of Δ*q* of NNH (+0.07) is obtained as the charge difference between the NNH group in **XXI** and the corresponding terminal dinitrogen ligand in **VIII**. The difference in the total charge is +1 for both systems since one proton is added. Comparison between Δ*q* (**II**–**1**) of unit A and Δ*q* (**XXI**–**VIII**) provides clues as to how unit B in the dinuclear system supports the protonation of the dinitrogen ligand in unit A as a mobile ligand. The values of Δ*q* calculated for the dinuclear complexes indicate that a large amount of electron (0.34e^−^) is donated from unit B to unit A by the protonation. By comparing Δ*q* (**II**–**1**) of unit A with Δ*q* (**XXI**-**VIII**), we are able to figure out that the donated electron mainly distributes on the NNH group (0.10e^−^) and the bridging dinitrogen ligand (0.15e^−^). The electron transfer between the two molybdenum cores would enhance the Brønsted basicity of the terminal dinitrogen ligand when attacked by LutH^+^. It is noteworthy that the NPA charges assigned to a terminal dinitrogen ligand in the di- and mononuclear dinitrogen complexes are almost identical (−0.12 for **1** and –0.11 for **VIII**). Tanaka *et al.*^53^ previously reported that the NPA charge on a dinitrogen ligand coordinated to a metal atom shows a good correlation with the reactivity of the metal–dinitrogen complex with a proton donor (LutH^+^). From a viewpoint of the NPA charge on dinitrogen ligands, the degree of dinitrogen activation of **1** is intrinsically insufficient for the protonation by LutH^+^. We could not calculate the proton transfer from LutH^+^ to mononuclear dinitrogen complexes such as **VIII** and **XIX**. These computational findings suggest that a terminal dinitrogen ligand coordinated to the active molybdenum site in **1** is not ‘preactivated’, but it can receive electron from the other molybdenum core via the bridging dinitrogen ligand only when necessary. Synergy of the molybdenum cores can be understood by looking at the spatial distribution of the HOMO of **1**. As depicted in Fig. 4b, the HOMO of **1** is highly delocalized between *d*-orbitals of the two molybdenum atoms via a bonding π-orbital of the bridging dinitrogen ligand. The intermetallic electron transfer stemmed from the orbital delocalization allows the dinuclear dinitrogen complex **1** to accept a proton from LutH^+^ at the first step of the catalytic formation of ammonia from molecular dinitrogen.

## Discussion

Previously we proposed a reaction pathway in which only mononuclear molybdenum–dinitrogen complexes worked as reactive intermediates. On the basis of the present experimental and theoretical studies reported here, we have proposed a new reaction pathway, where the dinuclear structure of the dinitrogen-bridged dimolybdenum–dinitrogen complex plays decisive roles in exhibiting catalytic ability for the transformation of molecular dinitrogen into ammonia. Synergy between the two molybdenum moieties connected with a bridging dinitrogen ligand has been observed at the protonation of the coordinated dinitrogen ligand. A molybdenum core donates electron to the active site of the other core through the bridging dinitrogen ligand, and thereby a terminal dinitrogen at the active site is reductively activated to accept a proton. This means that a mononuclear unit of the dinuclear molybdenum–dinitrogen complex bearing the PNP-type pincer ligands works as a mobile ligand to the other unit as an active site. This result is in sharp contrast to the common role of the dinitrogen-bridged dinuclear metal complexes bearing PNP-type and PCP-type pincer ligands, where dinitrogen-bridged dinuclear metal complexes are known to be used as precursors of mononuclear reactive metal species^54^,^55^,^56^. We consider that our new findings described in this paper provide a new opportunity to design and develop novel and more effective catalytic systems including not only the catalytic formation of ammonia from molecular dinitrogen (nitrogen fixation) but also other catalytic transformations of organic molecules by using dinitrogen-bridged dinuclear metal complexes as catalysts. In addition, we believe that the cooperative activation of molecular dinitrogen by the multinuclear metal complexes described in the present manuscript provides a mechanistic insight to elucidate the reaction pathway in the nitrogenase^3^,^4^,^5^,^6^,^7^,^8^,^9^.

## Methods

### General methods

^1^H NMR (270 MHz), ^31^P{^1^H} NMR (109 MHz), and ^15^N{^1^H} NMR (27 MHz) spectra were recorded on a JEOL Excalibur 270 spectrometer in suitable solvent, and spectra were referenced to residual solvent (^1^H) or external standard (^31^P{^1^H}: 85% H_3_PO_4_, ^15^N{^1^H}: CH_3_NO_2_). Infrared spectra were recorded on a JASCO FT/IR 4100 Fourier Transform infrared spectrometer. Absorption spectra were recorded on a Shimadzu MultiSpec-1500. Mass spectra were recorded on a JEOL Accu TOF JMS-T100LP. Elemental analyses were performed at Microanalytical Center of The University of Tokyo. All manipulations were carried out under an atmosphere of nitrogen by using standard Schlenk techniques or glovebox techniques unless otherwise stated. Solvents were dried by the usual methods, then distilled and degassed before use. NaN_2_ ^15^N (Cambridge Isotope Laboratories) was used as received. 2,6-bis(di-*tert*-butylphosphinomethyl)pyridine (**PNP**)^57^ and [MoCl_3_(thf)_3_] (ref. 58) were prepared according to the literature methods.

### Preparation of [Mo(N)Cl_2_(PNP)] (2)

A mixture of [MoCl_3_(thf)_3_] (125.0 mg, 0.30 mmol) and Me_3_SiN_3_ (42 μl, 0.32 mmol) in THF (9 ml) was stirred at 50 °C for 1 h. The resultant dark reddish brown solution was concentrated under reduced pressure. To the residue were added **PNP** (118.6 mg, 0.30 mmol) and THF (15 ml), and then the mixture was stirred at 50 °C for 4 h. After cooling at room temperature, the orange-brown cloudy solution was passed through glass filter. The solution was cooled at −40 °C to give 2·7/3C_4_H_8_O as orange crystals, which were collected by filtration and dried *in vacuo* to afford 2 (74.5 mg, 0.13 mmol, 43% yield). Anal. Calcd. for C_23_H_43_Cl_2_MoN_2_P_2_: C, 47.92; H, 7.52; N, 4.86. Found: C, 47.35; H, 7.34; N, 4.65.

### Preparation of [Mo(N)Cl(PNP)] (3)

To a suspension of 2 (57.4 mg, 0.10 mmol) in THF (5 ml) was added KC_8_ (13.7 mg, 0.10 mmol), and then the mixture was stirred at room temperature for 20 h. The solution was concentrated under reduced pressure. To the residue was added benzene (3 ml), and the solution was filtered through Celite and the filter cake was washed with benzene (1 ml × 4). The combined filtrate was concentrated to about 3 ml, slow addition of hexane (15 ml) afforded 3·1/6C_6_H_14_ as dark brown crystals, which were collected by filtration and dried *in vacuo* to afford 3 (31.5 mg, 0.06 mmol, 58% yield). ^31^P{^1^H} NMR (C_6_D_6_): *δ* 98.3 (br s). ^1^H NMR (C_6_D_6_): *δ* 6.69 (br, ArH, 3H), 3.47–3.41 (m, C*H*_2_P^*t*^Bu_2_, 2H), 3.24–3.18 (m, C*H*_2_P^*t*^Bu_2_, 2H), 1.51 (pseudo t, CH_2_P^*t*^*Bu*_2_, 18H), 1.14 (pseudo t, CH_2_P^*t*^*Bu*_2_, 18H). Infrared (C_6_D_6_, cm^−1^): 1,031 (ν_MoN_). Anal. Calcd. for C_23_H_43_ClMoN_2_P_2_: C, 51.07; H, 8.01; N, 5.18. Found: C, 50.72; H, 7.72; N, 5.04.

### Preparation of [Mo(^15^N)Cl(PNP)] (3-^15^N)

A mixture of NaN_2_ ^15^N (49.9 mg, 0.76 mmol) and Me_3_SiCl (190 μl, 1.50 mmol) in THF (3 ml) was stirred at room temperature for 24 h. The resultant white suspension was filtered through Celite and the filter cake was washed with THF (3 ml × 3). To the filtrate was added [MoCl_3_(thf)_3_] (209.3 mg, 0.50 mmol), and then the mixture was stirred at 50 °C for 1 h. The resultant dark reddish brown solution was concentrated under reduced pressure. To the residue were added **PNP** (197.4 mg, 0.50 mmol) and THF (20 ml), and then the mixture was stirred at 50 °C for 4 h. After cooling at room temperature, to the reaction mixture was added KC_8_ (67.3 mg, 0.50 mmol) and stirred at room temperature for 21 h. The solution was concentrated under reduced pressure. To the residue was added benzene (6 ml), and the solution was filtered through Celite and the filter cake was washed with benzene (2 ml × 4). The combined filtrate was concentrated to ca. 5 ml, and slow addition of hexane (15 ml) afforded 3-^15^N as dark brown crystals (42.2 mg, 0.08 mmol, 16% yield). ^15^N{^1^H} NMR (THF-*d*_8_): δ 737 (s, Mo^15^*N*). Infrared (C_6_D_6_, cm^−1^): 1,003 (ν_Mo15N_).

### Preparation of [Mo(N)Cl(PNP)]OTf (4)

To a solution of 3 (38.7 mg, 0.072 mmol) in THF (5 ml) was added AgOTf (18.5 mg, 0.072 mmol), and then the mixture was stirred at room temperature for 14 h. The solution was filtered through Celite and the filter cake was washed with THF (2 ml × 3). The combined filtrate was concentrated *in vacuo*, and the residue was washed with pentane (2 ml × 2). Recrystallization from THF (3 ml)-Et_2_O (20 ml) afforded 4 as yellow crystals (25.5 mg, 0.037 mmol, 52% yield). Calcd. for C_24_H_43_ClF_3_MoN_2_O_3_P_2_S. C, 41.77; H, 6.28; N, 4.06. Found. C, 41.55; H, 6.25; N, 3.97.

### Preparation of [Mo(NH)Cl(PNP)(C_5_H_5_N)]OTf·C_4_H_8_O (5·C_4_H_8_O)

To a solution of 3 (53.8 mg, 0.099 mmol) in C_6_H_6_ (5 ml) was added [C_5_H_5_NH]OTf (23.0 mg, 0.100 mmol), and then the mixture was stirred at room temperature for 18 h. The resultant dark green suspension was concentrated *in vacuo*. The residue was dissolved in THF (3 ml). The solution was filtered through Celite, and the filter cake was washed with THF (1 ml × 3). To the combined filtrate was slowly added Et_2_O (15 ml) to afford **5·**C_4_H_8_O as green crystals (44.2 mg, 0.052 mmol, 53% yield). ^31^P{^1^H} NMR (THF-*d*_8_): δ 73.9 (s). ^1^H NMR (THF-*d*_8_): *δ* 9.58 (d, *J*=5.1 Hz, 2H), 7.81 (t, *J*=7.1 Hz, 1H), 7.74–7.64 (m, 3H), 7.40–7.35 (m, 3H), 4.22 (dvt, *J*=16.2, 4.1Hz, C*H*_2_P^*t*^Bu_2_, 2H), 3.92 (dvt, *J*=16.2, 4.1Hz, C*H*_2_P^*t*^Bu_2_, 2H), 1.30–1.24 (m, CH_2_P^*t*^*Bu*_2_, 36H). Infrared (KBr, cm^−1^): 3126 (ν_NH_). Calcd. for C_33_H_57_ClF_3_MoN_3_O_4_P_2_S. C, 47.06; H, 6.82; N, 4.99. Found. C, 46.76; H, 6.95; N, 4.90.

### Catalytic reduction of dinitrogen to ammonia under N_2_

A typical experimental procedure for the catalytic reduction of dinitrogen into ammonia using the nitride complex 3 is described below. In a 50-ml Schlenk flask were placed 3 (11.0 mg, 0.020 mmol) and 2,6-lutidinium trifluoromethanesulphonate [LutH]OTf (247.1 mg, 0.96 mmol). Toluene (2.5 ml) was added under N_2_ (1 atm), and then a solution of CoCp_2_ (136.0 mg, 0.72 mmol) in toluene (2.5 ml) was slowly added to the stirred suspension in the Schlenk flask with a syringe pump at a rate of 2.5 ml h^−1^. After the addition of CoCp_2_, the mixture was further stirred at room temperature for 19 h. The amount of dihydrogen of the catalytic reaction was determined by GC analysis. The reaction mixture was evaporated under reduced pressure, and the distillate was trapped in dilute H_2_SO_4_ solution (0.5 M, 10 ml). Potassium hydroxide aqueous solution (30 wt%; 5 ml) was added to the residue, and the mixture was distilled into another dilute H_2_SO_4_ solution (0.5 M, 10 ml). NH_3_ present in each of the H_2_SO_4_ solutions was determined by the indophenol method^59^. The amount of ammonia was 0.020 mmol of NH_3_ collected before base distillation of the reaction mixture and 0.111 mmol of NH_3_ collected after base distillation to fully liberate NH_3_, respectively. The total amount of ammonia was 0.131 mmol (6.6 equiv per 3). No hydrazine was detected by using the *p*-(dimethylamino)benzaldehyde method^60^.

### Reaction of 3 with Cp_2_Co and [LutH]OTf under Ar

To a mixture of 3 (21.6 mg, 0.040 mmol), Cp_2_Co (30.3 mg, 0.16 mmol) and [LutH]OTf (41.3 mg, 0.16 mmol) was added toluene (5 ml) under Ar atmosphere, and the mixture was stirred at room temperature for 20 h. The reaction mixture was evaporated under reduced pressure, and the distillate was trapped in dilute H_2_SO_4_ solution (0.5 M, 10 ml). Potassium hydroxide aqueous solution (30 wt%; 5 ml) was added to the residue, and the mixture was distilled into dilute H_2_SO_4_ solution (0.5 M, 10 ml) under reduced pressure. The amount of NH_3_ in each of H_2_SO_4_ solution was determined by using the indophenol method. The total amount of NH_3_ was 0.033 mmol (0.83 equiv per 3).

### ESI-TOF-MS analysis

The reaction of **1** with 2 equiv of [LutH]OTf under N_2_ is as follows. To a mixture of **1** (11.0 mg, 0.010 mmol) and [LutH]OTf (5.3 mg, 0.021 mmol) was added toluene (1.5 ml) under N_2_ (1 atm), and the mixture was stirred at room temperature for 10 min. The resultant purple suspension was filtered and washed with toluene (1 ml × 2) and dried *in vacuo* to afford a brownish purple solid. ESI-TOF-MS of the solid in THF showed ion peaks at *m/z*=1,175.5, which were assigned as those of [Mo(N)(OTf)(**PNP**)](μ-N_2_)[Mo(**PNP**)] (*m/z*=1,175.4) and at *m/z*=656.2, which were assigned as those of [Mo(N)(OTf)(**PNP**)] (*m/z*=656.2). During the operation of the isolation of the target complexes, the decomposition of the complexes was observed.

The reaction of **1** with excess amounts of Cp_2_Co and [LutH]OTf under N_2_ is as follows. To a mixture of **1** (11.3 mg, 0.010 mmol), CoCp_2_ (45.8 mg, 0.242 mmol) and [LutH]OTf (82.2 mg, 0.320 mmol) was added toluene (2.0 mL) under N_2_ (1 atm), and the mixture was stirred at room temperature for 30 min. The resultant suspension was filtered and the filtrate was concentrated *in vacuo* to afford a blue solid. ESI-TOF-MS of the solid in THF showed ion peaks at *m/z*=1,084.5, which were assigned as those of [Mo(NH_3_)(**PNP**)](μ-N_2_)[Mo(N_2_)_2_(**PNP**)] or [Mo(NH_3_)(N_2_)(**PNP**)](μ-N_2_)[Mo(N_2_)(**PNP**)] (*m/z*=1,084.4). During the operation of the isolation of the target complex, the decomposition of the complex was observed.

### Computational methods

DFT calculations were performed to search all intermediates and transition structures on potential energy surfaces using the Gaussian 09 program^61^. To estimate the relative energy of different spin states properly, we adopted the B3LYP* functional, which is a reparametrized version of the B3LYP hybrid functional^62^,^63^ developed by Reiher and co-workers^64^,^65^. For all intermediates calculated in the present study, the minimum-energy structures have the lowest spin multiplicity (singlet or doublet). The B3LYP and B3LYP* energy expressions are given as equation (1):




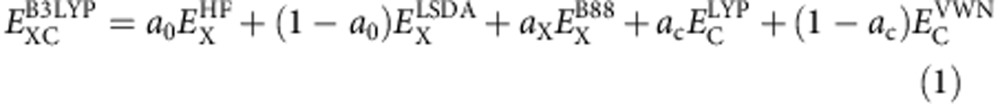




where *a*_0_=0.20 (B3LYP) or 0.15 (B3LYP*), *a*_x_=0.72, *a*_c_=0.81 and in which *E*_X_^HF^ is the Hartree-Fock exchange energy; *E*_X_^LSDA^ is the local exchange energy from the local spin density approximation; *E*_X_^B88^ is Becke’s gradient correction^66^ to the exchange functional; *E*_C_^LYP^ is the correlation functional developed by Lee *et al.*^67^; and *E*_C_^VMN^ is the correlation energy calculated using the local correlation functional of Vosko, Wilk and Nusair (VWN)^68^. For optimization, the LANL2DZ and 6–31G(d) basis sets were chosen for the Mo atom and the other atoms, respectively (BS1). All stationary-point structures were found to have the appropriate number of imaginary frequencies. To determine the energy profile of the proposed catalytic cycle, we performed single-point energy calculations at the optimized geometries using the SDD (Stuttgart/Dresden pseudopotentials) and 6-311+G(d,p) basis sets (BS2). Zero-point energy corrections were applied for energy changes (Δ*E*) and activation energies (*E*_a_) calculated for each reaction step. Solvation effects (toluene) were taken into account by using the polarizable continuum model^69^.

All protonation steps by lutidinium (LutH^+^) were assessed from a kinetic aspect by exploring reaction pathways. Energy profiles of reduction steps by cobaltocene were calculated based on the following equation, where [XH]^+^ is a protonated intermediate.




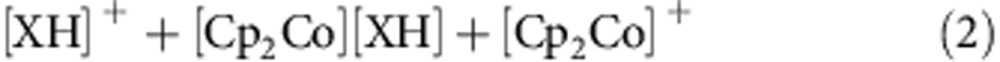




## Author contributions

K.Y. and Y.N. directed and conceived this project. K.A. and S.K. conducted the experimental work. H.T. and A.S. conducted the computational work. All authors discussed the results and wrote the manuscript.

## Additional information

**Accession codes:** The X-ray crystallographic coordinates for structures reported in this Article have been deposited at the Cambridge Crystallographic Data Centre (CCDC), under deposition number CCDC 986840, 986841, 973752, 986842. These data can be obtained free of charge from The Cambridge Crystallographic Data Centre via www.ccdc.cam.ac.uk/data_request/cif.

**How to cite this article:** Tanaka, H. *et al.* Unique behaviour of dinitrogen-bridged dimolybdenum complexes bearing pincer ligand towards catalytic formation of ammonia. *Nat. Commun.* 5:3737 doi: 10.1038/ncomms4737 (2014).

## Supplementary Material

Supplementary Figures, Tables, Methods and References Supplementary Figures 1-5, Supplementary Tables 1-74, Supplementary Methods and Supplementary References

Supplementary Data 1 Crystallographic Information File for 2

Supplementary Data 2 Crystallographic Information File for 3

Supplementary Data 3 Crystallographic Information File for 4

Supplementary Data 4 Crystallographic Information File for 5

## Figures and Tables

**Table 1:** Catalytic formation of ammonia by molybdenum complexes.*

					

**Table 2:** Bond dissociation energies.

					

**Table 3:** Differences in the NPA atomic charge (**Δ**q).

					
