# Supplementary material for: Unique behaviour of dinitrogen-bridged dimolybdenum complexes bearing pincer ligand towards catalytic formation of ammonia
Source: Nat Commun. 2014 Apr 28;5:3737. doi: 10.1038/ncomms4737 (PMC4015321; doi:10.1038/ncomms4737)
Supplement: Supplementary Figures, Tables, Methods and References — Supplementary Figures 1-5, Supplementary Tables 1-74, Supplementary Methods and Supplementary References [file ncomms4737-s1.pdf]

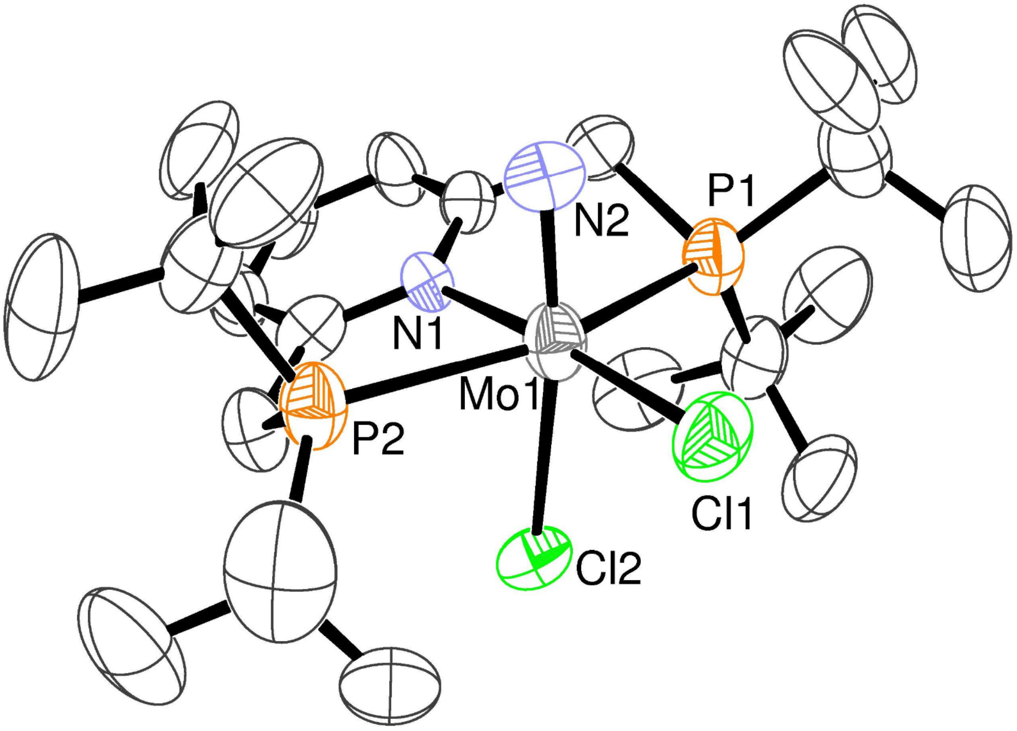

Supplementary Figure 1 | ORTEP drawing of 2. Thermal ellipsoids are shown at the 50% probability level. One of the two crystallographically disordered molecules is shown. All hydrogen atoms are omitted for clarity.

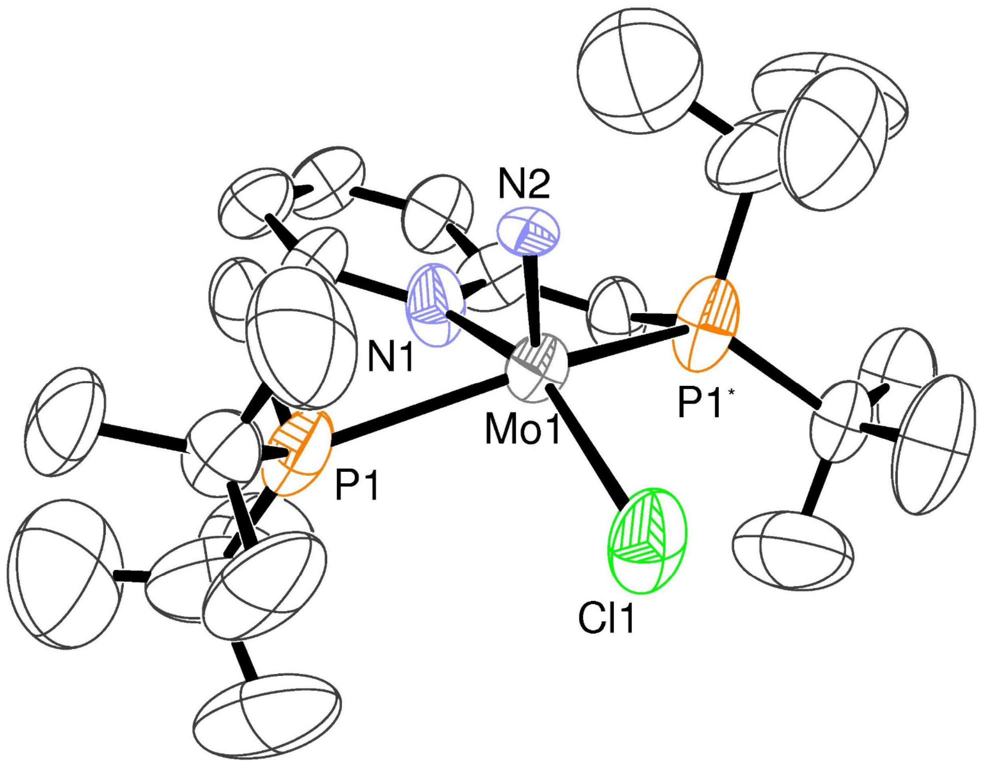

Supplementary Figure 2 | ORTEP drawing of 3. Thermal ellipsoids are shown at the 50% probability level. One of the two crystallographically disordered molecules is shown. All hydrogen atoms are omitted for clarity.

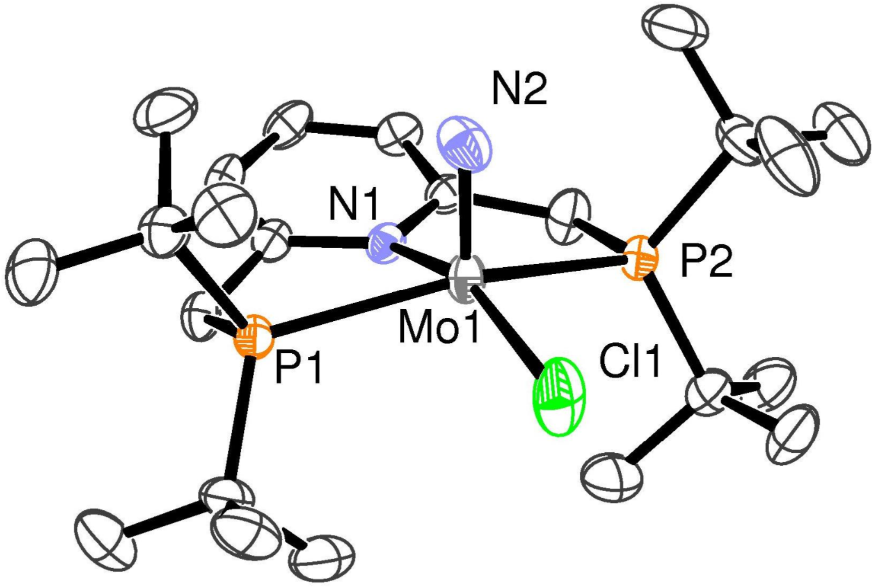

Supplementary Figure 3 | ORTEP drawing of the cationic part of 4. Thermal ellipsoids are shown at the 50% probability level. All hydrogen atoms are omitted for clarity.

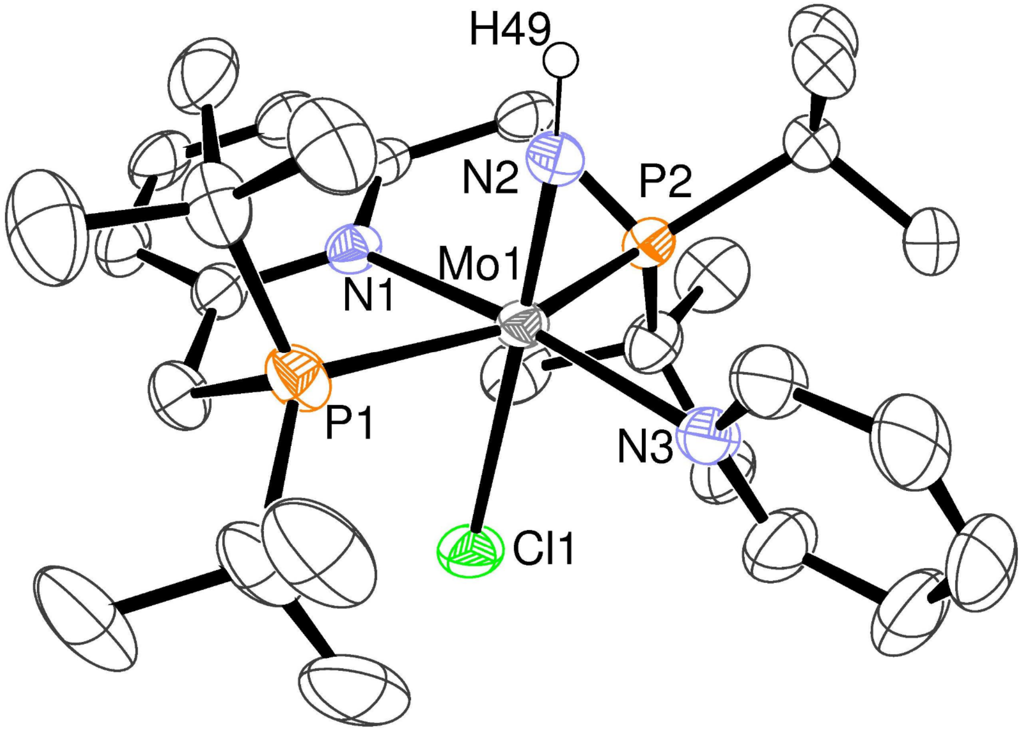

Supplementary Figure 4 | ORTEP drawing of the cationic part of 5. Thermal ellipsoids are shown at the 50% probability level. All hydrogen atoms except for H(49) are omitted for clarity.

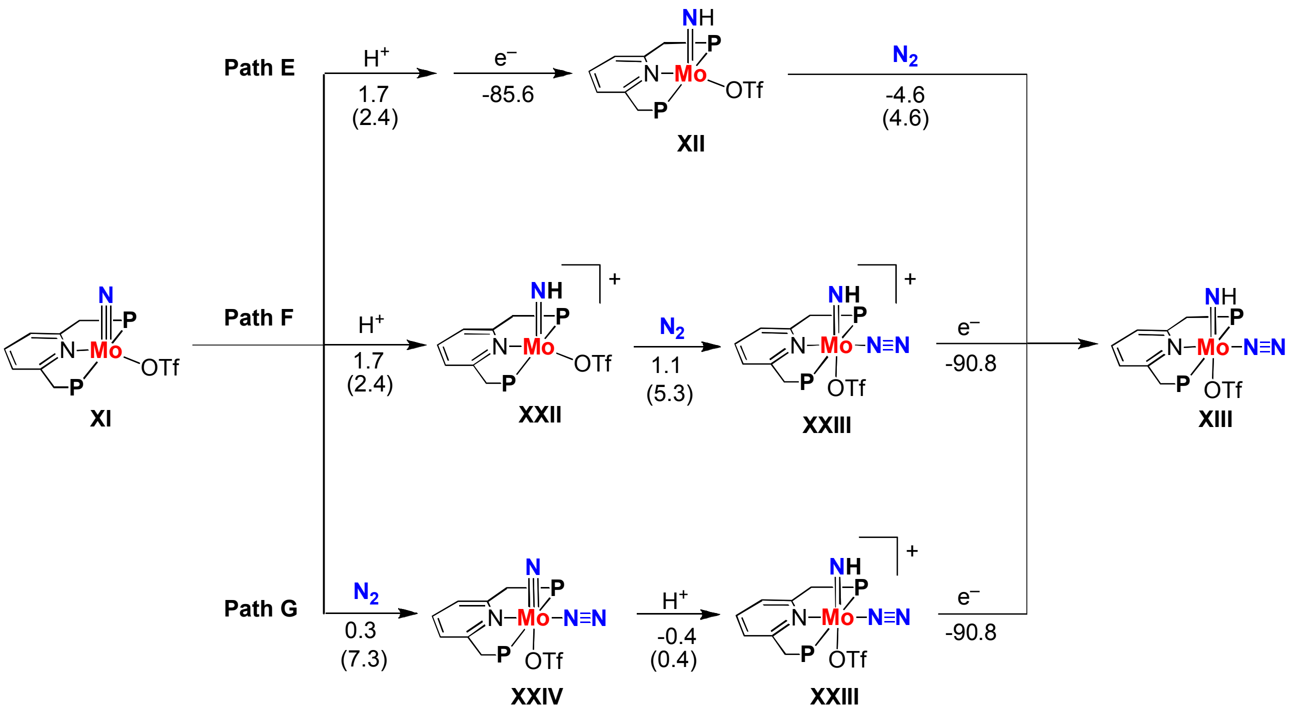

Supplementary Figure 5 | Three possible reaction pathways for formation of the six-coordinate imide complex XIII. Protons and electrons are supplied by lutidinium and cobaltocene, respectively. Energy changes and activation energies (in parenthesis) for individual reaction steps were calculated at the B3LYP*/BS2 level of theory (units in kcal/mol).

Supplementary Table 1 | X-ray Crystallographic Data for 2·7/3C_4H_8O and 3·1/6C_6H_{14}.

	**2**·7/3C_4H_8O	**3**·1/6C_6H_{14}
chemical formula	$C_{32.33}H_{61.67}Cl_2MoN_2O_{2.33}P_2$	$C_{24}H_{44.33}ClMoN_2P_2$
formula weight	744.56	554.30
dimensions of crystals	0.30 × 0.20 × 0.15	0.35 × 0.15 × 0.15
crystal system	trigonal	hexagonal
space group	$R\overline{3}$	$P6_122$
a, Å	36.248(1)	16.3159(5)
b, Å	36.248(1)	16.3159(5)
c, Å	17.0202(5)	19.6989(5)
α, deg	90	90
β, deg	90	90
γ, deg	120	120
V, Å^3	19366.9(9)	4541.4(2)
Z	18	6
ρ_{calcd}, g cm^{-3}	1.149	1.216
F(000)	7097.22	1747.98
μ, cm^{-1}	5.291	6.387
trans. factors range	0.518–0.924	0.908–0.909
no. reflections measured	49604	44063
no. unique reflections	9648 (R_{int} = 0.073)	3467 (R_{int} = 0.053)
no. parameters refined	364	168
$R1$ ($I > 2\sigma(I)$)[a]	0.0790	0.1103
$wR2$ (all data)[b]	0.1725	0.2165
GOF (all data)[c]	1.000	1.000
Flack parameters		0.10(15)
max diff peak / hole, e Å^{-3}	3.11/−1.35	1.37/−1.30
CCDC number	986840	986841

[a] $R1 = \Sigma||F_o|-|F_c||/\Sigma|F_o|$. [b] $wR2 = [\Sigma w(F_o^2-F_c^2)^2/\Sigma w(F_o^2)^2]^{1/2}$, $w = 4F_o^2/q\sigma(F_o^2)$ [q = 8.9 (**2**·7/3C_4H_8O); q = 62.2 (**3**·1/6C_6H_{14})]. [c] GOF = $[\Sigma w(F_o^2-F_c^2)^2/(N_o-N_{params})]^{1/2}$

Supplementary Table 2 | X-ray Crystallographic Data for 4 and 5·C_4H_8O.

	**4**	**5**·C_4H_8O
chemical formula	$C_{24}H_{43}ClF_3MoN_2O_3P_2S$	$C_{33}H_{57}ClF_3MoN_3O_4P_2S$
formula weight	690.01	842.23
dimensions of crystals	0.20 × 0.20 × 0.10	0.12 × 0.08 × 0.03
crystal system	monoclinic	monoclinic
space group	$C2/c$	$P2_1/c$
a, Å	33.3044(7)	16.4904(4)
b, Å	15.2043(3)	16.7621(3)
c, Å	13.0074(3)	16.7720(4)
α, deg	90	90
β, deg	109.7844(7)	119.0773(7)
γ, deg	90	90
V, Å^3	6197.8(3)	4051.7(2)
Z	8	4
ρ_{calcd}, g cm^{-3}	1.479	1.381
$F(000)$	2856	1760
μ, cm^{-1}	7.250	5.707
trans. factors range	0.692–0.930	0.778–0.983
no. reflections measured	29891	39582
no. unique reflections	7092 (R_{int} = 0.035)	9260 (R_{int} = 0.055)
no. parameters refined	377	493
$R1$ ($I > 2\sigma(I)$)[a]	0.0516	0.0449
$wR2$ (all data)[b]	0.1050	0.0718
GOF (all data)[c]	1.029	1.000
Flack parameters		
max diff peak / hole, e Å^{-3}	4.70/−1.36	0.81/–0.78
CCDC number	973752	986842

[a] $R1 = \Sigma||F_o|-|F_c||/\Sigma|F_o|$. [b] $wR2 = [\Sigma w(F_o^2-F_c^2)^2/\Sigma w(F_o^2)^2]^{1/2}$, $w = 4F_o^2/q\sigma(F_o^2)$ [q = 12.0 (**4**); q = 3.545 (**5**·C_4H_8O)]. [c] GOF = $[\Sigma w(F_o^2-F_c^2)^2/(N_o-N_{params})]^{1/2}$

Supplementary Table 3 | Selected Bond Lengths (Å) and Angles (deg) for 2·7/3C_4H_8O.

Mo(1)–N(1)	2.205(6)	P(1)–Mo(1)–N(1)	77.69(14)
Mo(1)–N(2)	1.745(12)	P(2)–Mo(1)–N(1)	77.86(14)
Mo(1)–P(1)	2.5971(19)		
Mo(1)–P(2)	2.591(3)		
Mo(1)–Cl(1)	2.407(2)		
Mo(1)–Cl(2)	2.602(4)		

Supplementary Table 4 | Selected Bond Lengths (Å) and Angles (deg) for 3·1/6C_6H_{14}.

Mo(1)–N(1)	2.202(7)	P(1)–Mo(1)–N(1)	74.92(14)
Mo(1)–N(2)	1.733(9)	P(1)*–Mo(1)–N(1)	78.14(14)
Mo(1)–P(1)	2.588(3)		
Mo(1)–P(1)*	2.437(3)		
Mo(1)–Cl(1)	2.440(3)		

Supplementary Table 5 | Selected Bond Lengths (Å) and Angles (deg) for 4.

Mo(1)–N(1)	2.227(3)	P(1)–Mo(1)–N(1)	79.30(8)
Mo(1)–N(2)	1.634(3)	P(2)–Mo(1)–N(1)	79.08(8)
Mo(1)–P(1)	2.5421(9)		
Mo(1)–P(2)	2.540(1)		
Mo(1)–Cl(1)	2.374(1)		

Supplementary Table 6 | Selected Bond lengths (Å) and Angles (deg) for 5·C_4H_8O.

Mo(1)–N(1)	2.184(3)	P(1)–Mo(1)–N(1)	78.90(6)
Mo(1)–N(2)	1.711(3)	P(2)–Mo(1)–N(1)	78.98(6)
Mo(1)–N(3)	2.225(3)	Mo(1)–N(2)–H(49)	172.3(13)
Mo(1)–P(1)	2.5209(7)		
Mo(1)–P(2)	2.5564(9)		
Mo(1)–Cl(1)	2.4993(8)		

Supplementary Table 7 | Cartesian coordinate of 1. Units are presented in Å.

SCF energy (in toluene) = -3962.57531261 hartree

ZPE = 1.284946 hartree

Atom	Coordinates (Angstroms) X	Y	Z
Mo	-2.635377403	0.091684026	-0.007458813
Mo	2.635380273	-0.091673298	-0.007498766
P	3.257938643	2.072237977	-1.172523258
P	-3.257930861	-2.072244635	-1.172454287
N	-4.874109596	0.176550058	0.016403149
N	4.874111726	-0.176565357	0.01632513
P	3.064974045	-2.293917874	1.177333274
P	-3.064977138	2.293933267	1.17736402
N	-0.575598978	0.020135817	-0.020344925
N	0.575601843	-0.020103726	-0.020353405
N	-2.580942878	1.547911994	-2.823171702
N	-2.60031816	1.014326216	-1.818570178
N	-2.628893009	-0.833551933	1.800962059
N	-2.625965989	-1.368782601	2.805023974
N	2.580906829	-1.5479145	-2.823202544
N	2.60029284	-1.014323536	-1.818603747
N	2.628930786	0.833577163	1.800914596
N	2.626024628	1.368817571	2.804971252
H	2.696071696	3.652111935	-4.699098118
H	-2.696117575	-3.652151969	-4.699024141
H	-4.060564816	-2.661077483	-4.178901924
H	4.060511247	2.661015124	-4.178997833
C	3.296519175	3.363526531	-3.822886069
C	-3.296549734	-3.363569215	-3.822800781
H	-2.399405059	-0.7702234	-3.810361023
H	2.39932748	0.770191107	-3.810385604
H	-3.801918011	-4.264748976	-3.465500605
H	3.801914063	4.264700003	-3.465607313
H	-1.110738815	-1.870053953	-4.317945243
H	1.11067494	1.870040202	-4.31796489
C	1.680139036	1.511598414	-3.447297632
C	-1.680194137	-1.511608973	-3.447273564
H	0.624030796	3.913124429	-3.262969456
H	-0.624046911	-3.913122746	-3.262942224
C	2.364157011	2.718911257	-2.774734561

C	-2.364174858	-2.718924521	-2.774678069
H	-4.560176303	-0.781957407	-2.729426349
H	4.56014255	0.781924078	-2.729508188
H	0.982272273	1.007568782	-2.771137475
H	-0.982325595	-1.007553569	-2.771134563
H	5.549454273	2.174134614	-2.27791347
H	-5.549464254	-2.174176795	-2.277804824
C	1.251025802	3.711230504	-2.382215536
C	-1.251016547	-3.711218793	-2.382172596
H	3.649490071	5.267175443	-1.615209943
H	-3.649479194	-5.267191698	-1.615101868
H	0.597053501	3.297948771	-1.603769292
H	-0.597025166	-3.297910781	-1.603756433
C	4.878942762	1.401904473	-1.878771139
C	-4.878955232	-1.401935504	-1.878679698
H	7.558132831	1.083664352	-1.623890887
H	-7.55814502	-1.08373068	-1.623750514
H	1.641724341	4.673412751	-2.034207479
H	-1.641690018	-4.673399068	-2.034131142
H	5.23316802	4.459834436	-1.64053702
H	-5.233157226	-4.459849818	-1.640408925
H	-4.510453255	2.624133323	-1.374739588
H	4.510481856	-2.624167541	-1.374750558
C	4.406524428	4.779202287	-0.992587811
C	-4.406502457	-4.779212021	-0.992471579
H	-2.644473704	5.181525873	-1.315718885
H	2.644413206	-5.181506163	-1.315747379
C	7.012103477	0.498016328	-0.887594267
C	-7.012109484	-0.498069449	-0.887469007
H	4.808315712	5.546514323	-0.312993051
H	-4.808282015	-5.546516938	-0.312861838
C	5.616357743	0.542006718	-0.883073009
C	-5.616363135	-0.542040514	-0.882974261
H	-4.886748773	4.351052054	-1.469889048
H	4.886711604	-4.351100028	-1.469898663
H	-2.028622076	3.524102423	-1.382682391
H	2.028624899	-3.524060827	-1.382738606
C	-4.716922695	3.513944395	-0.775981435
C	4.716909318	-3.513987309	-0.775991204
C	-2.347894097	4.317248985	-0.701537421
C	2.347854405	-4.31721247	-0.701579416
H	-8.775888422	0.326640621	0.058867799
H	8.775889217	-0.326708851	0.058716568
C	3.836256406	3.620069643	-0.150935903
C	-3.836219002	-3.620071328	-0.150842088
H	-5.652226727	3.350819267	-0.228012409
H	5.652212793	-3.350899953	-0.228010288

C	-7.68877878	0.284457067	0.047334331
C	7.688779955	-0.284510562	0.047203593
H	-1.484809403	4.622456071	-0.102310626
H	1.484747214	-4.622382016	-0.102365994
H	1.827338363	4.51342771	0.078845324
H	-1.827289109	-4.513414464	0.07890149
H	5.882593937	2.921943896	0.287569759
H	-5.882551063	-2.921952434	0.28770001
C	-3.549053164	3.882315662	0.166706126
C	3.549014495	-3.882316568	0.166681438
C	2.654673983	4.145881878	0.693297834
C	-2.654615044	-4.145872148	0.6933691
C	4.954992006	3.170903261	0.816182666
C	-4.954936111	-3.170900142	0.816295948
H	5.181821576	4.002417568	1.500351588
H	-5.181746191	-4.002407222	1.500480111
H	-4.376991634	5.873127686	0.339871226
H	4.376881901	-5.873156761	0.339860036
C	6.933853621	-1.011179349	0.967138457
C	-6.933844724	1.011145081	0.967247802
H	2.264192346	3.381338518	1.369957816
H	-2.264125452	-3.381322662	1.370017205
C	5.539351432	-0.94728827	0.932590247
C	-5.539342254	0.947272544	0.932673741
C	-4.015823922	5.082051319	1.014854934
C	4.015733767	-5.082066774	1.014838224
H	-4.847821782	4.825111337	1.683487594
H	4.84773233	-4.825154414	1.683480674
H	3.003867963	4.987412041	1.311296818
H	-3.0037903	-4.987402011	1.311379037
H	-7.416924854	1.635972573	1.715285786
H	7.416939428	-1.636006822	1.715172769
H	4.665735186	2.311453793	1.425216117
H	-4.665671558	-2.311441118	1.425312091
H	-1.246113548	4.771354575	2.012656388
H	1.24609735	-4.771315082	2.012685706
C	-4.716907816	1.743591673	1.915265251
C	4.716924211	-1.743588558	1.915202825
H	-3.211383954	5.516749447	1.616972266
H	3.211271746	-5.516736159	1.616947055
H	-5.317474315	2.563146789	2.33087665
H	5.317487648	-2.56314733	2.330811386
H	-0.313270445	3.323994335	1.5615421
H	0.313266444	-3.32394941	1.561563398
C	-0.921518906	3.781618296	2.351666744
C	0.921519909	-3.781569967	2.351686333
H	-4.429302673	1.095907809	2.75652926

H	4.429340797	-1.095893404	2.756465498
C	-2.096540732	2.871965886	2.762553858
C	2.096558505	-2.87192564	2.762544115
H	-0.844450964	1.066357215	2.729603618
H	0.844487748	-1.066302923	2.729597187
H	-0.266494549	3.933147486	3.221958516
H	0.26650521	-3.933079103	3.221989054
C	-1.490711912	1.61647054	3.42072228
C	1.49075242	-1.616418441	3.420710838
H	-3.414049403	4.51126643	3.46856709
H	3.414062116	-4.511232835	3.468549423
H	-3.764541359	2.933715902	4.199943545
H	3.764580596	-2.933679954	4.199908561
C	-2.962559741	3.581635653	3.824524777
C	2.962587035	-3.581594904	3.824507218
H	-0.879998808	1.929040155	4.28097385
H	0.880048	-1.928974504	4.28097355
H	-2.254595036	0.92730038	3.795014127
H	2.254648186	-0.927253636	3.794986817
H	-2.32768707	3.834931507	4.687340775
H	2.327724935	-3.834877829	4.687334779

Supplementary Table 8 | Cartesian coordinate of II. Units are presented in Å.

SCF energy (in toluene) = -3963.00659617 hartree

ZPE = 1.297034 hartree

Atom	Coordinates (Angstroms) X	Y	Z
Mo	-2.98085911	1.864639987	1.86942464
Mo	2.220665373	1.739301191	1.510909153
P	2.734184325	-0.686299738	2.23904729
P	-3.197099134	3.467708845	3.860766448
N	-5.229112399	1.942802453	2.199778669
N	4.408393943	1.559252753	1.008597744
P	2.744380469	4.06589171	0.584163906
P	-3.869188334	0.294304381	0.027172426
N	-1.007800911	1.811440824	1.643876166
N	0.150379146	1.781050891	1.576673071
N	-2.75736925	-0.58357741	3.870999807
N	-2.842218067	0.296187449	3.15965413
N	-3.099896775	3.426402951	0.563973123
N	-3.161053727	4.29728711	-0.160294839
N	2.901014311	2.626319517	4.359790493
N	2.544020331	2.277613717	3.233504784
N	1.820231798	1.058343241	-0.566577203
N	1.556737193	0.710809814	-1.59607928
H	2.649923429	-2.708290659	5.556130289
H	-1.999332633	3.519133372	7.566711604
H	-3.540491407	2.923961697	6.94918469
H	4.000191393	-1.77499332	4.910912508
C	3.105719571	-2.341843494	4.624799699
C	-2.733037333	3.640878941	6.756104192
H	-2.22433727	1.182262978	5.651792925
H	2.557461068	0.292657682	5.135118625
H	-3.144108524	4.649537654	6.840697885
H	3.421856595	-3.216815027	4.052631947
H	-0.741017405	1.882464741	6.302847438
H	1.248728228	-0.731873863	5.744322272
C	1.691567111	-0.305466642	4.832831219
C	-1.455568743	1.940004292	5.467964545
H	0.333720112	-2.552773524	4.569172146
H	-0.10192541	4.140135152	6.017888186
C	2.07327147	-1.461468325	3.888688276

C	-2.030202386	3.368141406	5.40868768
H	-4.451514604	1.768438283	5.019500283
H	4.444626678	0.284742808	3.623235971
H	0.953157559	0.36088145	4.375951895
H	-0.929974154	1.673017284	4.545463672
H	5.146356763	-1.145830173	2.855407239
H	-5.290917533	3.299568061	5.26415496
C	0.788296403	-2.269236209	3.609515044
C	-0.842991465	4.335739966	5.22755985
H	2.752604942	-3.925605828	2.164482209
H	-3.096546772	6.268604413	5.490407632
H	0.043542094	-1.677287644	3.062305018
H	-0.345950439	4.194624454	4.258457027
C	4.518925531	-0.265029448	2.673087499
C	-4.781544548	2.685973805	4.511042652
H	7.129046774	-0.225295572	1.927932858
H	-7.491549745	2.672447722	4.608333866
H	0.975007516	-3.195420435	3.056308711
H	-1.132718661	5.387499701	5.316566844
H	4.413040455	-3.312092474	1.99884435
H	-4.757068401	5.630830551	5.485473577
H	-4.994702291	-0.920052766	2.500455945
H	4.597461999	3.779453457	2.940458551
C	3.452306465	-3.433344635	1.482895356
C	-3.984806555	6.110150437	4.870425126
H	-3.477218981	-3.34821978	1.16300632
H	3.137789099	6.429035409	3.562750925
C	6.544153048	0.519277858	1.393980901
C	-7.121897513	2.371647736	3.631068085
H	3.622125449	-4.122014872	0.642432801
H	-4.365354074	7.104391137	4.593773228
C	5.175502389	0.631471159	1.652555065
C	-5.744015603	2.320833873	3.405849793
H	-5.573119033	-2.506073112	1.978188018
H	5.273924617	5.401426805	3.10453438
H	-2.649358149	-1.89517779	1.736822772
H	2.296900087	4.876665899	3.445967282
C	-5.383362626	-1.475959899	1.643837099
C	4.840973263	4.68969139	2.386991896
C	-3.156435853	-2.336339888	0.874635931
C	2.621950437	5.747463098	2.870865723
H	-9.079560869	2.079396644	2.761338178
H	8.204030094	1.276087892	0.236940081
C	2.911253854	-2.10119192	0.924922545
C	-3.684295208	5.320548214	3.580299506
H	-6.350671298	-1.044143315	1.362362236
H	5.620776709	4.464872299	1.649615794

C	-8.0038341	2.041232199	2.60445895
C	7.141349314	1.357577805	0.455276912
H	-2.427972391	-2.444197571	0.065240435
H	1.735420079	6.269170122	2.498754791
H	0.804058768	-2.757993905	0.9804819
H	-1.628110648	6.106395639	3.388847566
H	4.935419874	-1.616932406	0.201880893
H	-5.841299865	4.98880133	3.264320932
C	-4.400110962	-1.522340669	0.451230794
C	3.60123452	5.352178337	1.742614803
C	1.533107597	-2.353061138	0.272820587
C	-2.551373875	6.030511834	2.806187686
C	3.904270012	-1.644222784	-0.168053393
C	-4.96577378	5.35618659	2.716662011
H	3.875130665	-2.369910836	-0.993310408
H	-5.170520971	6.400241176	2.43979189
H	-5.485673742	-3.227095556	-0.313696349
H	4.662135485	7.232729388	1.73310105
C	6.356050715	2.309369259	-0.19044718
C	-7.47643983	1.66164118	1.372312631
H	1.106392333	-1.443588507	-0.15994332
H	-2.320629512	5.527337229	1.863558224
C	4.994543911	2.404262451	0.112024015
C	-6.091148161	1.614175043	1.194666346
C	-5.117897901	-2.263180003	-0.694856138
C	4.080998762	6.627976582	1.021995701
H	-5.989414779	-1.711963744	-1.069991298
H	4.739966411	6.412913826	0.170876204
H	1.647785829	-3.086517738	-0.538408777
H	-2.871122636	7.055122854	2.566054596
H	-8.129551132	1.398254219	0.543933744
H	6.790771067	2.990663244	-0.917412355
H	3.67044992	-0.662599153	-0.587466879
H	-4.872258431	4.782925416	1.791210047
H	-2.494105966	-1.755994103	-1.964354549
H	1.086320584	6.769076075	0.549461836
C	-5.512218174	1.200475542	-0.136906061
C	4.145801739	3.47059274	-0.537476974
H	-4.457267254	-2.483311193	-1.539311282
H	3.254030286	7.253426037	0.671322641
H	-6.259758464	0.639245463	-0.710854363
H	4.786498843	4.285085963	-0.896858537
H	-1.335441092	-0.653328522	-1.187482514
H	0.098916526	5.331028539	0.906345808
C	-2.102969597	-0.733569397	-1.968604544
C	0.607169742	5.88846879	0.108985396
H	-5.27498317	2.102703874	-0.719705214

H	3.643950382	3.058537561	-1.425246965
C	-3.206284911	0.330557169	-1.796227296
C	1.598700636	4.999204853	-0.669230444
H	-1.772032619	1.923807687	-1.305770644
H	0.194269341	3.307451637	-0.74284433
H	-1.606368778	-0.580185472	-2.937394672
H	-0.166701049	6.251117774	-0.582106506
C	-2.560384919	1.709405949	-2.034463556
C	0.773485917	3.938133326	-1.424656943
H	-4.797985635	-0.828892398	-2.820467607
H	2.919616317	6.66787274	-1.301982623
H	-5.058197785	0.92589478	-2.830383086
H	3.021785197	5.232080489	-2.338895093
C	-4.295182989	0.139122772	-2.874106868
C	2.345512128	5.841023281	-1.726016563
H	-2.111811662	1.718461773	-3.038743404
H	0.064674479	4.451176417	-2.090020123
H	-3.288335104	2.526645554	-1.999329646
H	1.398303341	3.297148051	-2.057552803
H	-3.823317222	0.207974293	-3.86528051
H	1.604104465	6.27790937	-2.410644432
H	2.094948989	2.809592234	4.989814654

Supplementary Table 9 | Cartesian coordinate of III. Units are presented in Å.

SCF energy (in toluene) = -3853.50852839 hartree

ZPE = 1.286557 hartree

Atom	X	Y	Z
Mo	-2.635377403	0.091684026	-0.007458813
Mo	2.635380273	-0.091673298	-0.007498766
P	3.257938643	2.072237977	-1.172523258
P	-3.257930861	-2.072244635	-1.172454287
N	-4.874109596	0.176550058	0.016403149
N	4.874111726	-0.176565357	0.01632513
P	3.064974045	-2.293917874	1.177333274
P	-3.064977138	2.293933267	1.17736402
N	-0.575598978	0.020135817	-0.020344925
N	0.575601843	-0.020103726	-0.020353405
N	-2.580942878	1.547911994	-2.823171702
N	-2.60031816	1.014326216	-1.818570178
N	-2.628893009	-0.833551933	1.800962059
N	-2.625965989	-1.368782601	2.805023974
N	2.580906829	-1.5479145	-2.823202544
N	2.60029284	-1.014323536	-1.818603747
N	2.628930786	0.833577163	1.800914596
N	2.626024628	1.368817571	2.804971252
H	2.696071696	3.652111935	-4.699098118
H	-2.696117575	-3.652151969	-4.699024141
H	-4.060564816	-2.661077483	-4.178901924
H	4.060511247	2.661015124	-4.178997833
C	3.296519175	3.363526531	-3.822886069
C	-3.296549734	-3.363569215	-3.822800781
H	-2.399405059	-0.7702234	-3.810361023
H	2.39932748	0.770191107	-3.810385604
H	-3.801918011	-4.264748976	-3.465500605
H	3.801914063	4.264700003	-3.465607313
H	-1.110738815	-1.870053953	-4.317945243
H	1.11067494	1.870040202	-4.31796489
C	1.680139036	1.511598414	-3.447297632
C	-1.680194137	-1.511608973	-3.447273564
H	0.624030796	3.913124429	-3.262969456
H	-0.624046911	-3.913122746	-3.262942224
C	2.364157011	2.718911257	-2.774734561

C	-2.364174858	-2.718924521	-2.774678069
H	-4.560176303	-0.781957407	-2.729426349
H	4.56014255	0.781924078	-2.729508188
H	0.982272273	1.007568782	-2.771137475
H	-0.982325595	-1.007553569	-2.771134563
H	5.549454273	2.174134614	-2.27791347
H	-5.549464254	-2.174176795	-2.277804824
C	1.251025802	3.711230504	-2.382215536
C	-1.251016547	-3.711218793	-2.382172596
H	3.649490071	5.267175443	-1.615209943
H	-3.649479194	-5.267191698	-1.615101868
H	0.597053501	3.297948771	-1.603769292
H	-0.597025166	-3.297910781	-1.603756433
C	4.878942762	1.401904473	-1.878771139
C	-4.878955232	-1.401935504	-1.878679698
H	7.558132831	1.083664352	-1.623890887
H	-7.55814502	-1.08373068	-1.623750514
H	1.641724341	4.673412751	-2.034207479
H	-1.641690018	-4.673399068	-2.034131142
H	5.23316802	4.459834436	-1.64053702
H	-5.233157226	-4.459849818	-1.640408925
H	-4.510453255	2.624133323	-1.374739588
H	4.510481856	-2.624167541	-1.374750558
C	4.406524428	4.779202287	-0.992587811
C	-4.406502457	-4.779212021	-0.992471579
H	-2.644473704	5.181525873	-1.315718885
H	2.644413206	-5.181506163	-1.315747379
C	7.012103477	0.498016328	-0.887594267
C	-7.012109484	-0.498069449	-0.887469007
H	4.808315712	5.546514323	-0.312993051
H	-4.808282015	-5.546516938	-0.312861838
C	5.616357743	0.542006718	-0.883073009
C	-5.616363135	-0.542040514	-0.882974261
H	-4.886748773	4.351052054	-1.469889048
H	4.886711604	-4.351100028	-1.469898663
H	-2.028622076	3.524102423	-1.382682391
H	2.028624899	-3.524060827	-1.382738606
C	-4.716922695	3.513944395	-0.775981435
C	4.716909318	-3.513987309	-0.775991204
C	-2.347894097	4.317248985	-0.701537421
C	2.347854405	-4.31721247	-0.701579416
H	-8.775888422	0.326640621	0.058867799
H	8.775889217	-0.326708851	0.058716568
C	3.836256406	3.620069643	-0.150935903
C	-3.836219002	-3.620071328	-0.150842088
H	-5.652226727	3.350819267	-0.228012409
H	5.652212793	-3.350899953	-0.228010288

C	-7.68877878	0.284457067	0.047334331
C	7.688779955	-0.284510562	0.047203593
H	-1.484809403	4.622456071	-0.102310626
H	1.484747214	-4.622382016	-0.102365994
H	1.827338363	4.51342771	0.078845324
H	-1.827289109	-4.513414464	0.07890149
H	5.882593937	2.921943896	0.287569759
H	-5.882551063	-2.921952434	0.28770001
C	-3.549053164	3.882315662	0.166706126
C	3.549014495	-3.882316568	0.166681438
C	2.654673983	4.145881878	0.693297834
C	-2.654615044	-4.145872148	0.6933691
C	4.954992006	3.170903261	0.816182666
C	-4.954936111	-3.170900142	0.816295948
H	5.181821576	4.002417568	1.500351588
H	-5.181746191	-4.002407222	1.500480111
H	-4.376991634	5.873127686	0.339871226
H	4.376881901	-5.873156761	0.339860036
C	6.933853621	-1.011179349	0.967138457
C	-6.933844724	1.011145081	0.967247802
H	2.264192346	3.381338518	1.369957816
H	-2.264125452	-3.381322662	1.370017205
C	5.539351432	-0.94728827	0.932590247
C	-5.539342254	0.947272544	0.932673741
C	-4.015823922	5.082051319	1.014854934
C	4.015733767	-5.082066774	1.014838224
H	-4.847821782	4.825111337	1.683487594
H	4.84773233	-4.825154414	1.683480674
H	3.003867963	4.987412041	1.311296818
H	-3.0037903	-4.987402011	1.311379037
H	-7.416924854	1.635972573	1.715285786
H	7.416939428	-1.636006822	1.715172769
H	4.665735186	2.311453793	1.425216117
H	-4.665671558	-2.311441118	1.425312091
H	-1.246113548	4.771354575	2.012656388
H	1.24609735	-4.771315082	2.012685706
C	-4.716907816	1.743591673	1.915265251
C	4.716924211	-1.743588558	1.915202825
H	-3.211383954	5.516749447	1.616972266
H	3.211271746	-5.516736159	1.616947055
H	-5.317474315	2.563146789	2.33087665
H	5.317487648	-2.56314733	2.330811386
H	-0.313270445	3.323994335	1.5615421
H	0.313266444	-3.32394941	1.561563398
C	-0.921518906	3.781618296	2.351666744
C	0.921519909	-3.781569967	2.351686333
H	-4.429302673	1.095907809	2.75652926

H	4.429340797	-1.095893404	2.756465498
C	-2.096540732	2.871965886	2.762553858
C	2.096558505	-2.87192564	2.762544115
H	-0.844450964	1.066357215	2.729603618
H	0.844487748	-1.066302923	2.729597187
H	-0.266494549	3.933147486	3.221958516
H	0.26650521	-3.933079103	3.221989054
C	-1.490711912	1.61647054	3.42072228
C	1.49075242	-1.616418441	3.420710838
H	-3.414049403	4.51126643	3.46856709
H	3.414062116	-4.511232835	3.468549423
H	-3.764541359	2.933715902	4.199943545
H	3.764580596	-2.933679954	4.199908561
C	-2.962559741	3.581635653	3.824524777
C	2.962587035	-3.581594904	3.824507218
H	-0.879998808	1.929040155	4.28097385
H	0.880048	-1.928974504	4.28097355
H	-2.254595036	0.92730038	3.795014127
H	2.254648186	-0.927253636	3.794986817
H	-2.32768707	3.834931507	4.687340775
H	2.327724935	-3.834877829	4.687334779

Supplementary Table 10 | Cartesian coordinate of IV. Units are presented in Å.

SCF energy (in toluene) = -4814.96151136 hartree

ZPE = 1.315695 hartree

Atom	X	Y	Z
Mo	-2.892002857	-0.043947612	0.008716323
Mo	2.298961332	0.581972042	0.422737997
P	2.865370221	-0.755980831	2.56707277
P	-3.572161518	2.388794852	0.468304394
N	-5.15232017	-0.141546221	-0.02188653
N	4.507776474	0.864377112	0.55198341
P	2.743287263	2.060776804	-1.607687564
P	-3.323209889	-2.515115106	-0.491316566
N	-0.874523202	0.047446495	0.076986685
N	0.275667019	0.121685667	0.141845048
N	-2.934428929	-0.595064937	3.131652597
N	-2.915948026	-0.394446364	2.013558067
N	-2.855246014	0.324839904	-1.994233371
N	-2.844770586	0.547220484	-3.107823427
N	1.724720741	3.02809401	2.141604095
N	1.99623111	2.02177399	1.450189986
H	1.804803082	-0.494624946	6.298779232
H	-3.115085588	4.943395904	3.375803124
H	-4.453398706	3.815486883	3.146310785
H	3.174484108	0.296944851	5.514588427
C	2.524495692	-0.586738482	5.471246438
C	-3.692950283	4.400038855	2.613071771
H	-2.757083296	1.954586574	3.389835209
H	1.482625305	1.540224763	4.039308305
H	-4.203335526	5.146932327	1.99938395
H	3.136026524	-1.47053859	5.667687348
H	-1.454046313	3.146262848	3.528782509
H	0.206448289	0.658412882	4.898887559
C	0.889756747	0.621112907	4.0371251
C	-2.039731719	2.550407246	2.814655617
H	0.015777095	-1.691364745	4.931451388
H	-1.000682204	4.80140615	1.948357483
C	1.744758499	-0.653908595	4.141503495
C	-2.725596749	3.499946972	1.815838669
H	-4.865196874	1.573024161	2.32252891

H	3.830786203	1.31086841	3.351720114
H	0.286679806	0.626111344	3.125303392
H	-1.350482929	1.870205017	2.308495488
H	4.960675744	0.018759124	3.774028735
H	-5.8653867	2.764011661	1.487388416
C	0.767245044	-1.849237614	4.144190295
C	-1.622943481	4.354282072	1.16120257
H	3.175738777	-3.322407034	4.538389945
H	-4.043867337	5.551188133	-0.070426425
H	0.227469979	-1.92757397	3.191728057
H	-0.955274653	3.745276343	0.540685792
C	4.309631927	0.394855303	2.975089139
C	-5.183421503	1.919055539	1.328074325
H	6.996698251	0.806697872	2.836974961
H	-7.858577946	1.511714942	1.165851612
H	1.253555051	-2.808968383	4.343758549
H	-2.02642596	5.170909465	0.552244428
H	4.730669991	-2.460559708	4.486845403
H	-5.609327352	4.742993051	0.175586785
H	-4.80710113	-2.08806533	2.038742476
H	3.877104202	3.588245963	0.670192432
C	4.048167576	-3.103526773	3.915405809
C	-4.777359334	4.879512911	-0.527537846
H	-2.911052652	-4.507366697	2.754336567
H	1.935688798	5.706292669	-0.702718721
C	6.518969719	0.916169361	1.866476583
C	-7.30137785	0.743152235	0.635102333
H	4.572302734	-4.056252269	3.746265785
H	-5.183238734	5.397169602	-1.41006658
C	5.138795706	0.733340535	1.755411554
C	-5.9055097	0.799527629	0.622616365
H	-5.172507994	-3.716495577	2.623494617
H	4.170481766	5.210426597	0.015709767
H	-2.294675168	-2.909656752	2.306817651
H	1.411415319	4.215633701	0.102968315
C	-4.996326771	-3.115085678	1.719219601
C	4.114292197	4.142050893	-0.24154955
C	-2.613451122	-3.874776089	1.904218051
C	1.743871313	4.632159451	-0.853256355
H	-9.048106351	-0.342802113	-0.036785068
H	8.34790918	1.339367559	0.797016169
C	3.691867708	-2.503859585	2.540129948
C	-4.162210517	3.540285351	-0.979869094
H	-5.922999813	-3.128427026	1.133538262
H	5.111954741	3.837116243	-0.577176638
C	-7.961551963	-0.286553933	-0.032690393
C	7.268878328	1.213969561	0.729576398

H	-1.748507449	-4.347937384	1.429959378
H	0.926500175	4.547132224	-1.574591183
H	1.811412097	-3.639357899	2.351035079
H	-2.186598995	4.415481425	-1.4356143
H	5.779907178	-1.855721666	2.325746392
H	-6.17612879	2.674263599	-1.214851953
C	-3.812560888	-3.731274934	0.940526324
C	3.044472163	3.95772701	-1.341724974
C	2.735161024	-3.455443549	1.790701406
C	-2.971036693	3.826890149	-1.920439638
C	5.013875199	-2.394963534	1.754408992
C	-5.248910311	2.794584083	-1.78688463
H	5.396208182	-3.407493789	1.565822817
H	-5.491080574	3.389531722	-2.68012609
H	-4.624190484	-5.69113494	1.357622498
H	3.818240446	5.7265619	-2.316492193
C	6.615822051	1.356167239	-0.490940867
C	-7.19372578	-1.242423169	-0.694909579
H	2.470047355	-3.056497936	0.808993835
H	-2.520991094	2.907133696	-2.303020494
C	5.226512585	1.198286207	-0.558462772
C	-5.799611067	-1.155091091	-0.671790633
C	-4.259979369	-5.133527483	0.481442922
C	3.568775928	4.690973432	-2.593829055
H	-5.085171697	-5.095537158	-0.241832966
H	4.483923457	4.234641482	-2.992712917
H	3.231454963	-4.424901139	1.638614235
H	-3.330113001	4.404819369	-2.785495469
H	-7.664868315	-2.06412093	-1.229376695
H	7.16739421	1.600185415	-1.395342349
H	4.891270536	-1.927803833	0.7777166
H	-4.923416993	1.808882346	-2.126597708
H	-1.502987287	-5.104730729	-0.550679555
H	1.01444108	3.834034096	-3.622565842
C	-4.96203239	-2.1939429	-1.371350209
C	4.498845443	1.418414782	-1.863245614
H	-3.444857625	-5.718716745	0.044538334
H	2.832680344	4.74224554	-3.401378715
H	-5.55645342	-3.099573619	-1.545879415
H	5.10442887	2.056886604	-2.519367949
H	-0.559766185	-3.595312431	-0.574359217
H	0.012969022	2.721705819	-2.659758062
C	-1.179053883	-4.268422375	-1.179617061
C	0.713063523	2.788311927	-3.502188507
H	-4.664877116	-1.811674068	-2.358959145
H	4.378700991	0.45474841	-2.379698483
C	-2.357929841	-3.524786481	-1.837659263

C	1.899716662	1.816268782	-3.337461119
H	-1.133544132	-1.7727566	-2.350327278
H	0.632568607	0.211880047	-2.531969064
H	-0.530238746	-4.68099325	-1.962705926
H	0.155689595	2.505275179	-4.406854382
C	-1.772206238	-2.513026258	-2.8412987
C	1.317688346	0.389254107	-3.367191744
H	-3.667994741	-5.306725739	-2.0221089
H	3.344072946	2.945146826	-4.586692975
H	-4.035218561	-4.014221137	-3.181093834
H	3.663930032	1.19631625	-4.485510596
C	-3.226844584	-4.518824075	-2.637236229
C	2.876123618	1.957515514	-4.523467965
H	-1.156325429	-3.055966732	-3.57083821
H	0.750459976	0.264854724	-4.30226203
H	-2.549717019	-1.981737258	-3.40053394
H	2.100537067	-0.369204446	-3.351426202
H	-2.592175752	-5.008176758	-3.391009415
H	2.319292625	1.797603173	-5.459112633
H	2.579411752	3.560674164	2.384499974
S	3.690710052	-2.177685502	-1.883237592
O	4.049212292	-1.458647926	-3.128907923
O	4.754152107	-2.982945827	-1.26294874
O	2.88557772	-1.341778779	-0.912410411
C	2.43674017	-3.439418835	-2.440391205
F	2.033727222	-4.199042224	-1.404122444
F	1.35229646	-2.845543614	-2.971149231
F	2.977131221	-4.241022756	-3.366924845

Supplementary Table 11 | Cartesian coordinate of V. Units are presented in Å.

SCF energy (in toluene) = -4815.53322129 hartree

ZPE = 1.328643 hartree

Atom	Coordinates (Angstroms) X	Y	Z
Mo	-2.901799164	-0.029739246	0.132061097
Mo	2.305167097	0.493603219	0.501869568
P	2.871768509	-1.34334194	2.256842318
P	-3.762531934	2.331176864	0.618290635
N	-5.144147229	-0.253853427	-0.095248925
N	4.534580918	0.70342239	0.693671936
P	2.816170713	2.419107316	-1.157128854
P	-3.167444741	-2.512067701	-0.444817183
N	-0.870499179	0.143639912	0.257653796
N	0.285455193	0.241315821	0.293560797
N	-3.045300509	-0.692653297	3.22743921
N	-2.999174818	-0.44885138	2.118035266
N	-2.75137532	0.386225296	-1.855772708
N	-2.661787737	0.625291237	-2.962033331
N	1.020138277	2.353469977	2.374813166
N	2.090552171	1.720475592	1.883883048
H	1.881365942	-1.981608017	5.963012485
H	-3.822983747	4.839262721	3.615656367
H	-5.039467891	3.62350399	3.225672084
H	3.302055993	-1.118607965	5.366317126
C	2.584195574	-1.911359228	5.11900173
C	-4.27061166	4.270439677	2.786415115
H	-3.286940746	1.858226346	3.607414723
H	1.706485171	0.57859524	4.283954684
H	-4.763989735	4.989367293	2.12807793
H	3.125603723	-2.85823892	5.06668905
H	-2.121935911	3.137078769	3.963255135
H	0.397238752	-0.390267101	4.9877857
C	1.031878069	-0.263419632	4.097350262
C	-2.544787245	2.523329339	3.153467709
H	0.010467707	-2.688811107	4.398426618
H	-1.578909492	4.890139132	2.472071253
C	1.786692248	-1.579660318	3.839810072
C	-3.159696789	3.454462562	2.089991657
H	-5.19297553	1.387500827	2.313611586

H	3.873382367	0.44903274	3.503221502
H	0.39009061	0.013321613	3.25779056
H	-1.745402625	1.895808869	2.747864522
H	4.983742549	-0.933124628	3.593748532
H	-6.161935294	2.557013512	1.412263599
C	0.728420471	-2.668145935	3.565415877
C	-2.052341012	4.410731988	1.60194809
H	3.098355481	-4.341228043	3.497578535
H	-4.349722852	5.494534897	0.109869756
H	0.162704721	-2.451307522	2.650562282
H	-1.26788198	3.881197608	1.045547819
C	4.338259194	-0.356713918	2.919119374
C	-5.425746575	1.750703272	1.301568898
H	7.023231049	0.029299561	2.881796043
H	-8.040885291	1.228799915	0.849622807
H	1.156706559	-3.671833211	3.475335165
H	-2.434139027	5.212838649	0.961263425
H	4.679062586	-3.540126133	3.661922421
H	-5.884212036	4.599239485	0.180431253
H	-4.855733229	-2.183210918	1.991089816
H	3.74412522	3.372608567	1.499842163
C	3.978545923	-3.99264321	2.948011077
C	-4.995563698	4.796619189	-0.432579149
H	-3.006277367	-4.594104254	2.775265781
H	2.10799783	5.811238716	0.523249181
C	6.551136879	0.388309755	1.970407832
C	-7.395569259	0.505807233	0.355800303
H	4.473714743	-4.884204623	2.534882471
H	-5.33890625	5.315817393	-1.340087325
C	5.168027344	0.265191573	1.820225713
C	-6.010913585	0.625017672	0.493041258
H	-5.255814653	-3.83921292	2.467361255
H	4.230357912	5.064293168	1.210749505
H	-2.37750821	-2.975226501	2.439596245
H	1.372237078	4.221208957	0.798184207
C	-5.006768746	-3.196005665	1.610020041
C	4.124184197	4.071647329	0.746366934
C	-2.641862447	-3.929604219	1.977054454
C	1.862844811	4.848669435	0.048404293
H	-9.00871081	-0.630635853	-0.534349658
H	8.390127621	1.042804086	1.041749017
C	3.642403685	-3.049004991	1.776698388
C	-4.270847158	3.501537991	-0.848882839
H	-5.878781892	-3.188243926	0.945901966
H	5.127194877	3.747931227	0.446675907
C	-7.93280372	-0.525516264	-0.412144452
C	7.30960949	0.953441269	0.94686088

H	-1.730200117	-4.37668142	1.570193643
H	1.144495218	5.054622087	-0.749031604
H	1.716759563	-4.017176129	1.314949157
H	-2.28950756	4.452556483	-1.067405519
H	5.743454348	-2.410804618	1.71720617
H	-6.214735782	2.571317391	-1.31132083
C	-3.751593516	-3.770415808	0.914173792
C	3.16676878	4.196210817	-0.461032852
C	2.657532682	-3.750699036	0.817505482
C	-3.00531458	3.868325465	-1.654442539
C	4.966652163	-2.778262744	1.034879304
C	-5.236844256	2.732766581	-1.779532584
H	5.326727484	-3.721018054	0.600582235
H	-5.403228029	3.336874518	-2.683855162
H	-4.558482279	-5.755941224	1.202903822
H	4.08492207	6.083233037	-0.991503395
C	6.65833202	1.404348688	-0.19782752
C	-7.051598669	-1.418529451	-1.018800844
H	2.429347629	-3.124638582	-0.04899675
H	-2.491688853	2.981897914	-2.035888355
C	5.268030589	1.284110031	-0.299421582
C	-5.673843633	-1.270004382	-0.843036867
C	-4.134019173	-5.16455773	0.377411462
C	3.833585135	5.134140969	-1.489123636
H	-4.897656989	-5.11652281	-0.409853556
H	4.770093548	4.72181476	-1.88526939
H	3.11266233	-4.681549462	0.448484674
H	-3.295713905	4.481234542	-2.521110754
H	-7.420938243	-2.240608052	-1.627651859
H	7.213789513	1.85629002	-1.015840119
H	4.854413981	-2.083637908	0.202686278
H	-4.845589115	1.764771303	-2.099250125
H	-1.271317687	-5.056013248	-0.385352447
H	1.305747368	4.696083421	-2.839774383
C	-4.722135795	-2.247358645	-1.479157495
C	4.556217625	1.802276065	-1.524642669
H	-3.274648048	-5.721630882	-0.008727519
H	3.182912375	5.374187328	-2.335812094
H	-5.249630056	-3.175962421	-1.730367467
H	5.181691772	2.551403797	-2.025827621
H	-0.372150107	-3.522374717	-0.273863787
H	0.175204125	3.522188047	-2.119601653
C	-0.911220402	-4.192287395	-0.955020431
C	0.911687543	3.677470039	-2.918459524
H	-4.347442744	-1.828133082	-2.424630823
H	4.42274026	0.976659245	-2.238667472
C	-2.042357235	-3.459651821	-1.703765059

C	2.005256348	2.590269044	-2.911915774
H	-0.803778835	-1.673738722	-2.061338719
H	0.562429687	0.997649077	-2.453667818
H	-0.181125572	-4.561246892	-1.686402546
H	0.369288083	3.613759434	-3.872935466
C	-1.39226458	-2.406767795	-2.62186459
C	1.306098328	1.251179073	-3.21744352
H	-3.283249483	-5.26831242	-2.066216335
H	3.609968159	3.787413294	-3.861628848
H	-3.543927173	-3.961268976	-3.238602761
H	3.726432973	2.032896711	-4.152286874
C	-2.792782032	-4.45857252	-2.61248475
C	3.032278477	2.871288486	-4.028344784
H	-0.713587788	-2.915714297	-3.316798832
H	0.784945423	1.338880897	-4.182861967
H	-2.12982781	-1.866791613	-3.225763288
H	2.017890735	0.431586173	-3.298901188
H	-2.064388547	-4.919268313	-3.296350425
H	2.495471185	2.989126683	-4.981855164
H	1.205340682	3.212840142	2.8880902
S	3.693501068	-1.649522777	-2.475551835
O	4.11373025	-0.642907539	-3.480785727
O	4.71829664	-2.612026722	-2.038980131
O	2.850627349	-1.069101369	-1.363541247
C	2.469102981	-2.702140695	-3.407898005
F	1.909961461	-3.617816469	-2.590419167
F	1.484269211	-1.954612162	-3.93753438
F	3.087150051	-3.350646629	-4.403733005
H	0.170862553	2.368720271	1.806787125

Supplementary Table 12 | Cartesian coordinate of VI. Units are presented in Å.

SCF energy (in toluene) = -4815.96314996 hartree

ZPE = 1.342531 hartree

Atom	X	Y	Z
Mo	-2.903339004	-0.10771704	0.028472292
Mo	2.24707775	0.551525093	0.30580415
P	2.820946551	-0.926364495	2.451287231
P	-3.931435105	2.229489112	0.485401451
N	-5.151093968	-0.486738959	-0.156823849
N	4.492580606	0.720469741	0.373626793
P	2.739585736	2.14645619	-1.750841771
P	-3.029814405	-2.639683167	-0.486027002
N	-0.923219673	0.163845251	0.1285249
N	0.237793023	0.306380102	0.177640502
N	-2.942706564	-0.709634757	3.135237501
N	-2.940914164	-0.497788872	2.019875988
N	-2.829508312	0.286120215	-1.975026147
N	-2.786378761	0.508096364	-3.084521039
N	0.864244731	2.732051679	1.67876216
N	2.172213107	2.006699995	1.460534638
H	2.071642036	-0.589753588	6.244128901
H	-4.082113865	4.750906934	3.458950284
H	-5.200987317	3.427806409	3.134012005
H	3.456795236	0.073810974	5.372234058
C	2.711636926	-0.731477057	5.360971088
C	-4.505878729	4.128348853	2.656945949
H	-3.313146045	1.803192942	3.462730517
H	1.866158729	1.515748738	3.930363884
H	-5.079487088	4.787242617	2.00140874
H	3.233828279	-1.681217085	5.488619805
H	-2.234695988	3.164977053	3.773032565
H	0.637266963	0.858855993	5.037915198
C	1.144081428	0.701773084	4.075107353
C	-2.640267507	2.518431415	2.979682907
H	0.051067483	-1.490206279	5.035976026
H	-1.866684673	4.936914666	2.261665303
C	1.827871739	-0.679257605	4.095411716
C	-3.356140044	3.394686185	1.932973358
H	-5.263144335	1.198852931	2.21350343

H	4.013485642	1.021360484	3.195256773
H	0.382537533	0.734099252	3.287090415
H	-1.820081817	1.938170364	2.540703122
H	5.037406284	-0.388533412	3.509458339
H	-6.312903936	2.29235148	1.30982528
C	0.696845631	-1.726762233	4.178441699
C	-2.341170245	4.430568671	1.407629837
H	2.900137928	-3.545381	4.381884008
H	-4.716392857	5.341737767	-0.040926424
H	0.066680603	-1.711725482	3.280078673
H	-1.551096548	3.964532018	0.801855502
C	4.380053576	0.077839579	2.766687913
C	-5.528631707	1.533887827	1.19978213
H	7.068445277	0.188141087	2.499126709
H	-8.115198711	0.84666251	0.789221993
H	1.067487115	-2.744954656	4.32141065
H	-2.80521908	5.207947975	0.79365671
H	4.547141204	-2.8830282	4.247383736
H	-6.192754065	4.35599984	0.051422666
H	-4.687307337	-2.336771762	1.969846168
H	3.635715124	3.623781374	0.702345925
C	3.755866905	-3.421912369	3.711342237
C	-5.322183182	4.599492875	-0.570253208
H	-2.657781717	-4.597056017	2.790886626
H	2.086233811	5.753598882	-0.607161621
C	6.561509329	0.44657807	1.573012088
C	-7.431625526	0.151133077	0.308505916
H	4.144524239	-4.428212975	3.498219436
H	-5.703302009	5.088253345	-1.478451003
C	5.167330709	0.410666146	1.518270422
C	-6.054613587	0.357410188	0.417951522
H	-4.950110143	-3.997158125	2.517105449
H	4.217354499	5.179870016	0.049955401
H	-2.131344368	-2.957836089	2.390489541
H	1.136259579	4.257252791	-0.467176568
C	-4.769760479	-3.372504144	1.630557778
C	4.055893474	4.116756295	-0.18132809
C	-2.350159609	-3.940532707	1.963714749
C	1.830349834	4.811083708	-1.111639027
H	-8.98053956	-1.11282956	-0.515752063
H	8.37344855	0.841214776	0.464215718
C	3.404785725	-2.758917025	2.364096171
C	-4.525015268	3.346482557	-0.985906246
H	-5.655529337	-3.457340978	0.990594788
H	5.041649571	3.680138145	-0.375657151
C	-7.911691541	-0.938269497	-0.414824707
C	7.286041193	0.815558421	0.441603073

H	-1.424091288	-4.34952609	1.549895991
H	1.273928054	5.06742516	-2.016022329
H	1.359887025	-3.583172562	2.254275063
H	-2.62442425	4.440530907	-1.256248817
H	5.542557047	-2.380843693	2.036699924
H	-6.403723968	2.268024956	-1.394246425
C	-3.492875585	-3.884452895	0.924635558
C	3.125264676	4.018625977	-1.411602998
C	2.283833002	-3.556298007	1.664615089
C	-3.293848092	3.777543737	-1.81414063
C	4.680845037	-2.800281188	1.5020894
C	-5.455673792	2.505330092	-1.889923721
H	4.919317871	-3.85021428	1.280516888
H	-5.695990457	3.096959648	-2.784819611
H	-4.166673843	-5.907222087	1.276367307
H	4.054798148	5.744147514	-2.330494376
C	6.593666733	1.157153713	-0.714488648
C	-6.989324136	-1.799429042	-1.003495157
H	2.05883359	-3.138599733	0.679439185
H	-2.714711778	2.919062397	-2.167210484
C	5.195720936	1.103800921	-0.729014779
C	-5.620118043	-1.559923332	-0.856386452
C	-3.792612274	-5.314252152	0.429047449
C	3.846621861	4.69748735	-2.596949134
H	-4.569664714	-5.335955175	-0.345539797
H	4.812426464	4.228883718	-2.819176102
H	2.615226105	-4.594407611	1.521037293
H	-3.635568152	4.331684225	-2.700188176
H	-7.319048037	-2.662620622	-1.576308978
H	7.124862197	1.453621282	-1.615099262
H	4.584458937	-2.287247229	0.544751177
H	-5.000123409	1.572322589	-2.228713407
H	-0.983685047	-5.045317981	-0.401569241
H	1.337203429	4.085357117	-3.899708329
C	-4.619602499	-2.499180332	-1.4802024
C	4.467773544	1.464108465	-2.006508804
H	-2.905987247	-5.827421219	0.044185278
H	3.252881568	4.706698031	-3.515872027
H	-5.089964868	-3.470994754	-1.673379599
H	5.090376494	2.144960668	-2.598111161
H	-0.173558042	-3.462575227	-0.315062923
H	0.141020888	3.245855002	-2.878862739
C	-0.67479192	-4.171910942	-0.984984045
C	0.851316027	3.123758182	-3.706918373
H	-4.307242719	-2.10038354	-2.456154269
H	4.347137886	0.563767569	-2.621623033
C	-1.84857665	-3.51799544	-1.741035842

C	1.84536286	1.969421697	-3.461807338
H	-0.70921505	-1.67431882	-2.134110858
H	0.25682637	0.722780756	-2.607711723
H	0.071955742	-4.51112488	-1.712050537
H	0.261724578	2.876877128	-4.600550551
C	-1.271096326	-2.439342254	-2.679110052
C	1.01143356	0.672816345	-3.400452251
H	-2.996857843	-5.39333511	-2.050102008
H	3.500844573	2.760988705	-4.699137694
H	-3.335100964	-4.130432358	-3.251551774
H	3.45181022	0.980098292	-4.588620269
C	-2.554468496	-4.573144291	-2.620852721
C	2.841298481	1.887114232	-4.637459544
H	-0.579228926	-2.921022342	-3.381844286
H	0.486281762	0.5514281	-4.359213306
H	-2.04662552	-1.946076408	-3.274809093
H	1.630456368	-0.210349039	-3.243470071
H	-1.808667062	-5.012093714	-3.29912004
H	2.270747157	1.841731439	-5.576477775
S	3.655941962	-1.87979256	-2.132702528
O	3.594794863	-1.243644992	-3.466320025
O	4.997194427	-2.051428525	-1.549142089
O	2.628101099	-1.313714547	-1.157252904
C	3.031038581	-3.606464368	-2.463426998
F	2.873680078	-4.295627164	-1.320565911
F	1.846670291	-3.559806047	-3.098992907
F	3.908877958	-4.248075437	-3.235464421
H	0.954722089	3.701248071	1.350623511
H	0.649667075	2.747300768	2.68449226
H	0.088180536	2.269330419	1.17820841

Supplementary Table 13 | Cartesian coordinate of VII. Units are presented in Å.

SCF energy (in toluene) = -4759.65039062 hartree

ZPE = 1.300929 hartree

Atom	X	Y	Z
Mo	-2.924811878	-0.084439953	0.000810928
Mo	2.227885423	0.737577683	0.48241388
P	2.761924421	-0.634176434	2.619828969
P	-3.636015502	2.314840674	0.519047444
N	-5.180332744	-0.222006742	-0.07852094
N	4.495025027	0.779917131	0.496635945
P	2.742721221	2.07986163	-1.636853994
P	-3.287483054	-2.552142603	-0.568057957
N	-0.923507871	0.069597987	0.091425142
N	0.222204759	0.19020411	0.154032495
N	-3.029128698	-0.74796598	3.099067966
N	-2.983549467	-0.505297437	1.989803131
N	-2.866579304	0.337906495	-1.991745443
N	-2.846388381	0.584841446	-3.099606364
N	1.965140971	2.103755598	1.409732184
H	1.996488316	-0.181225481	6.397189141
H	-3.272534669	4.787756241	3.510057476
H	-4.62578659	3.704680381	3.175894992
H	3.50949459	0.162364911	5.554483669
C	2.614131549	-0.472672637	5.534548986
C	-3.821565632	4.28156452	2.701749716
H	-2.973195759	1.796432195	3.467870397
H	2.172226808	1.914122917	4.212426034
H	-4.277923513	5.059540694	2.084743284
H	2.925234092	-1.506851344	5.693043483
H	-1.686766311	2.982681663	3.718332119
H	0.762333722	1.399682071	5.156915108
C	1.331862055	1.20973601	4.234496399
C	-2.218370445	2.399652887	2.951894345
H	-0.108154286	-0.856967451	5.117949355
H	-1.122627312	4.677153158	2.182363098
C	1.788269147	-0.263656102	4.246071367
C	-2.835037008	3.376189041	1.931886191
H	-5.006603807	1.474142484	2.306824413
H	4.074760664	1.243833743	3.320843728

H	0.696915086	1.439256943	3.376892112
H	-1.499099792	1.722209921	2.482961693
H	5.026059108	-0.209455429	3.667725823
H	-5.974375254	2.661248169	1.432933665
C	0.522846042	-1.145482248	4.264840562
C	-1.683158385	4.221894685	1.352961645
H	2.564252724	-3.26661789	4.514860401
H	-4.057672879	5.501383071	0.048784545
H	-0.0741467	-1.007149413	3.355412709
H	-0.974687959	3.607271799	0.783592721
C	4.387408504	0.270282989	2.916072282
C	-5.279004073	1.822654239	1.300068233
H	7.079592357	0.271646351	2.615218548
H	-7.940024186	1.380640675	1.052912364
H	0.747207863	-2.212358597	4.370557779
H	-2.035843081	5.038036558	0.713096358
H	4.260628381	-2.725197395	4.478419154
H	-5.637564219	4.703692234	0.235201422
H	-4.830422356	-2.241797771	1.951660371
H	3.870694811	3.549618889	0.698773165
C	3.463697885	-3.202388741	3.893696263
C	-4.78591226	4.848181076	-0.44251633
H	-2.885055235	-4.631190985	2.629586912
H	2.010920595	5.663201558	-0.512654838
C	6.569315769	0.491304415	1.680849202
C	-7.357504999	0.625989903	0.529429495
H	3.788731272	-4.233064403	3.688256388
H	-5.162897836	5.39039533	-1.322967482
C	5.172999192	0.513657372	1.649395921
C	-5.962763416	0.701429442	0.556064293
H	-5.167401871	-3.898050442	2.469551539
H	4.284471057	5.141581025	0.030062007
H	-2.305543941	-3.002032578	2.254318959
H	1.447855965	4.104819179	0.134614611
C	-4.984729204	-3.261074388	1.591254991
C	4.160511874	4.076328114	-0.215671154
C	-2.585661059	-3.960622169	1.809580258
C	1.792579423	4.615723712	-0.770993036
H	-9.069673639	-0.478413147	-0.19827732
H	8.375796448	0.713582842	0.51757116
C	3.227076955	-2.500903036	2.542150251
C	-4.171186377	3.514219989	-0.910471067
H	-5.896774243	-3.277781121	0.982849126
H	5.136983627	3.70593724	-0.550308771
C	-7.98452792	-0.406658688	-0.165169281
C	7.287767619	0.740532086	0.514101069
H	-1.696260871	-4.392605042	1.34103496

H	0.979848723	4.62106185	-1.504217648
H	1.124495404	-3.13170612	2.296312085
H	-2.170414718	4.37340489	-1.280075828
H	5.396519959	-2.249450274	2.293587425
H	-6.190940986	2.688762251	-1.225774459
C	-3.766289407	-3.820076136	0.822489597
C	3.077712999	3.953443536	-1.311025368
C	2.089406901	-3.21787307	1.78060502
C	-2.949198412	3.81300631	-1.806423622
C	4.53226095	-2.62706633	1.731539769
C	-5.243180786	2.800025654	-1.765551038
H	4.717368758	-3.689584171	1.521158029
H	-5.445280045	3.413744974	-2.655898695
H	-4.531533649	-5.812994957	1.163668147
H	3.837208995	5.740353127	-2.263237452
C	6.591209628	1.02701766	-0.654720185
C	-7.186121697	-1.344733501	-0.816417919
H	1.983531514	-2.823165276	0.765342694
H	-2.500413525	2.900336147	-2.205925216
C	5.191583273	1.049796554	-0.64305598
C	-5.794196048	-1.237145773	-0.757659582
C	-4.162715045	-5.22010995	0.313004506
C	3.580968733	4.710822573	-2.555302567
H	-4.970586558	-5.182859376	-0.429270917
H	4.487702312	4.259683738	-2.979115333
H	2.3307156	-4.288023269	1.699852469
H	-3.273918741	4.424871636	-2.661723723
H	-7.631513495	-2.16695495	-1.371722852
H	7.117090836	1.228867979	-1.584187152
H	4.484470673	-2.127760374	0.763700487
H	-4.926848568	1.813410279	-2.112004253
H	-1.390672634	-5.081209916	-0.655552398
H	0.951483493	3.85849289	-3.556042857
C	-4.926600371	-2.253370086	-1.457237389
C	4.449783741	1.363949823	-1.919939307
H	-3.320691282	-5.768696374	-0.120072635
H	2.828501212	4.77627528	-3.347366924
H	-5.500044804	-3.170733151	-1.64172575
H	5.077053868	1.995363185	-2.562154778
H	-0.492011311	-3.54494288	-0.623938275
H	0.001080689	2.724112733	-2.568566272
C	-1.080092777	-4.220294657	-1.257420156
C	0.660833188	2.808325196	-3.441257504
H	-4.63186816	-1.858851949	-2.440533612
H	4.289007189	0.420559051	-2.465035521
C	-2.267599583	-3.494483467	-1.920521488
C	1.864076981	1.849948863	-3.339291593

H	-1.077289102	-1.69978668	-2.361922621
H	0.651939393	0.208519288	-2.52393748
H	-0.404491175	-4.592134162	-2.037306373
H	0.066827813	2.534851053	-4.325306269
C	-1.688239961	-2.441534041	-2.885228735
C	1.316201726	0.407901037	-3.37116729
H	-3.513482187	-5.313677908	-2.182379247
H	3.243730463	3.031418683	-4.613009014
H	-3.904414675	-3.999177534	-3.308617038
H	3.608286896	1.289101531	-4.54763319
C	-3.088825699	-4.492388592	-2.764863119
C	2.801890145	2.031631479	-4.550737043
H	-1.04337665	-2.949376121	-3.614764785
H	0.733680598	0.279234341	-4.296075895
H	-2.466870491	-1.913945001	-3.446461951
H	2.114314837	-0.337269937	-3.374357293
H	-2.42440106	-4.936460703	-3.520792284
H	2.221248295	1.87116782	-5.471547991
S	3.793500353	-2.240781724	-2.017687932
O	4.116441824	-1.511520418	-3.271243919
O	4.892320877	-3.012923286	-1.409953484
O	2.979180375	-1.425719898	-1.049172953
C	2.579455875	-3.540548652	-2.572245301
F	2.169762707	-4.286047399	-1.525354346
F	1.489073823	-2.975675962	-3.130350565
F	3.138790646	-4.357117208	-3.475358232

Supplementary Table 14 | Cartesian coordinate of VIII. Units are presented in Å.

SCF energy (in toluene) = -2036.04242654 hartree

ZPE = 0.646560 hartree

Atom	X	Y	Z
Mo	2.659031782	0.003847832	0.008369209
P	3.167843185	-2.187772084	1.173564489
N	4.906853844	-0.007006176	-0.01577229
P	3.163919188	2.190493396	-1.168030075
N	-0.508143769	0.018982042	0.042746851
N	0.627561218	0.013603454	0.030405016
N	2.662630055	1.459652118	2.825545605
N	2.660579558	0.929070231	1.820843475
N	2.612468294	-0.921232062	-1.803679598
N	2.587683343	-1.451571597	-2.808187894
H	2.567874951	-3.759688747	4.692952513
H	3.96971534	-2.823388195	4.17194231
C	3.171438333	-3.485674866	3.814250437
H	2.425844126	-0.839078522	3.819871772
H	3.632313823	-4.402358742	3.438180152
H	1.098194837	-1.870639728	4.362576217
C	1.660067706	-1.541287762	3.475510079
H	0.456011328	-3.865804503	3.299453632
C	2.259907168	-2.782856672	2.785543271
H	4.583045111	-0.998852873	2.724041302
H	0.970996805	-0.999607972	2.819495308
H	5.471467097	-2.439279022	2.222512248
C	1.08130661	-3.704834949	2.409012857
H	3.353441261	-5.407803699	1.596245137
H	0.444785363	-3.254971913	1.636277115
C	4.840090478	-1.620427243	1.853681776
H	7.529906315	-1.428800512	1.585164276
H	1.404826626	-4.690934288	2.059916852
H	4.985541238	-4.703605064	1.6078241
H	4.586895642	2.510699728	1.431443644
C	4.133138029	-4.965394645	0.967925165
H	2.77280051	5.040841415	1.365740974
C	7.008650439	-0.800835529	0.866373158
H	4.47570422	-5.751867209	0.278370178
C	5.611279997	-0.780771798	0.86354941

H	5.018219022	4.224266167	1.477764825
H	2.082092821	3.41146625	1.347492285
C	4.819410149	3.375694509	0.80638095
C	2.457890629	4.215348003	0.709481568
H	8.804228586	-0.02636472	-0.05794856
C	3.630337868	-3.765645268	0.139930314
H	5.748505437	3.168450972	0.262075075
C	7.716190911	-0.020951537	-0.046160799
H	1.626245933	4.58214344	0.100421341
H	1.580611626	-4.564628207	-0.061448199
H	5.711716058	-3.190246672	-0.311400994
C	3.663566063	3.76380265	-0.144816697
C	2.402374745	-4.205557804	-0.688265065
C	4.769282685	-3.388475719	-0.835765944
H	4.94556586	-4.238815635	-1.511195284
H	4.524550091	5.741953761	-0.301597692
C	6.996851554	0.766030252	-0.943252353
H	2.02051792	-3.398061208	-1.318039147
C	5.59974615	0.759828074	-0.910165642
C	4.160025735	4.958740343	-0.983552035
H	4.996154122	4.688899839	-1.641330826
H	2.695264658	-5.033861188	-1.351155844
H	7.508641891	1.388893739	-1.673197316
H	4.531822864	-2.52118115	-1.455774679
H	1.406482908	4.710570111	-2.017101473
C	4.815666429	1.607112843	-1.883596421
H	3.371387409	5.408464648	-1.595298852
H	5.446844387	2.420022884	-2.265691935
H	0.442138222	3.283842693	-1.572593809
C	1.066194707	3.727574549	-2.358924065
H	4.534452264	0.988187275	-2.748370088
C	2.227609878	2.794386132	-2.760372986
H	0.921261607	1.023549904	-2.766721664
H	0.423508514	3.894451565	-3.23579184
C	1.601371812	1.558706111	-3.437306025
H	3.60096828	4.400936905	-3.442397685
H	3.908025158	2.818816169	-4.182677875
C	3.123712345	3.488556612	-3.808356499
H	1.023924249	1.893501421	-4.312250871
H	2.352915134	0.849258126	-3.797825383
H	2.504253275	3.768088938	-4.674161249

Supplementary Table 15 | Cartesian coordinate of IX. Units are presented in Å.

SCF energy (in toluene) = -2779.48508967 hartree

ZPE = 0.679697 hartree

Atom	X	Y	Z
Mo	2.543823026	0.411087344	0.304962972
P	3.112064197	-0.816271482	2.452595458
N	4.819973895	0.741782854	0.396727368
P	2.885497538	2.084537431	-1.490692965
N	-0.409376143	1.153250176	0.99823078
N	0.858593696	0.852979878	0.679488653
H	2.343054054	0.076753165	6.155909035
H	3.913666948	-0.029009395	5.354473619
C	2.894183031	-0.436660801	5.353689838
H	2.927620783	1.886310352	3.919072874
H	2.950001762	-1.492440103	5.624122728
H	1.367080979	1.694024606	4.726312815
C	1.97522642	1.340154574	3.879986472
H	0.187288856	-0.442514985	4.872072221
C	2.166797956	-0.187433479	4.014982109
H	4.629260146	0.865316976	3.292881454
H	1.468407628	1.606777502	2.949194325
H	5.459758528	-0.684020981	3.369123104
C	0.772680906	-0.847426253	4.032711912
H	2.572452585	-3.270557708	4.533092202
H	0.222080356	-0.637962716	3.107777129
C	4.817633669	-0.062392411	2.733689199
H	7.494804204	-0.04554184	2.309701134
H	0.828440887	-1.934009354	4.167227704
H	4.310288375	-2.881166511	4.561676825
H	3.862419376	3.255554398	1.033470441
C	3.508540393	-3.336892515	3.966602987
H	1.669996928	5.26699428	0.270369266
C	6.945498746	0.313187232	1.442761896
H	3.748283746	-4.4062851	3.865468769
C	5.549070721	0.315306381	1.4681052
H	4.013786769	4.99458937	0.69436015
H	1.377496274	3.547636677	0.617332491
C	4.035856241	3.992599945	0.240153112
C	1.603727927	4.265671126	-0.181114125

H	8.703009468	0.786173175	0.277896343
C	3.401719698	-2.728443914	2.556973606
H	5.045604622	3.836929945	-0.158768687
C	7.615514285	0.783279312	0.315722283
H	0.765168737	4.276526066	-0.884502704
H	1.257327829	-3.161845281	2.254277927
H	5.596599117	-2.704582012	2.374848479
C	2.948151823	3.915456668	-0.85404903
C	2.229752542	-3.39037626	1.798938719
C	4.715323256	-3.034915907	1.808845588
H	4.80397298	-4.124268532	1.684858212
H	3.411565684	5.946750689	-1.452572653
C	6.866730962	1.259349459	-0.757776777
H	2.20496844	-3.072580281	0.751920475
C	5.470964051	1.224352686	-0.699991838
C	3.286271656	4.963368328	-1.931147348
H	4.226150364	4.734622392	-2.449946811
H	2.356694744	-4.483315778	1.820526879
H	7.352011711	1.639035029	-1.653452535
H	4.753306378	-2.601949324	0.806683828
H	0.78074871	3.895661883	-3.031496793
C	4.667566875	1.690899871	-1.894941765
H	2.49844638	5.069480679	-2.684063331
H	5.192863849	2.515835564	-2.39438956
H	0.039422986	2.457206206	-2.291209632
C	0.657408883	2.811462562	-3.127333991
H	4.625101661	0.847711487	-2.601821592
C	1.99549	2.049175729	-3.207690656
H	1.059405513	0.129618939	-2.698400073
H	0.090903237	2.629752929	-4.052480022
C	1.669007799	0.569883302	-3.496374939
H	3.102648301	3.656824032	-4.257597772
H	3.774305361	2.029256407	-4.498770006
C	2.845890335	2.598572549	-4.371294774
H	1.100478806	0.510302776	-4.437399403
H	2.56772968	-0.042523699	-3.606283541
H	2.276699	2.498206778	-5.307878983
H	-0.56781326	2.148544079	1.167909318
S	4.056777356	-2.087775663	-1.969034381
O	4.402978764	-1.19535193	-3.100475362
O	5.141784748	-2.931527722	-1.442851517
O	3.218086967	-1.436336993	-0.885228582
C	2.810545059	-3.2791059	-2.677410508
F	2.377470098	-4.124432748	-1.726619731
F	1.748059144	-2.619723417	-3.171282095
F	3.366299123	-3.99342107	-3.665039201
H	-1.092194081	0.762945582	0.346312067

Supplementary Table 16 | Cartesian coordinate of X. Units are presented in Å.

SCF energy (in toluene) = -2779.89642593 hartree

ZPE = 0.693512 hartree

Atom	X	Y	Z
Mo	-0.087599173	-0.532788822	-0.704128949
P	2.185305993	-1.37841144	-0.079536441
N	-0.212800431	-0.690744436	1.694965242
P	-2.575492022	-0.425750657	-0.120873092
N	0.41610222	1.048227851	-3.11315416
N	0.12803547	-0.273856939	-2.524925169
H	3.23714817	-5.002836989	-0.987186835
H	2.934698861	-4.340184699	0.620785511
C	3.269186298	-4.079460144	-0.391209116
H	0.486742976	-3.795512315	-0.17522473
H	4.315363847	-3.766084013	-0.333646613
H	0.935991261	-4.525545502	-1.718664205
C	0.928596813	-3.57988771	-1.156830927
H	2.831757967	-3.657149128	-3.079164975
C	2.369095437	-3.021397641	-1.059969462
H	1.537213741	-2.988266245	1.618246485
H	0.255683377	-2.904936168	-1.716225748
H	2.832837849	-1.987879916	2.248013442
C	2.872370285	-2.731237579	-2.488299229
H	5.24994675	-1.823398868	-0.844594144
H	2.245035704	-1.986909203	-2.994250684
C	1.898049467	-1.951785514	1.67805799
H	1.827366916	-1.296750375	4.322530523
H	3.912069554	-2.385846525	-2.498864605
H	5.074803495	-1.868900772	0.925768175
H	-1.57290243	-3.00218433	1.039706046
C	5.07945256	-1.210852156	0.047351248
H	-3.719344465	-3.998279066	-0.852242877
C	0.952318118	-0.939752511	3.785605509
H	5.944606124	-0.540746909	0.151935341
C	0.856213158	-1.136122269	2.408662796
H	-3.068227132	-3.856733483	1.453762656
H	-2.307741478	-3.096658699	-1.430115235
C	-2.626323347	-2.86109404	1.308882428
C	-3.341737303	-2.990491545	-1.075395294

H	-0.033288349	-0.133082405	5.531393691
C	3.802902063	-0.354696742	-0.047128176
H	-2.66659997	-2.34294592	2.273625839
C	-0.0890953	-0.301246012	4.458127557
H	-3.940286552	-2.592012613	-1.898809468
H	3.82151421	-0.092061469	-2.242586466
H	3.871158485	0.031000134	2.121289663
C	-3.420207052	-2.128870874	0.204029919
C	3.832845084	0.510150369	-1.325985177
C	3.740107316	0.579708135	1.180309678
H	4.569621492	1.297794697	1.11314085
H	-5.258096028	-3.012207646	0.927821513
C	-1.206919385	0.102405025	3.735697931
H	2.986675031	1.20636498	-1.339657553
C	-1.243898068	-0.099895752	2.352243343
C	-4.882772108	-2.009796437	0.677131408
H	-4.979047201	-1.395411494	1.581840074
H	4.755146466	1.107429493	-1.335594259
H	-2.044274016	0.590041735	4.228215721
H	2.809566809	1.152696968	1.241571024
H	-5.150431779	-0.771346913	-1.963926222
C	-2.450100242	0.368460287	1.565324044
H	-5.548145378	-1.599853709	-0.088800932
H	-3.359436606	0.235204067	2.165943607
H	-3.672645041	-0.524933088	-2.923950754
C	-4.415324139	-0.027840407	-2.286625655
H	-2.348096213	1.44832154	1.382449953
C	-3.760030855	0.728627407	-1.112948186
H	-2.218552547	1.472790532	-2.465844682
H	-4.949493701	0.701334009	-2.911639584
C	-2.913355184	1.868747558	-1.714400543
H	-5.469765526	0.602665721	0.291598348
H	-4.423268817	2.016659088	0.546509506
C	-4.849134403	1.350036999	-0.211950666
H	-3.586475472	2.574363359	-2.22237102
H	-2.345277417	2.425412077	-0.964110123
H	-5.514996201	1.960233727	-0.83852727
H	-0.304600157	1.289002059	-3.806076748
S	0.245349562	2.732539455	0.557591478
O	-1.10938386	3.296970139	0.676473625
O	0.947335655	2.350258591	1.788379745
O	0.347574249	1.649118777	-0.54355041
C	1.28018391	4.04161934	-0.281652863
F	2.560027031	3.646090344	-0.335807584
F	0.837094236	4.218862134	-1.544552192
F	1.193104104	5.188141	0.380001553
H	0.435641675	1.766558704	-2.34933828

```
  H     1.324037558    1.027603791   -3.595498159
---------------------------------------------------
```

Supplementary Table 17 | Cartesian coordinate of XI. Units are presented in Å.

SCF energy (in toluene) = -2723.59879416 hartree

ZPE = 0.652246 hartree

Atom	X	Y	Z
Mo	2.583513278	0.730782997	0.409409573
P	2.995062843	-0.769789456	2.416464735
N	4.757091388	1.126657939	0.815022187
P	3.244896452	1.976654377	-1.679615958
N	1.879410911	1.923562635	1.330637526
H	1.762388891	-1.619454027	5.980769889
H	3.361986914	-1.055123373	5.481143387
C	2.476762123	-1.610966812	5.144165766
H	2.088601597	1.046588563	4.815384926
H	2.771805811	-2.649512006	4.959980453
H	0.490260479	0.334297345	5.044844567
C	1.310307485	0.450557362	4.321788555
H	-0.116999127	-1.845221245	4.355484762
C	1.802813318	-0.958598675	3.920513181
H	3.64591389	1.279415371	3.452814163
H	0.930210823	1.015158552	3.463468692
H	4.820267747	0.071392837	4.004476248
C	0.563370005	-1.773797338	3.493667564
H	4.902285143	-2.563573034	4.115334066
H	0.019507253	-1.29046129	2.675640766
C	4.272764212	0.438989547	3.128152062
H	6.884999939	1.088090122	3.450642577
H	0.80409459	-2.792233343	3.178113665
H	5.937856171	-1.740625382	2.927227283
H	3.442912966	3.853925235	0.576391667
C	5.200580173	-2.542380245	3.06092807
H	1.297572487	5.214904784	-1.220843538
C	6.550870125	1.245534546	2.428236408
H	5.712848759	-3.491964812	2.845395877
C	5.227972755	0.944667155	2.089368545
H	3.50558259	5.451326728	-0.194979208
H	1.100474642	3.632067012	-0.432898949
C	3.737524951	4.385376505	-0.334878186
C	1.456907929	4.1327868	-1.339607812
H	8.44799931	1.992736359	1.712159273

C	3.997308672	-2.39376266	2.108750592
H	4.824934241	4.303419404	-0.461262911
C	7.417318648	1.749107017	1.462441393
H	0.841466908	3.798690014	-2.18266913
H	2.772116456	-3.845316132	3.235876429
H	5.178783886	-1.464357829	0.486204582
C	2.961933017	3.872905698	-1.568903
C	3.10008858	-3.64483715	2.210216815
C	4.514593457	-2.320981908	0.652814615
H	5.089638867	-3.231963834	0.428337483
H	3.359102042	5.727825947	-2.604871093
C	6.936210587	1.933785939	0.167419716
H	2.221264661	-3.566535746	1.562336194
C	5.612409232	1.609609717	-0.136415712
C	3.440857553	4.650105438	-2.809005804
H	4.490351469	4.444558683	-3.058567611
H	3.685800356	-4.516906212	1.88196965
H	7.580528423	2.324616963	-0.616406577
H	3.68012728	-2.275717026	-0.058999727
H	1.271737439	2.639945432	-3.98580946
C	5.116290643	1.759780511	-1.552367055
H	2.826791799	4.440961746	-3.691775672
H	5.678560232	2.549590504	-2.06684166
H	0.760379795	1.193670361	-3.07493594
C	1.458832188	1.570329103	-3.83173764
H	5.332855787	0.827364578	-2.09274885
C	2.924347705	1.283406873	-3.444956342
H	2.40096366	-0.700719998	-2.642543027
H	1.230640464	1.060264005	-4.779247946
C	3.102331257	-0.250811702	-3.350358867
H	3.757109609	2.877811328	-4.736355205
H	4.93229976	1.622816835	-4.298403971
C	3.877218777	1.809410168	-4.538297683
H	2.906373231	-0.687833962	-4.340550882
H	4.124880091	-0.543687207	-3.073754975
H	3.66433001	1.277011774	-5.477286327
S	0.089146906	-1.419983755	-0.335890063
O	0.797085184	-2.654910767	0.055771813
O	-1.021706026	-0.957066605	0.506478993
O	1.044678771	-0.29867308	-0.773504031
C	-0.667275678	-1.796842083	-1.997369384
F	-1.227645381	-0.695393523	-2.521999249
F	0.272695625	-2.243010908	-2.850573339
F	-1.608103164	-2.739623976	-1.867002721

Supplementary Table 18 | Cartesian coordinate of XII. Units are presented in Å.

```
SCF energy (in toluene) = -2724.16007909 hartree

ZPE =  0.659619 hartree

---------------------------------------------------

 Atom            Coordinates (Angstroms)

             X               Y                Z

---------------------------------------------------

 Mo     -0.46557288     0.04962626    -0.09442448
  P    -1.424416763   -2.217968855    0.558353543
  N    -1.919728212    -0.23348405    -1.69912721
  P     -0.82598655    2.397945233   -0.859908169
  N     0.943409506   -0.290738103   -1.130450282
  H    -0.491633022   -5.855901291    -0.34949788
  H    -2.099859598   -5.125149911   -0.275420628
  C    -1.085209757   -5.070401584    0.141201108
  H    -1.245013406   -3.612871679   -2.165986335
  H    -1.132461204   -5.318251039    1.206164821
  H     0.369051989   -4.330838256   -2.038456797
  C    -0.280325784   -3.533180563   -1.648939084
  H     1.592839111    -4.49852419    0.077067477
  C    -0.413307798   -3.709117676   -0.120974804
  H    -3.317462342   -3.155084003   -0.814102163
  H     0.173894921   -2.571900504   -1.907726372
  H    -3.722882116   -1.702866817    0.093196441
  C     0.994192337   -3.681879428    0.508806563
  H    -3.127304329   -4.607046965    1.781818043
  H     1.503488786   -2.733466562    0.309221165
  C    -2.946071656   -2.160005561   -0.538726332
  H    -4.292092461   -2.375020553   -2.847285128
  H     0.970340865   -3.817823682      1.5944459
  H    -4.245497191   -3.249490307    1.539333307
  H     1.981340696    1.960275954   -1.448158463
  C    -3.424067161   -3.624892546     2.16200653
  H     2.055368391     4.52717406    0.423260928
  C    -3.630403401   -1.511555484   -2.855943652
  H    -3.834143976   -3.776624177    3.171474023
  C    -2.803053432   -1.291567862   -1.758392243
  H     2.686801079    3.578492705   -1.655255398
  H     1.502731049    2.915882361    0.925533548
  C     1.771441027    2.983340678   -1.783188427
  C     1.183334829    3.858344958    0.473199161
  H    -4.270492728   -0.786053971   -4.798277729
```

C	-2.244144976	-2.633269065	2.260949157
H	1.547295702	2.968628276	-2.85784695
C	-3.628892539	-0.624923818	-3.935399896
H	0.452285782	4.323146764	1.14140053
H	-0.885417011	-4.203987711	2.99121723
H	-3.500710461	-0.816714822	2.121707503
C	0.643065257	3.640133658	-0.956606446
C	-1.200580574	-3.186580424	3.252476935
C	-2.783069024	-1.290529028	2.807505551
H	-3.316351655	-1.47865398	3.75091456
H	1.19846975	5.594423788	-1.701521561
C	-2.796396217	0.49155189	-3.850787918
H	-0.310037421	-2.555148615	3.321367421
C	-1.965992275	0.67218012	-2.746942706
C	0.28412816	4.989959476	-1.606983133
H	-0.131178285	4.868497604	-2.616440001
H	-1.656132706	-3.23382755	4.252484781
H	-2.779759536	1.233999453	-4.64592042
H	-1.979191933	-0.579976765	3.024664668
H	-1.584807648	5.012589486	0.755133866
C	-1.098442751	1.901535706	-2.652437405
H	-0.425898986	5.572162492	-1.009443227
H	-0.088571143	1.668879284	-3.011177282
H	-1.716290879	3.536945107	1.740066488
C	-2.198511546	4.126795484	0.950365713
H	-1.492885395	2.698920094	-3.294993983
C	-2.451316513	3.285476615	-0.317427489
H	-3.125040062	1.563480931	0.880674237
H	-3.164245924	4.479581018	1.34127845
C	-3.478616395	2.190890781	0.052308302
H	-2.391868875	4.948196698	-1.776874874
H	-3.371882493	3.560167716	-2.294376012
C	-3.066149528	4.160252126	-1.428092353
H	-4.41086033	2.676722146	0.377521145
H	-3.719682628	1.539228986	-0.794522129
H	-3.971522721	4.649300676	-1.038466125
S	0.911540264	0.082587539	3.048663729
O	0.126201658	0.19068768	4.288567084
O	1.494379867	-1.226983716	2.704960317
O	0.229262059	0.783134816	1.863308173
C	2.374226692	1.214759083	3.284111025
F	3.108556601	1.270554337	2.159482998
F	1.965146273	2.458210342	3.585032082
F	3.145807213	0.761752996	4.280009104
H	1.081220374	-0.500193908	-2.13643322

Supplementary Table 19 | Cartesian coordinate of XIII. Units are presented in Å.

SCF energy (in toluene) = -2833.66915087 hartree

ZPE = 0.670964 hartree

Atom	X	Y	Z
Mo	-0.331746965	0.588211857	0.384911767
P	0.345354832	-0.881270949	2.456881255
N	1.854007513	1.031342458	0.698688477
P	0.261712061	2.064082234	-1.653581534
N	-0.76999767	1.965947074	1.505005982
H	-1.143370689	-1.778287848	5.932414017
H	0.457495088	-1.109141695	5.587022174
C	-0.347335096	-1.731565546	5.174401428
H	-0.854948492	0.911353767	4.692297669
H	0.033347232	-2.749037652	5.042646459
H	-2.380882291	0.050995029	4.944375337
C	-1.567498189	0.220922682	4.224117409
H	-2.832499264	-2.159975587	4.102043581
C	-0.933395111	-1.142276298	3.876809772
H	0.766968332	1.334244168	3.295971871
H	-1.993467064	0.710383486	3.343873427
H	2.032859142	0.270637277	3.966966041
C	-2.050384032	-2.06180142	3.334270405
H	2.092822739	-2.399867048	4.551324778
H	-2.508067116	-1.651458932	2.428239782
C	1.458397299	0.514943471	3.066921457
H	4.084501115	1.247610592	3.231198479
H	-1.69237547	-3.065494201	3.090152047
H	3.256910049	-1.522581831	3.535054212
H	0.428987968	3.656022738	0.87157071
C	2.574755628	-2.380854705	3.569786047
H	-1.004179601	5.610860861	-1.096087525
C	3.705945614	1.303859399	2.213608276
H	3.197449521	-3.284438145	3.494087018
C	2.373148843	0.967890567	1.959706497
H	0.89947379	5.28388236	0.310767679
H	-1.688883827	4.042463111	-0.626746671
C	0.972098093	4.206910036	0.096302696
C	-1.038353776	4.542735816	-1.355222364
H	5.572217234	1.961849483	1.343550131

C	1.580583684	-2.381847868	2.385966631
H	2.033810608	3.937631093	0.151328153
C	4.53108108	1.701952274	1.163138903
H	-1.502102199	4.459931047	-2.345139201
H	0.334108134	-3.939810751	3.32658823
H	3.045991798	-1.384499731	1.068920107
C	0.391240376	3.963993676	-1.31495395
C	0.81376978	-3.721129406	2.365747015
C	2.421541801	-2.284280116	1.091095619
H	3.099112351	-3.150355016	1.062488865
H	1.417494301	5.752631406	-1.951978653
C	3.99768979	1.759114197	-0.123610051
H	0.055223425	-3.753976391	1.578190311
C	2.656886068	1.424945357	-0.328871704
C	1.306890487	4.717083799	-2.305502889
H	2.315805626	4.285478988	-2.341460884
H	1.533249974	-4.529604214	2.171142381
H	4.609048703	2.06135365	-0.970253036
H	1.817492333	-2.317211998	0.182911834
H	-2.444472305	2.406385157	-2.84268436
C	2.047065113	1.466139486	-1.705973288
H	0.915828309	4.762523268	-3.322779105
H	2.68545571	2.029433669	-2.397451236
H	-2.171980764	0.664169439	-2.643608967
C	-1.94706667	1.528375142	-3.273789846
H	1.979003604	0.436459626	-2.081692135
C	-0.424083861	1.73345654	-3.433525894
H	0.156749389	-0.38855284	-3.247799144
H	-2.390849021	1.349233081	-4.264143371
C	0.204922946	0.429778388	-3.969236547
H	-0.683899838	3.771283602	-4.258253116
H	0.892023511	3.031205865	-4.639757598
C	-0.174872842	2.828914769	-4.487797192
H	-0.349686907	0.116475651	-4.86497326
H	1.250525205	0.579085534	-4.269633305
H	-0.577074906	2.472896828	-5.447869158
S	-0.622915474	-2.626982622	-1.090052089
O	0.398494557	-3.631796928	-1.42115567
O	-1.498432953	-2.87989411	0.071027543
O	-0.065006998	-1.204196454	-1.12091806
C	-1.794467413	-2.653843641	-2.543775011
F	-2.732674872	-1.69559267	-2.423550674
F	-1.134329095	-2.456398797	-3.698323705
F	-2.405804542	-3.8420822	-2.600015272
N	-2.330456562	0.207038688	0.133964308
N	-3.44526954	0.054798279	0.046878968
H	-1.744633999	2.250447486	1.706150778

Supplementary Table 20 | Cartesian coordinate of XIV. Units are presented in Å.

SCF energy (in toluene) = -2834.28035937 hartree

ZPE = 0.685336 hartree

Atom	X	Y	Z
Mo	-0.340549375	0.6615316	0.466625953
P	0.353693507	-0.951939655	2.440003058
N	1.823997944	1.088307471	0.793945033
P	0.27926516	2.09215135	-1.659110849
H	-0.99841343	-1.92212065	5.944552009
H	0.5565577	-1.179556143	5.540169397
C	-0.233188045	-1.840441544	5.158161328
H	-0.927865949	0.790842413	4.636923051
H	0.191036364	-2.840194124	5.016482096
H	-2.360572177	-0.179793357	4.995583478
C	-1.604507571	0.031384684	4.22544186
H	-2.733946315	-2.386828394	4.161285395
C	-0.890570624	-1.292791213	3.875987046
H	0.804961995	1.143208991	3.54067388
H	-2.122305615	0.453801417	3.359133077
H	2.110070593	0.03573333	3.969382801
C	-1.972560427	-2.274291269	3.374738855
H	2.219648354	-2.505714344	4.377392885
H	-2.463064152	-1.908575824	2.467708023
C	1.48485934	0.384465926	3.140850919
H	4.025168836	1.295077656	3.366367667
H	-1.574152026	-3.266631321	3.15132186
H	3.344132719	-1.626533777	3.316656884
H	-0.136080601	4.14435076	0.626037113
C	2.651335681	-2.474758354	3.372529757
H	-1.098679561	5.657259688	-1.614132842
C	3.654281393	1.397257661	2.348931014
H	3.254247857	-3.386042523	3.247967091
C	2.350553862	0.985715069	2.057154604
H	0.757691331	5.531219176	-0.027994835
H	-1.846066693	4.098629158	-1.214061284
C	0.659145798	4.436035767	-0.062206633
C	-1.077420803	4.579444513	-1.833406688
H	5.475672323	2.258849021	1.558565331
C	1.595856179	-2.442681228	2.243567447

H	1.604876012	4.00790721	0.290570847
C	4.461082748	1.929642262	1.345434009
H	-1.360150957	4.45548707	-2.883608541
H	0.454036959	-4.064594689	3.20284211
H	2.943678932	-1.343865254	0.889169586
C	0.326162654	4.023805054	-1.511381921
C	0.854826216	-3.79545939	2.218235961
C	2.361050346	-2.271392925	0.911164774
H	3.06992572	-3.10744946	0.815982517
H	1.351827207	5.769782298	-2.267346722
C	3.937056277	2.009964143	0.054125868
H	0.04089194	-3.81249197	1.488395136
C	2.635386168	1.57642196	-0.196725943
C	1.398152263	4.679845121	-2.409233454
H	2.410124504	4.364940159	-2.124214015
H	1.575776626	-4.577215706	1.93791377
H	4.537593271	2.391358609	-0.76843182
H	1.707178738	-2.291130739	0.03782556
H	-2.322487306	2.375018828	-3.16129129
C	2.080399264	1.567931243	-1.595239232
H	1.265816076	4.484872962	-3.473841745
H	2.730366198	2.123743068	-2.281628189
H	-2.111889647	0.689443502	-2.660979017
C	-1.810807234	1.434625461	-3.400782958
H	2.053353175	0.523505906	-1.934134355
C	-0.277772623	1.608417043	-3.457126497
H	0.290818247	-0.494182193	-3.061538158
H	-2.173489662	1.101438677	-4.384056728
C	0.388220013	0.266809673	-3.837341874
H	-0.408866034	3.579054727	-4.470265781
H	1.15512793	2.752594289	-4.684138774
C	0.073321525	2.602984457	-4.581587366
H	-0.096907463	-0.11662085	-4.745747823
H	1.452092565	0.402591429	-4.071966225
H	-0.282170521	2.175777123	-5.531079317
S	-0.854228345	-2.469012776	-1.009840774
O	0.03340331	-3.500829115	-1.564363723
O	-1.586116738	-2.769138646	0.235132812
O	-0.16626932	-1.100927839	-0.989759217
C	-2.215699416	-2.29885252	-2.279507468
F	-3.096005617	-1.348916646	-1.921095921
F	-1.717866965	-1.981490915	-3.488346856
F	-2.864432973	-3.464741087	-2.380235393
N	-2.329628019	0.477699137	0.424557686
N	-3.462547801	0.490416934	0.517442512
N	-0.625025822	2.120119614	1.721326508
H	-1.544679986	2.38044182	2.091184682

H	0.0864368	2.738186956	2.124352349

Supplementary Table 21 | Cartesian coordinate of XV. Units are presented in Å.

SCF energy (in toluene) = -2834.87044070 hartree

ZPE = 0.697281 hartree

Atom	X	Y	Z
Mo	-0.291080171	0.477582874	0.32830479
P	0.322995639	-0.91768583	2.400825447
N	1.845790627	1.000813457	0.672304147
P	0.285221807	2.05195651	-1.620786606
H	-1.14032503	-1.728337351	5.925175832
H	0.414353557	-0.966284835	5.553244889
C	-0.340649127	-1.667067758	5.171263512
H	-1.032956102	0.922957286	4.438339417
H	0.114805515	-2.659790408	5.102894052
H	-2.450851453	-0.039104995	4.856775937
C	-1.69266364	0.121172654	4.07656675
H	-2.823246488	-2.266714655	4.058300731
C	-0.944198856	-1.206800791	3.831369249
H	0.762353333	1.309518606	3.313927423
H	-2.220335253	0.453701821	3.176747961
H	1.998076205	0.232115675	3.942761348
C	-1.991496275	-2.231641497	3.338198227
H	2.124475485	-2.307275083	4.547723138
H	-2.393866986	-1.960984247	2.356621018
C	1.43481728	0.486765922	3.038294257
H	4.063057732	1.188303756	3.233013385
H	-1.582173729	-3.241512008	3.25623328
H	3.285355082	-1.475902032	3.48855007
H	0.862879946	3.593690546	0.820211459
C	2.604765927	-2.332454021	3.565016739
H	-0.853926613	5.665098806	-1.03692169
C	3.699100901	1.252627369	2.209980613
H	3.230511075	-3.236576512	3.531358927
C	2.366759911	0.933843919	1.94138395
H	1.094000056	5.273093895	0.28079523
H	-1.572946319	4.112419916	-0.557375552
C	1.215585649	4.217397747	-0.004437644
C	-0.923483698	4.598238187	-1.297923748
H	5.590001093	1.880859538	1.362446777
C	1.6088021	-2.392778072	2.384042775

H	2.290508743	4.025993059	-0.096055381
C	4.546932272	1.638494199	1.1717846
H	-1.438013386	4.529437228	-2.263536069
H	0.43418171	-3.965925349	3.388530981
H	3.031742759	-1.393799565	1.021887532
C	0.486878521	3.973411031	-1.350194197
C	0.883774996	-3.754870133	2.41106456
C	2.446296996	-2.31678599	1.086276037
H	3.159267567	-3.154886293	1.095135937
H	1.560439136	5.730750751	-2.022573279
C	4.01978838	1.70315137	-0.12031707
H	0.110581623	-3.827475155	1.640495067
C	2.678850945	1.392563663	-0.343031677
C	1.332983994	4.723868367	-2.405035917
H	2.294673934	4.229300084	-2.592045657
H	1.624726016	-4.545141463	2.220290114
H	4.64368045	1.991800384	-0.963208748
H	1.846806468	-2.410393339	0.179746885
H	-2.434616404	2.42995065	-2.776632799
C	2.073005514	1.464543677	-1.71737449
H	0.823233336	4.851492445	-3.36040198
H	2.712016204	2.043809211	-2.396238397
H	-2.173964477	0.688847524	-2.567753164
C	-1.952272758	1.544118926	-3.211354297
H	1.997250713	0.444577142	-2.116294135
C	-0.43160655	1.743628109	-3.399393915
H	0.167384964	-0.375105303	-3.244887822
H	-2.417586166	1.36217202	-4.19166732
C	0.187220436	0.447166482	-3.962121129
H	-0.723932253	3.780020059	-4.215853882
H	0.850143113	3.053672231	-4.631089241
C	-0.211233641	2.842099741	-4.456671709
H	-0.389234134	0.135621634	-4.844839197
H	1.223324852	0.606745427	-4.28918717
H	-0.631217845	2.485360682	-5.40901611
S	-0.537410293	-2.603135125	-1.112979517
O	0.454469329	-3.587225888	-1.56933912
O	-1.322952576	-2.907019648	0.098116391
O	0.042754627	-1.178354175	-1.138618893
C	-1.823272037	-2.572933129	-2.46848966
F	-2.7454495	-1.621163897	-2.239491404
F	-1.259837825	-2.340031043	-3.666191176
F	-2.439454469	-3.760077889	-2.512077947
N	-2.261569627	0.116269551	0.3222717
N	-3.395243952	0.042983002	0.433398719
N	-1.122029718	2.274780337	1.579479064
H	-2.140519907	2.171525427	1.53375032

H	-0.912597668	3.209841216	1.230559055
H	-0.887600513	2.28018887	2.574241073

Supplementary Table 22 | Cartesian coordinate of XVI. Units are presented in Å.

SCF energy (in toluene) = -1873.53202305 hartree

ZPE = 0.663820 hartree

Atom	X	Y	Z
Mo	-0.138937183	-1.752521887	-0.401938545
P	-0.268631959	0.437173404	0.645550084
N	-2.153806962	-1.847088081	0.499827701
P	-0.97716311	-3.720799675	-1.526405736
N	1.755671092	-1.778846737	-1.033080221
N	2.860932423	-1.823845355	-1.34436333
H	1.526897614	2.583091887	3.399548615
H	0.007052656	1.694390972	3.555140659
C	0.652250271	2.218083616	2.837893334
H	1.334791579	-0.446009364	3.042018609
H	0.110316317	3.096229922	2.477028892
H	2.77887849	0.560766805	2.915616518
C	1.940623863	0.143247874	2.337328673
H	2.985716128	2.358167041	1.324906019
C	1.128710373	1.291589145	1.700180493
H	-0.912676583	-0.317253995	2.838911042
H	2.355256147	-0.521085106	1.57010795
H	-2.090017988	0.901120948	2.380040454
C	2.085713242	2.059059911	0.765861402
H	-0.444871067	3.707213247	0.517020265
H	2.410449836	1.437072707	-0.078234687
C	-1.527384079	0.035885927	1.997026036
H	-3.887462327	-0.575166497	3.133066699
H	1.635360866	2.975044818	0.367039892
H	-1.907298411	3.051739184	1.286639039
H	-0.809151388	-5.17012947	1.030405785
C	-1.382540497	3.172385175	0.329674714
H	0.990420073	-6.913749363	-0.743116167
C	-3.681249611	-1.227269524	2.286224777
H	-2.004717497	3.826028855	-0.301670492
C	-2.489899708	-1.05444621	1.57796369
H	-1.048086971	-6.872604619	0.603519675
H	1.343410259	-5.197508317	-0.440978663
C	-1.264958794	-5.839789426	0.290427184
C	0.808506644	-5.889698941	-1.104561735

H	-5.509368682	-2.378494488	2.471540156
C	-1.162286109	1.8277227	-0.385591771
H	-2.35126798	-5.700525138	0.346297106
C	-4.585465681	-2.223792539	1.9186769
H	1.268091286	-5.802804934	-2.095012425
H	0.623790599	2.501229368	-1.502216446
H	-3.208561572	1.13643358	0.081667194
C	-0.707802786	-5.604796641	-1.131093868
C	-0.36049628	2.05671729	-1.684702362
C	-2.54805126	1.272634365	-0.783395697
H	-3.039492639	1.983000018	-1.465769227
H	-1.281057666	-7.622690738	-1.704839153
C	-4.273953926	-2.999196756	0.797653387
H	-0.202085835	1.113963107	-2.224586996
C	-3.089098084	-2.781976185	0.099310009
C	-1.393566227	-6.595791993	-2.087924103
H	-2.469663856	-6.402084432	-2.188563692
H	-0.91464293	2.737780862	-2.349078219
H	-4.96371883	-3.76186834	0.441388733
H	-2.468517826	0.307726321	-1.298950876
H	0.560608448	-4.986015339	-4.004362483
C	-2.826801929	-3.503604663	-1.198638988
H	-0.947923046	-6.575504919	-3.088872503
H	-3.413920572	-4.429946897	-1.25422156
H	1.289380457	-3.492637516	-3.364170199
C	0.469948318	-3.893010008	-3.973979058
H	-3.192528576	-2.860619136	-2.012748682
C	-0.905655841	-3.443777592	-3.443826558
H	-0.204582107	-1.406909671	-2.993761805
H	0.608630237	-3.529276099	-5.003797872
C	-0.995496958	-1.905472841	-3.582774737
H	-1.978758246	-5.171407428	-4.301480748
H	-3.024881471	-3.783017086	-3.957903623
C	-2.022613045	-4.077924389	-4.294593117
H	-0.841854039	-1.610713131	-4.632697857
H	-1.973611348	-1.512206918	-3.276893632
H	-1.922434554	-3.741331046	-5.338597824
H	1.220852771	-2.295904308	2.034363338
N	0.549156108	-2.823145325	1.473963122
H	0.999966307	-3.705075772	1.227176976
H	-0.24184852	-3.051980654	2.081388699

Supplementary Table 23 | Cartesian coordinate of XVII. Units are presented in Å.

SCF energy (in toluene) = -3909.60899623 hartree

ZPE = 1.314194 hartree

Atom	X	Y	Z
Mo	-2.615071018	0.048465227	-0.107133721
Mo	2.64334993	-0.093101933	-0.030091376
P	3.259671686	2.060792396	-1.170605418
P	-3.256994285	-2.111725063	-1.245982266
N	-4.848854948	0.138013293	-0.057795965
N	4.859755984	-0.208053674	-0.068351916
P	3.060084175	-2.296079687	1.154199534
P	-3.028481872	2.255828683	1.079411288
N	-0.539082574	-0.00448716	-0.113389541
N	0.618324799	-0.026432017	-0.072525259
N	-2.565028023	1.485514588	-2.929041299
N	-2.587053814	0.959030902	-1.918782154
N	-2.596442958	-0.869321355	1.707510494
N	-2.590413874	-1.401465744	2.712856535
N	2.715804552	0.779442891	1.73831046
N	2.77917287	1.291959135	2.764130509
H	2.670606158	3.749263408	-4.664837062
H	-2.737857574	-3.711293722	-4.781217592
H	-4.085748856	-2.702106807	-4.250865532
H	4.043696947	2.747822903	-4.184514789
C	3.279316298	3.436678064	-3.801938873
C	-3.327489908	-3.413167759	-3.900310992
H	-2.399561399	-0.832724694	-3.902311361
H	2.385501748	0.865280448	-3.877816529
H	-3.84119839	-4.30718857	-3.536990595
H	3.787036636	4.327839965	-3.425304685
H	-1.118420929	-1.943914069	-4.403353488
H	1.079703626	1.964532097	-4.314091537
C	1.664322539	1.583219432	-3.463743567
C	-1.689152749	-1.579770873	-3.534652487
H	0.614309032	3.975498868	-3.195610198
H	-0.658395934	-3.994999368	-3.353705421
C	2.362274968	2.763386251	-2.758652014
C	-2.383590862	-2.777977946	-2.857151972
H	-4.564392543	-0.825375709	-2.802508271
H	4.548282695	0.842455244	-2.807809054
H	0.97485274	1.061734101	-2.79150284
H	-1.001399715	-1.069906091	-2.850125179
H	5.545307983	2.199153991	-2.297131279

H	-5.55690529	-2.213603769	-2.341505384
C	1.250410019	3.741287779	-2.328831806
C	-1.279923356	-3.782624363	-2.470261575
H	3.64713027	5.277220248	-1.557106387
H	-3.672779631	-5.30895293	-1.681075153
H	0.60581025	3.302521092	-1.556855597
H	-0.623951697	-3.378523885	-1.687496956
C	4.873572269	1.413828583	-1.925001941
C	-4.880992843	-1.442537661	-1.948938477
H	7.546201341	1.039736	-1.728260567
H	-7.556705234	-1.117784467	-1.665611154
H	1.645942677	4.689555988	-1.951107198
H	-1.678486963	-4.740239958	-2.120155646
H	5.228644144	4.467560491	-1.636418974
H	-5.251030671	-4.490685893	-1.687062983
H	-4.495205052	2.574187776	-1.462595771
H	4.467199025	-2.627259757	-1.371215465
C	4.416138753	4.771745561	-0.963463104
C	-4.418269425	-4.814581761	-1.049310373
H	-2.641680416	5.136582727	-1.425955411
H	2.66629935	-5.189537974	-1.350287028
C	7.004360979	0.45012711	-0.991290275
C	-7.001158541	-0.533092973	-0.935530757
H	4.834036012	5.521979406	-0.274535866
H	-4.816258872	-5.578773594	-0.36401783
C	5.610814165	0.514012537	-0.965938373
C	-5.606112714	-0.578733347	-0.948112745
H	-4.881990735	4.299397294	-1.556954777
H	4.827749733	-4.365033394	-1.52505583
H	-2.023140271	3.479813303	-1.494492596
H	1.808715149	-3.645894121	-1.147344948
C	-4.702292933	3.463805805	-0.863559562
C	4.679129653	-3.542227404	-0.808950839
C	-2.336873086	4.274545507	-0.812491113
C	2.33815956	-4.442212517	-0.61142559
H	-8.752980667	0.29211965	0.034265933
H	8.774024357	-0.408095093	-0.079749999
C	3.863181995	3.594325974	-0.136273297
C	-3.831788581	-3.656983879	-0.216739127
H	-5.632261412	3.296094749	-0.30804012
H	5.633292231	-3.378724948	-0.296493258
C	-7.666225046	0.248699129	0.008825547
C	7.687807868	-0.350480253	-0.075374554
H	-1.468556189	4.582934077	-0.222778084
H	1.597537961	-4.925641806	0.032273278
H	1.861143167	4.48440967	0.158117612
H	-1.832176254	-4.576689176	-0.001339418
H	5.909662007	2.858096505	0.242069736
H	-5.868936571	-2.943332544	0.238983814
C	-3.528073702	3.838632123	0.068438391
C	3.556008635	-3.921661089	0.185023363
C	2.699937147	4.09738419	0.745393674

C	-2.645121131	-4.186096113	0.618447239
C	5.002074211	3.123946303	0.796981831
C	-4.93895794	-3.198997468	0.759848304
H	5.262662981	3.953378626	1.471863155
H	-5.165585265	-4.028525283	1.44647694
H	-4.3593475	5.828036895	0.245926563
H	4.454159247	-5.887364223	0.381811914
C	6.931171863	-1.063329949	0.857312054
C	-6.897633607	0.972896396	0.91990406
H	2.326760218	3.311864474	1.407309944
H	-2.233025277	-3.416168523	1.276028122
C	5.539195403	-0.979569453	0.846273711
C	-5.504065298	0.908389742	0.868039249
C	-3.990477814	5.03850437	0.918651409
C	4.102310215	-5.079762475	1.043545277
H	-4.816077471	4.780323887	1.594719976
H	4.958188929	-4.773264368	1.658226266
H	3.062932514	4.921240577	1.379521644
H	-2.99629284	-5.012288003	1.255653381
H	-7.37007062	1.597483945	1.675003459
H	7.415333889	-1.689224079	1.604326286
H	4.711343583	2.273396972	1.416794934
H	-4.638415615	-2.341214881	1.36544488
H	-1.208964972	4.742080608	1.886608845
H	1.362694904	-4.846277118	2.116308427
C	-4.669095165	1.704467815	1.839642859
C	4.723283345	-1.743905491	1.858323598
H	-3.181889032	5.475293127	1.51357181
H	3.343922176	-5.513172287	1.70335908
H	-5.264286551	2.523285936	2.264475019
H	5.323612987	-2.553467943	2.293961752
H	-0.273171342	3.298243018	1.425881901
H	0.353218061	-3.478371137	1.58291911
C	-0.874641242	3.754146385	2.221814894
C	0.98646355	-3.855826686	2.395722857
H	-4.367905454	1.057400696	2.676609116
H	4.445047306	-1.066903291	2.679065524
C	-2.038760074	2.838627768	2.649291162
C	2.110672392	-2.864043749	2.757275212
H	-0.772967134	1.042408053	2.599574723
H	0.768830988	-1.128678299	2.640573478
H	-0.208856174	3.909074499	3.083107638
H	0.338453532	-3.992051299	3.273814817
C	-1.41632173	1.586253612	3.298166725
C	1.437495132	-1.614096566	3.358707048
H	-3.360625284	4.468144251	3.36973786
H	3.531671172	-4.389183268	3.517496799
H	-3.684813948	2.889821525	4.111996143
H	3.765527246	-2.770431755	4.205862028
C	-2.894891821	3.543964524	3.721987946
C	3.013761797	-3.482740573	3.844784283
H	-0.796388785	1.902386529	4.150295144

H	0.839544574	-1.921137468	4.230341363
H	-2.171326047	0.892396465	3.681967469
H	2.161604027	-0.867715873	3.698283634
H	-2.248844647	3.806043097	4.573732743
H	2.39108964	-3.755957265	4.710517817
N	2.431015879	-1.197238412	-2.134082584
H	2.636854754	-2.193617533	-2.088620738
H	2.973660841	-0.828016373	-2.915339023
H	1.448015151	-1.113562321	-2.400858067

Supplementary Table 24 | Cartesian coordinate of XVIII. Units are presented in Å.

SCF energy (in toluene) = -3853.04790381 hartree

ZPE = 1.273448 hartree

Atom	X	Y	Z
Mo	-2.562034206	0.030977988	-0.006149736
Mo	2.674593022	-0.228681369	-0.238041528
P	3.181279597	1.820472758	-1.522609474
P	-3.133965873	-1.954366333	-1.497529218
N	-4.796945202	0.121279208	-0.0581995
N	4.920084413	-0.281036126	-0.278739324
P	3.111200153	-2.066975571	1.338869504
P	-3.03903636	2.046858694	1.459635426
N	-0.499927308	-0.059403703	-0.024084473
N	0.655918576	-0.116024817	-0.103156886
N	-2.477766836	1.857854676	-2.593965847
N	-2.495189973	1.195493906	-1.66892309
N	-2.646458056	-1.139463827	1.650471637
N	-2.719601641	-1.806876582	2.569989335
N	2.664683627	-2.010707006	-2.762065512
N	2.672045774	-1.339543785	-1.830061928
H	2.582679774	3.382708752	-5.043390273
H	-2.448621458	-3.024624839	-5.185622197
H	-3.826053538	-2.100005635	-4.580376097
H	4.032852646	2.546656128	-4.482711941
C	3.178634245	3.148273652	-4.147950293
C	-3.08067144	-2.855451188	-4.300681927
H	-2.1825315	-0.303763754	-3.895943189
H	2.610309078	0.418526515	-4.106146486
H	-3.60520792	-3.791166945	-4.091155671
H	3.561676623	4.094737883	-3.75542262
H	-0.854477243	-1.319415328	-4.476554727
H	1.255854903	1.362892373	-4.748924296
C	1.793676185	1.094833707	-3.826941732
C	-1.476938735	-1.087230596	-3.599936845
H	0.440761704	3.379447555	-3.691535299
H	-0.421447652	-3.493461989	-3.722768151
C	2.290101317	2.385444637	-3.14373915
C	-2.184265718	-2.375968846	-3.139163671
H	-4.373713512	-0.442997444	-2.900498717
H	4.582674913	0.67322177	-3.088745097
H	1.111316726	0.535780541	-3.180458628
H	-0.825402141	-0.684482407	-2.818834695
H	5.484612331	2.094803442	-2.560220254

H	-5.378006029	-1.879117538	-2.686916239
C	1.053641227	3.234756203	-2.789896297
C	-1.086156023	-3.420293738	-2.850282698
H	3.292287046	5.050752624	-1.858078279
H	-3.499530323	-5.052622466	-2.388233404
H	0.421889432	2.73564872	-2.045022975
H	-0.463246929	-3.127384081	-1.996125964
C	4.848305161	1.272420146	-2.205374625
C	-4.722866546	-1.174980397	-2.158028922
H	7.505747658	0.870134497	-2.150836826
H	-7.408225449	-0.875577296	-1.974154691
H	1.319544856	4.229739459	-2.413306815
H	-1.490208567	-4.420768794	-2.661001676
H	4.946240453	4.400890802	-1.859016823
H	-5.077853828	-4.234320118	-2.384448478
H	-4.369769965	2.811171634	-1.105530047
H	4.153280567	-3.378625977	-1.064415006
C	4.076858096	4.621143546	-1.225662812
C	-4.288148511	-4.649899922	-1.745281945
H	-2.482930938	5.217840729	-0.619359602
H	1.862124104	-5.332537165	-0.179346409
C	7.006412881	0.307610667	-1.364586486
C	-6.894389079	-0.402905396	-1.140007966
H	4.382756223	5.402124647	-0.512064346
H	-4.725514469	-5.498865222	-1.19802921
C	5.619226613	0.402947568	-1.243057798
C	-5.500369599	-0.458600851	-1.081163447
H	-4.757505595	4.522478543	-0.889614153
H	4.274311986	-5.090251972	-0.623462007
H	-1.852402127	3.574672425	-0.817955059
H	1.644837824	-3.653478433	-0.715848735
C	-4.59936922	3.572642247	-0.357106251
C	4.291117801	-4.065134713	-0.223334481
C	-2.216377894	4.295367547	-0.081101475
C	1.829095943	-4.285456304	0.159283606
H	-8.697543043	0.294958582	-0.165826747
H	8.813174538	-0.606275838	-0.592583059
C	3.596519219	3.381154615	-0.450646934
C	-3.758318954	-3.622811367	-0.725314537
H	-5.547263671	3.307726455	0.125759258
H	5.2886901	-3.898216465	0.202056639
C	-7.611065149	0.246924308	-0.135873049
C	7.733657004	-0.509112466	-0.499542441
H	-1.390319374	4.538017266	0.594861514
H	0.977337473	-4.184751247	0.841656204
H	1.50710575	4.07340421	-0.238095301
H	-1.767855439	-4.565400333	-0.542304391
H	5.664768671	2.773266168	0.033241815
H	-5.823525812	-2.983992279	-0.292297237
C	-3.460482441	3.771097169	0.669310693
C	3.170570452	-3.925376001	0.831879741
C	2.346325348	3.741303606	0.381732658

C	-2.611635536	-4.266416858	0.08674989
C	4.712041467	2.974864708	0.537195516
C	-4.922118248	-3.306647919	0.241810413
H	4.88086382	3.796182254	1.250188312
H	-5.179345064	-4.224021511	0.792595266
H	-4.246112227	5.740713637	1.097690328
H	3.584269373	-5.921086336	1.566271511
C	7.031729521	-1.188629483	0.495949042
C	-6.89603104	0.833769649	0.9075279
H	2.000345697	2.884415108	0.975823771
H	-2.234590942	-3.597237872	0.864794288
C	5.648206861	-1.046321148	0.599513429
C	-5.501693512	0.764880155	0.924634857
C	-3.939203388	4.847819034	1.663653552
C	3.455387867	-4.909251375	1.980601941
H	-4.810785776	4.521391844	2.245808264
H	4.376018756	-4.661829999	2.525418523
H	2.590150758	4.557174787	1.079566625
H	-2.990253951	-5.173246196	0.582981459
H	-7.410708502	1.35290664	1.713120864
H	7.552394821	-1.819747319	1.21315523
H	4.442763362	2.078766616	1.111036943
H	-4.667110267	-2.540135546	0.977049395
H	-1.24205839	4.353951294	2.725602495
H	0.883707914	-3.546695968	3.01620785
C	-4.722326846	1.415362469	2.040312191
C	4.91179582	-1.678084832	1.752189728
H	-3.156338918	5.163257298	2.360479787
H	2.631789002	-4.957441405	2.701956393
H	-5.340092931	2.175207209	2.536351219
H	5.474514933	-2.534318142	2.147055778
H	-0.303890602	2.968234821	2.127626294
H	0.304436282	-2.085477714	2.186007268
C	-0.947848184	3.321807683	2.94322607
C	0.875515414	-2.450580418	3.047665998
H	-4.474521182	0.658712644	2.799033797
H	4.865373555	-0.9388189	2.566323646
C	-2.152073856	2.382997181	3.160509815
C	2.298991614	-1.862054009	3.080265886
H	-0.936484141	0.570141058	2.917521243
H	1.578625389	0.129256885	2.489336961
H	-0.339294301	3.345362333	3.859815285
H	0.327950899	-2.155702592	3.955087277
C	-1.592284817	1.036654599	3.659241047
C	2.183365088	-0.333071243	3.281138216
H	-3.496456122	3.91794627	4.034751795
H	3.162736642	-3.536776464	4.23442205
H	-3.886860604	2.253966218	4.507758967
H	4.095417104	-2.033645744	4.350122241
C	-3.065637452	2.942216219	4.271848932
C	3.081356239	-2.446707046	4.273377423
H	-1.008908453	1.213901949	4.575801552

H	1.689731212	-0.120574025	4.241509963
H	-2.380928914	0.319936328	3.910427325
H	3.163551972	0.164132583	3.304690655
H	-2.47305567	3.060686925	5.192039808
H	2.558421211	-2.191713211	5.208132432

Supplementary Table 25 | Cartesian coordinate of XIX. Units are presented in Å.

SCF energy (in toluene) = -1926.49168292 hartree

ZPE = 0.636867 hartree

Atom	Coordinates (Angstroms) X	Y	Z
Mo	-2.710355514	-0.010499466	-0.00026362
P	-3.176757357	2.166886364	1.162682403
N	-4.8657873	-0.019120168	-0.000685088
P	-3.159000725	-2.191597472	-1.164680654
N	-2.523750295	-1.460698013	2.804388677
N	-2.612751224	-0.922188471	1.802535277
N	-2.617934544	0.901819174	-1.802945522
N	-2.531898127	1.440739355	-2.804842053
H	-2.505526961	3.733237914	4.681119516
H	-3.889101192	2.752011255	4.194297878
C	-3.12765102	3.446950467	3.819125764
H	-2.271231142	0.837921248	3.771264127
H	-3.633920982	4.350900708	3.469867974
H	-0.947398821	1.908455319	4.250811946
C	-1.550741423	1.57032626	3.39457229
H	-0.464693686	3.958422477	3.21805025
C	-2.229373517	2.793951202	2.74755613
H	-4.484121327	0.918543104	2.746460515
H	-0.885355729	1.055231298	2.691648757
H	-5.451500265	2.336579151	2.320536768
C	-1.107507473	3.772528465	2.344813988
H	-3.473770083	5.372498092	1.614986764

H	-0.471365409	3.359037459	1.550701867
C	-4.80610366	1.550808618	1.90604522
H	-7.490224229	1.318663234	1.678437978
H	-1.487103803	4.743542979	2.010890417
H	-5.079287841	4.610333132	1.644050261
H	-4.567031198	-2.479780223	1.415232324
C	-4.246159414	4.907401171	0.993596999
H	-2.814203372	-5.086930144	1.331196024
C	-6.973898815	0.718991229	0.932019921
H	-4.628001817	5.68782011	0.317613317
C	-5.578396805	0.71750554	0.916163225
H	-5.021889288	-4.188728637	1.497327165
H	-2.12265031	-3.459283747	1.371690052
C	-4.822829951	-3.352563343	0.810197346
C	-2.488341477	-4.24359986	0.703208507
H	-8.776665226	-0.035404818	-0.000643399
C	-3.71295986	3.733847815	0.147881231
H	-5.756548389	-3.140413052	0.276719694
C	-7.688892651	-0.030822594	-0.000703991
H	-1.649148501	-4.59537999	0.095940285
H	-1.685636919	4.58446332	-0.09710577
H	-5.779866381	3.092075646	-0.280337526
C	-3.68366227	-3.762852918	-0.150092863
C	-2.521017686	4.224212032	-0.704681884
C	-4.848376029	3.315162378	-0.813275252
H	-5.055792152	4.151476324	-1.497736172
H	-4.584392139	-5.723383154	-0.319550379
C	-6.967689877	-0.774547882	-0.933524281
H	-2.147467158	3.442355375	-1.371700655
C	-5.572250921	-0.761388932	-0.91769891

C	-4.207235611	-4.940802606	-0.995650521
H	-5.041496099	-4.650211856	-1.647581789
H	-2.853771953	5.063735337	-1.334140504
H	-7.47899307	-1.378463222	-1.679982107
H	-4.584088201	2.44692935	-1.421236657
H	-1.448027874	-4.754207834	-2.013124364
C	-4.79301649	-1.587876592	-1.907915466
H	-3.430556418	-5.400769563	-1.615496241
H	-5.431889939	-2.378695898	-2.322870017
H	-0.444117025	-3.360960876	-1.553236321
C	-1.07684293	-3.779965414	-2.347184382
H	-4.476023204	-0.9524521	-2.747846258
C	-2.207104069	-2.811051265	-2.749725667
H	-0.878135376	-1.060907385	-2.694540012
H	-0.432746536	-3.960275434	-3.220628454
C	-1.539308908	-1.58172858	-3.397210187
H	-3.598725454	-4.379951624	-3.470449678
H	-3.86808666	-2.783729598	-4.195463936
C	-3.100424096	-3.471942732	-3.820549121
H	-0.93345711	-1.914690094	-4.253708004
H	-2.266257698	-0.855541253	-3.773558624
H	-2.47648974	-3.753225505	-4.682885984

Supplementary Table 26 | Cartesian coordinate of XIX'. Units are presented in Å.

SCF energy (in toluene) = -1926.51501551 hartree

ZPE = 0.635509 hartree

Atom	X	Y	Z
Mo	-2.823392577	-0.297373761	-0.117155257
P	-2.959511308	1.956761547	0.86075548
N	-5.001449443	-0.213743216	0.428289921
P	-3.620596518	-2.327617328	-1.232000062
N	-2.297791738	-1.631859269	2.62445636
N	-2.471778093	-1.131754545	1.608633977
N	-0.881421448	-0.369678098	-0.642125623
N	0.213264266	-0.405531562	-0.962860586
H	-1.514545657	4.025236012	3.826078923
H	-3.04726976	3.150071315	3.78539391
C	-2.309298209	3.662899398	3.156267944
H	-1.776654846	0.922657469	3.398079498
H	-2.790572459	4.541307665	2.71518575
H	-0.310969982	1.90265722	3.566438366
C	-1.047424783	1.522976041	2.841975403
H	0.235636256	3.709096655	2.038523457
C	-1.689812456	2.718119032	2.106525937
H	-3.9936812	1.173757488	2.875788499
H	-0.532564386	0.852689564	2.145562331
H	-4.962365231	2.540346148	2.323443024
C	-0.571718268	3.449431446	1.337761425
H	-2.930942395	5.190956471	0.649498483
H	-0.136908071	2.815008099	0.554984814
C	-4.441065031	1.634808992	1.982937003
H	-7.052223231	1.37739734	2.607869315
H	-0.915333769	4.38343806	0.878438011
H	-4.561890225	4.653272354	1.105056503
H	-4.043972741	-3.240937205	1.508906791
C	-3.843194261	4.71280073	0.277066644
H	-2.004155088	-5.338663046	0.437054946
C	-6.756055073	0.664761356	1.841283974
H	-4.275282849	5.384198327	-0.481159943
C	-5.434191583	0.662599685	1.388597937
H	-4.241407956	-4.995637652	1.372556632
H	-1.670190398	-3.600697082	0.591040264
C	-4.368143649	-4.042515474	0.837801687
C	-2.063568287	-4.370115043	-0.082131025
H	-8.700720535	-0.270772741	1.679318847
C	-3.571017091	3.335898799	-0.354445777

H	-5.441178464	-3.919939414	0.642421726
C	-7.672216697	-0.249569364	1.324958935
H	-1.406772722	-4.425230489	-0.957936189
H	-1.572165752	3.874242941	-1.134482668
H	-5.706311589	2.768402853	-0.268611932
C	-3.53315967	-4.087169167	-0.462597865
C	-2.529532934	3.477196743	-1.486555003
C	-4.885526115	2.83529198	-0.99254541
H	-5.195291546	3.538530145	-1.780226176
H	-4.072977813	-6.161346717	-0.778643566
C	-7.237618948	-1.129922645	0.335005598
H	-2.329171165	2.510992691	-1.969088033
C	-5.915076396	-1.081749166	-0.108900588
C	-4.069185409	-5.222203431	-1.352774514
H	-5.099708069	-5.041687556	-1.686701954
H	-2.912172052	4.164955662	-2.255861911
H	-7.922052587	-1.847199052	-0.112367131
H	-4.766743919	1.846543749	-1.454285425
H	-1.805573566	-4.051389262	-3.134654351
C	-5.474332064	-1.947953707	-1.264040273
H	-3.444445799	-5.386996842	-2.238078596
H	-6.109475832	-2.840271384	-1.339624317
H	-1.075412889	-2.488898823	-2.699896615
C	-1.826201316	-2.973704507	-3.335881683
H	-5.639227496	-1.378287781	-2.190699586
C	-3.229608947	-2.372114328	-3.122205721
H	-2.4163292	-0.333845827	-2.978460214
H	-1.5203477	-2.831908202	-4.383430411
C	-3.181710955	-0.884923524	-3.544389498
H	-4.315009819	-4.171907105	-3.796486672
H	-5.260735605	-2.678514218	-3.930352826
C	-4.251622817	-3.102045487	-4.015752133
H	-2.918497685	-0.807095824	-4.610139746
H	-4.148427002	-0.378057884	-3.41487108
H	-3.949673803	-3.001264587	-5.069695012

Supplementary Table 27 | Cartesian coordinate of XX. Units are presented in Å.

```
SCF energy (in toluene) = -1983.07761485 hartree

ZPE =  0.675588 hartree

-------------------------------------------------

 Atom            Coordinates (Angstroms)

            X               Y               Z

-------------------------------------------------

  Mo   -0.152898577  -1.480244295   -0.773851492
   P   -0.293363018    0.56371018    0.679897559
   N   -2.117150883  -1.775287798     0.22775867
   P   -0.926135757  -3.642730945   -1.828355833
   N    1.667860116  -1.272300771   -1.592632524
   N    2.738927203  -1.206767523   -1.989093174
   H    1.696616676   2.144426273    3.671088457
   H    0.271251533   1.103765114    3.761007759
   C    0.784678227   1.842951393    3.132206875
   H    1.728491836  -0.643515682    2.724657716
   H    0.145727289   2.725057384    3.058213011
   H    3.056027236   0.491711701    2.532730438
   C    2.158336491   0.097420811    2.033832068
   H    2.895703519   2.556589541    1.463458821
   C     1.18009505   1.257351935    1.760305656
   H   -0.667202549  -0.845217785    2.612024423
   H    2.476097891  -0.396447933      1.1091428
   H   -1.844887202   0.463070274    2.707063875
   C    1.959864093     2.3073353    0.940749836
   H   -0.599790581    3.74060149    1.400602016
   H    2.225718287   1.926362877   -0.053935936
   C    -1.37345518  -0.247158259    2.014677089
   H   -3.841790757  -0.816775823    2.986477192
   H    1.403599868   3.241334999    0.813939862
   H   -1.933357318   2.823497835    2.140181792
   H   -1.136567655  -4.278406005    1.024912104
   C    -1.53130542   3.204257666    1.191862223
   H    0.697792331  -6.648862888   -0.047226735
   C   -3.652027215  -1.326891287    2.044419224
   H   -2.249915026   3.946268585    0.811680557
   C   -2.419747256  -1.152018667    1.411248456
   H   -1.251759196  -6.050849571    1.125822998
   H    1.247300985  -4.977023624   -0.271307832
   C   -1.481963771  -5.175253961    0.500015394
   C    0.638007848  -5.773495083   -0.712127733
   H   -5.590776107  -2.286033364    1.944703891
   C   -1.363588911   2.095247588    0.133219231
   H    -2.57315677  -5.096883898    0.437680468
   C   -4.624803442  -2.141367572    1.465493314
```

H	1.113954922	-6.049203497	-1.657566982
H	0.247808946	3.133159603	-0.970320685
H	-3.330537282	1.262791036	0.684592354
C	-0.839976557	-5.360656901	-0.895666896
C	-0.746628536	2.709450582	-1.143749415
C	-2.784249285	1.589544878	-0.208414267
H	-3.351607015	2.422626609	-0.64996662
H	-1.568073827	-7.404029374	-0.892476724
C	-4.328676845	-2.756156846	0.24825998
H	-0.673340387	1.979553722	-1.95366815
C	-3.08213177	-2.562324169	-0.349369403
C	-1.588881951	-6.531235287	-1.564220555
H	-2.643648501	-6.294388852	-1.752242842
H	-1.394747465	3.528487721	-1.491741803
H	-5.061990978	-3.387347709	-0.249033201
H	-2.782377872	0.774086547	-0.934133946
H	0.386013753	-5.928818612	-3.636530283
C	-2.761630125	-3.215768624	-1.672427561
H	-1.132904032	-6.843124234	-2.509103834
H	-3.441722778	-4.059634824	-1.850784449
H	1.39651782	-4.470378166	-3.491309571
C	0.506730732	-4.894219173	-3.9749009
H	-2.932098588	-2.487185804	-2.478181352
C	-0.74458018	-4.025749706	-3.728369373
H	0.354968758	-2.149394674	-4.057467101
H	0.707994567	-4.930278229	-5.055744278
C	-0.51355914	-2.685049045	-4.454234309
H	-2.222552436	-5.669006797	-3.933766161
H	-2.871539434	-4.056661371	-4.28733501
C	-1.981745281	-4.693504813	-4.364707048
H	-0.335868503	-2.891266677	-5.520845449
H	-1.37593985	-2.015810835	-4.387517013
H	-1.790743589	-4.847083672	-5.437823897
H	1.951926439	-2.876479332	0.463522012
N	1.040804884	-2.683171438	0.886691685
H	0.645497827	-3.586270324	1.145833123
H	1.233799028	-2.1872557	1.75545544
N	-1.072707171	-0.528709531	-2.245503266
N	-1.642862429	0.013915347	-3.079168521

Supplementary Table 28 | Cartesian coordinate of XXI. Units are presented in Å.

SCF energy = -2035.41892234 hartree (BS1)

ZPE = 0.654866 hartree

Atom	X	Y	Z
Mo	2.253304433	1.763908953	1.595322264
P	2.772655486	-0.70658602	2.231955693
N	4.449473132	1.570339988	1.08363936
P	2.757593984	4.085394413	0.560093557
N	-0.889984676	2.121920524	2.035342973
N	0.217937739	1.988401951	1.881983029
N	2.652641221	2.802006608	4.43711244
N	2.508855014	2.315256905	3.327044459
N	1.630656224	1.032561112	-0.385463841
N	1.230547961	0.669679351	-1.363482858
H	2.632835199	-2.792849247	5.495508699
H	4.00783804	-1.874833761	4.880940157
C	3.098831868	-2.408320364	4.577123275
H	2.664290115	0.23176081	5.122513846
H	3.392332569	-3.272110277	3.977449064
H	1.356528672	-0.760517751	5.769003383
C	1.764080142	-0.329364679	4.844153204
H	0.298256784	-2.486113343	4.544970017
C	2.088717389	-1.47848684	3.8695263
H	4.511173932	0.167765136	3.652431527
H	1.015284289	0.358721611	4.43483356
H	5.17579971	-1.221817451	2.793596627
C	0.77563096	-2.237395471	3.58716655
H	2.812254593	-3.935723327	2.082984539
H	0.062395003	-1.633116151	3.011712556
C	4.561263367	-0.322352899	2.669402146
H	7.167280755	-0.255001621	1.936728632
H	0.939662285	-3.181375174	3.058103987
H	4.457459499	-3.286574869	1.893186522
H	4.545469314	3.821589588	2.938723071
C	3.487283676	-3.414236411	1.39763748
H	3.031980573	6.518929346	3.494108681
C	6.585578339	0.509380284	1.428073501
H	3.649052941	-4.080765087	0.538619352
C	5.217412394	0.618452275	1.688804695
H	5.177333243	5.454973238	3.160020478
H	2.240274802	4.941603069	3.432563169
C	4.791309614	4.747370507	2.413261155
C	2.548524184	5.795005041	2.822698269

H	8.251483271	1.306988624	0.30676734
C	2.899856182	-2.084631627	0.87956114
H	5.605002945	4.549818647	1.705499437
C	7.187730218	1.379110487	0.522144411
H	1.65543538	6.277047085	2.415640703
H	0.834868895	-2.85842376	0.992597209
H	4.881115711	-1.512876794	0.094393233
C	3.564712643	5.391377626	1.730474865
C	1.502714518	-2.366506974	0.280716558
C	3.83989899	-1.580544929	-0.240891594
H	3.813127008	-2.303924407	-1.067440029
H	4.605887416	7.278785672	1.708673216
C	6.399841129	2.341129207	-0.103423533
H	1.000023564	-1.464753751	-0.077550223
C	5.036065404	2.420830112	0.19315459
C	4.052571047	6.65490776	0.992738362
H	4.738788064	6.427553701	0.167231669
H	1.616235597	-3.042549738	-0.578247621
H	6.831693393	3.034310164	-0.82058407
H	3.5516981	-0.608196112	-0.649791392
H	1.114010324	6.791714956	0.411252579
C	4.179196682	3.452351936	-0.501617032
H	3.231634303	7.265557677	0.604577944
H	4.808208047	4.264541907	-0.884133545
H	0.092669396	5.388104146	0.799899074
C	0.631410531	5.902528902	-0.006052401
H	3.702781253	2.989315157	-1.379290995
C	1.622897099	4.96886757	-0.732135908
H	0.167457021	3.316003315	-0.777447504
H	-0.117139759	6.251640751	-0.730498274
C	0.796042625	3.893301654	-1.463863204
H	3.022390752	6.562607116	-1.387845103
H	3.053718289	5.10885359	-2.405460905
C	2.409051489	5.759090193	-1.801278505
H	0.129532244	4.393021441	-2.180378668
H	1.421051598	3.203846655	-2.042962316
H	1.688591307	6.220618182	-2.491383069
H	2.206802864	2.273621368	5.205239783

Supplementary Table 29 | Cartesian coordinate of XXII. Units are presented in Å.

SCF energy (in toluene) = -2724.02957112 hartree

ZPE = 0.662558 hartree

Atom	X	Y	Z
Mo	-0.486151315	0.056417154	-0.130749294
P	-1.45945757	-2.257989173	0.536902636
N	-2.046892262	-0.219131363	-1.729957396
P	-0.760522602	2.413786967	-0.937488039
N	0.9976722	-0.296171455	-0.917103535
H	-0.370321203	-5.83904272	-0.370268801
H	-2.00500484	-5.171977535	-0.33639149
C	-1.006336107	-5.082038684	0.109586797
H	-1.12253424	-3.610736726	-2.199044246
H	-1.071858257	-5.342079855	1.17037295
H	0.499152454	-4.282028222	-2.011636692
C	-0.182239756	-3.502584096	-1.64347656
H	1.65168359	-4.418628213	0.147188815
C	-0.368690029	-3.697571626	-0.12322723
H	-3.228234012	-3.247449981	-0.92680791
H	0.259675184	-2.531277365	-1.888830877
H	-3.786572549	-1.876913215	0.007189718
C	1.006509978	-3.637649599	0.57357894
H	-3.077753604	-4.69059879	1.718626582
H	1.496066707	-2.668773919	0.432497225
C	-2.947332636	-2.237775571	-0.604827019
H	-4.275878986	-2.465826179	-2.926371262
H	0.937118984	-3.812358196	1.651450262
H	-4.266508509	-3.398325131	1.451186088
H	1.944088901	1.917538311	-1.97058863
C	-3.435100895	-3.725613312	2.087249986
H	2.329917254	4.398243104	0.04163856
C	-3.665881812	-1.566737752	-2.903017502
H	-3.853056935	-3.894366328	3.089333904
C	-2.850366805	-1.322988283	-1.794800927
H	2.646747935	3.527651131	-2.174812486
H	1.884776941	2.745178958	0.49986376
C	1.705403423	2.962777082	-2.200906699
C	1.484171918	3.708941256	0.173124278
H	-4.346801003	-0.818964363	-4.811125975
C	-2.314939683	-2.668307669	2.215069365
H	1.327532205	3.021794908	-3.228759615
C	-3.711160643	-0.646277305	-3.945333983
H	0.86093345	4.103648194	0.979672017

H	-0.924588426	-4.16557313	3.028673948
H	-3.662525049	-0.926791824	1.975444232
C	0.738287875	3.589094314	-1.17215884
C	-1.280954442	-3.15331396	3.251559739
C	-2.949838398	-1.345181599	2.701819037
H	-3.517034071	-1.544153993	3.621544781
H	1.224864982	5.567353056	-1.888828975
C	-2.943412989	0.510911761	-3.848151407
H	-0.41630097	-2.490083409	3.3338496
C	-2.1163067	0.705596393	-2.739990515
C	0.31772453	4.981903378	-1.684144481
H	-0.253106921	4.931032072	-2.620281878
H	-1.766552279	-3.189111299	4.236356624
H	-2.968334466	1.262840285	-4.632351138
H	-2.199539653	-0.588080526	2.946233848
H	-1.213176924	4.913983591	0.89919684
C	-1.259768117	1.947072164	-2.679011042
H	-0.268154496	5.542496635	-0.948782436
H	-0.312034193	1.728835899	-3.188423622
H	-1.254061692	3.385708303	1.813493472
C	-1.819209607	4.032101727	1.131295702
H	-1.738041416	2.759377878	-3.239467796
C	-2.27346943	3.27578807	-0.135642939
H	-2.784215586	1.479076541	1.039200522
H	-2.711482352	4.38386577	1.667596725
C	-3.246145929	2.15747385	0.30688722
H	-2.391082892	5.042008061	-1.460429812
H	-3.428296139	3.689518336	-1.965713448
C	-3.017335655	4.22141125	-1.098002261
H	-4.108651056	2.616021813	0.810453091
H	-3.633578379	1.575273006	-0.536865135
H	-3.867483782	4.669097019	-0.564328759
S	0.596732523	0.195775147	3.032455166
O	-0.302971076	0.203826611	4.188613143
O	1.335662879	-1.02633891	2.683596075
O	-0.084375006	0.841786218	1.773926055
C	1.88456513	1.508701595	3.362653105
F	2.787319998	1.516657246	2.367153922
F	1.305187712	2.713525841	3.43505734
F	2.498106588	1.236906069	4.510534833
H	1.990107042	-0.468265333	-1.085707351

Supplementary Table 30 | Cartesian coordinate of XXIII. Units are presented in Å.

SCF energy (in toluene) = -2833.53000327 hartree

ZPE = 0.673437 hartree

Atom	X	Y	Z
Mo	-0.308541516	0.577312982	0.365987468
P	0.352379363	-0.912459408	2.466752952
N	1.843437955	0.992223993	0.661296892
P	0.260058095	2.066632293	-1.69182129
N	-0.944556502	1.829436646	1.391572068
H	-1.146593795	-1.700615145	5.9413488
H	0.432896082	-0.996863934	5.574005145
C	-0.349918554	-1.660921546	5.185399744
H	-0.960887802	0.96570834	4.521828766
H	0.059953749	-2.671040614	5.096862707
H	-2.411335529	0.040014512	4.912379482
C	-1.647022065	0.199348119	4.139763648
H	-2.820995124	-2.213318928	4.116928746
C	-0.955539235	-1.151864378	3.861873414
H	0.76798474	1.333013449	3.294253886
H	-2.158916137	0.585397854	3.252516859
H	2.031366126	0.274245119	3.940151878
C	-2.031580923	-2.138222647	3.355876424
H	2.088511664	-2.384725556	4.586950904
H	-2.490526457	-1.800950165	2.420883316
C	1.448835017	0.509811528	3.044200029
H	4.073439237	1.249542218	3.186745021
H	-1.639972107	-3.143421325	3.183820909
H	3.264140683	-1.508436865	3.581165701
H	0.394224554	3.652715838	0.89433081
C	2.584107543	-2.368262725	3.613019542
H	-0.944431913	5.626216144	-1.078405018
C	3.69113435	1.285682844	2.170316573
H	3.2126533	-3.267679876	3.55312784
C	2.358867368	0.943755824	1.926330165
H	0.947342645	5.2440645	0.343008491
H	-1.684882783	4.073315074	-0.653441755
C	0.977576899	4.167190847	0.126809114
C	-1.000158718	4.564935019	-1.356875363
H	5.556913199	1.913469668	1.283111258
C	1.610705405	-2.394561086	2.411787918
H	2.023768678	3.852376	0.21187304
C	4.514254925	1.656616787	1.109007495
H	-1.442902797	4.513991041	-2.357076116

H	0.380494945	-3.956637951	3.362135623
H	3.112499264	-1.402231893	1.132531419
C	0.415489467	3.952991001	-1.296274635
C	0.855558146	-3.740767305	2.398988969
C	2.467906498	-2.287832399	1.12937482
H	3.129586204	-3.164407037	1.094685852
H	1.471004197	5.729751671	-1.915259127
C	3.982963302	1.694438578	-0.17881286
H	0.098003799	-3.793270831	1.612401105
C	2.639527187	1.371084881	-0.379946977
C	1.369313902	4.692000778	-2.262126435
H	2.377082695	4.257216366	-2.259507272
H	1.584759898	-4.540854238	2.212395063
H	4.597624789	1.978122464	-1.028705097
H	1.877907565	-2.299985163	0.211904619
H	-2.463474692	2.480347581	-2.817445883
C	2.024985674	1.421637985	-1.752928306
H	1.01179804	4.729287101	-3.291285169
H	2.666868946	1.98140143	-2.44249129
H	-2.235776703	0.721690018	-2.67965678
C	-1.993626532	1.603290439	-3.279067076
H	1.941390059	0.397354185	-2.139955551
C	-0.467803559	1.769859355	-3.45491127
H	0.06343085	-0.37221306	-3.317441286
H	-2.452034685	1.46839865	-4.268423314
C	0.127704668	0.461714596	-4.017927504
H	-0.674251399	3.832704612	-4.24013929
H	0.875368309	3.051320519	-4.651131905
C	-0.194918061	2.880441732	-4.488682191
H	-0.439630702	0.182784088	-4.916161599
H	1.173566108	0.589302938	-4.324635882
H	-0.617364227	2.551710626	-5.448550838
S	-0.610542241	-2.548957281	-1.020730469
O	0.508036683	-3.446855253	-1.309188476
O	-1.488025265	-2.809676771	0.129734708
O	-0.141383906	-1.055049073	-1.045703781
C	-1.731300949	-2.646473925	-2.517275576
F	-2.670383773	-1.685922817	-2.458932502
F	-1.023000485	-2.49525207	-3.640387326
F	-2.321559667	-3.837189715	-2.51817273
N	-2.394738056	0.092709835	0.041427658
N	-3.484341038	-0.112317893	-0.074081377
H	-1.622429529	2.409917649	1.89103253

Supplementary Table 31 | Cartesian coordinate of XXIV. Units are presented in Å.

SCF energy (in toluene) = -2833.09807794 hartree

ZPE = 0.661422 hartree

Atom	Coordinates (Angstroms) X	Y	Z
Mo	-0.309247343	0.541421146	0.380043695
P	0.371213047	-0.83705176	2.501938319
N	1.877811881	1.000307405	0.635794674
P	0.201848281	2.051524522	-1.640906701
N	-0.918300621	1.734522416	1.389615658
H	-1.184236674	-1.606485458	5.979082064
H	0.48803385	-1.147316597	5.63425705
C	-0.393175893	-1.650701909	5.215872865
H	-0.598036849	1.009287036	4.881416609
H	-0.152134069	-2.70691116	5.06204036
H	-2.237802578	0.363414099	5.003939539
C	-1.373264505	0.459845349	4.330922859
H	-2.878281389	-1.836752957	4.173318918
C	-0.908438185	-0.959936326	3.938548635
H	0.883523139	1.388250852	3.268857442
H	-1.677273845	1.058905886	3.466282226
H	2.145886012	0.319027979	3.914815479
C	-2.124939185	-1.730900827	3.378628665
H	1.957553012	-2.557176454	4.649610319
H	-2.585809434	-1.191141282	2.544894216
C	1.546296577	0.547343387	3.026408453
H	4.186923363	1.251298809	3.090173121
H	-1.86867549	-2.733826942	3.022220523
H	3.162535062	-1.618876809	3.741965416
H	0.262463857	3.611682988	0.94467736
C	2.475448194	-2.473121274	3.690957641
H	-1.010747717	5.604503326	-0.994106177
C	3.774326175	1.291011528	2.085417965
H	3.097303908	-3.373082752	3.57565677
C	2.432057471	0.955396815	1.881061521
H	0.863440466	5.205032309	0.431193415
H	-1.747599378	4.037887751	-0.610095473
C	0.880568839	4.128332451	0.205875247
C	-1.062557417	4.548956298	-1.298204495
H	5.611827978	1.922176664	1.142613968
C	1.525501157	-2.388649392	2.476338231
H	1.918523027	3.79261971	0.313703006
C	4.563804083	1.665574584	1.000779856
H	-1.494088233	4.519958479	-2.304857514

H	0.120143957	-3.861174381	3.326018742
H	3.131409398	-1.48020901	1.269409772
C	0.352627702	3.935503428	-1.233707304
C	0.669802038	-3.671045025	2.396716563
C	2.412894926	-2.306557376	1.212083254
H	2.992992505	-3.237553548	1.138349484
H	1.392414169	5.734691777	-1.824840453
C	3.990474248	1.709445311	-0.268744563
H	-0.043263995	-3.646142376	1.566042121
C	2.641679571	1.380135387	-0.426411779
C	1.319319851	4.692767727	-2.169509328
H	2.333429444	4.273503702	-2.131757909
H	1.339206366	-4.528024113	2.233891307
H	4.575116038	1.999895073	-1.137741404
H	1.838706032	-2.21190403	0.288693342
H	-2.518151129	2.51493695	-2.733753866
C	1.9796032	1.432426127	-1.777217291
H	0.996794259	4.717612835	-3.211682404
H	2.597572105	1.990555592	-2.490656902
H	-2.305324239	0.754570296	-2.652308251
C	-2.061588237	1.650265505	-3.230299666
H	1.87662833	0.405674438	-2.153801473
C	-0.53404074	1.802771809	-3.411839018
H	-0.016989745	-0.336497613	-3.267778098
H	-2.530849835	1.548164263	-4.219701041
C	0.026800016	0.484881025	-3.985342286
H	-0.713004376	3.872489712	-4.182211911
H	0.820106935	3.069924598	-4.606982035
C	-0.252890619	2.913096303	-4.442309947
H	-0.573092499	0.197381831	-4.860323689
H	1.064428178	0.599232668	-4.326267788
H	-0.684261285	2.600349429	-5.404764395
S	-0.390202435	-2.767070801	-1.258427365
O	0.708991278	-3.723189607	-1.463900115
O	-1.383991824	-3.071225987	-0.207835819
O	0.080389284	-1.318512526	-1.229624614
C	-1.382577425	-2.83937384	-2.838979976
F	-2.322066615	-1.871435498	-2.857608415
F	-0.588254357	-2.670654778	-3.911736846
F	-1.99253343	-4.024963771	-2.94367346
N	-2.28340505	-0.122304973	-0.047009194
N	-3.342578068	-0.429361928	-0.240102938

Supplementary Table 32 | Cartesian coordinate of the reactant complex for protonation of 1 by lutidinium. Units are presented in Å.

SCF energy (in toluene) = -4289.81340546 hartree

ZPE = 1.442197 hartree

Atom	X	Y	Z
Mo	3.00651646	0.426313984	-0.109637676
Mo	-1.970290333	-1.092783803	0.614780896
P	-2.387603348	-2.562062225	-1.432904872
P	2.843615817	2.968193782	-0.107032579
N	5.156738659	1.051406235	-0.396855324
N	-4.040947467	-1.861543037	1.011212457
P	-2.499914572	-0.03231846	2.873953907
P	4.229284241	-1.825875227	-0.236404882
N	1.066447593	-0.128304261	0.143991107
N	-0.03401148	-0.445874064	0.289112553
N	2.547333806	0.419440637	-3.245052665
N	2.716500385	0.431309469	-2.12128003
N	3.253162218	0.405044462	1.910678403
N	3.379089323	0.407618222	3.039418561
N	-3.268201364	1.291353849	-0.935451595
N	-2.755564482	0.424256168	-0.374770992
N	-1.195693387	-2.710888444	1.647014803
N	-0.784077955	-3.610903794	2.19402028
H	-2.584843671	-2.007244753	-5.28751913
H	1.099779417	5.688998808	-2.290150321
H	2.689496063	4.970980927	-2.556124564
H	-3.910825546	-1.917265579	-4.12321335
C	-2.936123393	-2.384961683	-4.315600823
C	1.951237441	5.211301342	-1.781480179
H	1.584592614	2.71931118	-2.882130294
H	-2.688231172	0.029435763	-3.031501913
H	2.38911852	5.955790974	-1.11173771
H	-3.09362852	-3.460802853	-4.423364973
H	0.029026668	3.514474777	-2.623586795
H	-1.394371444	-0.165059799	-4.226651936
C	-1.742335581	-0.485552701	-3.233537744
C	0.857323145	2.998043081	-2.113516256
H	-0.169287728	-2.154041982	-4.53210471
H	-0.53274448	4.710662971	-0.668911351
C	-1.897942631	-2.01888756	-3.234122975
C	1.451318142	3.94557307	-1.053029355
H	3.941471216	2.954848806	-2.251432174
H	-4.362267499	-1.259190936	-1.838042533

H	-1.011369988	-0.150058601	-2.490908092
H	0.464972964	2.078154959	-1.66635647
H	-4.816128944	-2.950525226	-2.067425573
H	4.680625277	4.243967236	-1.30047163
C	-0.523362923	-2.615535293	-3.599073056
C	0.312103954	4.316076629	-0.083247074
H	-2.126763048	-5.096605233	-3.454450243
H	2.453497418	5.983919917	1.037025228
H	0.228100317	-2.407560238	-2.826910601
H	-0.049518408	3.442394171	0.474206132
C	-4.253855312	-2.268681565	-1.416761363
C	4.322216305	3.2082866	-1.251588787
H	-6.719029838	-3.104596063	-0.648477128
H	6.982158192	3.67708545	-1.510762976
H	-0.560688549	-3.69666846	-3.76567699
H	0.595277691	5.092890802	0.633584932
H	-3.790092117	-4.992391557	-2.831591362
H	4.108823506	5.780308966	0.419958169
H	4.998960631	-0.520216805	-2.804989808
H	-4.730569416	0.922632401	1.149394156
C	-2.755719674	-5.25243756	-2.572219043
C	3.424668852	5.520082499	1.237895665
H	3.850901262	-3.257568228	-3.773157719
H	-3.535985643	3.595087428	2.244686294
C	-6.126218147	-2.766140359	0.198256047
C	6.784460264	2.690635457	-1.097984167
H	-2.74155204	-6.33087759	-2.355429887
H	3.821576461	5.989141869	2.150462898
C	-4.829360947	-2.29534947	-0.019680628
C	5.462812649	2.282369146	-0.904987416
H	5.757945836	-1.81794245	-3.735278396
H	-5.49261528	2.210885324	2.10051997
H	2.860206226	-2.075798126	-2.905155414
H	-2.578090187	2.468076072	1.254940076
C	5.558460186	-1.454601541	-2.71655203
C	-4.919006191	1.273594675	2.166686793
C	3.533559747	-2.927462011	-2.7726645
C	-2.87199223	2.716345102	2.279277188
H	8.86655095	2.14176083	-0.891505516
H	-7.644250438	-3.187814838	1.68135316
C	-2.249348804	-4.494624683	-1.327964184
C	3.332531237	3.997114304	1.46550925
H	6.528333782	-1.23480788	-2.255829367
H	-5.560116084	0.541193221	2.670225277
C	7.831308187	1.837395556	-0.753919229
C	-6.639684172	-2.815031084	1.493739816
H	2.966408177	-3.748307706	-2.32336073
H	-1.972621796	3.014103132	2.826706606
H	-0.120680035	-4.624837001	-1.903557302
H	1.311349276	4.074775393	2.356517801
H	-4.177120694	-4.877909087	-0.330082215
H	5.521470493	3.837409153	1.24960319

C	4.785065611	-2.551173248	-1.948711556
C	-3.620708082	1.548521142	2.958288451
C	-0.777879569	-4.885433733	-1.06849278
C	2.316767308	3.713886995	2.594422758
C	-3.103342562	-4.975024661	-0.132161381
C	4.72837925	3.530794416	1.941084613
H	-2.898827523	-6.041861754	0.038388883
H	4.940808904	4.004629726	2.910443235
H	6.063666745	-4.03500165	-2.865158277
H	-4.749139914	2.817787546	4.298797686
C	-5.833222414	-2.378312428	2.543110495
C	7.519089355	0.585342837	-0.22853795
H	-0.380184952	-4.416796383	-0.164790478
H	2.251369151	2.648026979	2.828946488
C	-4.545827187	-1.902532528	2.281788844
C	6.182613679	0.211681129	-0.066263061
C	5.716847354	-3.77617967	-1.853717282
C	-4.044802521	1.975870269	4.377877847
H	6.609978032	-3.576419194	-1.247949162
H	-4.563915625	1.173382357	4.917249106
H	-0.716504903	-5.975068949	-0.930408205
H	2.642786252	4.234157354	3.507376491
H	8.30414996	-0.111827654	0.054380886
H	-6.193798818	-2.401099245	3.568785107
H	-2.877938866	-4.443862327	0.795376687
H	4.794881489	2.449418434	2.082297596
H	3.337528564	-4.864697997	-0.529216271
H	-1.133526415	2.229360679	4.644104665
C	5.8356822	-1.1498964	0.482759005
C	-3.67144575	-1.413907449	3.411337687
H	5.217117781	-4.661855632	-1.449406882
H	-3.204576597	2.3155291	4.990751326
H	6.687694093	-1.830824188	0.36481045
H	-4.289611721	-1.155267585	4.28005558
H	1.991896935	-3.775804489	-0.121152592
H	-0.067857446	1.600270035	3.363687254
C	2.872847552	-4.291918482	0.28002079
C	-0.501551482	1.385279008	4.348791147
H	5.640892875	-1.06652923	1.561608009
H	-3.004966258	-2.22848087	3.730954481
C	3.849643725	-3.311366696	0.958721274
C	-1.250163755	0.035886067	4.359195833
H	2.234596631	-2.183638102	1.934637669
H	0.308308956	-0.959287293	3.169726153
H	2.516875644	-5.015490563	1.027630296
H	0.329222409	1.335446498	5.066714246
C	3.139197573	-2.74015698	2.20118184
C	-0.194542785	-1.065384043	4.136187157
H	5.668992549	-4.539400582	0.63492376
H	-2.631944701	0.54591419	6.021326869
H	5.782402264	-3.423191001	2.00918403
H	-2.362310711	-1.194401699	5.80934469

C	5.102178438	-4.071679578	1.443939419
C	-1.889720772	-0.206376815	5.743247071
H	2.846270733	-3.574816549	2.854834407
H	0.568720688	-0.983299057	4.923130106
H	3.785325417	-2.079590978	2.788226746
H	-0.618035041	-2.072957986	4.201050797
H	4.784797427	-4.873079956	2.127481799
H	-1.097549429	-0.180147999	6.505912546
H	-4.381845322	2.466847176	-1.845213731
C	-6.777405615	3.515194032	-3.8522425
C	-6.642772516	4.893061987	-3.634472155
C	-5.663242663	5.363896191	-2.750449513
C	-4.831635732	4.467775383	-2.093898617
N	-4.99771554	3.132599594	-2.344155611
C	-5.939712526	2.624818729	-3.197224646
H	-7.530995283	3.127801894	-4.531988951
H	-7.296673841	5.593152065	-4.148222522
H	-5.541444035	6.42746318	-2.566168069
C	-3.761456242	4.85107529	-1.119401824
H	-2.791397048	4.423964253	-1.408817964
H	-3.664878791	5.939158854	-1.062449278
H	-3.98819719	4.468401571	-0.114502612
C	-5.985625696	1.13567696	-3.35729762
H	-5.065037415	0.761121529	-3.826945045
H	-6.079806797	0.638691792	-2.381525451
H	-6.833856411	0.842427487	-3.982724651

Supplementary Table 33 | Cartesian coordinate of the transition state for protonation of a terminal dinitrogen ligand in 1 by lutidinium. Units are presented in Å.

SCF energy (in toluene) = -4289.79497531 hartree

ZPE = 1.437074 hartree

imaginary frequency: 1033*i*

Atom	X	Y	Z
Mo	3.064380177	0.378827923	-0.091650051
Mo	-2.009976507	-0.940106368	0.382749833
P	-2.342611027	-2.366704275	-1.726725403
P	2.930735153	2.913517979	-0.320532506
N	5.246651898	0.969717769	-0.307424952
N	-4.051660231	-1.806081108	0.727119494
P	-2.589942961	0.017618685	2.676546675
P	4.277834618	-1.884571907	0.012855615
N	1.119338427	-0.100354354	0.089298971
N	-0.00494368	-0.34980282	0.175784782
N	2.748330246	0.094915286	-3.233060783
N	2.872746988	0.20900963	-2.109717063
N	3.193539952	0.515220618	1.936210238
N	3.244633693	0.594714386	3.068199453
N	-2.95370604	1.593272217	-1.096683767
N	-2.665308657	0.591921438	-0.528363392
N	-1.169202828	-2.62480041	1.383242245
N	-0.70591427	-3.512035024	1.895041779
H	-2.529687847	-1.678223533	-5.554671811
H	1.361738729	5.461643288	-2.816596873
H	2.975093896	4.754807444	-2.896696636
H	-3.86576525	-1.668543872	-4.402169935
C	-2.878035396	-2.10365028	-4.601988333
C	2.170921597	5.034251282	-2.205176405
H	1.897951778	2.480650808	-3.177456152
H	-2.711977995	0.267817312	-3.224032883
H	2.54173242	5.830373332	-1.555034908
H	-3.003483759	-3.178310402	-4.753569375
H	0.326649701	3.280911657	-3.084647821
H	-1.413539408	0.156102143	-4.420140839
C	-1.751343566	-0.211611922	-3.440220593
C	1.113308782	2.799824397	-2.48409636
H	-0.117108857	-1.781528746	-4.788114872
H	-0.391954959	4.580302558	-1.260513359
C	-1.85665578	-1.747208878	-3.50100051
C	1.62039785	3.812734314	-1.438182304

H	4.175809809	2.733349984	-2.37585097
H	-4.357733046	-1.122221103	-2.095600885
H	-1.032896226	0.119571602	-2.684175846
H	0.685817701	1.907166036	-2.016199226
H	-4.75163748	-2.817271507	-2.397634576
H	4.857394883	4.087446443	-1.472187937
C	-0.461703359	-2.28849476	-3.875844452
C	0.405210384	4.23330398	-0.586768864
H	-2.003693524	-4.817543482	-3.826618901
H	2.468807073	6.00167041	0.558711279
H	0.279113855	-2.084309642	-3.092443905
H	0.003916393	3.391210106	-0.008179762
C	-4.217515558	-2.144003994	-1.71520709
C	4.487869902	3.059500265	-1.372500248
H	-6.653370076	-3.105814772	-1.006343621
H	7.158795727	3.498798991	-1.495470031
H	-0.464671436	-3.363234708	-4.083331571
H	0.627846308	5.056682135	0.099672726
H	-3.671319139	-4.762558723	-3.211142741
H	4.169462534	5.767670935	0.097146182
H	5.174655833	-0.750491523	-2.590060728
H	-4.713361548	0.960906662	0.895820244
C	-2.634738119	-5.016772541	-2.95514834
C	3.420390983	5.560948945	0.872509045
H	4.061152669	-3.580460918	-3.417963589
H	-3.682046689	3.638035714	2.139406912
C	-6.085223708	-2.769824582	-0.142130294
C	6.928621405	2.545405475	-1.025844742
H	-2.604417352	-6.101973362	-2.778113011
H	3.740575729	6.096399989	1.778664682
C	-4.804470046	-2.243642762	-0.328506652
C	5.593901745	2.158372231	-0.88326434
H	5.946643007	-2.11207544	-3.41266029
H	-5.569295628	2.218058766	1.813924743
H	3.033686108	-2.332259479	-2.700742379
H	-2.630082128	2.579907364	1.171098317
C	5.717902318	-1.684105274	-2.425728832
C	-4.984201364	1.290782286	1.902287977
C	3.697104732	-3.168502953	-2.465059967
C	-2.991400415	2.781574652	2.184343139
H	8.989720202	2.000251937	-0.661725932
H	-7.605500874	-3.290893952	1.305911003
C	-2.146309186	-4.296272966	-1.681493261
C	3.3088442	4.058395288	1.200829854
H	6.674833671	-1.445743703	-1.947139327
H	-5.641190632	0.534319654	2.347178095
C	7.945271157	1.712471791	-0.563457027
C	-6.613744625	-2.874191883	1.144044228
H	3.106642272	-3.94996027	-1.977193429
H	-2.137216986	3.087432089	2.795032143
H	-0.012052785	-4.340864869	-2.257214373
H	1.232106254	4.210135179	1.941493904

H	-4.061403468	-4.790135825	-0.707560923
H	5.505786624	3.873196031	1.148886426
C	4.910131418	-2.725491266	-1.616427023
C	-3.741574493	1.567477533	2.776713542
C	-0.664295944	-4.660258914	-1.439322524
C	2.213150612	3.854627222	2.271012404
C	-2.985675321	-4.849229768	-0.506685698
C	4.664497597	3.628726458	1.807314533
H	-2.739455961	-5.911989739	-0.369593268
H	4.816646107	4.181045063	2.746233296
H	6.225682167	-4.263530379	-2.37536123
H	-4.983576702	2.735537103	4.106607148
C	-5.841150441	-2.436315255	2.21753731
C	7.590872591	0.501701214	0.027221506
H	-0.278133561	-4.228024886	-0.512540433
H	2.116968189	2.805086595	2.561465347
C	-4.570017409	-1.901952157	1.988685844
C	6.242959398	0.149866599	0.13789888
C	5.836423067	-3.93874085	-1.399029779
C	-4.24753709	1.921746193	4.188592407
H	6.704675055	-3.698431043	-0.772383067
H	-4.753239179	1.079724618	4.678107239
H	-0.57506591	-5.753286607	-1.355735381
H	2.482821719	4.425344465	3.172039202
H	8.35095966	-0.179627396	0.401992562
H	-6.215783734	-2.501407848	3.236207053
H	-2.78822492	-4.341330295	0.440379299
H	4.709577647	2.562864025	2.042976654
H	3.36796547	-4.930627962	-0.101566564
H	-1.31606629	2.242514385	4.557945945
C	5.852830963	-1.165370139	0.762816928
C	-3.73525654	-1.412259089	3.147291735
H	5.318840761	-4.796766133	-0.958733619
H	-3.450453312	2.276865909	4.848615596
H	6.703038885	-1.858261873	0.740138215
H	-4.381054492	-1.194622352	4.007251863
H	2.022544818	-3.805202202	0.192420261
H	-0.209102768	1.687971667	3.277382387
C	2.886001591	-4.301046471	0.653760259
C	-0.657595212	1.425653219	4.244019622
H	5.604281371	-1.001266273	1.821976886
H	-3.052447858	-2.213636989	3.466855043
C	3.852759218	-3.282140329	1.29137024
C	-1.377968339	0.062084414	4.192181915
H	2.218238871	-2.084359262	2.141539349
H	0.2193893	-0.876952404	3.006988204
H	2.502200997	-4.969924332	1.437560683
H	0.159264402	1.367877649	4.976928666
C	3.114036746	-2.620015347	2.471053391
C	-0.299783186	-1.015251676	3.960813994
H	5.669344115	-4.55068977	1.120740129
H	-2.811002633	0.497911589	5.830309881

H	5.746688468	-3.341400789	2.416626396
H	-2.499606591	-1.230954325	5.582169836
C	5.079102317	-4.019929286	1.871682396
C	-2.046810215	-0.232006172	5.552155682
H	2.805420629	-3.40347663	3.178753941
H	0.447886292	-0.943363143	4.763309763
H	3.747557899	-1.916615515	3.02146906
H	-0.709810719	-2.03070838	3.994839377
H	4.72672031	-4.768067774	2.597438723
H	-1.274192591	-0.209551346	6.334617108
H	-4.03357622	2.131847686	-1.302594369
C	-7.432950301	3.290432513	-2.060170629
C	-7.226023318	4.669557844	-2.059902323
C	-5.944238822	5.166318973	-1.832060735
C	-4.893036288	4.274824166	-1.598401018
N	-5.11888892	2.939861009	-1.595060973
C	-6.352619017	2.436650319	-1.827654011
H	-8.418679078	2.871315746	-2.244338091
H	-8.0556173	5.349891176	-2.241398684
H	-5.748416996	6.235377307	-1.8356395
C	-3.487798943	4.748377615	-1.34277426
H	-2.771170996	4.218124191	-1.980475421
H	-3.403362779	5.82392422	-1.527849784
H	-3.192148001	4.552132218	-0.303875614
C	-6.50290552	0.93989519	-1.848273016
H	-5.923927339	0.506629649	-2.675013962
H	-6.142219179	0.487505624	-0.916369065
H	-7.5511875	0.655236569	-1.983823039

Supplementary Table 34 | Cartesian coordinate of the product complex for protonation of a terminal dinitrogen ligand in 1 by lutidinium. Units are presented in Å.

SCF energy (in toluene) = -4289.80099118 hartree

ZPE = 1.440119 hartree

Atom	Coordinates (Angstroms) X	Y	Z
Mo	3.100398543	0.180335551	-0.078324009
Mo	-2.017931589	-0.787138268	0.411176457
P	-2.476997113	-2.191986341	-1.698619099
P	3.022877386	2.708983878	-0.479872388
N	5.296438377	0.694925753	-0.381901787
N	-4.073999507	-1.607397128	0.843217929
P	-2.509366549	0.232182789	2.703238721
P	4.249569909	-2.112657042	0.188766238
N	1.157545909	-0.207552284	0.13477316
N	0.016221037	-0.387885059	0.216995385
N	2.764000568	-0.312147213	-3.193520629
N	2.892679153	-0.126229383	-2.081511285
N	3.278933182	0.46603051	1.930821032
N	3.37064321	0.630353543	3.050212044
N	-2.772858447	1.819824859	-1.008105127
N	-2.586752431	0.752033523	-0.457106901
N	-1.237964232	-2.551338734	1.441499423
N	-0.786334857	-3.434889153	1.96169808
H	-2.84838236	-1.507721776	-5.508514194
H	1.44877867	5.107487338	-3.108021378
H	3.066346458	4.405412616	-3.140906038
H	-4.129905321	-1.52005167	-4.297000498
C	-3.14696933	-1.941437873	-4.542912357
C	2.257967791	4.721855339	-2.470218069
H	1.968384475	2.11640159	-3.293105404
H	-2.947474241	0.433479915	-3.179672669
H	2.622130048	5.557470654	-1.867724482
H	-3.263402103	-3.016986753	-4.693446047
H	0.399431895	2.927589414	-3.234307227
H	-1.707845186	0.333149829	-4.434489993
C	-1.99172259	-0.034974922	-3.437854234
C	1.189361106	2.478977951	-2.614457887
H	-0.410586764	-1.599486766	-4.865601715
H	-0.306765489	4.321233185	-1.470873614
C	-2.080010594	-1.571492811	-3.490322832
C	1.707160405	3.547786249	-1.632596378
H	4.235532814	2.380278602	-2.536550409
H	-4.505682069	-0.926143751	-1.958855809

H	-1.242390151	0.31017947	-2.719967762
H	0.760226381	1.619321529	-2.090719723
H	-4.914215041	-2.619942901	-2.246195706
H	4.968767583	3.753968689	-1.709328373
C	-0.697148145	-2.097055351	-3.928559111
C	0.503356729	4.019778042	-0.791651081
H	-2.195585096	-4.640253824	-3.811556493
H	2.616756658	5.849692752	0.230903969
H	0.080655526	-1.867363239	-3.189632098
H	0.108300935	3.216026844	-0.157729852
C	-4.344944737	-1.949720487	-1.590082078
C	4.570175047	2.743783223	-1.553846913
H	-6.759927306	-2.876742035	-0.774704533
H	7.247530526	3.085454574	-1.77194024
H	-0.690903869	-3.174801758	-4.120023366
H	0.738733047	4.88344601	-0.160929026
H	-3.846279438	-4.600192742	-3.151088374
H	4.299436657	5.559379769	-0.264980557
H	5.11897928	-1.239498953	-2.49388697
H	-4.654457089	1.184792942	0.942781412
C	-2.801016951	-4.845196768	-2.923644818
C	3.570646348	5.412163991	0.542118653
H	3.833764819	-4.074800343	-3.072561514
H	-3.553199406	3.873723642	2.166951828
C	-6.151441938	-2.543678639	0.062327887
C	7.001475384	2.168997289	-1.241065199
H	-2.756203194	-5.930052587	-2.747809982
H	3.92539204	5.993038894	1.406372601
C	-4.869878015	-2.041101881	-0.178858208
C	5.659890849	1.835906567	-1.037045156
H	5.782655585	-2.693373664	-3.2507762
H	-5.459904486	2.473660376	1.85235753
H	2.899529595	-2.716582325	-2.429861772
H	-2.560711928	2.81029289	1.15213601
C	5.6191054	-2.188162445	-2.287983377
C	-4.894102199	1.536237335	1.949990515
C	3.526686321	-3.566262814	-2.146742307
C	-2.876380092	3.005912297	2.181045823
H	9.054004513	1.574387448	-0.911477695
H	-7.623058106	-3.016407337	1.572849871
C	-2.285496874	-4.12123348	-1.662530422
C	3.444421917	3.933925283	0.961607127
H	6.606314106	-1.972390486	-1.862998025
H	-5.558826797	0.803791634	2.423558842
C	8.004651919	1.328696519	-0.76365948
C	-6.630622598	-2.620180016	1.36850817
H	2.913268581	-4.269852925	-1.575408454
H	-1.995411633	3.293621537	2.761789332
H	-0.174846856	-4.175377551	-2.310574821
H	1.382538021	4.159395246	1.719363304
H	-4.178331407	-4.621652554	-0.651984294
H	5.637143452	3.72146901	0.880780695

C	4.790222167	-3.121690392	-1.376712231
C	-3.630094727	1.80516401	2.797200962
C	-0.79447877	-4.474943654	-1.460097311
C	2.363541947	3.813605291	2.058743208
C	-3.098698507	-4.679361007	-0.471787401
C	4.804762127	3.514404123	1.563478078
H	-2.848789987	-5.7422406	-0.343241375
H	4.9801881	4.101539108	2.476596054
H	6.0057595	-4.777215674	-2.046509841
H	-4.830344334	2.998698672	4.138938731
C	-5.81565972	-2.176866692	2.407188158
C	7.632116388	0.167057843	-0.091107508
H	-0.373865372	-4.011680317	-0.56307875
H	2.258267855	2.787327788	2.41991709
C	-4.545309757	-1.666702402	2.123304188
C	6.278003175	-0.129641083	0.085002214
C	5.664112036	-4.359140063	-1.088021964
C	-4.098177429	2.181491045	4.216801982
H	6.563094198	-4.11385933	-0.508413815
H	-4.597168108	1.352398923	4.734840502
H	-0.696464446	-5.564705403	-1.349465462
H	2.653948066	4.439472629	2.915564998
H	8.382439238	-0.515126568	0.301205917
H	-6.157094468	-2.215646703	3.43856288
H	-2.88698745	-4.176721364	0.474801141
H	4.840180992	2.457878482	1.839311996
H	3.226855625	-5.115123382	0.381380119
H	-1.0895688	2.460771057	4.465425738
C	5.867595234	-1.381248886	0.820952168
C	-3.66692433	-1.162690116	3.242843438
H	5.122785387	-5.15553067	-0.567495365
H	-3.28086209	2.543482653	4.848193992
H	6.695287532	-2.101031291	0.825352819
H	-4.285300316	-0.902934045	4.11064283
H	1.928776258	-3.925831247	0.625899641
H	-0.069132954	1.833290302	3.147505783
C	2.796477396	-4.406472006	1.095731154
C	-0.473879611	1.614286861	4.14390129
H	5.663589017	-1.124906457	1.871288
H	-2.996360672	-1.970218746	3.57230553
C	3.821357618	-3.372215131	1.603567887
C	-1.242379723	0.276949258	4.169626821
H	2.265731788	-2.04502193	2.410628119
H	0.270925672	-0.758243022	2.957386988
H	2.426357319	-4.991130187	1.950068979
H	0.377329426	1.549341169	4.83601336
C	3.15504684	-2.585756671	2.749153537
C	-0.21594508	-0.848512795	3.933461554
H	5.577461986	-4.725941331	1.476620099
H	-2.589220858	0.823925821	5.845505492
H	5.765242439	-3.402221744	2.643218667
H	-2.355449816	-0.923875516	5.64744746

C	5.046214919	-4.101263543	2.198499779
C	-1.866399024	0.054770661	5.563717391
H	2.847186696	-3.295750085	3.53097441
H	0.564053739	-0.782133184	4.704856777
H	3.834667942	-1.865627727	3.216032252
H	-0.663188271	-1.845710312	4.015860954
H	4.701471388	-4.76119452	3.008370671
H	-1.062171315	0.072847783	6.313728486
H	-3.769238433	2.13087495	-1.178245935
C	-7.635343464	3.499691381	-2.381594502
C	-7.40211243	4.865348124	-2.536514309
C	-6.129092198	5.366166384	-2.272336599
C	-5.121694538	4.485812317	-1.856621954
N	-5.353945603	3.164590766	-1.708871921
C	-6.582921317	2.675088672	-1.967416022
H	-8.615571127	3.072553814	-2.580360648
H	-8.201703214	5.529437383	-2.859664352
H	-5.910350685	6.425523725	-2.3839598
C	-3.730215763	4.978918327	-1.546574235
H	-2.978043115	4.404532544	-2.101068008
H	-3.620159464	6.039581373	-1.797084507
H	-3.501928759	4.852296437	-0.479597566
C	-6.772672566	1.187046304	-1.807360003
H	-6.199821968	0.642692199	-2.571651056
H	-6.418078194	0.850377771	-0.824809806
H	-7.825766732	0.903096105	-1.911693258

Supplementary Table 35 | Cartesian coordinate of the transition state for elimination of a terminal dinitrogen ligand trans to the NNH group in II. Units are presented in Å.

SCF energy (in toluene) = -3962.99773733 hartree

ZPE = 1.295192 hartree

imaginary frequency: 70*i*

Atom	Coordinates (Angstroms) X	Y	Z
Mo	-2.926412567	1.815830106	1.787003454
Mo	2.236463706	1.832097899	1.71627339
P	2.690803741	-0.589994917	2.410247608
P	-3.161018178	3.408472139	3.793269838
N	-5.181803854	1.875816967	2.124578634
N	4.382039313	1.559029539	1.056095458
P	2.746308082	4.11587768	0.703325688
P	-3.825764201	0.241503198	-0.061295426
N	-0.964337859	1.792003761	1.591458566
N	0.203038537	1.776251132	1.540915579
N	-2.716671521	-0.642211858	3.776352354
N	-2.790828278	0.241504279	3.069525937
N	-3.053977579	3.383286141	0.490194413
N	-3.121486253	4.256022353	-0.230956986
N	2.434787222	2.893329603	4.544131472
N	2.493132798	2.430446612	3.390261479
N	1.760969851	0.930815442	-1.279456563
N	1.338290282	0.572623766	-2.239194878
H	2.807566652	-2.713517564	5.659081574
H	-1.927207529	3.442035906	7.475443755
H	-3.453922369	2.796628983	6.870380005
H	4.16792484	-1.9429596	4.843617813
C	3.174247843	-2.378163161	4.678118477
C	-2.674828517	3.544272743	6.675377296
H	-2.110515439	1.118251075	5.534182993
H	3.037458373	0.32802041	5.266981546
H	-3.120764755	4.537014745	6.774125927
H	3.288512374	-3.267224612	4.054467597
H	-0.635191224	1.852638279	6.174481825
H	1.785386396	-0.599700679	6.099388134
C	2.080575698	-0.187334148	5.123331594
C	-1.362043127	1.896749977	5.351238047
H	0.410825228	-2.23848729	4.992622658
H	-0.045879459	4.108349529	5.894679553
C	2.175034899	-1.345702783	4.112513392
C	-1.97417844	3.310970957	5.319316297

H	-4.38723445	1.679001261	4.93730858
H	4.625339942	0.153342793	3.630816102
H	1.340060322	0.560554943	4.831439295
H	-0.832150488	1.659029639	4.423453637
H	5.113693423	-1.266664883	2.707749439
H	-5.241847981	3.197357482	5.203923126
C	0.771394728	-1.972488846	3.989010864
C	-0.816827411	4.314046604	5.140298087
H	2.290997865	-3.810204757	2.250518017
H	-3.11346862	6.186775459	5.445758578
H	0.045869499	-1.271166714	3.559170826
H	-0.331785003	4.208276537	4.162326204
C	4.543081589	-0.331641011	2.646708467
C	-4.730703471	2.598076697	4.440796199
H	7.077808218	-0.3812268	1.702557733
H	-7.440740545	2.555853782	4.549894764
H	0.764822331	-2.890135816	3.391471958
H	-1.136774665	5.353578997	5.267771104
H	3.99769207	-3.432953664	1.919711112
H	-4.762699493	5.520787539	5.432261716
H	-4.942551817	-0.985151912	2.413783514
H	4.635040992	3.769932566	2.966686137
C	2.979945961	-3.390885181	1.511607199
C	-3.998725724	6.021431361	4.823728437
H	-3.440498611	-3.405569284	1.060813238
H	3.311144568	6.519009629	3.623806765
C	6.491636637	0.408325841	1.23913122
C	-7.072400409	2.270512371	3.567543288
H	2.957806709	-4.056717776	0.63608838
H	-4.397055498	7.011900111	4.558601511
C	5.154188068	0.574872048	1.607838606
C	-5.694744947	2.235400248	3.336816147
H	-5.527158013	-2.565860511	1.883585819
H	5.369691026	5.373912638	3.157235211
H	-2.600914796	-1.956044848	1.627326414
H	2.375490896	5.013921966	3.552576953
C	-5.337105261	-1.533886899	1.555339754
C	4.903945913	4.688250363	2.435091241
C	-3.117331973	-2.395333153	0.769513415
C	2.744630291	5.853331221	2.955484378
H	-9.030691883	1.972568079	2.701373469
H	8.096562	1.130813807	-0.014145419
C	2.586977033	-1.97093372	1.058443179
C	-3.683316503	5.253148312	3.524066742
H	-6.304909879	-1.098309265	1.282113434
H	5.666752266	4.44220348	1.686389845
C	-7.955262709	1.94546753	2.540178532
C	7.057520885	1.250810838	0.285197905
H	-2.396500199	-2.506987659	-0.045968193
H	1.884440249	6.421492572	2.589979025
H	0.428992815	-2.365049141	1.276944789
H	-1.642133875	6.076242925	3.33804161

H	4.596785055	-1.738135855	0.190304548
H	-5.834585952	4.887724132	3.201791029
C	-4.360599613	-1.57533332	0.356951207
C	3.677852724	5.394399658	1.811644691
C	1.136498374	-1.99399904	0.528037766
C	-2.565385608	5.993386831	2.7573188
C	3.547012788	-1.595330011	-0.092614017
C	-4.964264658	5.274401513	2.659280692
H	3.350210078	-2.253247293	-0.951333941
H	-5.186187393	6.317306189	2.391423671
H	-5.454633794	-3.272031735	-0.413671778
H	4.823525752	7.224926845	1.735369363
C	6.268300579	2.251852108	-0.275503277
C	-7.429306444	1.585329031	1.301501139
H	0.796260564	-0.99886796	0.222000713
H	-2.329844487	5.507521045	1.806765669
C	4.937492097	2.393939633	0.12734709
C	-6.044352736	1.550954485	1.119473645
C	-5.08842343	-2.305607337	-0.789886324
C	4.190674576	6.635717807	1.055712233
H	-5.962412687	-1.750058404	-1.152767963
H	4.805243587	6.382805301	0.18221529
H	1.075320491	-2.661308183	-0.344282132
H	-2.904668194	7.015231017	2.532285737
H	-8.082932199	1.32623445	0.472168349
H	6.675717366	2.931249304	-1.019889416
H	3.430213183	-0.562875813	-0.432488408
H	-4.859189166	4.711120626	1.728670385
H	-2.494718333	-1.812803353	-2.084126373
H	1.087333964	6.826853134	0.70130288
C	-5.465286126	1.153389315	-0.216794973
C	4.086140412	3.48632984	-0.471662946
H	-4.436149532	-2.519845663	-1.642241421
H	3.376641093	7.290939048	0.729803478
H	-6.212336282	0.598691166	-0.797496007
H	4.729088138	4.284668118	-0.861785836
H	-1.314444149	-0.74670785	-1.29150485
H	0.131018536	5.382215775	1.112215606
C	-2.083301867	-0.798543937	-2.073276377
C	0.599545471	5.936807222	0.28924335
H	-5.226688211	2.061884912	-0.789183476
H	3.537274264	3.084627406	-1.335901611
C	-3.165396187	0.284248201	-1.884577624
C	1.571112622	5.052504282	-0.518595207
H	-1.704784854	1.848050339	-1.376330431
H	0.217399068	3.31659804	-0.547650645
H	-1.584789376	-0.640493565	-3.040296834
H	-0.204901247	6.285446192	-0.373764463
C	-2.495834012	1.654381907	-2.108374899
C	0.736401241	3.980152514	-1.246627613
H	-4.786161945	-0.835005325	-2.906227884
H	2.841125232	6.743137422	-1.188300207

H	-5.006129208	0.925408721	-2.912224842
H	2.959410004	5.302608296	-2.216882012
C	-4.261431523	0.121616584	-2.95982221
C	2.279203619	5.899834542	-1.596530704
H	-2.047910233	1.667154207	-3.112974891
H	-0.022766411	4.478525123	-1.866123034
H	-3.210139165	2.48298835	-2.063754322
H	1.350486417	3.374084635	-1.922557283
H	-3.791110057	0.181597687	-3.952315153
H	1.516546447	6.314773614	-2.271592143
H	3.381404546	3.084613821	4.919469486

Supplementary Table 36 | Cartesian coordinate of the product complex for elimination of a terminal dinitrogen ligand trans to the NNH group in II. Units are presented in Å.

SCF energy (in toluene) = -3963.00710453 hartree

ZPE = 1.293303 hartree

Atom	X	Y	Z
Mo	-2.891301886	1.870391597	1.874668552
Mo	2.263664809	1.830721043	1.847080226
P	2.630525452	-0.574090615	2.464391344
P	-3.099531237	3.431187606	3.912849801
N	-5.147550979	1.942451959	2.234405731
N	4.440876927	1.527724934	1.214582499
P	2.721635809	4.008210978	0.668236855
P	-3.825480965	0.340063683	0.001198951
N	-0.92935363	1.831325869	1.678947083
N	0.240913287	1.796831211	1.645469056
N	-2.721753866	-0.61551171	3.830611227
N	-2.773309791	0.277544739	3.133540779
N	-3.036709133	3.458971345	0.606209041
N	-3.135868901	4.34265886	-0.097451143
N	2.483400472	2.918754185	4.661965574
N	2.549078294	2.455395844	3.507343728
N	4.072921928	0.955776187	-3.135101657
N	4.149145115	0.091915234	-3.823969524
H	2.699333943	-2.946515369	5.533220509
H	-1.837901867	3.387928	7.583777961
H	-3.366679037	2.747105199	6.97912682
H	4.086029382	-2.161835998	4.778042667
C	3.0745965	-2.539046386	4.583258777
C	-2.592040391	3.50119102	6.791423661
H	-2.043015832	1.099076832	5.592165787
H	3.085819455	0.143047888	5.335368802
H	-3.040997195	4.490266587	6.910536774
H	3.149107808	-3.373020883	3.879733308
H	-0.562526258	1.814288921	6.242356251
H	1.810051966	-0.766496657	6.152590079
C	2.100935201	-0.318485611	5.191181853
C	-1.292581102	1.878727419	5.423337913
H	0.333682125	-2.290730636	4.989766368
H	0.036757329	4.072415045	5.999364869
C	2.113776883	-1.424204068	4.115586351
C	-1.900382533	3.295110061	5.426439565
H	-4.3296573	1.692697851	5.037045263
H	4.617529385	-0.09786011	3.720280797

H	1.3938939	0.482018198	4.957497212
H	-0.766819552	1.660238053	4.488493405
H	5.001630925	-1.436414536	2.643975319
H	-5.169060398	3.213317086	5.335911387
C	0.685636151	-1.990374655	3.992747628
C	-0.742897699	4.299530632	5.260506959
H	2.237211033	-3.743157225	1.989247495
H	-3.010131315	6.176354713	5.613499744
H	-0.023209158	-1.24791808	3.608182334
H	-0.269815536	4.218270489	4.274916993
C	4.494699596	-0.464082178	2.690434184
C	-4.669717476	2.622550144	4.558527898
H	7.080561766	-0.462763274	1.938322144
H	-7.377137234	2.602438768	4.692367908
H	0.640618739	-2.880284888	3.354582832
H	-1.05905696	5.336079966	5.417951799
H	3.906593967	-3.282982075	1.588303203
H	-4.668320518	5.53322447	5.598583128
H	-4.934249839	-0.929850155	2.458077622
H	4.459792489	4.122998247	3.005066313
C	2.863577455	-3.233009911	1.250095796
C	-3.901151988	6.03393608	4.994064908
H	-3.436064464	-3.32786657	1.045177139
H	2.632589548	6.715045763	3.346544809
C	6.533549479	0.360245283	1.485272678
C	-7.021113739	2.32944244	3.702020224
H	2.796604329	-3.809530935	0.315704334
H	-4.287605921	7.034250393	4.74912296
C	5.17019973	0.508180754	1.75672242
C	-5.646108727	2.286395998	3.457178384
H	-5.521699433	-2.500001201	1.901335127
H	4.927742135	5.829077809	3.144639525
H	-2.599592022	-1.890188067	1.646161234
H	2.078972047	5.032297439	3.432674717
C	-5.332470365	-1.462348815	1.591179489
C	4.625317612	5.044112589	2.4367828
C	-3.115969542	-2.309827921	0.778659008
C	2.27415666	5.864126467	2.748005772
H	-8.990277716	2.061390846	2.850863865
H	8.238381895	1.174227803	0.436686515
C	2.399881662	-1.788744106	0.985652616
C	-3.605482835	5.284990023	3.678607813
H	-6.301261271	-1.021657157	1.329735035
H	5.467686412	4.882925686	1.752315307
C	-7.916736498	2.027799667	2.678732494
C	7.175910084	1.270560057	0.65034891
H	-2.395820315	-2.398507317	-0.040413084
H	1.327015544	6.165996895	2.28772715
H	0.260318911	-2.218344654	1.343908099
H	-1.559241537	6.094939532	3.490398311
H	4.291496412	-1.24943526	-0.028182512
H	-5.763484541	4.947371806	3.375371482

C	-4.361227438	-1.48270762	0.388132559
C	3.348937943	5.5053263	1.698611076
C	0.910357562	-1.787204669	0.576821374
C	-2.486280938	6.026205745	2.913872559
C	3.209878696	-1.233676764	-0.206983997
C	-4.895236445	5.332456862	2.828315332
H	3.013646365	-1.856813664	-1.091370758
H	-5.108694215	6.381388595	2.577878607
H	-5.460423209	-3.164504043	-0.406389303
H	4.173957786	7.496053851	1.529030176
C	6.428488408	2.302253195	0.089309519
C	-7.405639386	1.682506828	1.429751716
H	0.550369547	-0.772170204	0.36648437
H	-2.259090554	5.550103788	1.956160944
C	5.064040758	2.405632175	0.374000675
C	-6.022878998	1.640342904	1.233388207
C	-5.093855617	-2.192822476	-0.768389847
C	3.694777489	6.757102225	0.870472118
H	-5.968051518	-1.630615732	-1.120266823
H	4.397619312	6.548145424	0.053606188
H	0.787586244	-2.387770733	-0.336590042
H	-2.819621984	7.052963113	2.703173047
H	-8.069216569	1.441785897	0.602795497
H	6.889437928	3.025687587	-0.578333748
H	2.922230746	-0.204682526	-0.461564182
H	-4.807544578	4.781572343	1.888653168
H	-2.490527006	-1.661963824	-2.058372699
H	0.65705907	6.339956908	0.17968481
C	-5.461972706	1.261649976	-0.115904552
C	4.242505575	3.469313999	-0.306160884
H	-4.443806456	-2.394754593	-1.625580606
H	2.804385439	7.233909776	0.446777046
H	-6.218215931	0.718931633	-0.696103073
H	4.882402305	4.312068166	-0.595698742
H	-1.310693683	-0.579510198	-1.284443471
H	-0.091331804	4.792716403	0.641808834
C	-2.095513769	-0.641106483	-2.049250799
C	0.37576099	5.344352157	-0.18330696
H	-5.228505963	2.178642269	-0.676802918
H	3.856993526	3.044155038	-1.243313448
C	-3.189913585	0.422829222	-1.830284158
C	1.57784325	4.580004044	-0.771701263
H	-1.741540032	1.995830905	-1.318240015
H	0.507471203	2.658116374	-0.725568009
H	-1.621742002	-0.468818504	-3.02652436
H	-0.38894232	5.484542048	-0.960194562
C	-2.541393955	1.804597596	-2.040490188
C	1.053357963	3.287242444	-1.436342655
H	-4.808028951	-0.699556756	-2.854253945
H	2.613195982	6.398883835	-1.491177959
H	-5.052568553	1.057212086	-2.822274522
H	3.1114525	4.915754394	-2.32647976

C	-4.297524894	0.265024976	-2.894244398
C	2.262365683	5.432325821	-1.860906566
H	-2.107838413	1.838315746	-3.050976724
H	0.362334388	3.556684049	-2.247881304
H	-3.264908185	2.623651112	-1.973337191
H	1.8555347	2.686468328	-1.887448823
H	-3.840244915	0.351835236	-3.890839
H	1.534018451	5.634065925	-2.659911857
H	3.428982681	3.09464447	5.045905097

Supplementary Table 37 | Cartesian coordinate of the reactant complex for protonation of IV. Units are presented in Å.

SCF energy (in toluene) = -5142.20480431 hartree

ZPE = 1.474342 hartree

Atom	X	Y	Z
Mo	3.160798658	0.5710895	-0.068883044
Mo	-1.963312028	-0.560294953	0.076507263
P	-2.198341933	-1.669476812	-2.275643903
P	3.022235276	3.048066629	0.55634873
N	5.336829767	1.263528973	-0.210970985
N	-4.093581278	-1.219439106	0.10648629
P	-2.710712268	0.214835178	2.43146639
P	4.421310451	-1.572545176	-0.771022973
N	1.246300676	0.006998414	0.050784863
N	0.127412926	-0.279426172	0.120242033
N	2.705673903	1.352636034	-3.102656844
N	2.875184465	1.070113866	-2.015160905
N	3.399493931	0.061183487	1.900352707
N	3.510296855	-0.213178895	2.99360162
N	-2.638205396	2.172483193	-1.133249057
N	-2.350757245	1.023811958	-0.636264793
H	-1.792112998	-0.352758089	-5.91135348
H	1.307416226	6.24121724	-0.888308958
H	2.901646429	5.612355094	-1.305177764
H	-3.264740902	-0.369503941	-4.936253726
C	-2.306259183	-0.881215436	-5.095011271
C	2.153476835	5.650533516	-0.504509121
H	1.778009243	3.527342873	-2.20774532
H	-2.074188624	1.206136172	-3.313884366
H	2.580983385	6.203697659	0.335101042
H	-2.515349119	-1.89411936	-5.444893891
H	0.228543807	4.235593762	-1.751224436
H	-0.65603789	1.115433876	-4.377057829
C	-1.151963312	0.650298315	-3.512527735
C	1.0543055	3.601221872	-1.389918023
H	0.476661345	-0.895930665	-4.88397284
H	-0.335193748	4.903783242	0.443268147
C	-1.416085251	-0.831885675	-3.835260689
C	1.646663635	4.24406628	-0.120266892
H	4.106158931	3.552532502	-1.53790197
H	-3.983569452	-0.093671738	-2.563382577
H	-0.491522589	0.760978546	-2.646245957
H	0.666618513	2.595327479	-1.192845872

H	-4.55695091	-1.642773994	-3.201633943
H	4.850276423	4.574034181	-0.304525318
C	-0.044757201	-1.481893349	-4.11370995
C	0.50427433	4.363622448	0.906858124
H	-1.685956675	-3.752680958	-4.711058847
H	2.669502484	5.698738679	2.406202949
H	0.588165078	-1.480603758	-3.217901218
H	0.133096218	3.379305336	1.218911255
C	-4.016065632	-1.175058768	-2.36945814
C	4.493199495	3.556871982	-0.5083257
H	-6.582389965	-2.01775058	-2.03601144
H	7.145897291	4.08683339	-0.676903336
H	-0.119782551	-2.509736503	-4.480112578
H	0.78863867	4.927394532	1.80075271
H	-3.437494851	-3.623811895	-4.423001339
H	4.317177289	5.63528343	1.742110303
H	5.154624738	0.36714793	-2.90761038
H	-4.534652496	1.538967496	0.526828758
C	-2.494322821	-4.019157207	-4.023806813
C	3.636016856	5.189830751	2.478183644
H	4.005315663	-2.074576044	-4.54542304
H	-3.552050231	3.955659275	2.261909715
C	-6.082542964	-1.852206022	-1.08484019
C	6.954768089	3.028984542	-0.513272668
H	-2.577756233	-5.115635245	-4.035346573
H	4.044934062	5.417434615	3.473612777
C	-4.75255508	-1.42653952	-1.071786168
C	5.636170143	2.579850838	-0.406852195
H	5.876122508	-0.633048076	-4.17465625
H	-5.444902093	2.711725903	1.509149257
H	3.026573443	-1.134060616	-3.410783714
H	-2.490802789	2.968499371	1.227708768
C	5.711446836	-0.555327474	-3.090088086
C	-4.919839453	1.744298214	1.529018683
C	3.699295358	-1.991831882	-3.491882394
C	-2.917528759	3.055436214	2.232296356
H	9.039056573	2.453103236	-0.475680342
H	-7.769474726	-2.451178061	0.126186949
C	-2.242335248	-3.578639987	-2.567592266
C	3.524519254	3.659340015	2.329012905
H	6.697453258	-0.462516504	-2.620228624
H	-5.662255784	0.979722299	1.783600279
C	8.005712465	2.121127597	-0.402401204
C	-6.741426074	-2.094471903	0.12068302
H	3.13375642	-2.895441834	-3.244731459
H	-2.103301251	3.212245955	2.943872029
H	-0.0568356	-3.8329326	-2.692611044
H	1.509117244	3.537932934	3.229845544
H	-4.373660274	-3.915014242	-2.158410635
H	5.708704293	3.523404383	2.058225301
C	4.960366236	-1.821232761	-2.616939584
C	-3.782603283	1.822160838	2.570089298

C	-0.900705738	-4.158562261	-2.07376175
C	2.511817893	3.121381198	3.363961389
C	-3.395700768	-4.16689015	-1.729777724
C	4.914262448	3.070019296	2.661759509
H	-3.308460737	-5.261532329	-1.736464558
H	5.141806312	3.286041483	3.715630463
H	6.2377429	-2.998185171	-3.902449198
H	-5.112203476	2.884139899	3.908067754
C	-6.06513837	-1.871173689	1.315447252
C	7.700571411	0.778001552	-0.196492102
H	-0.701515712	-3.875530919	-1.038386217
H	2.435027378	2.031073953	3.337604633
C	-4.741297589	-1.413732328	1.290876109
C	6.365470976	0.37255504	-0.11342345
C	5.901971661	-3.018780295	-2.855072312
C	-4.44343811	2.011185559	3.950766352
H	6.801837028	-2.977130629	-2.228557778
H	-5.057373994	1.149110716	4.239616168
H	-0.942629679	-5.255785439	-2.120505314
H	2.84975941	3.404110454	4.371792297
H	8.488317588	0.034203736	-0.105073748
H	-6.55061274	-2.043240631	2.272540015
H	-3.363793174	-3.867510196	-0.682728685
H	4.957923182	1.986283715	2.531190304
H	3.529309678	-4.422449739	-1.806390534
H	-1.696380371	2.153682557	4.763522591
C	6.02576878	-1.081297835	0.084211668
C	-4.021425585	-1.128867564	2.586921988
H	5.413296166	-3.984654939	-2.69411175
H	-3.720784685	2.196401165	4.749890988
H	6.876678254	-1.70925712	-0.206239853
H	-4.754582048	-0.919978461	3.375857238
H	2.188449762	-3.513229589	-1.06703907
H	-0.416935465	1.811134039	3.570910393
C	3.093505367	-4.096774698	-0.856322333
C	-0.989931652	1.395900424	4.409848725
H	5.840954656	-1.269813645	1.151756398
H	-3.473222543	-2.031892642	2.895735006
C	4.093073532	-3.310280874	0.015345017
C	-1.648885432	0.050310556	4.043112462
H	2.520003131	-2.483545652	1.31180005
H	0.093194063	-0.618981114	2.877132759
H	2.778242522	-4.997737687	-0.314374131
H	-0.277552541	1.215192716	5.227109516
C	3.439187885	-3.071388639	1.389035575
C	-0.508612509	-0.938615273	3.734991816
H	5.893222188	-4.420031159	-0.67040599
H	-3.300040451	0.137590903	5.523887164
H	6.070110899	-3.665840096	0.926237355
H	-2.812269339	-1.511162744	5.058457143
C	5.362510705	-4.159415365	0.248567364
C	-2.450259937	-0.493835924	5.244005404

H	3.180530906	-4.042365614	1.831416948
H	0.153743668	-0.993009203	4.611543221
H	4.113834951	-2.567431231	2.089974004
H	-0.888489834	-1.941521125	3.547176687
H	5.064580903	-5.101573103	0.731566715
H	-1.781392259	-0.547085121	6.115413749
S	-1.79241797	-3.941325311	1.794873097
O	-2.30498577	-3.657102944	3.154805281
O	-2.449828412	-5.021428326	1.047999508
O	-1.548605079	-2.683792364	0.972044005
C	-0.060455894	-4.571484831	2.085649634
F	0.558949296	-4.790871077	0.910822895
F	0.667590649	-3.689933895	2.789376386
F	-0.115484025	-5.72090871	2.762399969
C	-5.962205539	3.366469924	-2.073797646
N	-4.737541612	3.787688511	-1.664839118
C	-4.423653348	5.09812797	-1.493525824
C	-5.400639479	6.063560623	-1.731602019
C	-6.67283374	5.669708747	-2.145711644
C	-6.953786455	4.314293489	-2.320116083
H	-7.935345132	3.982461109	-2.646187753
H	-7.442144297	6.415658452	-2.333279534
H	-5.155349833	7.112502066	-1.591948122
C	-6.17751858	1.893561268	-2.251694269
H	-7.219485351	1.688981677	-2.514857443
H	-5.536879148	1.503930111	-3.053404855
H	-5.934934616	1.34070728	-1.336629899
C	-3.024483771	5.43579507	-1.070142859
H	-2.951166709	6.493055628	-0.798722596
H	-2.713088061	4.82250517	-0.216933062
H	-2.316830645	5.245416606	-1.889428128
H	-3.945581077	3.036097097	-1.43876798
H	-1.769121093	2.656831473	-1.406157877

Supplementary Table 38 | Cartesian coordinate of the transition state for protonation of IV. Units are presented in Å.

```
SCF energy (in toluene) = -5142.20262299 hartree

ZPE =  1.471585 hartree

imaginary frequency: 537i

------------------------------------------------

 Atom            Coordinates (Angstroms)

            X              Y               Z

------------------------------------------------

  Mo   3.154649901     0.500927421    -0.06880634
  Mo  -1.993262882    -0.505088168     0.05806155
   P  -2.231402687    -1.607259229   -2.301379536
   P   3.074149155     2.974492595    0.585762497
   N   5.347696133     1.147857442   -0.205961381
   N  -4.129487925    -1.147410873    0.080999696
   P  -2.737167216     0.268035649    2.420349745
   P   4.372173815    -1.664018333   -0.794665576
   N   1.228160883    -0.012995619    0.044976348
   N    0.10016882    -0.265769931    0.109502562
   N   2.714291868     1.323505284   -3.094176489
   N   2.879467522     1.026308472   -2.010120863
   N   3.382876889    -0.035589288    1.894848032
   N   3.488496245    -0.324977848    2.984541302
   N  -2.583131882      2.26108672   -1.125546694
   N  -2.352913204     1.090030151   -0.641498649
   H   -1.81443358     -0.27748375   -5.930245389
   H   1.427053884     6.220207482   -0.817626088
   H   3.007993694     5.563921501   -1.242497172
   H  -3.288492668    -0.292912862   -4.957646784
   C  -2.331603027    -0.807440184   -5.116844066
   C   2.260614863     5.607495526   -0.441364225
   H   1.841302495     3.514299601   -2.171314012
   H  -2.097076999     1.271484728   -3.325249858
   H   2.699194078     6.141164716    0.405066705
   H  -2.543812063    -1.818083201     -5.4710748
   H   0.307656762     4.250050525   -1.706172578
   H  -0.681398762     1.182520694   -4.390510559
   C  -1.176259478     0.714581461   -3.526938162
   C   1.119020202     3.593320063   -1.352675971
   H   0.451797818    -0.830364126   -4.900134212
   H  -0.241282362     4.903942479    0.500001377
   C  -1.443080752    -0.765663067   -3.855530546
   C   1.724432103     4.207120752   -0.074772012
   H   4.165280678       3.4784205   -1.504812626
   H  -4.014071537    -0.029352485   -2.597188523
```

H	-0.513698869	0.8206412	-2.661675529
H	0.710198485	2.5933257	-1.168440667
H	-4.585768723	-1.58060106	-3.227179131
H	4.933372789	4.468824189	-0.260874361
C	-0.072765545	-1.417974191	-4.133322147
C	0.585293533	4.337532651	0.954653179
H	-1.714546064	-3.680062237	-4.742964482
H	2.782614197	5.609032511	2.46753048
H	0.558119118	-1.421372932	-3.23614494
H	0.191588534	3.357383595	1.252257982
C	-4.046310645	-1.109022328	-2.396510641
C	4.553842406	3.462201613	-0.475924891
H	-6.616909066	-1.933990765	-2.067405466
H	7.216570803	3.936310896	-0.644223087
H	-0.14930674	-2.444197241	-4.503827048
H	0.883114168	4.881068708	1.856606063
H	-3.467594098	-3.552062351	-4.463278285
H	4.427160678	5.517568483	1.798852621
H	5.142686519	0.28389773	-2.908200229
H	-4.539081047	1.626385876	0.521386584
C	-2.526288019	-3.948689487	-4.060758459
C	3.737979238	5.078493945	2.531259306
H	3.939634583	-2.11694926	-4.572705892
H	-3.538768095	4.018979725	2.27149342
C	-6.11841619	-1.770794699	-1.115198597
C	7.002976242	2.881115019	-0.491752552
H	-2.610227922	-5.045078533	-4.07698251
H	4.153836262	5.285386591	3.528295854
C	-4.78592377	-1.353670697	-1.099072921
C	5.675155033	2.459358744	-0.387661918
H	5.83882722	-0.715377884	-4.190139023
H	-5.440871472	2.799925737	1.508259956
H	2.982901944	-1.168475543	-3.426023128
H	-2.47448908	3.028326317	1.244961227
C	5.679344277	-0.647728447	-3.104152272
C	-4.924688301	1.828066158	1.524035242
C	3.637645495	-2.038938445	-3.517672987
C	-2.911419775	3.113952253	2.245181812
H	9.07432516	2.259972553	-0.46458076
H	-7.813573153	-2.35356565	0.091681464
C	-2.281097527	-3.515067588	-2.601263457
C	3.592612889	3.552607931	2.36475172
H	6.668585704	-0.581470081	-2.636595156
H	-5.674582319	1.067962995	1.769695441
C	8.034174259	1.949644545	-0.392831583
C	-6.782807312	-2.004830628	0.088658647
H	3.054243467	-2.933331204	-3.278490887
H	-2.103507754	3.260625151	2.965939567
H	-0.095914022	-3.771860788	-2.713196288
H	1.575936034	3.464203907	3.266884372
H	-4.416155393	-3.844325069	-2.206596412
H	5.772856707	3.371496113	2.088478121

C	4.903585067	-1.903440279	-2.643833438
C	-3.790238132	1.886795615	2.568836273
C	-0.944259262	-4.1005739	-2.10209491
C	2.569843945	3.025524217	3.39546663
C	-3.441845523	-4.101779111	-1.772897197
C	4.969455169	2.928790625	2.688058893
H	-3.359126155	-5.196763471	-1.783418503
H	5.203284496	3.127540108	3.743919877
H	6.154406727	-3.091192599	-3.94504817
H	-5.111527513	2.951637381	3.911971015
C	-6.108885036	-1.783669301	1.285009071
C	7.700396285	0.61136255	-0.200699366
H	-0.751499311	-3.823876592	-1.063733257
H	2.47022118	1.937402206	3.358807379
C	-4.781758076	-1.335787342	1.263886189
C	6.356908583	0.234101305	-0.119618872
C	5.820169985	-3.117112379	-2.89731439
C	-4.453248306	2.070614296	3.94929052
H	6.721844753	-3.100919268	-2.272283239
H	-5.078566856	1.213564563	4.228388839
H	-0.987282263	-5.197402654	-2.156388783
H	2.914823681	3.291308544	4.405495088
H	8.47197381	-0.150229997	-0.118553552
H	-6.598670286	-1.950352595	2.240831231
H	-3.415608272	-3.806768211	-0.72455987
H	4.988954848	1.845893449	2.545059801
H	3.420129138	-4.485434075	-1.855163005
H	-1.709139312	2.178183946	4.770029574
C	5.986502192	-1.213894603	0.063739563
C	-4.064609202	-1.059242161	2.563573838
H	5.312199638	-4.074679905	-2.746677857
H	-3.731341192	2.240407208	4.752556713
H	6.823582434	-1.856732934	-0.234061868
H	-4.799588917	-0.841645245	3.34831545
H	2.100354886	-3.556584838	-1.102613188
H	-0.431163621	1.836732422	3.575563942
C	2.994013949	-4.1602348	-0.900520949
C	-1.008329833	1.418357661	4.410077595
H	5.798994742	-1.409193116	1.129586795
H	-3.530709131	-1.969630568	2.875239927
C	4.012195219	-3.402678158	-0.024896757
C	-1.676751337	0.08107248	4.030595822
H	2.461322875	-2.55758441	1.286400092
H	0.062426906	-0.588571433	2.861403426
H	2.662375939	-5.059924287	-0.366191346
H	-0.298404458	1.224764177	5.226489329
C	3.368983773	-3.164070615	1.353830164
C	-0.542648872	-0.912525773	3.715280384
H	5.787442969	-4.540913936	-0.728817107
H	-3.331302522	0.166516908	5.507395624
H	5.984797952	-3.80770016	0.875201736
H	-2.851355494	-1.481434777	5.030691833

C	5.265184899	-4.279464429	0.194702092
C	-2.484322917	-0.467656182	5.225222998
H	3.093438237	-4.134011594	1.788299319
H	0.118039717	-0.979491532	4.592230837
H	4.056504509	-2.680235274	2.056503386
H	-0.928417639	-1.911112944	3.517784935
H	4.949784014	-5.220515716	0.668623349
H	-1.818116058	-0.530993863	6.097949575
S	-1.878004001	-3.890382961	1.77293125
O	-2.381530445	-3.601293536	3.135030495
O	-2.558370425	-4.954192775	1.023493847
O	-1.611965143	-2.633797547	0.954142281
C	-0.158437882	-4.555206442	2.06003324
F	0.454517694	-4.787266572	0.884336399
F	0.58779623	-3.688276365	2.762655742
F	-0.236177431	-5.703070287	2.736892961
C	-5.826412434	3.474712813	-2.052049203
N	-4.596667712	3.86556426	-1.633678374
C	-4.280333637	5.171114029	-1.440576066
C	-5.246400964	6.153744917	-1.658167683
C	-6.523161333	5.782683631	-2.077515984
C	-6.813641055	4.433801118	-2.278657558
H	-7.797794936	4.11570967	-2.611202916
H	-7.285896546	6.539306476	-2.248774953
H	-4.989386283	7.197228015	-1.498746453
C	-6.063647723	2.009462909	-2.272123016
H	-7.116099763	1.821864596	-2.50544794
H	-5.457413757	1.64348257	-3.111192188
H	-5.7951192	1.42228799	-1.386696617
C	-2.878877743	5.500772722	-1.012064458
H	-2.793758032	6.562696843	-0.762649174
H	-2.582482079	4.905450655	-0.141162445
H	-2.16542076	5.284356927	-1.819616775
H	-3.758130795	3.055913267	-1.397567138
H	-1.699268308	2.720960492	-1.385974066

Supplementary Table 39 | Cartesian coordinate of the product complex for protonation of IV. Units are presented in Å.

SCF energy (in toluene) = -5142.21833182 hartree

ZPE = 1.476087 hartree

Atom	X	Y	Z
Mo	3.153544859	0.543933102	0.004532361
Mo	-1.947133053	-0.500454003	0.248402179
P	-2.291335435	-1.500804232	-2.182243582
P	3.036953631	3.005906401	0.724675169
N	5.326984365	1.247519101	-0.176049063
N	-4.095683236	-1.134646471	0.306183341
P	-2.63973349	0.194279156	2.667982601
P	4.403738242	-1.582339241	-0.812000454
N	1.25317507	-0.008055086	0.167748754
N	0.12874594	-0.284623376	0.250404603
N	2.592813509	1.398319684	-2.996190968
N	2.802865657	1.092178857	-1.923480314
N	3.46880915	-0.029902021	1.943421364
N	3.628095371	-0.348336726	3.017828444
N	-2.454486612	2.380615074	-0.689604239
N	-2.308679873	1.117028803	-0.358134121
H	-2.013380993	0.023732747	-5.741394924
H	1.245215549	6.228246669	-0.537535475
H	2.839219827	5.646088777	-1.022603726
H	-3.456545208	-0.09083338	-4.73052171
C	-2.490983837	-0.566776054	-4.94576411
C	2.110209752	5.635915269	-0.203309456
H	1.741663647	3.57677716	-1.987484826
H	-2.25961399	1.425400268	-3.098439642
H	2.548757261	6.157770676	0.650076282
H	-2.68218802	-1.561686172	-5.350918495
H	0.193735569	4.248074827	-1.479081285
H	-0.847371014	1.413372884	-4.165287305
C	-1.324009793	0.899375323	-3.317901861
C	1.030481993	3.608525696	-1.156042929
H	0.302720719	-0.552921575	-4.807202112
H	-0.356004292	4.783510595	0.764124194
C	-1.565576354	-0.569408	-3.710675601
C	1.633835262	4.205671991	0.129327513
H	4.066627764	3.592543999	-1.376582178
H	-4.091109161	0.101512565	-2.326976112
H	-0.657100687	0.984119287	-2.45413677
H	0.664888102	2.58783589	-0.993383103

H	-4.676627204	-1.424288183	-2.995742498
H	4.838169656	4.559605612	-0.119012395
C	-0.19233506	-1.182754953	-4.054706888
C	0.513228812	4.256925055	1.185728704
H	-1.849122096	-3.455610031	-4.736471161
H	2.715135537	5.575129412	2.681173778
H	0.462235596	-1.218789403	-3.175107438
H	0.182836539	3.251930272	1.478479757
C	-4.104348957	-0.986538494	-2.169199477
C	4.477477135	3.552054256	-0.357215668
H	-6.660690939	-1.812932319	-1.785659996
H	7.126714122	4.088159563	-0.561945636
H	-0.266255736	-2.191854751	-4.4705406
H	0.806561267	4.80008803	2.089154279
H	-3.593401893	-3.321924902	-4.404933826
H	4.35027103	5.552955521	1.980672482
H	5.068286847	0.436702669	-2.893345786
H	-4.49868538	1.656642904	0.861229743
C	-2.647225367	-3.747786415	-4.047604396
C	3.687452162	5.073074951	2.712051315
H	3.881735956	-1.938667414	-4.588620238
H	-3.460121952	3.947787121	2.70548353
C	-6.128398547	-1.695852973	-0.845293787
C	6.938133371	3.024560112	-0.438135395
H	-2.744379988	-4.840978974	-4.114012485
H	4.116179354	5.263427871	3.706818809
C	-4.793091164	-1.287276506	-0.856508661
C	5.62172906	2.571139895	-0.322891694
H	5.763074691	-0.511893594	-4.213911419
H	-5.363857263	2.763962984	1.941739542
H	2.924492919	-1.066108499	-3.383559439
H	-2.400558036	3.031257621	1.622028407
C	5.624711116	-0.474858708	-3.123823531
C	-4.84677854	1.794562811	1.88805848
C	3.60660806	-1.90871919	-3.523900646
C	-2.822329798	3.054257973	2.630557354
H	9.021992237	2.448265328	-0.465918894
H	-7.788479612	-2.30819457	0.393587667
C	-2.358294491	-3.390299344	-2.575388341
C	3.583758185	3.547936826	2.506293282
H	6.620827854	-0.394739345	-2.673821013
H	-5.590106838	1.020548344	2.110340017
C	7.990242646	2.113114767	-0.385405205
C	-6.754550535	-1.970094297	0.369386666
H	3.062639017	-2.83126984	-3.3007662
H	-2.009593381	3.175968982	3.35091455
H	-0.176376942	-3.634219374	-2.760451615
H	1.596498541	3.389145619	3.461887319
H	-4.483254432	-3.725532515	-2.1326821
H	5.763911767	3.444191443	2.182153096
C	4.89149254	-1.760821743	-2.679230305
C	-3.686608952	1.805462744	2.907136008

C	-1.008521306	-4.005542316	-2.150640948
C	2.601648396	2.96579423	3.547072849
C	-3.498482695	-4.011748713	-1.743268296
C	4.986142527	2.956572273	2.780677491
H	-3.425342446	-5.105162323	-1.814280679
H	5.234622747	3.128473968	3.837690197
H	6.138193182	-2.886827736	-4.038106172
H	-4.975982485	2.794410558	4.330517163
C	-6.03955092	-1.800268963	1.550211762
C	7.689541425	0.762851051	-0.226677112
H	-0.785867702	-3.805207815	-1.100878544
H	2.522170548	1.877517886	3.479075258
C	-4.710377065	-1.363273888	1.501748434
C	6.356137718	0.353690771	-0.133261755
C	5.832031234	-2.944020326	-2.983196767
C	-4.315065828	1.915441805	4.312149751
H	6.748388226	-2.916696725	-2.380552803
H	-4.931898956	1.045000236	4.568222248
H	-1.053821677	-5.095732499	-2.28058629
H	2.970411034	3.2063905	4.554939545
H	8.479391004	0.01731606	-0.178742816
H	-6.49973258	-1.99726131	2.514825235
H	-3.438511507	-3.77220545	-0.682089514
H	5.037789889	1.879890623	2.601847883
H	3.500903615	-4.402282412	-1.926012623
H	-1.524906471	1.989245904	5.05946249
C	6.024616231	-1.107980828	0.019848811
C	-3.94618348	-1.148243608	2.785119771
H	5.35292992	-3.917191129	-2.839926251
H	-3.572991821	2.053516652	5.10377013
H	6.872782039	-1.719759299	-0.310716697
H	-4.651517853	-0.974559411	3.606052906
H	2.174853996	-3.508079072	-1.142744751
H	-0.295815774	1.694873747	3.804022446
C	3.079381352	-4.102093792	-0.961151897
C	-0.841151057	1.243406662	4.64241905
H	5.868013209	-1.334133642	1.084860491
H	-3.398910093	-2.070499379	3.02964046
C	4.094935972	-3.346640935	-0.080495314
C	-1.534940789	-0.07178031	4.234823078
H	2.551553251	-2.567620217	1.279202773
H	0.154500287	-0.732761951	2.989343415
H	2.768092047	-5.017917033	-0.442086086
H	-0.101883431	1.012643925	5.42218791
C	3.471374974	-3.157870822	1.314861687
C	-0.427104413	-1.071142912	3.853516426
H	5.885436846	-4.417250286	-0.845523137
H	-3.138661798	-0.010380337	5.766283903
H	6.087237941	-3.73763128	0.78191444
H	-2.719049862	-1.647853804	5.205884969
C	5.37003246	-4.200480321	0.093129193
C	-2.318810909	-0.652857135	5.43049315

H	3.221935763	-4.144178171	1.728279575
H	0.261337652	-1.172041004	4.705432759
H	4.162344584	-2.679455748	2.017747845
H	-0.832944069	-2.059246486	3.642952523
H	5.081539157	-5.164368138	0.53694178
H	-1.626120373	-0.769987519	6.276360082
S	-1.794485586	-3.932513599	1.78905173
O	-2.28240069	-3.751793002	3.172888263
O	-2.457108946	-4.951218195	0.966908085
O	-1.577445531	-2.606438729	1.061525784
C	-0.050428015	-4.565331422	1.99203578
F	0.5380873	-4.693936349	0.788978139
F	0.689327039	-3.731461089	2.738487433
F	-0.085538078	-5.759871645	2.584727602
C	-5.904254269	3.428408454	-2.52935725
N	-4.57231706	3.639833041	-2.472123279
C	-4.026762826	4.602509359	-3.245962843
C	-4.798969676	5.378172105	-4.118668305
C	-6.172541331	5.154399619	-4.188207435
C	-6.73460635	4.168363049	-3.38016384
H	-7.803709283	3.970949551	-3.400425563
H	-6.796058478	5.743163526	-4.858351509
H	-4.325057525	6.144166867	-4.727820397
C	-6.473119879	2.366292868	-1.622583081
H	-7.55563702	2.265444521	-1.756158101
H	-6.01336797	1.388835731	-1.818503301
H	-6.279362322	2.610247767	-0.570072147
C	-2.53822047	4.823678742	-3.126053298
H	-2.20100476	5.631487388	-3.784143591
H	-2.269604989	5.094944061	-2.095302716
H	-1.980186295	3.915780198	-3.391610845
H	-3.289576066	2.663430688	-1.241406448
H	-1.588545365	2.836300562	-0.983778172

Supplementary Table 40 | Cartesian coordinate of the reactant complex for protonation of V. Units are presented in Å.

SCF energy (in toluene) = -5142.76298601 hartree

ZPE = 1.486976 hartree

Atom	X	Y	Z
Mo	-3.149402713	0.420766827	0.02396704
Mo	1.997880702	-0.355821831	0.095769749
P	2.220975083	-1.503612389	2.497478942
P	-3.411488849	2.973931214	-0.125872433
N	-5.416131898	0.716219723	0.016843084
N	4.143722218	-1.026548593	0.082493022
P	2.734070925	0.362049674	-2.289040295
P	-4.065650379	-2.004013786	0.170308959
N	-1.162284024	0.146051537	0.014873031
N	-0.007568151	-0.012967307	0.021338258
N	-3.062217663	0.624749637	3.183174259
N	-3.104292093	0.551110377	2.050550188
N	-3.152871415	0.281972147	-2.014031823
N	-3.149374338	0.211577915	-3.14485736
N	1.753321476	2.48653866	0.992870937
N	2.405792925	1.241273226	0.891960779
H	1.953361183	-0.192357778	6.149168817
H	-2.466580852	6.045016555	2.088789057
H	-3.936765977	5.077453102	2.192877453
H	3.403414722	-0.269908445	5.142828776
C	2.424167149	-0.734817549	5.315849306
C	-3.150053776	5.40944562	1.505292058
H	-2.532522603	3.081031771	2.805195281
H	2.27520706	1.350232614	3.500836227
H	-3.608501071	6.041780304	0.740865268
H	2.589622126	-1.760541629	5.649553564
H	-1.095413997	4.098281633	2.68470661
H	0.935070024	1.367912935	4.667731592
C	1.326768433	0.868451601	3.769529309
C	-1.762791644	3.435058287	2.112558722
H	-0.359853925	-0.575708599	5.168914719
H	-0.483243632	5.294413733	0.752894971
C	1.510873099	-0.628574455	4.075470193
C	-2.352169736	4.221393962	0.925189068

H	-4.75755407	2.866651804	1.869578644
H	4.076342403	-0.008974724	2.806251076
H	0.608241922	1.015134987	2.956937781
H	-1.19193874	2.560770394	1.780153677
H	4.577576747	-1.607013196	3.373680658
H	-5.554900883	3.978143094	0.755736336
C	0.106622124	-1.190322001	4.385739097
C	-1.175257391	4.756682072	0.087394988
H	1.603557631	-3.558833655	4.949172814
H	-3.387744301	5.955740737	-1.399528204
H	-0.548092078	-1.148512492	3.506590245
H	-0.613104324	3.949668153	-0.401207436
C	4.053877537	-1.080773617	2.566600748
C	-5.040003489	3.011925874	0.816774678
H	6.580206683	-1.981474794	2.224852106
H	-7.747539001	3.068375015	0.721180449
H	0.126337679	-2.220669862	4.751115739
H	-1.491685711	5.462440765	-0.68651199
H	3.357206506	-3.542430708	4.642117465
H	-5.055975532	5.487355924	-1.000679152
H	-5.292590917	-0.653160576	2.52326146
H	4.454702902	1.830551747	-0.407919073
C	2.385540697	-3.870221422	4.250544867
C	-4.22579362	5.318160168	-1.698664497
H	-3.927092858	-3.164612384	3.817033247
H	3.482489543	4.128927005	-2.267474897
C	6.100760899	-1.763175512	1.273833309
C	-7.351759494	2.106938251	0.402813832
H	2.397757346	-4.96986505	4.252491854
H	-4.55999623	5.671067115	-2.685306984
C	4.782933002	-1.30405845	1.257250015
C	-5.969963525	1.90360337	0.396746966
H	-5.969226213	-1.978424438	3.478469559
H	5.358328575	2.98521971	-1.42436212
H	-3.005660558	-1.902676444	2.986383805
H	2.363777468	3.168345493	-1.270951383
C	-5.712510192	-1.659777802	2.457883844
C	4.861248442	2.003596934	-1.40934708
C	-3.539848735	-2.845385047	2.838087713
C	2.854856458	3.224600911	-2.247698215
H	-9.281966675	1.222817253	-0.010042937
H	7.806018391	-2.318058452	0.067718752
C	2.151557744	-3.405579568	2.798828054
C	-3.845189712	3.827656911	-1.811814303
H	-6.648551188	-1.608443067	1.890146612
H	5.633916006	1.250790771	-1.601310789
C	-8.203438306	1.081685013	-0.00255677

C	6.782730759	-1.948329094	0.072237454
H	-2.819541765	-3.59930016	2.508649735
H	2.087236325	3.347831547	-3.015125464
H	-0.050817015	-3.463807949	2.902394423
H	-1.770775104	4.244846365	-2.453985846
H	4.250407187	-3.890119509	2.376728144
H	-5.982489911	3.282894419	-1.860947676
C	-4.72515212	-2.687426326	1.85963212
C	3.753600404	1.99388906	-2.485130782
C	0.769262176	-3.886490945	2.31150807
C	-2.649304323	3.67875827	-2.779694476
C	3.25488654	-4.060981632	1.946969089
C	-5.063548968	3.101921487	-2.429931854
H	3.09080543	-5.147807184	1.936082838
H	-5.22486891	3.499138855	-3.44236843
H	-5.907273677	-4.269528324	2.737122772
H	5.06155995	3.026271505	-3.865081825
C	6.130278105	-1.65644133	-1.119772256
C	-7.638769572	-0.128226328	-0.397878391
H	0.614778087	-3.627849612	1.260833744
H	-2.354524647	2.63367333	-2.912435735
C	4.808931868	-1.197082242	-1.09529378
C	-6.25065892	-0.290490621	-0.373018758
C	-5.479013147	-4.028719433	1.752886954
C	4.438477726	2.11940694	-3.862213359
H	-6.313587072	-3.984466471	1.041602668
H	5.103476873	1.274673865	-4.077566078
H	0.714189725	-4.980439882	2.40109134
H	-2.938760464	4.068431868	-3.766597957
H	-8.264192924	-0.955944796	-0.723106187
H	6.627235743	-1.796634448	-2.07610971
H	3.2653821	-3.732034093	0.908740789
H	-4.920737846	2.022838723	-2.521133541
H	-2.843276809	-4.84724543	0.791220945
H	1.720787776	2.144474858	-4.754651087
C	-5.636996093	-1.602394729	-0.783772546
C	4.114094148	-0.90744282	-2.409378885
H	-4.82953906	-4.863440664	1.47250716
H	3.728477641	2.209601532	-4.688769462
H	-6.388402797	-2.399453612	-0.731478908
H	4.862678281	-0.626442269	-3.159831547
H	-1.590056242	-3.632283412	0.442651782
H	0.449019746	1.939060255	-3.521994392
C	-2.353553274	-4.278959657	-0.005971828
C	0.999979922	1.438994654	-4.329174548
H	-5.314528304	-1.540075171	-1.833383924
H	3.653322132	-1.82872652	-2.78604886

C	-3.349601108	-3.474056434	-0.863110277
C	1.629778554	0.108452318	-3.863863117
H	-1.776098193	-2.196925274	-1.714326323
H	-0.143652833	-0.366071182	-2.663285328
H	-1.830556519	-4.997783655	-0.646665327
H	0.271222988	1.208091157	-5.118603879
C	-2.572048686	-2.869694861	-2.04896077
C	0.45929513	-0.812422933	-3.461008912
H	-5.02717464	-4.914501204	-0.66930753
H	3.286647568	0.04284302	-5.334598837
H	-5.103845466	-3.906965537	-2.129615922
H	2.719364481	-1.558829684	-4.786399747
C	-4.425840853	-4.420404132	-1.436935164
C	2.408063236	-0.537810578	-5.029115698
H	-2.103972197	-3.683878646	-2.617457172
H	-0.190015637	-0.947428348	-4.338587956
H	-3.222534707	-2.322467067	-2.739813584
H	0.799763589	-1.797634075	-3.142576916
H	-3.920510172	-5.208535639	-2.013668788
H	1.740994738	-0.603419483	-5.901208623
S	2.329702468	-3.723755405	-1.707434426
O	2.281089867	-3.424820968	-3.157002035
O	3.636744661	-4.162270546	-1.183226277
O	1.646173079	-2.672271143	-0.849048929
C	1.224877923	-5.222089975	-1.566348773
F	1.005102014	-5.554276002	-0.282080861
F	0.036952571	-4.984441363	-2.15236362
F	1.805705684	-6.254539044	-2.181289596
C	4.956983233	4.243002292	1.750806706
N	3.823447417	4.67504263	1.108485177
C	3.71821857	5.900365293	0.499324698
C	4.825062763	6.732788206	0.499891277
C	6.018195018	6.319579668	1.113260839
C	6.07154156	5.070950393	1.743155541
H	6.97620481	4.735643637	2.24281953
H	6.88990963	6.968807567	1.107244692
H	4.7517713	7.705712746	0.02208426
C	4.892359031	2.925050819	2.454730907
H	5.900336965	2.542983899	2.645868162
H	4.388305607	3.03632559	3.427384907
H	4.31970736	2.180669791	1.875715616
C	2.392143226	6.269948447	-0.09396659
H	2.481212326	7.183578404	-0.689328274
H	2.00099693	5.471760234	-0.736307093
H	1.64889527	6.452743044	0.69545652
H	3.035684679	3.996340621	1.008281217
H	1.57232267	2.661695437	1.988395341

```
 H    0.829648488   2.443052918   0.539934561
--------------------------------------------------
```

Supplementary Table 41 | Cartesian coordinate of the transition state for protonation of V. Units are presented in Å.

SCF energy (in toluene) = -5142.75689633 hartree

ZPE = 1.483863 hartree

imaginary frequency: 1073*i*

Atom	X	Y	Z
Mo	-3.166950834	0.264719998	0.037267371
Mo	2.029435834	-0.195665343	0.086184158
P	2.311630419	-1.36186074	2.471784185
P	-3.612358092	2.78043877	-0.136807538
N	-5.451946485	0.406746569	0.077323519
N	4.197382594	-0.814123341	0.046747822
P	2.723813658	0.534894237	-2.327075892
P	-3.928119796	-2.216701882	0.224555572
N	-1.153965395	0.145566495	0.013484433
N	0.009428618	0.082603746	0.027925851
N	-3.045776238	0.525863975	3.189435782
N	-3.100344596	0.429279634	2.058535249
N	-3.190546227	0.098990088	-1.99772073
N	-3.200870373	0.017018573	-3.127967863
N	1.808139055	2.664865739	1.017349076
N	2.514668085	1.386793146	0.908119217
H	2.01714333	-0.051833393	6.122276933
H	-2.809158516	5.931228867	2.018473378
H	-4.200306364	4.860594401	2.185901503
H	3.460419409	-0.072030939	5.102916883
C	2.502739687	-0.576275763	5.285914941
C	-3.466027096	5.241656281	1.466509799
H	-2.616225703	2.982975211	2.763512584
H	2.245203342	1.49699761	3.469950764
H	-3.998069906	5.83130473	0.715594808
H	2.713356546	-1.593756048	5.62014284
H	-1.267927549	4.103965028	2.564108042
H	0.907863815	1.456678385	4.641538755
C	1.321622474	0.973377945	3.744517684
C	-1.908121449	3.383030352	2.031523318
H	-0.28542612	-0.538270047	5.163603383
H	-0.852927402	5.336727365	0.602606522

C	1.575533022	-0.513488266	4.053142932
C	-2.607632692	4.108144944	0.863712432
H	-4.886151744	2.604383458	1.902242751
H	4.122967197	0.181964687	2.783567367
H	0.59064309	1.076699448	2.934126406
H	-1.289395815	2.547911337	1.682467518
H	4.674133717	-1.407334014	3.329039285
H	-5.787554678	3.65473187	0.806883569
C	0.199402782	-1.134041641	4.377027952
C	-1.509102406	4.718186563	-0.025909053
H	1.786189402	-3.4375327	4.926039392
H	-3.833916314	5.744475052	-1.443928709
H	-0.464722315	-1.121128568	3.504182324
H	-0.892700048	3.944436161	-0.505036451
C	4.13007312	-0.886402717	2.532048424
C	-5.208780916	2.7245847	0.857783937
H	6.683467274	-1.692455179	2.164434197
H	-7.916126585	2.601074246	0.837769636
H	0.265421987	-2.161910629	4.743524656
H	-1.90598571	5.367352154	-0.811732687
H	3.535012116	-3.355256241	4.604188433
H	-5.445565612	5.166933348	-0.966650603
H	-5.196704855	-0.917887306	2.590156793
H	4.426514747	2.069551264	-0.486601645
C	2.57319096	-3.719716	4.220770838
C	-4.637981139	5.047043219	-1.7000351
H	-3.644721833	-3.32095087	3.879667517
H	3.369504039	4.323878789	-2.334411453
C	6.189076826	-1.485641269	1.218477159
C	-7.466046327	1.666839884	0.510679794
H	2.626961094	-4.818094719	4.223056316
H	-5.039089643	5.364555111	-2.673618565
C	4.855284252	-1.074588716	1.214395787
C	-6.074465139	1.555464285	0.469165432
H	-5.769850044	-2.273433423	3.570164897
H	5.283124623	3.242643756	-1.523564573
H	-2.830116937	-2.007235534	3.018363065
H	2.263805895	3.331060194	-1.364742327
C	-5.552821856	-1.949727427	2.541900392
C	4.815963052	2.246729578	-1.49428853
C	-3.300800466	-2.986104358	2.88977748
C	2.769035578	3.401742981	-2.332440757
H	-9.343236636	0.655961537	0.146457877
H	7.904564299	-1.969106458	-0.003833559
C	2.310107782	-3.265241705	2.770703567
C	-4.160562511	3.584833799	-1.816517988
H	-6.500680183	-1.963568812	1.9919915

H	5.608018356	1.515163859	-1.688591833
C	-8.257859855	0.586622945	0.127445512
C	6.868302165	-1.637673987	0.010692
H	-2.537447466	-3.692275518	2.551372431
H	2.006274335	3.506241879	-3.10723343
H	0.111683957	-3.400896219	2.886306383
H	-2.130043556	4.104007074	-2.529070689
H	4.421446759	-3.678351045	2.332916214
H	-6.259893964	2.905624321	-1.793477974
C	-4.513247173	-2.9188248	1.934754406
C	3.697936853	2.192571659	-2.557970407
C	0.943578439	-3.795616411	2.292844149
C	-2.996544673	3.507176998	-2.830265851
C	3.429241209	-3.881050395	1.909597261
C	-5.347441814	2.771196323	-2.385082747
H	3.301257448	-4.972677531	1.897222217
H	-5.561760125	3.136683675	-3.399709865
H	-5.568043366	-4.563307873	2.858094285
H	4.959387019	3.244473679	-3.966598398
C	6.19605877	-1.363875171	-1.174213993
C	-7.62388748	-0.584052235	-0.280610575
H	0.776355064	-3.544144438	1.242476535
H	-2.664584721	2.478690876	-2.99658535
C	4.857969391	-0.95508957	-1.136338109
C	-6.227844654	-0.653994438	-0.28872563
C	-5.180244377	-4.30733779	1.860985615
C	4.36287041	2.320186532	-3.944726027
H	-6.032770866	-4.325926763	1.170487691
H	5.04959926	1.492455157	-4.156922611
H	0.927282363	-4.890722023	2.383892043
H	-3.341274815	3.901937547	-3.797212923
H	-8.200506274	-1.452625236	-0.589071497
H	6.68994877	-1.481498464	-2.135182497
H	3.422044397	-3.549049857	0.872169474
H	-5.139930524	1.701045276	-2.459964977
H	-2.530780825	-4.972847866	0.851262364
H	1.626529295	2.253288919	-4.800668345
C	-5.537348566	-1.923759976	-0.70568307
C	4.142860263	-0.691516135	-2.445697551
H	-4.484800354	-5.101374705	1.573095572
H	3.640919182	2.378685495	-4.763788394
H	-6.232757933	-2.768742653	-0.637907582
H	4.875991082	-0.388866745	-3.202832592
H	-1.357805782	-3.694612088	0.456162269
H	0.382564263	2.026201074	-3.542773425
C	-2.092343073	-4.390381237	0.034569508
C	0.933715262	1.532700225	-4.354078077

H	-5.235169738	-1.844983395	-1.760246564
H	3.71091233	-1.628289024	-2.817506312
C	-3.150730951	-3.655229894	-0.809164685
C	1.611460637	0.22709555	-3.885684495
H	-1.672002104	-2.298094714	-1.704435186
H	-0.126623115	-0.297505202	-2.652772676
H	-1.544238849	-5.088519647	-0.607759458
H	0.198224099	1.271764198	-5.127656959
C	-2.435039132	-3.018370522	-2.01676286
C	0.478305848	-0.728515838	-3.457402378
H	-4.734987572	-5.19068625	-0.564778794
H	3.249410854	0.202163651	-5.378684017
H	-4.901254008	-4.20736163	-2.034112127
H	2.744514584	-1.411970063	-4.805561916
C	-4.179984402	-4.670659805	-1.350089426
C	2.395670639	-0.404848636	-5.054779344
H	-1.933802985	-3.809276378	-2.589448146
H	-0.179638632	-0.891969983	-4.323576043
H	-3.130336082	-2.514700558	-2.697026075
H	0.856636435	-1.699115797	-3.137046248
H	-3.64003229	-5.43406968	-1.928701822
H	1.719118125	-0.502655483	-5.916492923
S	2.480999967	-3.566284897	-1.743578865
O	2.40934114	-3.281786165	-3.19539852
O	3.812182353	-3.933320735	-1.224038012
O	1.749702978	-2.543315729	-0.891640308
C	1.462774513	-5.123701474	-1.587310088
F	1.254841294	-5.451387068	-0.299429063
F	0.266822586	-4.964556086	-2.182807632
F	2.106589764	-6.12903062	-2.184414074
C	4.772297591	4.345899268	1.743679553
N	3.572094085	4.631556797	1.17893222
C	3.306227094	5.858613947	0.659973205
C	4.2888658	6.846979277	0.660820643
C	5.542331758	6.569277586	1.208079807
C	5.78146493	5.313789867	1.758681701
H	6.741830508	5.070742456	2.205457112
H	6.321280313	7.328908458	1.20996915
H	4.065646276	7.822111454	0.236704728
C	4.961808722	2.99785884	2.37615415
H	6.015852509	2.701083009	2.342048231
H	4.666898171	3.040180353	3.436016854
H	4.345255359	2.233792244	1.882758319
C	1.923571082	6.115681118	0.130492057
H	1.87351363	7.09203919	-0.361070646
H	1.614637731	5.351626278	-0.591357817
H	1.193719119	6.115211998	0.950831152

H	2.706974839	3.717296108	1.069586307
H	1.343291406	2.664520304	1.934727545
H	1.047215793	2.726141596	0.3255895

Supplementary Table 42 | Cartesian coordinate of the product complex for protonation of V. Units are presented in Å.

SCF energy (in toluene) = -5142.76598702 hartree

ZPE = 1.487369 hartree

Atom	X	Y	Z
Mo	-2.908412705	-0.143291308	-0.042507989
Mo	2.216046337	0.675570716	0.412347894
P	2.780739776	-1.003377369	2.426723672
P	-3.937054422	2.190009014	0.222617886
N	-5.153961586	-0.550947642	-0.123596205
N	4.472126682	0.703068712	0.423775733
P	2.746264938	2.338589984	-1.565966977
P	-3.027927046	-2.709580196	-0.397962372
N	-0.932776224	0.23076755	0.096261848
N	0.203533584	0.454546107	0.235937121
N	-2.95237981	-0.503168843	3.102145227
N	-2.946488716	-0.383542728	1.972517393
N	-2.844150679	0.124272124	-2.068045231
N	-2.814665301	0.285508149	-3.18834917
N	1.119590864	2.936761001	2.022242222
N	2.270210838	2.031597472	1.675405569
H	2.081812222	-0.869985625	6.247756144
H	-3.888709878	4.977578967	2.947305085
H	-5.021514646	3.629478414	2.833162189
H	3.466231228	-0.183113177	5.391894367
C	2.709341343	-0.976594054	5.350545254
C	-4.365970392	4.280268537	2.24243597
H	-3.10965618	2.036727926	3.176691218
H	1.859037597	1.349847804	4.103285993
H	-4.990315262	4.87253798	1.569696762
H	3.220269567	-1.938519602	5.424577586
H	-2.00981455	3.413610788	3.270344427
H	0.594432944	0.618230302	5.116109514
C	1.133701344	0.530836388	4.161992794
C	-2.475736526	2.696468939	2.57615319
H	0.041339083	-1.699208029	5.011238703
H	-1.770746173	5.038060254	1.575566456
C	1.813548146	-0.849776159	4.099423287
C	-3.269997807	3.476979398	1.510670746

H	-5.176808748	1.338261144	2.107400721
H	4.061840629	0.843496321	3.274112957
H	0.399320205	0.624899532	3.351916849
H	-1.697641093	2.073371797	2.120097209
H	5.036671393	-0.621194853	3.470931026
H	-6.281031925	2.332730676	1.154790506
C	0.67704893	-1.8937655	4.135776129
C	-2.294256059	4.445041604	0.812870926
H	2.855703151	-3.721446969	4.210515979
H	-4.806191524	5.228945424	-0.521123776
H	0.0393237	-1.823940715	3.24581791
H	-1.540624002	3.9102889	0.216523223
C	4.382737031	-0.08092362	2.776112529
C	-5.493967538	1.572303041	1.08062398
H	7.068322956	-0.070560119	2.447126222
H	-8.08592229	0.811717782	0.882321062
H	1.039299718	-2.921401674	4.218285978
H	-2.799375844	5.15405654	0.150223831
H	4.507708634	-3.07560388	4.067336186
H	-6.254786961	4.229393958	-0.269149907
H	-4.634075973	-2.30750931	2.085065569
H	3.951816819	3.592367345	0.842421939
C	3.695927756	-3.576666973	3.525136183
C	-5.423074778	4.435249177	-0.954777536
H	-2.47676738	-4.443788207	2.971699621
H	2.446325214	6.027873336	-0.457361965
C	6.552130008	0.263290431	1.550550435
C	-7.417718264	0.090564539	0.417944977
H	4.064783458	-4.576395549	3.253563627
H	-5.861379093	4.839861507	-1.878587057
C	5.156888668	0.29680584	1.531388178
C	-6.040776293	0.323678079	0.434911349
H	-4.817689491	-3.93695948	2.743725614
H	4.539718683	5.17133417	0.267698214
H	-2.066882975	-2.79874496	2.466068855
H	1.651078399	4.612141833	0.223585747
C	-4.686574636	-3.364758363	1.813924947
C	4.321430534	4.136230088	-0.033524919
C	-2.237689609	-3.815113812	2.101193624
C	2.087360161	5.015897473	-0.694787331
H	-8.987748972	-1.250175913	-0.225972913
H	8.353969123	0.623525648	0.413461591
C	3.322745378	-2.841712883	2.221148616
C	-4.618390507	3.167513858	-1.307084233
H	-5.586921974	-3.521986028	1.208978175
H	5.268810939	3.684849116	-0.347366427
C	-7.918086102	-1.05486227	-0.197214455

C	7.266383136	0.653892651	0.41937553
H	-1.302241646	-4.189055553	1.674037257
H	1.29480765	5.114154722	-1.439738077
H	1.26056325	-3.621508448	2.123214176
H	-2.790440918	4.312234912	-1.796817085
H	5.461536294	-2.501552304	1.85797283
H	-6.494169946	2.027795813	-1.533531667
C	-3.413338761	-3.875766319	1.101852585
C	3.280819	4.164882861	-1.174637509
C	2.169481448	-3.580305075	1.512508765
C	-3.447219829	3.556423653	-2.238225518
C	4.576510835	-2.871958858	1.325441614
C	-5.570669367	2.231215196	-2.087314458
H	4.781452577	-3.916262866	1.049747368
H	-5.85708202	2.729907996	-3.024217298
H	-3.990327258	-5.89256871	1.618233008
H	4.291934221	5.850276514	-2.077215275
C	6.564360496	1.085450765	-0.699739627
C	-7.014511217	-1.943066842	-0.774603066
H	1.929193666	-3.104323414	0.558381499
H	-2.836405788	2.694668197	-2.519681506
C	5.165274714	1.104385507	-0.677667298
C	-5.643610629	-1.676498642	-0.719600776
C	-3.673105636	-5.346543677	0.717652821
C	3.928936102	4.860668607	-2.391413726
H	-4.476889975	-5.451938823	-0.021696492
H	4.796312495	4.311265278	-2.775507751
H	2.475830482	-4.615046083	1.303772666
H	-3.857631921	3.986086511	-3.163408158
H	-7.360031783	-2.848769835	-1.266904773
H	7.087322511	1.397150387	-1.600013505
H	4.473433313	-2.311274226	0.396180809
H	-5.109840801	1.276739061	-2.351578646
H	-0.925751095	-5.058308513	-0.266829165
H	1.310859759	4.45270173	-3.494644873
C	-4.661290026	-2.642253775	-1.328685669
C	4.424519279	1.569778559	-1.913662785
H	-2.781812163	-5.851164618	0.332807211
H	3.232104258	5.017804773	-3.220023689
H	-5.127119224	-3.628459956	-1.442668238
H	5.068959714	2.247526743	-2.485652174
H	-0.162301235	-3.453218855	-0.345677023
H	0.134245978	3.555278559	-2.49993547
C	-0.675848262	-4.224628526	-0.931306141
C	0.810315985	3.488462661	-3.362852966
H	-4.394504139	-2.298503029	-2.338687945
H	4.238006156	0.714082467	-2.573751734

C	-1.908330668	-3.656448138	-1.660194619
C	1.779360781	2.293672373	-3.245286193
H	-0.83420766	-1.82580781	-2.254254512
H	0.190855843	1.036916925	-2.403912582
H	0.040808347	-4.597297134	-1.672630926
H	0.18145732	3.328715575	-4.249787121
C	-1.417765257	-2.635262188	-2.70579687
C	0.907823798	1.019981642	-3.231731702
H	-3.023192205	-5.576184624	-1.77604076
H	3.401108882	3.112644175	-4.511294052
H	-3.462739088	-4.404625398	-3.034544901
H	3.3039485	1.330684369	-4.49642754
C	-2.635333	-4.785819294	-2.423339293
C	2.719261144	2.255621769	-4.467804322
H	-0.76923923	-3.153225356	-3.423309477
H	0.33755019	0.979780776	-4.171567417
H	-2.242330628	-2.195235721	-3.276174767
H	1.504773313	0.109792831	-3.165743527
H	-1.918464144	-5.25201304	-3.114579352
H	2.106991094	2.278097062	-5.381051954
S	3.505845047	-1.7025246	-2.191003381
O	3.382143536	-1.017096174	-3.496511876
O	4.875878049	-1.891205812	-1.680362094
O	2.528259586	-1.179069985	-1.146784158
C	2.891064662	-3.423507754	-2.567208957
F	2.827926269	-4.176231156	-1.454573327
F	1.665542514	-3.374346736	-3.116816122
F	3.72676606	-4.00408297	-3.42989492
C	3.377190137	5.167925002	4.064066389
N	2.217697026	5.332213052	3.388955151
C	1.687845185	6.572003767	3.282662441
C	2.312460485	7.6935647	3.835512408
C	3.516956955	7.530987359	4.519598477
C	4.054463722	6.252741457	4.636917737
H	4.988339152	6.085034133	5.168034431
H	4.025991778	8.38851869	4.955149402
H	1.857996422	8.675297667	3.727659673
C	3.905877722	3.764698253	4.213312168
H	4.984807709	3.767198914	4.406985016
H	3.42132397	3.264890523	5.066324912
H	3.700844298	3.166890923	3.316736585
C	0.376599573	6.698526753	2.549973416
H	0.048382683	7.741908748	2.49567186
H	0.455639397	6.314426936	1.524439292
H	-0.408072113	6.125291177	3.062774282
H	1.485512965	3.833154007	2.460277924
H	0.531771598	2.460674404	2.719096065

H	0.526437286	3.135915037	1.205880385

Supplementary Table 43 | Cartesian coordinate of the reactant complex for protonation of IX. Units are presented in Å.

SCF energy (in toluene) = -3106.71020800 hartree

ZPE = 0.837539 hartree

Atom	X	Y	Z
Mo	0.441189	0.058288165	-0.686129409
P	-0.670861103	2.341608145	-0.683735007
N	2.28185543	1.534548221	-0.200360327
P	2.549116342	-1.308007536	-1.041167246
N	-1.536374521	-2.178873005	-0.423003632
N	-0.806031229	-1.192990266	-1.119394879
H	-2.144801775	4.248179523	-3.730378306
H	-0.635593109	4.587837994	-2.881923712
C	-1.641301611	4.168315184	-2.755874355
H	0.184001014	2.172312589	-3.527690624
H	-2.188254721	4.801946112	-2.053816414
H	-1.348467256	2.086897493	-4.403860278
C	-0.871998904	1.886275488	-3.432571392
H	-3.528724187	2.180818408	-3.207946194
C	-1.606931025	2.685068639	-2.331687411
H	1.076415923	3.44498845	-1.948979063
H	-0.918991705	0.805038095	-3.253252285
H	0.660085728	4.45338353	-0.570962034
C	-3.044103768	2.13786502	-2.221412996
H	-3.167317058	4.335888594	-0.160193982
H	-3.046061638	1.091680587	-1.888058379
C	0.854065501	3.417486906	-0.872376685
H	2.811450803	4.818486474	0.412210999
H	-3.660507136	2.722034138	-1.529134119
H	-1.643326486	5.211533337	0.11592477
H	2.652807645	1.090007785	-2.831015332
C	-2.318748023	4.449169974	0.524278377
H	2.593903418	-1.154358163	-4.860758412
C	3.012876174	3.750419252	0.391236956
H	-2.717172914	4.846223893	1.469386443
C	2.07769936	2.879316889	-0.169297331
H	3.908799851	0.66633968	-4.008961293
H	1.279817756	-0.905865792	-3.694004045
C	3.55757331	0.489389327	-2.982353327

C	2.232378966	-1.427482548	-3.858838266
H	4.946985616	3.895955633	1.345078665
C	-1.595951971	3.120979175	0.816030375
H	4.333019528	0.862146056	-2.302888271
C	4.206215947	3.236594547	0.897804964
H	2.027237186	-2.501976923	-3.865743771
H	-3.367470272	1.81977877	0.589341924
H	0.135984709	4.193224302	1.660607778
C	3.289481899	-1.022320854	-2.807725585
C	-2.607161482	2.077278275	1.338526622
C	-0.546797312	3.374758493	1.919982969
H	-1.0719949	3.676111186	2.837609257
H	4.997581749	-1.454867478	-4.066001391
C	4.443846815	1.869058763	0.796914567
H	-2.086743688	1.165619597	1.652427144
C	3.466974696	1.037578368	0.238347488
C	4.614315101	-1.761547687	-3.081792482
H	5.38848984	-1.514429156	-2.343905508
H	-3.130335034	2.490260764	2.213151607
H	5.374301449	1.435126417	1.154431842
H	0.037904928	2.485624813	2.170479833
H	2.853297636	-4.065482107	-2.598088398
C	3.7332333	-0.446340303	0.115711723
H	4.4993859	-2.849573601	-3.107174146
H	4.785496718	-0.611951467	-0.151995196
H	1.194498794	-3.774978772	-2.032677057
C	2.205053316	-4.066786265	-1.716314493
H	3.567757331	-0.901595786	1.101958284
C	2.730691796	-3.172369066	-0.574700484
H	0.793515214	-3.237961532	0.444280165
H	2.153541984	-5.103561987	-1.354412747
C	1.849338284	-3.428230089	0.662960152
H	4.884933502	-3.407031283	-1.040885774
H	4.545964384	-3.023054214	0.659358725
C	4.180281598	-3.565673923	-0.220248609
H	1.949207171	-4.484525601	0.953978392
H	2.129438787	-2.809887577	1.519088145
H	4.203231539	-4.636083737	0.030857615
H	-1.395781979	-3.089146969	-0.874929185
S	0.750285544	-0.337556486	2.845008783
O	2.063500185	-1.00306126	2.758026235
O	0.635920419	0.834611018	3.720360378
O	0.048178772	-0.146031339	1.499250939
C	-0.404607965	-1.603881323	3.591911934
F	-1.60804616	-1.050864275	3.835949995
F	-0.603900685	-2.639588318	2.732475435
F	0.089775038	-2.09078707	4.725526236

H	-1.182031781	-2.247594072	0.540750236
C	-4.917888017	-1.493342218	0.713899619
N	-4.245035033	-1.6302212	-0.458835757
C	-4.821487924	-1.555182449	-1.685162888
C	-6.191394915	-1.30759556	-1.756914485
C	-6.925615043	-1.159783707	-0.579188218
C	-6.289372419	-1.254688189	0.660682674
H	-6.845462796	-1.143177199	1.586960703
H	-7.995967871	-0.970738529	-0.626533676
H	-6.669858736	-1.237797103	-2.729725593
C	-3.943158959	-1.77406094	-2.884069533
H	-4.367915739	-1.280689034	-3.764053326
H	-2.92577369	-1.397958666	-2.716399699
H	-3.872491589	-2.848141204	-3.110791445
C	-4.130229597	-1.593385298	1.986692608
H	-4.796765607	-1.558323698	2.853222079
H	-3.554400455	-2.526269784	2.033915023
H	-3.41388032	-0.767167819	2.072841808
H	-3.178528865	-1.8318016	-0.419023097

Supplementary Table 44 | Cartesian coordinate of the transition state for protonation of IX. Units are presented in Å.

SCF energy (in toluene) = -3106.70281635 hartree

ZPE = 0.833893 hartree

imaginary frequency: 967*i*

Atom	X	Y	Z
Mo	0.417341928	0.03430861	-0.671770101
P	-0.650227024	2.333915248	-0.648112727
N	2.2802417	1.476518709	-0.137754061
P	2.517312022	-1.34674811	-1.047040955
N	-1.629562098	-2.044454827	-0.18687632
N	-0.907014647	-1.166138092	-1.062173401
H	-2.028743645	4.226656119	-3.741486408
H	-0.552401129	4.57472311	-2.839592147
C	-1.559696628	4.149251059	-2.749941139
H	0.305083308	2.147723452	-3.444505968
H	-2.13540778	4.778972913	-2.067491451
H	-1.189343698	2.063537274	-4.382775947
C	-0.755109585	1.86543679	-3.391666337
H	-3.424593792	2.162877002	-3.263232417
C	-1.534967344	2.666875923	-2.323643467
H	1.133713288	3.450575914	-1.85283438
H	-0.820368361	0.783143192	-3.217301664
H	0.700083389	4.425085881	-0.456629015
C	-2.972031673	2.111242794	-2.262279156
H	-3.178954562	4.283943063	-0.197893399
H	-2.982540187	1.061307824	-1.942400996
C	0.889374862	3.395961502	-0.782167677
H	2.834366523	4.731517639	0.594093312
H	-3.613079485	2.686451489	-1.58517603
H	-1.679432509	5.182217627	0.137985501
H	2.702902664	1.105303021	-2.757527022
C	-2.357425873	4.406665354	0.516658292
H	2.604984602	-1.08402812	-4.858829515
C	3.02329183	3.662346791	0.541299695
H	-2.79511759	4.790658544	1.44949552
C	2.091034968	2.822234587	-0.068204728
H	3.952817314	0.680272183	-3.940259571

H	1.28356651	-0.833200861	-3.703479118
C	3.589745149	0.482096304	-2.921881906
C	2.224667309	-1.375296204	-3.869236689
H	4.936374661	3.751233933	1.542984051
C	-1.623118331	3.089374814	0.828568464
H	4.370948496	0.811907221	-2.227034013
C	4.198637023	3.116458756	1.056897521
H	1.990623526	-2.44304348	-3.908920528
H	-3.34752939	1.73597188	0.538162895
H	0.056979943	4.19627526	1.729493467
C	3.279876826	-1.026755376	-2.796020674
C	-2.628989222	2.023927639	1.316013005
C	-0.609951609	3.356710937	1.96132703
H	-1.167151819	3.634220069	2.867598539
H	4.989836876	-1.466410485	-4.048098281
C	4.423297757	1.750498089	0.915749803
H	-2.099671893	1.129148066	1.662438284
C	3.449214661	0.949835053	0.309113477
C	4.589000964	-1.791231484	-3.076955503
H	5.360755698	-1.583851634	-2.324534788
H	-3.200669422	2.430984965	2.162482699
H	5.34047249	1.293973292	1.279344125
H	-0.006952551	2.48086573	2.216996929
H	2.772567671	-4.061536482	-2.677245143
C	3.701827226	-0.53359855	0.143990407
H	4.447369612	-2.87477469	-3.134154849
H	4.753780833	-0.70135968	-0.12384341
H	1.111467086	-3.752536803	-2.125606421
C	2.111586761	-4.074195254	-1.805142085
H	3.529251753	-1.017409707	1.115282045
C	2.639425437	-3.226644703	-0.629036501
H	0.681680898	-3.309640139	0.352828826
H	2.033761826	-5.119735873	-1.47491688
C	1.734126206	-3.504419676	0.587606806
H	4.796140702	-3.486681289	-1.069599835
H	4.43727866	-3.153253194	0.637521631
C	4.075901739	-3.659208424	-0.265410149
H	1.825614614	-4.567371806	0.855278103
H	1.998220854	-2.90691239	1.463370792
H	4.074566087	-4.737612291	-0.051102347
H	-1.441237151	-3.016638449	-0.461394504
S	0.769907524	-0.38984772	2.827399015
O	2.019690274	-1.168232865	2.755333935
O	0.798871986	0.87380118	3.57205102
O	0.015620394	-0.286999271	1.493045218
C	-0.449161436	-1.478589735	3.732431895
F	-1.594649327	-0.812100052	3.953901017

F	-0.748725677	-2.569419344	2.979214454
F	0.055151619	-1.888089991	4.890764946
H	-1.281078983	-1.916178187	0.777792922
C	-4.931701546	-1.612332985	0.817549804
N	-4.212792795	-1.736822331	-0.325219153
C	-4.798204472	-1.753610481	-1.545525231
C	-6.184876317	-1.61774181	-1.651723867
C	-6.949170358	-1.484256591	-0.493781642
C	-6.319547949	-1.485121835	0.752041929
H	-6.89290647	-1.387165723	1.669860246
H	-8.030454413	-1.382400698	-0.55988765
H	-6.650593335	-1.624508166	-2.633743084
C	-3.912591503	-1.957785503	-2.746540079
H	-4.362990097	-1.511701044	-3.639700773
H	-2.914624537	-1.527061557	-2.596244593
H	-3.787367704	-3.032229779	-2.948267827
C	-4.177752717	-1.605126739	2.120138407
H	-4.870319901	-1.608199087	2.967365582
H	-3.523174465	-2.480795167	2.217539441
H	-3.544537824	-0.713293936	2.206801283
H	-2.919732451	-1.867493875	-0.24879658

Supplementary Table 45 | Cartesian coordinate of the product complex for protonation of IX. Units are presented in Å.

SCF energy (in toluene) = -3106.70689941 hartree

ZPE = 0.837796 hartree

Atom	X	Y	Z
Mo	0.464455677	0.043523483	-0.797841915
P	-0.608292433	2.330498394	-0.749918236
N	2.2929667	1.486654742	-0.157234007
P	2.604846735	-1.291491334	-1.170116891
N	-1.615530555	-1.923081533	-0.153415157
N	-0.927947667	-1.10948394	-1.145365567
H	-1.861347726	4.20216564	-3.906046314
H	-0.436147289	4.5731272	-2.933750199
C	-1.439280761	4.130496046	-2.893305481
H	0.496998317	2.160237055	-3.479915468
H	-2.05822207	4.749007188	-2.238261876
H	-0.940946486	2.031009496	-4.497668727
C	-0.558336699	1.8555603	-3.481440823
H	-3.23419376	2.10957707	-3.511401104
C	-1.41398172	2.649047744	-2.465882035
H	1.199982948	3.520937851	-1.8438969
H	-0.614392596	0.771158859	-3.304108815
H	0.699409294	4.430645646	-0.426003799
C	-2.842816517	2.070378224	-2.484674038
H	-3.188208946	4.210868121	-0.408196361
H	-2.859574185	1.022457024	-2.160651622
C	0.916388403	3.418831122	-0.786578224
H	2.798917406	4.715059977	0.710268681
H	-3.531461106	2.641255869	-1.85235848
H	-1.728710024	5.125889577	0.038925194
H	2.87870641	1.240091	-2.746244297
C	-2.415378265	4.33138546	0.358457521
H	2.950565723	-0.870324688	-4.952862459
C	2.995136269	3.649057637	0.628878835
H	-2.915943469	4.68689824	1.270549395
C	2.094124002	2.82745882	-0.048047299
H	4.198484365	0.877139689	-3.872366735
H	1.552939377	-0.631205184	-3.890887503
C	3.779368773	0.631280878	-2.886494616

C	2.494706242	-1.185711736	-4.003617936
H	4.862314531	3.709701052	1.715234355
C	-1.677752601	3.019671631	0.685949132
H	4.517251443	0.933967767	-2.134477971
C	4.149146079	3.089469223	1.176640102
H	2.249003785	-2.24720901	-4.094642164
H	-3.345468845	1.631180738	0.253256804
H	-0.085210261	4.142727107	1.713622739
C	3.474010596	-0.882796659	-2.847802559
C	-2.687310709	1.921257779	1.080403926
C	-0.735452829	3.27508745	1.881047394
H	-1.348189974	3.501514883	2.765338461
H	5.26478881	-1.269677257	-3.998597791
C	4.383990459	1.729310692	1.003370079
H	-2.162936539	1.030229473	1.442194618
C	3.439807223	0.94735899	0.329881793
C	4.803994799	-1.630033076	-3.06766541
H	5.522225598	-1.443505976	-2.25911761
H	-3.322917701	2.293987551	1.896533868
H	5.283926103	1.262573178	1.395762108
H	-0.108107444	2.41386547	2.129506843
H	3.019907291	-3.916473424	-2.90886025
C	3.69474905	-0.533497013	0.141698668
H	4.67483392	-2.711494472	-3.169180316
H	4.764304611	-0.705343414	-0.038606817
H	1.311950481	-3.661099116	-2.482329395
C	2.288787937	-3.982366195	-2.097442267
H	3.437673939	-1.052075523	1.077199415
C	2.699960043	-3.190753591	-0.838646053
H	0.663142287	-3.342361268	-0.067506832
H	2.20336402	-5.044312697	-1.827177462
C	1.687553156	-3.537131694	0.271527877
H	4.891260493	-3.400376757	-1.092591132
H	4.372313591	-3.173915026	0.592045078
C	4.103740219	-3.628544353	-0.368576118
H	1.765893909	-4.611636479	0.492117428
H	1.854784485	-2.98710808	1.201511292
H	4.100132602	-4.717662562	-0.219419084
H	-1.442222114	-2.917186969	-0.350955373
S	0.895628223	-0.392952064	2.673845793
O	1.9166446	-1.454219199	2.738347349
O	1.280197776	0.950363022	3.123130651
O	0.098143694	-0.378006761	1.35447176
C	-0.494371794	-0.964154257	3.782659173
F	-1.465748867	-0.038201744	3.824206721
F	-1.025961664	-2.105453331	3.285818557
F	-0.046467464	-1.19355589	5.011195831

H	-1.234403658	-1.702278421	0.788957507
C	-5.087172974	-1.571516329	0.991114597
N	-4.403556904	-1.678529369	-0.171305276
C	-5.061820069	-1.659606358	-1.350346065
C	-6.452479956	-1.505378408	-1.401241306
C	-7.166233187	-1.386384246	-0.210990125
C	-6.477252581	-1.424630886	1.001441335
H	-7.005410432	-1.34182971	1.94801552
H	-8.248014864	-1.268564029	-0.226002243
H	-6.95974913	-1.487265266	-2.362885236
C	-4.24776529	-1.855340404	-2.60574639
H	-4.742175849	-1.403837765	-3.473176664
H	-3.242869746	-1.42634362	-2.50931001
H	-4.130591004	-2.927986229	-2.821932061
C	-4.289581091	-1.61819945	2.270598596
H	-4.949088119	-1.631927235	3.144601349
H	-3.658300015	-2.515626145	2.314183004
H	-3.628476164	-0.746877039	2.366692406
H	-2.692126078	-1.777629651	-0.17304515

Supplementary Table 46 | Cartesian coordinate of the reactant complex for protonation of XI. Units are presented in Å.

SCF energy (in toluene) = -3050.84437788 hartree

ZPE = 0.810189 hartree

Atom	X	Y	Z
Mo	0.445715914	0.162627326	-0.0993269
P	0.27710922	2.54576064	0.871507359
N	1.919020395	1.278522297	-1.421247794
P	1.766347114	-1.726317333	-1.086496071
N	-0.918204086	0.001174918	-1.066088275
H	-1.994867291	5.560177769	0.035464751
H	-0.260478216	5.56453439	0.372390039
C	-1.198844574	5.036278051	0.584363547
H	-0.136629594	4.091636985	-1.751497272
H	-1.42128268	5.137471002	1.651224831
H	-1.89811631	4.031735428	-1.855797702
C	-1.02109183	3.53936047	-1.409378171
H	-3.332850677	3.43024549	0.050296786
C	-1.171003117	3.565211178	0.126950322
H	1.724973518	4.35091378	-0.109812048
H	-0.962487666	2.515266533	-1.793252977
H	2.616328142	3.135438729	0.78202672
C	-2.499608969	2.882023432	0.514974166
H	0.512667638	5.237041239	2.583368809
H	-2.529102171	1.842269782	0.172286594
C	1.807239472	3.268779425	0.049418763
H	3.276887192	4.335595599	-1.933063156
H	-2.666356929	2.878882859	1.59690088
H	2.155219713	4.594834172	2.380191236
H	-0.796824063	-2.235072583	-2.354372779
C	1.19925101	4.446600133	2.897511469
H	-0.256923958	-4.928290032	-0.791217428
C	3.055526691	3.287934898	-2.120046443
H	1.394295783	4.593163883	3.969355245
C	2.245951854	2.58844365	-1.219887036
H	-0.73851301	-3.932050025	-2.880815277
H	-0.451477014	-3.350169897	0.005571262
C	-0.131989707	-3.031821549	-2.709045761
C	0.274654514	-4.025717255	-0.456618198

H	4.228527647	3.159455843	-3.929577159
C	0.626892132	3.025128317	2.704734576
H	0.285371025	-2.739681299	-3.680740166
C	3.589605957	2.63134953	-3.224905032
H	0.980180596	-4.332133257	0.320405532
H	-1.383741998	3.670685425	3.32170705
H	2.622489978	2.068806291	2.63829437
C	0.96288177	-3.371265682	-1.673073789
C	-0.660700864	2.875725581	3.541437553
C	1.682137142	2.011948389	3.206006764
H	1.929075056	2.246543997	4.250853743
H	1.408912367	-5.22354601	-2.700010819
C	3.308242478	1.278721631	-3.396361791
H	-1.151030593	1.90786349	3.399279511
C	2.472775692	0.621803911	-2.489029169
C	1.960033995	-4.350765194	-2.321078516
H	2.4876823	-3.904900127	-3.174398434
H	-0.402593053	2.955544038	4.606401978
H	3.726515771	0.725050115	-4.232961886
H	1.31231315	0.984059927	3.187102112
H	3.169792686	-4.124167722	0.411826135
C	2.158137908	-0.83754271	-2.697090754
H	2.704848967	-4.723648859	-1.610694559
H	1.223585039	-0.9089734	-3.268283626
H	2.458214035	-2.931605349	1.527975518
C	3.29894771	-3.129663179	0.851604475
H	2.941585492	-1.309352183	-3.303226453
C	3.453444666	-2.023577666	-0.21509947
H	3.068193978	-0.457144134	1.288508183
H	4.214257723	-3.161731235	1.459540908
C	3.819647066	-0.717815285	0.530019059
H	4.393698472	-3.271551472	-1.777810832
H	4.798950673	-1.545355417	-1.888260559
C	4.593056992	-2.368630685	-1.192180907
H	4.770196397	-0.868630885	1.06132638
H	3.957040749	0.132192223	-0.147836423
H	5.515032097	-2.544338982	-0.619695931
S	-0.816564167	-0.961908867	2.867696662
O	-0.210424595	-0.564461812	4.143750283
O	-2.090144747	-0.327907372	2.470174394
O	0.197832978	-1.030235567	1.711602926
C	-1.192602951	-2.780881252	3.058288959
F	-1.885001585	-3.231492734	1.987672163
F	-0.058406342	-3.486029316	3.152698292
F	-1.929525742	-2.976144244	4.151297191
C	-3.931394463	0.19703417	-3.081604478
N	-3.569179563	-0.398430242	-1.914690534

C	-4.408741031	-1.128772268	-1.132372825
C	-5.734563375	-1.261468017	-1.546444063
C	-6.154027294	-0.664231072	-2.734575046
C	-5.2483879	0.063234444	-3.510497831
H	-5.553789043	0.528041475	-4.443477529
H	-7.186311776	-0.770127745	-3.061490074
H	-6.421826834	-1.838148131	-0.934559048
C	-2.887678326	0.978518524	-3.824661441
H	-3.19855527	1.139721771	-4.861327447
H	-2.737341172	1.960427835	-3.355920907
H	-1.920576036	0.463507328	-3.813857881
C	-3.8494525	-1.755580275	0.103792165
H	-3.043449737	-2.454985026	-0.150714746
H	-3.413174818	-1.015230488	0.788513191
H	-4.629122761	-2.30810875	0.636055702
H	-2.56272041	-0.275656922	-1.589097802

Supplementary Table 47 | Cartesian coordinate of the transition state for protonation of XI. Units are presented in Å.

SCF energy (in toluene) = -3050.83593497 hartree

ZPE = 0.805643 hartree

imaginary frequency: 117*i*

Atom	X	Y	Z
Mo	-0.43292601	0.172783684	-0.166609727
P	-2.163636581	-1.610250788	0.553805765
N	-1.964768605	0.380300523	-1.812692517
P	0.183469921	2.408980752	-1.124911682
N	0.815608805	-0.727130542	-0.883942499
H	-2.429579563	-5.383104323	-0.218375699
H	-3.72697618	-4.184658909	-0.161947474
C	-2.741540606	-4.438331714	0.249026948
H	-2.425515238	-3.118570608	-2.116586097
H	-2.848801443	-4.628315363	1.321178928
H	-1.109459768	-4.282023567	-1.9301323
C	-1.48310014	-3.306880482	-1.586378673
H	-0.049997619	-4.763693527	0.257560166
C	-1.675507713	-3.367901779	-0.055402478
H	-4.187793512	-1.941296426	-0.885999078
H	-0.757167909	-2.541829505	-1.881844039
H	-4.190593889	-0.423507526	-0.012343022
C	-0.342222955	-3.764609274	0.611856392
H	-4.48595471	-3.362346727	1.874360381
H	0.458855334	-3.063036418	0.358353111
C	-3.551131669	-1.094367866	-0.603961358
H	-4.900468676	-0.891447991	-2.9150707
H	-0.415679366	-3.806372694	1.703502997
H	-5.183885137	-1.790563929	1.431984006
H	2.551405888	0.888140897	-1.940292453
C	-4.517422882	-2.302230555	2.137527999
H	3.762837278	3.169305825	-0.115173964
C	-3.991232984	-0.296229852	-2.931891169
H	-4.989415183	-2.227818424	3.127348269
C	-3.130169942	-0.332099454	-1.831080674
H	3.788886944	2.115442186	-2.2573098
H	2.655203175	1.904463894	0.456732939

C	2.70816529	1.919097468	-2.279191117
C	2.704027544	2.903856922	0.014713108
H	-4.356032591	0.561623292	-4.880011591
C	-3.119166784	-1.649668036	2.22683422
H	2.391798651	2.003214852	-3.326408141
C	-3.69152842	0.517328499	-4.019744199
H	2.275641907	3.605721751	0.735188155
H	-2.251456172	-3.463000915	3.118866203
H	-3.878698867	0.407773451	1.906786693
C	2.009897749	2.948096346	-1.362952873
C	-2.280255869	-2.381127707	3.294857504
C	-3.309934527	-0.173435518	2.647442029
H	-3.891782541	-0.148514699	3.579367815
H	3.222142156	4.543110298	-2.17330546
C	-2.534285716	1.289896318	-3.975825413
H	-1.252999651	-2.009865067	3.357893278
C	-1.684170592	1.206480812	-2.870612709
C	2.155218139	4.345735786	-1.997241124
H	1.646855775	4.42079686	-2.967155953
H	-2.745943384	-2.223946094	4.277406351
H	-2.276325948	1.953481495	-4.796930638
H	-2.3620693	0.330003779	2.853646888
H	0.635841759	5.0745809	0.489183611
C	-0.417354445	2.026641123	-2.858618997
H	1.783193393	5.144472974	-1.347644663
H	0.386987651	1.419211478	-3.29375939
H	0.045745556	3.742425328	1.513877134
C	-0.245426306	4.484788806	0.761038307
H	-0.533907691	2.907115054	-3.502100814
C	-0.921174728	3.830606088	-0.463728715
H	-2.072155307	2.471296304	0.83817459
H	-0.963014547	5.172345443	1.230048041
C	-2.243359306	3.19100918	0.024814873
H	-0.363781609	5.379462991	-1.939542481
H	-1.830757287	4.470030803	-2.36466371
C	-1.253621527	4.890676123	-1.531053527
H	-2.887689837	3.981694831	0.434755261
H	-2.801766662	2.702694338	-0.782024384
H	-1.875615254	5.672823095	-1.072934426
S	0.560910643	0.263348439	3.087109605
O	-0.435729719	0.55147207	4.124447147
O	1.045930351	-1.11715813	2.922336086
O	0.209087144	0.929032611	1.727926047
C	2.053724433	1.291423026	3.538450324
F	3.076767008	1.018895134	2.70661543
F	1.761575784	2.596515906	3.443660494
F	2.423992179	1.009301415	4.785673721

C	3.110127496	-2.711236714	-2.640474017
N	2.978440007	-2.131082245	-1.425608131
C	3.901877422	-2.323327855	-0.454926314
C	5.011186625	-3.14352492	-0.690639454
C	5.161850116	-3.752958005	-1.934095858
C	4.203788427	-3.531716818	-2.923968351
H	4.296186071	-3.988071819	-3.906176099
H	6.01943071	-4.39275194	-2.132743063
H	5.742950821	-3.293031158	0.098935938
C	2.034403495	-2.43864644	-3.660599533
H	2.299865549	-2.867141771	-4.632349638
H	1.075810955	-2.873073511	-3.347101273
H	1.878120406	-1.359892855	-3.78937099
C	3.701973282	-1.611388944	0.854020603
H	3.810903118	-0.52667406	0.723495983
H	2.704350671	-1.780803516	1.276084459
H	4.444978389	-1.935016426	1.589983449
H	1.773293139	-1.347993706	-1.128198114

Supplementary Table 48 | Cartesian coordinate of the product complex for protonation of XI. Units are presented in Å.

SCF energy (in toluene) = -3050.83838305 hartree

ZPE = 0.806890 hartree

Atom	X	Y	Z
Mo	-0.487588874	0.042074193	-0.106607041
P	-1.449439632	-2.277831768	0.523800724
N	-2.046256271	-0.22265206	-1.717345387
P	-0.732831082	2.400121757	-0.924097019
N	0.969405829	-0.312715066	-0.90629951
H	-0.422424807	-5.862204588	-0.459432505
H	-2.050085219	-5.184940452	-0.343132865
C	-1.031719522	-5.108195935	0.058776713
H	-1.207429439	-3.619763282	-2.233936794
H	-1.052856568	-5.382782397	1.117820363
H	0.419550812	-4.291896421	-2.095237898
C	-0.25293835	-3.516293012	-1.702145166
H	1.615378782	-4.482591704	0.048513197
C	-0.398313462	-3.723480519	-0.179185511
H	-3.268067105	-3.231540156	-0.914215027
H	0.180006859	-2.540240298	-1.944799699
H	-3.783315473	-1.864438497	0.050340385
C	1.000136003	-3.675579774	0.471494842
H	-3.011804406	-4.758382527	1.762070307
H	1.501490877	-2.722483471	0.276007768
C	-2.964870375	-2.229891862	-0.586680842
H	-4.366638915	-2.399472962	-2.868716008
H	0.963596032	-3.816157508	1.556361867
H	-4.211949636	-3.486301354	1.45517297
H	2.023326696	1.885143037	-1.822163619
C	-3.38101944	-3.788444184	2.104198922
H	2.356161327	4.403734907	0.032033905
C	-3.731685845	-1.517400292	-2.856841344
H	-3.803465758	-3.937839921	3.107789893
C	-2.88013826	-1.303259216	-1.769026587
H	2.692182555	3.493616235	-2.135827513
H	1.810430399	2.813795176	0.598635442
C	1.765638517	2.903420364	-2.135872579
C	1.472596607	3.767788186	0.184329589

H	-4.443882602	-0.737835765	-4.740717842
C	-2.27522836	-2.715036635	2.208889782
H	1.407443419	2.878680954	-3.172618229
C	-3.779365147	-0.58950506	-3.892205239
H	0.845197902	4.255721983	0.934808048
H	-0.830944899	-4.184644544	2.979778477
H	-3.686480949	-1.017238359	2.025369026
C	0.763750369	3.578529183	-1.173256086
C	-1.206169874	-3.182276987	3.218101657
C	-2.922939084	-1.405314905	2.715781306
H	-3.434243624	-1.611438784	3.666314796
H	1.268284884	5.52679641	-1.962366848
C	-2.974461355	0.543479884	-3.810055048
H	-0.355297183	-2.498497492	3.28411476
C	-2.114202534	0.708305558	-2.722057809
C	0.358194186	4.942731599	-1.766020816
H	-0.174793059	4.843106298	-2.720647748
H	-1.663165481	-3.235024643	4.21574418
H	-2.998586775	1.300728094	-4.589211235
H	-2.184993427	-0.623650651	2.913663731
H	-1.218271134	4.984147314	0.809941968
C	-1.223329983	1.924580564	-2.670323835
H	-0.261442234	5.531678497	-1.081958751
H	-0.272549644	1.672375776	-3.157892981
H	-1.218476033	3.490135565	1.781348803
C	-1.802734298	4.09889016	1.080419212
H	-1.669609134	2.740666998	-3.251235052
C	-2.250432354	3.290349232	-0.156169515
H	-2.758916917	1.549017562	1.098267283
H	-2.698047603	4.451263247	1.611479476
C	-3.221572384	2.190057061	0.334212119
H	-2.364766872	4.989696839	-1.56445049
H	-3.421291317	3.624600824	-1.989606275
C	-2.996467295	4.195817418	-1.154278359
H	-4.088864042	2.669141374	0.810209114
H	-3.60266621	1.567026703	-0.482922362
H	-3.836071579	4.679217615	-0.635080332
S	0.593599231	0.22627369	3.095204394
O	-0.33633815	0.111553389	4.22331046
O	1.463772527	-0.914108337	2.76117136
O	-0.085679439	0.835394957	1.835509176
C	1.731992114	1.642085874	3.528619612
F	2.694946888	1.764264932	2.594461274
F	1.042456604	2.789128758	3.585227975
F	2.302314729	1.40615207	4.708015492
C	3.70909668	-1.27450467	-2.960461609
N	3.480070701	-0.873582653	-1.690200385

C	4.478887491	-0.862647394	-0.77858559
C	5.767751508	-1.281601508	-1.130354456
C	6.018286644	-1.70308325	-2.43416428
C	4.978354785	-1.695873861	-3.364133304
H	5.141992717	-2.012401288	-4.391187821
H	7.014299916	-2.030931328	-2.725396919
H	6.557717548	-1.269365996	-0.383890371
C	2.542650955	-1.246634681	-3.915967745
H	2.858688545	-1.509857856	-4.930627123
H	1.763071306	-1.955914441	-3.607464441
H	2.084800274	-0.249163652	-3.945985989
C	4.157204147	-0.362123066	0.603393183
H	3.910614311	0.707360455	0.577840184
H	3.293857384	-0.875413547	1.043748632
H	5.012341653	-0.491967259	1.274672846
H	2.023651307	-0.526018499	-1.234353702

Supplementary Table 49 | Cartesian coordinate of the reactant complex for coordination of dinitrogen to XII. Units are presented in Å.

SCF energy (in toluene) = -2833.65797456 hartree

ZPE = 0.667056 hartree

Atom	X	Y	Z
Mo	-0.098363812	0.589512026	0.342224076
P	0.447304354	-0.857238664	2.399874949
N	1.94945628	1.097831266	0.76690574
P	0.460438132	2.027091404	-1.665961838
N	-1.357752767	1.547878619	1.113655134
H	-0.881831866	-1.768744088	5.921656369
H	0.659357657	-1.013673703	5.49114931
C	-0.124800964	-1.690720389	5.126984971
H	-0.839075639	0.935136494	4.5380186
H	0.311594621	-2.686949227	4.999239898
H	-2.247898814	-0.036424278	4.972344426
C	-1.517414562	0.154216585	4.172870151
H	-2.658755125	-2.232136419	4.17117396
C	-0.803321352	-1.173760206	3.841903396
H	0.846880086	1.278208715	3.421057713
H	-2.069506828	0.532106785	3.307493891
H	2.117732464	0.189457806	4.002539399
C	-1.89272615	-2.165726987	3.383884735
H	2.356549349	-2.401290297	4.31481073
H	-2.372587287	-1.83983158	2.45671613
C	1.548306691	0.489637438	3.115361041
H	4.080970125	1.398082087	3.400869112
H	-1.506141198	-3.172857405	3.212470015
H	3.426915804	-1.519189429	3.202508968
H	-0.036262467	3.705539473	0.792251666
C	2.753513504	-2.379786168	3.294771355
H	-1.109336695	5.491657739	-1.354103022
C	3.744446632	1.467664959	2.3685446
H	3.365099052	-3.284418061	3.162090036
C	2.463513534	1.032806267	2.057564718
H	0.614294466	5.290965537	0.3067107
H	-1.787337441	3.87208149	-1.098682738
C	0.683378366	4.205718586	0.138227875
C	-1.032031036	4.437544304	-1.659118219

H	5.587852551	2.338194583	1.612522793
C	1.654350903	-2.368092048	2.211734419
H	1.690995526	3.888898487	0.42834763
C	4.584801684	1.992271163	1.377630306
H	-1.283245644	4.388195263	-2.724245095
H	0.504867333	-3.947419898	3.238146267
H	2.915130567	-1.319373662	0.732622011
C	0.392036035	3.928532392	-1.353758047
C	0.887414359	-3.706201856	2.239879059
C	2.347147531	-2.250262239	0.834386939
H	3.057085088	-3.084815536	0.732456376
H	1.332237567	5.782720401	-1.952045562
C	4.085022312	2.043161279	0.070845073
H	0.055554155	-3.724450606	1.528420057
C	2.802957217	1.596429719	-0.215877972
C	1.435048678	4.711340468	-2.179359063
H	2.459738497	4.424599021	-1.910794184
H	1.582119114	-4.510018612	1.955588268
H	4.699599933	2.420879618	-0.743937365
H	1.635373776	-2.329453607	0.006905275
H	-2.157490361	2.367126713	-3.147717874
C	2.282062211	1.584517868	-1.626117577
H	1.316152769	4.593676025	-3.259164529
H	2.905042454	2.202314146	-2.283564292
H	-1.948270723	0.662113692	-2.701943168
C	-1.646106587	1.435124871	-3.414731308
H	2.319762029	0.555834794	-2.014689259
C	-0.110341654	1.599415471	-3.459701566
H	0.364826877	-0.525782771	-3.089983632
H	-2.00461652	1.135409458	-4.41055899
C	0.520532772	0.243374222	-3.851336888
H	-0.221313109	3.593027122	-4.409883839
H	1.354092416	2.786843254	-4.603668739
C	0.270206737	2.623700032	-4.545455588
H	0.041271696	-0.107857163	-4.776352046
H	1.594121624	0.336989762	-4.059684211
H	-0.051432327	2.232475766	-5.522292052
S	-1.166219253	-2.442028051	-0.971121596
O	-0.168233994	-3.4519462	-1.35571261
O	-1.967298603	-2.675501309	0.241914767
O	-0.617618147	-1.006729539	-1.077746593
C	-2.400105277	-2.40559762	-2.369676441
F	-3.320299135	-1.449796826	-2.159874514
F	-1.783819256	-2.153373326	-3.536519827
F	-3.018310394	-3.588869047	-2.45409848
N	-4.752720167	-0.266405128	0.235804597
N	-4.455154026	0.187694818	1.201045521

H	-1.817341005	2.047980795	1.883695759

Supplementary Table 50 | Cartesian coordinate of the transition state for coordination of dinitrogen to XII. Units are presented in Å.

SCF energy (in toluene) = -2833.65137694 hartree

ZPE = 0.667834 hartree

imaginary frequency: 78*i*

Atom	X	Y	Z
Mo	-0.180974764	0.62120118	0.392570568
P	0.386508949	-0.87039605	2.432900129
N	1.87792231	1.063713035	0.736695598
P	0.356645565	2.051864656	-1.647176161
N	-1.068403126	1.8299693	1.350514135
H	-1.067824633	-1.786848316	5.908143398
H	0.525066683	-1.112431274	5.535101021
C	-0.285894746	-1.735767647	5.135814497
H	-0.833556838	0.89103199	4.701679244
H	0.096050463	-2.751908672	4.994651866
H	-2.365057115	0.033791895	4.90253165
C	-1.536943674	0.212344754	4.201434394
H	-2.788022618	-2.168176146	4.10005513
C	-0.896286216	-1.147454703	3.84841496
H	0.801349059	1.291628146	3.392461234
H	-1.943130838	0.714582699	3.316945971
H	2.07318505	0.215878365	3.99380788
C	-2.01430726	-2.073384684	3.323307671
H	2.180793361	-2.446897871	4.444627409
H	-2.480782171	-1.673177358	2.418156028
C	1.496582084	0.494369703	3.104289732
H	4.074878994	1.287644433	3.321111304
H	-1.655877727	-3.077459293	3.082973156
H	3.314379386	-1.551425095	3.407542936
H	0.4119794	3.661538912	0.852967774
C	2.632257139	-2.409230512	3.448444371
H	-1.123821868	5.517132802	-1.103259841
C	3.719366981	1.361471801	2.295672411
H	3.24848338	-3.313295385	3.334608797
C	2.407212162	0.992557984	2.017242283
H	0.814344273	5.303205884	0.298386697
H	-1.722525719	3.899991506	-0.671537104

C	0.933419874	4.23115603	0.081219864
C	-1.091646565	4.452814657	-1.379197412
H	5.590802806	2.104101014	1.485927331
C	1.597990319	-2.381045219	2.301978284
H	2.002369287	3.998787997	0.147301131
C	4.563130523	1.816624882	1.27896572
H	-1.532675342	4.361563018	-2.378916725
H	0.375061327	-3.94876765	3.255724746
H	3.00361687	-1.351679837	0.949093274
C	0.367755268	3.956432694	-1.330610315
C	0.82066452	-3.713778859	2.282254122
C	2.380274952	-2.25221898	0.974093229
H	3.050887394	-3.119745746	0.885065312
H	1.282007216	5.804155743	-1.975530019
C	4.038668679	1.884344057	-0.015597538
H	0.033425511	-3.728709813	1.523020281
C	2.723127712	1.51280726	-0.265891416
C	1.244130233	4.759076633	-2.316756298
H	2.279240687	4.394157057	-2.338430198
H	1.52590122	-4.522914739	2.043384935
H	4.653564075	2.220753703	-0.847574619
H	1.725797185	-2.257079265	0.099387457
H	-2.338708842	2.292172928	-2.931557407
C	2.16327265	1.548445096	-1.661466143
H	0.860538683	4.766054049	-3.337890766
H	2.796563829	2.153585421	-2.321747966
H	-2.022555203	0.573197835	-2.622211781
C	-1.805347067	1.405534033	-3.296968836
H	2.155317316	0.524719241	-2.061406353
C	-0.285875505	1.648498774	-3.428207052
H	0.330716473	-0.454458779	-3.160895809
H	-2.217321333	1.156363466	-4.286038191
C	0.391298516	0.34432994	-3.904485433
H	-0.594833582	3.64661176	-4.320769243
H	1.023159486	2.963321722	-4.623802622
C	-0.039954199	2.72030817	-4.5064251
H	-0.126331759	-0.006635449	-4.808498863
H	1.442650662	0.507956382	-4.175258632
H	-0.39125753	2.324078886	-5.470849202
S	-0.885373245	-2.570130071	-1.020013867
O	0.137528402	-3.581626342	-1.326360195
O	-1.770286049	-2.804613301	0.136733045
O	-0.337480873	-1.144110039	-1.081655875
C	-2.033057546	-2.600874473	-2.4915284
F	-3.003579493	-1.675844306	-2.361688091
F	-1.362899079	-2.351256969	-3.62900986
F	-2.606225764	-3.805783034	-2.586135966

N	-3.046455975	0.0422315	0.048373638
N	-4.133412639	0.12750276	0.247774175
H	-1.039124668	2.612776969	2.020340571

Supplementary Table 51 | Cartesian coordinate of the reactant complex for protonation of XIV. Units are presented in Å.

SCF energy (in toluene) = -3161.50806660 hartree

ZPE = 0.842892 hartree

Atom	X	Y	Z
Mo	-0.591757089	0.540795729	0.610906259
P	0.050652003	-0.99675011	2.552221543
N	1.521457456	1.150166406	1.00062542
P	-0.061602743	2.398500607	-1.240937682
H	-1.200459949	-2.133586295	6.038292077
H	0.30078494	-1.287225343	5.645453757
C	-0.447999906	-1.98643249	5.250378351
H	-1.302218141	0.614794917	4.77579524
H	0.033661224	-2.955207106	5.085947351
H	-2.647534305	-0.445310288	5.179517955
C	-1.94260665	-0.184840516	4.378368106
H	-2.944627172	-2.638858162	4.253042335
C	-1.153684874	-1.454799509	3.986697919
H	0.441128321	1.155936939	3.664321076
H	-2.542329152	0.196841909	3.54497426
H	1.703374679	0.041260026	4.166349674
C	-2.176140247	-2.498540923	3.47975008
H	2.012594472	-2.517295582	4.41223417
H	-2.675731061	-2.179990906	2.560314811
C	1.127523425	0.381533931	3.29971111
H	3.749902696	1.111737086	3.549226819
H	-1.721958788	-3.473167403	3.289956092
H	3.087074181	-1.556106501	3.370683645
H	0.591228984	3.756881286	1.316477053
C	2.432397934	-2.434920953	3.406001778
H	-1.205389834	5.943846513	-0.37629213
C	3.373904993	1.263385248	2.540832509
H	3.074417488	-3.31298885	3.24907875
C	2.042590711	0.955995932	2.251763696
H	0.788104939	5.462120248	0.883015913
H	-1.876857063	4.384331648	0.128642827
C	0.908848032	4.432573654	0.518365347
C	-1.286677512	4.881882662	-0.650145756
H	5.247424138	1.977487583	1.735333574

C	1.367126256	-2.416346573	2.288431291
H	1.981855514	4.264365913	0.37534091
C	4.204121989	1.746756549	1.531541774
H	-1.861670866	4.833066188	-1.582311663
H	0.293297048	-4.110690069	3.203431843
H	2.651132703	-1.240718355	0.92573857
C	0.126465402	4.282830317	-0.809902619
C	0.667986709	-3.790366818	2.225516911
C	2.10121117	-2.187047741	0.948521515
H	2.84028334	-2.991570907	0.826175332
H	1.183063336	6.082988364	-1.371332193
C	3.674709224	1.925878089	0.254223518
H	-0.154002798	-3.808091186	1.503217725
C	2.329584369	1.638727671	0.012155594
C	0.924778061	5.121096593	-1.837081408
H	1.870035422	4.645323943	-2.127614243
H	1.409259118	-4.536433065	1.90667345
H	4.29559673	2.288507143	-0.560485141
H	1.438323121	-2.231611834	0.081152984
H	-2.803712242	2.743318477	-2.442094553
C	1.726408388	1.822027689	-1.352159341
H	0.361401451	5.346205844	-2.74198757
H	2.362141249	2.468259937	-1.969255028
H	-2.453962311	1.014752068	-2.248004524
C	-2.268774161	1.888861684	-2.877255079
H	1.679014915	0.843159526	-1.847640368
C	-0.763641688	2.188893597	-3.043298034
H	-0.063065903	0.089796726	-3.135549278
H	-2.711353817	1.696248178	-3.864685906
C	-0.057307318	1.001504457	-3.73415782
H	-1.172821091	4.280423093	-3.640224811
H	0.42168709	3.678252949	-4.166613444
C	-0.621971745	3.399532966	-3.986295686
H	-0.584977398	0.785400589	-4.673367719
H	0.978401185	1.249900599	-3.998564716
H	-1.052608562	3.116973608	-4.957485177
S	-1.053856625	-2.188264675	-1.185703006
O	0.0003516	-2.952027685	-1.851606366
O	-1.674112309	-2.688697658	0.047398792
O	-0.560013347	-0.675239704	-1.053070242
C	-2.455791647	-2.022865209	-2.418153833
F	-3.405211227	-1.213666185	-1.923820596
F	-2.005675376	-1.517450921	-3.569980345
F	-2.969729334	-3.23113778	-2.62849271
N	-2.563408531	0.018026103	0.81657294
N	-3.645499389	-0.242441875	0.997188664
N	-1.43218539	2.220119465	1.931231594

H	-0.81129501	3.024891788	2.024198432
H	-1.605264192	1.883893709	2.881521027
C	-5.130087821	3.750826582	1.033542335
N	-4.036430609	3.870847176	1.817168233
C	-4.044855353	4.778420213	2.818805216
C	-5.150676063	5.5975835	3.069573927
C	-6.279725016	5.477536634	2.260348856
C	-6.269252068	4.543648784	1.227770831
H	-7.129918126	4.422056931	0.574467884
H	-7.152432728	6.105054423	2.431398249
H	-5.120968683	6.316856514	3.88431568
C	-2.801303885	4.883262322	3.670214896
H	-2.91237147	5.64921674	4.445076365
H	-2.585268722	3.930194955	4.173211238
H	-1.925963499	5.14800678	3.060300846
C	-5.087346235	2.713120156	-0.058167018
H	-5.894446926	2.862238044	-0.783756573
H	-4.128308669	2.747400638	-0.589353396
H	-5.191691727	1.701725206	0.357908227
H	-2.345988924	2.634430897	1.648508596

Supplementary Table 52 | Cartesian coordinate of the transition state for protonation of XIV. Units are presented in Å.

SCF energy (in toluene) = -3161.48814983 hartree

ZPE = 0.840082 hartree

imaginary frequency: 361*i*

Atom	X	Y	Z
Mo	-0.670013889	0.730333195	0.634886974
P	-0.027769099	-0.830962002	2.57640244
N	1.49234963	1.232271616	0.948777458
P	-0.062980416	2.442456158	-1.333781766
H	-1.30016617	-1.875439944	6.089938372
H	0.228737842	-1.088029796	5.676183721
C	-0.545701416	-1.768037153	5.297004606
H	-1.310421077	0.843143626	4.787645543
H	-0.09746535	-2.755359196	5.147779516
H	-2.706216085	-0.159971383	5.187022319
C	-1.980557308	0.066509109	4.39318215
H	-3.065868197	-2.353753492	4.304343715
C	-1.234253149	-1.235019246	4.0252004
H	0.439684909	1.330627619	3.621971825
H	-2.544109537	0.462612144	3.541479413
H	1.685658324	0.197059698	4.144869828
C	-2.289687055	-2.251505942	3.531902646
H	1.887897011	-2.387634889	4.456999142
H	-2.772172968	-1.931045042	2.604013114
C	1.103202784	0.528272224	3.278749395
H	3.746365381	1.160426133	3.475074138
H	-1.866822873	-3.243457146	3.359633923
H	2.990701464	-1.475288632	3.401642638
H	0.657801662	3.865391421	1.147939529
C	2.308554355	-2.332426553	3.449219359
H	-1.094958609	6.047093075	-0.598385608
C	3.365890287	1.304541903	2.467191086
H	2.922202526	-3.232557679	3.303439611
C	2.018721093	1.045686049	2.19960361
H	0.913795261	5.550475868	0.658219963
H	-1.757143532	4.516298373	0.00965459
C	0.995013332	4.504761498	0.328296619

C	-1.200566272	4.974365579	-0.817181766
H	5.25885012	1.922077591	1.628585303
C	1.242987847	-2.29614213	2.332012506
H	2.060081573	4.296638303	0.178430421
C	4.205276594	1.72847242	1.439506774
H	-1.808867343	4.885321622	-1.725672634
H	0.124180085	-3.946013581	3.274044613
H	2.561656799	-1.180715409	0.953000348
C	0.193677095	4.336883217	-0.986489545
C	0.503084818	-3.649484141	2.289918166
C	1.986041967	-2.111020105	0.989943275
H	2.703047651	-2.937869383	0.885271478
H	1.29766679	6.08626752	-1.617300988
C	3.671125384	1.892403588	0.161635308
H	-0.321965183	-3.650955495	1.57120572
C	2.313842606	1.651475107	-0.057644497
C	0.994351277	5.122410673	-2.051519072
H	1.914839443	4.605355743	-2.351258661
H	1.220849269	-4.420926477	1.977325028
H	4.29799486	2.206143591	-0.669030326
H	1.325181742	-2.153338468	0.121414431
H	-2.783520922	2.79448643	-2.63967392
C	1.704687533	1.80621195	-1.421578119
H	0.418371043	5.34374037	-2.950035844
H	2.356020621	2.40532109	-2.069520333
H	-2.460615511	1.075447284	-2.321918321
C	-2.2502407	1.905206279	-3.001191672
H	1.621521262	0.809223674	-1.872707187
C	-0.738005683	2.175189945	-3.144324187
H	-0.084482481	0.060579866	-3.13960123
H	-2.669570705	1.652934416	-3.985469194
C	-0.047938515	0.945849332	-3.776255073
H	-1.097649892	4.248056404	-3.830906257
H	0.492778946	3.596489724	-4.306108932
C	-0.558606917	3.343432123	-4.133020692
H	-0.567593427	0.702551288	-4.713262697
H	0.996588227	1.160057378	-4.035407827
H	-0.978396826	3.031060038	-5.100076462
S	-1.153682804	-2.089511462	-1.11468473
O	-0.131179722	-2.920858924	-1.753752491
O	-1.804505711	-2.548830494	0.121077381
O	-0.630508692	-0.609491868	-1.020848854
C	-2.542779193	-1.939494639	-2.359410969
F	-3.484856014	-1.093889346	-1.905176721
F	-2.081656272	-1.481604727	-3.529434273
F	-3.08661	-3.141680633	-2.537512688
N	-2.638226393	0.211690337	0.801881195

N	-3.719755097	-0.054442225	0.994929608
N	-1.404377736	2.367735252	1.834785308
H	-0.80966558	3.197710848	1.831416127
H	-1.467558398	2.060747353	2.80830082
C	-4.791504536	3.526880425	1.052472431
N	-3.829696385	3.543705078	2.008009354
C	-4.00232223	4.167358746	3.206279925
C	-5.207002534	4.809516616	3.475319277
C	-6.221795947	4.806007385	2.515042046
C	-6.010749861	4.165441733	1.298760886
H	-6.778700059	4.150533209	0.529949225
H	-7.167241549	5.305074401	2.716476204
H	-5.342397765	5.308842384	4.430572268
C	-2.875151409	4.135581677	4.20237902
H	-3.107502659	4.780846523	5.054824326
H	-2.713567163	3.119117385	4.585883978
H	-1.934030459	4.481012807	3.757701409
C	-4.521514293	2.813655624	-0.237793481
H	-4.883416349	3.411302838	-1.08267616
H	-3.458540892	2.603666085	-0.374583128
H	-5.05341453	1.853233706	-0.257006893
H	-2.814259351	3.007278402	1.825859164

Supplementary Table 53 | Cartesian coordinate of the product complex for protonation of XIV. Units are presented in Å.

```
SCF energy (in toluene) = -3161.50809391 hartree

ZPE = 0.843324 hartree

--------------------------------------------------

 Atom            Coordinates (Angstroms)

            X              Y               Z

--------------------------------------------------

 Mo    -0.551514869   0.641235399   0.569535989
  P     0.042143238  -0.845332904   2.561690192
  N     1.589756008   1.179481236    0.91626541
  P     0.027560862   2.407821275  -1.351935219
  H    -1.248149996  -1.788587626   6.091914379
  H      0.29589136  -1.042074721   5.662619932
  C    -0.493809559  -1.711128781   5.295851469
  H    -1.194616175   0.908076776   4.751612108
  H    -0.067705868   -2.71026552   5.160861024
  H     -2.61635942  -0.047380241   5.159109367
  C    -1.883948598    0.14536774   4.363148241
  H    -3.027051101  -2.258770167   4.327872059
  C    -1.175601389  -1.183430904   4.017826894
  H     0.559918182   1.320655783   3.604286002
  H    -2.438380315   0.549932006   3.509886292
  H       1.7729373   0.159506907   4.112431417
  C    -2.255310815  -2.185164459   3.548361856
  H     1.932458452  -2.404872128   4.469270737
  H    -2.740587039  -1.870219648   2.620286378
  C     1.198245591   0.502598031   3.246086117
  H     3.850509321   1.119943156   3.436561506
  H    -1.853637285  -3.188111324   3.388862061
  H     3.052553952  -1.535113404   3.395408901
  H     0.777059772   3.796048874   1.157082197
  C     2.351666442    -2.3760736   3.459809527
  H    -1.005863413   6.005566607   -0.56140065
  C     3.467520558    1.25867944   2.428981079
  H     2.945816504  -3.291362944    3.32907678
  C      2.12060687   1.000719997   2.166155008
  H     0.987055282   5.492935027   0.689497013
  H    -1.723761771   4.477475984  -0.025660587
  C     1.082949899   4.455377228   0.340273924
  C    -1.119547992   4.941902684  -0.815297673
  H      5.35803523   1.871658586   1.580419005
```

C	1.284334817	-2.338005143	2.344488455
H	2.149838465	4.2681139	0.175937841
C	4.303564232	1.678875593	1.396265708
H	-1.701399481	4.890743678	-1.742963741
H	0.131149923	-3.946981583	3.316735455
H	2.608914941	-1.262700034	0.938470917
C	0.274529235	4.296291679	-0.973071653
C	0.516207072	-3.676190293	2.327621819
C	2.025281347	-2.187321884	0.997240281
H	2.73443451	-3.022384116	0.906477027
H	1.377048205	6.049852733	-1.593607599
C	3.763596864	1.844329236	0.121555685
H	-0.309316147	-3.673952774	1.609451821
C	2.405238574	1.605296897	-0.095325104
C	1.080103169	5.086426619	-2.032465619
H	2.004123003	4.573420788	-2.327993177
H	1.217169418	-4.468109197	2.028644459
H	4.387522178	2.158415578	-0.711115648
H	1.360802675	-2.241666894	0.131902344
H	-2.71342639	2.801251488	-2.53599041
C	1.794303236	1.771783164	-1.458519283
H	0.507977398	5.308322879	-2.932927967
H	2.44552699	2.37799652	-2.099660182
H	-2.415418572	1.067517835	-2.307980825
C	-2.208324016	1.921938071	-2.956764721
H	1.708293885	0.781414033	-1.924573912
C	-0.695480244	2.167874374	-3.141140684
H	-0.062011625	0.046089467	-3.167083705
H	-2.66422963	1.721866939	-3.936657347
C	-0.034545477	0.935674633	-3.797811237
H	-1.045163539	4.249902364	-3.806136652
H	0.53126597	3.585840682	-4.312229398
C	-0.519814068	3.342756993	-4.123026756
H	-0.580250091	0.70369874	-4.722701379
H	1.005047151	1.14192921	-4.082332602
H	-0.95910788	3.041006649	-5.084501674
S	-1.123429613	-2.132674711	-1.135000969
O	-0.100693807	-2.961610728	-1.771969642
O	-1.76548885	-2.564408177	0.112467689
O	-0.569054859	-0.638674194	-1.053005132
C	-2.516432766	-1.961533448	-2.376700872
F	-3.442847966	-1.109182637	-1.912052641
F	-2.048935648	-1.506568687	-3.542611698
F	-3.066511689	-3.159655353	-2.550937968
N	-2.545714215	0.190098449	0.780825026
N	-3.637627315	-0.028496199	0.952600532
N	-1.333596341	2.411863694	1.786075917

H	-0.87614645	3.297331736	1.569146524
H	-1.194753004	2.261970987	2.788604514
C	-5.14114145	3.632799449	1.118339645
N	-4.144618436	3.585138495	2.026986241
C	-4.315497759	4.197542909	3.220231851
C	-5.492827944	4.878929255	3.542722507
C	-6.523836001	4.931250338	2.604500537
C	-6.345608831	4.302529208	1.376067232
H	-7.124863338	4.323319067	0.617782264
H	-7.449506208	5.45775665	2.82882413
H	-5.59382778	5.361540305	4.511877361
C	-3.175348263	4.120032966	4.208167977
H	-3.401271787	4.681061341	5.12109012
H	-2.976111166	3.078429778	4.49761385
H	-2.252156285	4.535042135	3.780202248
C	-4.929230865	2.913566517	-0.189027169
H	-5.527696986	3.360241181	-0.991390517
H	-3.873879666	2.93463082	-0.481862752
H	-5.223255556	1.858265711	-0.10264844
H	-2.353931748	2.622843413	1.708400136

Supplementary Table 54 | Cartesian coordinate of the transition state for elimination of the ammonia ligand from XVII. Units are presented in Å.

SCF energy (in toluene) = -3909.60899623 hartree

ZPE = 1.310831 hartree

imaginary frequency: 51*i*

Atom	X	Y	Z
Mo	-2.58903079	0.070012599	-0.046776179
Mo	2.657553379	0.023549409	0.171806494
P	3.271924049	2.116711781	-1.055686633
P	-3.231564893	-2.073008172	-1.228988968
N	-4.82580549	0.157140233	0.003526994
N	4.876214688	-0.116770939	0.130922574
P	3.042692367	-2.163790721	1.328428754
P	-3.00736197	2.259788254	1.176122604
N	-0.519990422	0.035574895	-0.03269409
N	0.636391075	0.035272928	0.041643107
N	-2.558952102	1.552876705	-2.844890528
N	-2.568482667	1.011030631	-1.843112597
N	-2.574491809	-0.879256747	1.751514672
N	-2.571196262	-1.427712257	2.747842661
N	2.688595597	0.921893736	1.886163761
N	2.726768769	1.47321419	2.895008633
H	2.622580609	3.542367364	-4.637799778
H	-2.720890414	-3.587575148	-4.800772257
H	-4.064647941	-2.587499828	-4.242867676
H	4.016461019	2.595977661	-4.106038556
C	3.244282922	3.296417285	-3.76290893
C	-3.308011695	-3.309472309	-3.911728748
H	-2.368610442	-0.739242746	-3.863118099
H	2.45233542	0.640904965	-3.479113081
H	-3.823522704	-4.210715545	-3.569017706
H	3.74002036	4.218181787	-3.446808565
H	-1.081893721	-1.838726017	-4.373502926
H	1.173560018	1.658744356	-4.179638432
C	1.703699687	1.406709068	-3.248279961
C	-1.660331657	-1.49191432	-3.502646953
H	0.558562328	3.788272559	-3.185595421
H	-0.639992111	-3.915178425	-3.381138682

C	2.349431984	2.679647552	-2.667960674
C	-2.359803577	-2.703816645	-2.85528689
H	-4.541143798	-0.755055885	-2.757383008
H	4.544843872	0.795257832	-2.614042557
H	0.980423745	0.972201956	-2.549609369
H	-0.979431863	-0.993804347	-2.802822538
H	5.549235701	2.192876369	-2.205967484
H	-5.531091403	-2.153173948	-2.322704167
C	1.211424672	3.654688621	-2.310117118
C	-1.261286425	-3.722878201	-2.49287733
H	3.609060386	5.290916456	-1.61494054
H	-3.647887178	-5.259779345	-1.728921494
H	0.590130563	3.265265537	-1.493928662
H	-0.60283414	-3.342771926	-1.700986487
C	4.881495718	1.431575536	-1.781098256
C	-4.855651049	-1.389367394	-1.915640424
H	7.560358144	1.085737477	-1.560473742
H	-7.531744074	-1.074948599	-1.624879162
H	1.579939054	4.646018179	-2.023357312
H	-1.664477849	-4.687476101	-2.16807122
H	5.208737654	4.514296709	-1.640111614
H	-5.227532817	-4.444288415	-1.713069949
H	-4.48034249	2.615456874	-1.359932006
H	4.229839451	-2.475326926	-1.351672058
C	4.386172934	4.838934926	-0.989024417
C	-4.391761841	-4.779098903	-1.08484066
H	-2.619987042	5.179919767	-1.283207016
H	2.414765438	-4.857800632	-1.300759149
C	7.017506806	0.511019655	-0.812926764
C	-6.976847184	-0.501936039	-0.88516363
H	4.783148438	5.636559399	-0.342289727
H	-4.786251325	-5.557533921	-0.41375997
C	5.624243561	0.581812537	-0.784410826
C	-5.581646669	-0.545464095	-0.898445099
H	-4.862550572	4.342530447	-1.428717088
H	4.67832562	-4.192509586	-1.451115764
H	-2.008234749	3.522733326	-1.386835067
H	1.720465499	-3.244009871	-0.995615805
C	-4.683628171	3.496842001	-0.747532787
C	4.521658573	-3.349470412	-0.759288524
C	-2.316633606	4.304703558	-0.6880401
C	2.162834093	-4.125390022	-0.51699184
H	-8.729382274	0.304977203	0.098218821
H	8.783393158	-0.349162284	0.101324361
C	3.850027159	3.699879404	-0.100347817
C	-3.804675282	-3.637793764	-0.230219389
H	-5.613050971	3.323850358	-0.19265639

H	5.485511333	-3.138004078	-0.283004344
C	-7.642570335	0.263509535	0.071894452
C	7.697351232	-0.286211613	0.107883853
H	-1.445270624	4.597923451	-0.095054318
H	1.39535377	-4.579776591	0.116544811
H	1.829235895	4.562114784	0.138071885
H	-1.801131887	-4.553344186	-0.034387147
H	5.895342681	2.986254111	0.323342691
H	-5.841417638	-2.93296865	0.240264478
C	-3.507024461	3.85645552	0.187640896
C	3.436389702	-3.743641277	0.267193349
C	2.673826762	4.227813277	0.749479271
C	-2.616793798	-4.181973842	0.593505485
C	4.987273145	3.285581098	0.860377313
C	-4.911306102	-3.199066883	0.755657777
H	5.248064938	4.151551739	1.487894578
H	-5.137943219	-4.04199953	1.425748986
H	-4.337440531	5.842967832	0.395217227
H	4.235663313	-5.757987761	0.335132187
C	6.940989675	-0.999124732	1.039730353
C	-6.875031122	0.974309089	0.994079183
H	2.315298904	3.471951294	1.454006798
H	-2.209174926	-3.426215125	1.270264594
C	5.549440136	-0.903354539	1.034085676
C	-5.481294461	0.912453738	0.94091637
C	-3.966986551	5.044244648	1.056018168
C	3.949648523	-4.967932487	1.047258666
H	-4.790802596	4.777214973	1.730836511
H	4.840579531	-4.733742459	1.644595838
H	3.017448762	5.093815407	1.336429679
H	-2.965526322	-5.024677098	1.209979307
H	-7.34820586	1.586189227	1.759017442
H	7.423298863	-1.634881467	1.779339219
H	4.692995101	2.471179138	1.527145917
H	-4.610366774	-2.353646986	1.378319101
H	-1.1811479	4.728029791	2.021645736
H	1.22124453	-4.640298687	2.223648884
C	-4.647785299	1.694437969	1.925194987
C	4.727337427	-1.678272955	2.031163248
H	-3.156390103	5.472493333	1.654305358
H	3.192618457	-5.394139276	1.714192825
H	-5.244287311	2.506362914	2.361161232
H	5.310447028	-2.517378533	2.432570624
H	-0.251633066	3.287642873	1.53817763
H	0.285554711	-3.187032485	1.791803293
C	-0.850764721	3.733601392	2.341326805
C	0.915850175	-3.64442871	2.564397442

H	-4.348106188	1.034942344	2.752982093
H	4.475056154	-1.022240023	2.877286733
C	-2.019205021	2.817070568	2.755037933
C	2.111978656	-2.744049543	2.932289836
H	-0.764419797	1.015269601	2.680539125
H	0.859429199	-0.941151225	2.945089621
H	-0.183654698	3.871404928	3.204323216
H	0.287439294	-3.784055547	3.456132154
C	-1.403990538	1.552798284	3.387066289
C	1.542024412	-1.481388497	3.608697001
H	-3.334715565	4.442177077	3.496577843
H	3.456434499	-4.388795447	3.572648595
H	-3.668254943	2.854820731	4.215142476
H	3.812872289	-2.824283071	4.329800919
C	-2.874376467	3.510668925	3.836099801
C	3.006732162	-3.46937635	3.959497446
H	-0.781392836	1.853888147	4.242479192
H	0.980174677	-1.784390866	4.505357591
H	-2.163000348	0.858242512	3.761885263
H	2.32019176	-0.780415944	3.926757771
H	-2.228483346	3.756814399	4.692617877
H	2.393926794	-3.745982516	4.831232969
N	2.36947968	-1.815523547	-3.124763629
H	2.478102686	-2.829155618	-3.078251846
H	2.577321469	-1.546304176	-4.087868262
H	1.375778131	-1.624474772	-2.987212004

Supplementary Table 55 | Cartesian coordinate of the product complex for elimination of the ammonia ligand from XVII. Units are presented in Å.

SCF energy (in toluene) = -3909.59414048 hartree

ZPE = 1.309396 hartree

Atom	X	Y	Z
Mo	-2.624288372	0.136416637	0.053949422
Mo	2.623528343	0.01263996	0.18633786
P	3.168858121	1.803472654	-1.417443345
P	-3.297214154	-1.843014158	-1.391566767
N	-4.857790983	0.267611985	0.102144851
N	4.864338105	-0.104892158	0.191124275
P	2.988704416	-2.067085365	1.461473112
P	-2.999971883	2.177635102	1.519161591
N	-0.559062058	0.068871314	0.048563771
N	0.599513768	0.050174655	0.098505687
N	-2.646582241	1.927573127	-2.557856373
N	-2.617964202	1.27967235	-1.621999226
N	-2.651600293	-1.025228268	1.72086341
N	-2.684655192	-1.692478296	2.642079024
N	2.745487107	1.11374055	1.780243644
N	2.821399087	1.780816991	2.712271664
H	2.597416181	1.891143881	-5.278712933
H	-2.835569232	-2.938433074	-5.114677923
H	-4.157815552	-1.987161935	-4.433795826
H	4.126797395	1.657776209	-4.424091301
C	3.140458249	2.132538681	-4.35169859
C	-3.412339009	-2.755760014	-4.194884039
H	-2.441533956	-0.226805715	-3.861317933
H	3.086240955	-0.455569114	-3.36267065
H	-3.942022903	-3.680742928	-3.953449426
H	3.283560289	3.217242164	-4.333050231
H	-1.16285487	-1.270581146	-4.496283408
H	1.601130686	-0.105571934	-4.262534119
C	2.133818863	0.089482478	-3.318877727
C	-1.736974624	-1.020026994	-3.591026122
H	0.3786047	2.005092377	-4.018782125
H	-0.754780184	-3.459763298	-3.771844289
C	2.331816271	1.614428157	-3.145315801
C	-2.445371688	-2.291500875	-3.085170915

H	-4.578228315	-0.324251534	-2.749439788
H	4.855668425	0.56867648	-2.649529224
H	1.519179899	-0.327634032	-2.50798316
H	-1.045785515	-0.616293511	-2.844490668
H	5.548790264	2.130934946	-2.215932649
H	-5.593396067	-1.7432284	-2.479095446
C	0.945406305	2.285743981	-3.118581934
C	-1.356810475	-3.359918188	-2.856276012
H	2.847773909	4.695925149	-2.816169743
H	-3.772096231	-4.946477366	-2.250643712
H	0.356367428	1.972306888	-2.248725195
H	-0.676689186	-3.076183153	-2.042794092
C	4.934941456	1.304079467	-1.834293973
C	-4.903928159	-1.0455427	-1.985993524
H	7.583862138	1.229451837	-1.340121281
H	-7.573038497	-0.699724889	-1.680992663
H	1.015908032	3.380268926	-3.109761861
H	-1.769646023	-4.349087186	-2.631193483
H	4.573719848	4.312315362	-2.630036764
H	-5.332237864	-4.098420943	-2.163469119
H	-4.443855119	2.935689707	-0.989371932
H	4.234214561	-2.45746324	-1.187566636
C	3.665667234	4.61441583	-2.091624306
C	-4.514671046	-4.5231999	-1.566720806
H	-2.494544615	5.325790187	-0.608795009
H	2.137786464	-4.715406043	-1.154097514
C	7.028005257	0.630934518	-0.621050158
C	-7.01294922	-0.228780316	-0.876063532
H	3.846049933	5.623311752	-1.689332813
H	-4.935892518	-5.360784753	-0.989983615
C	5.634013364	0.609033547	-0.695937287
C	-5.61899695	-0.307900303	-0.880979151
H	-4.789036315	4.65629891	-0.77445309
H	4.509963094	-4.212327722	-1.296606507
H	-1.907634006	3.670792593	-0.841321462
H	1.721794688	-2.991376075	-1.030688215
C	-4.622333143	3.709434954	-0.239231168
C	4.414081979	-3.365502333	-0.598545609
C	-2.217814552	4.39741048	-0.085885178
C	1.980263091	-3.88544915	-0.44772606
H	-8.756445583	0.509976507	0.17414082
H	8.778208329	-0.10894346	0.424085719
C	3.327494814	3.660688163	-0.932294786
C	-3.910421415	-3.500851836	-0.584225087
H	-5.550575708	3.467850094	0.291760616
H	5.376995703	-3.24882947	-0.086523907
C	-7.6707545	0.442838375	0.153884419

C	7.692528762	-0.115703132	0.353061307
H	-1.352647992	4.621728889	0.545097507
H	1.113507087	-4.136751789	0.172598916
H	1.153946499	4.044988747	-0.950556998
H	-1.938777943	-4.494060911	-0.504166402
H	5.438715273	3.512808271	-0.297882542
H	-5.934785129	-2.813839159	-0.045177726
C	-3.431579072	3.89790039	0.728301497
C	3.26001345	-3.65827182	0.385562676
C	2.007292819	4.098874707	-0.264975694
C	-2.734632744	-4.161779525	0.169798268
C	4.453382203	3.750705373	0.122428402
C	-5.01549899	-3.15912483	0.442012994
H	4.500968206	4.782350186	0.503273717
H	-5.266131449	-4.071916611	1.003414875
H	-4.168834093	5.881503063	1.178308924
H	3.834632748	-5.749354037	0.432954109
C	6.917305497	-0.872850034	1.231072368
C	-6.897349682	1.026485122	1.156513956
H	1.783733241	3.477801703	0.609021361
H	-2.294722025	-3.49020005	0.911934394
C	5.524812018	-0.848435256	1.140046227
C	-5.504981398	0.933348943	1.110255368
C	-3.843973285	4.989742254	1.736154826
C	3.612566796	-4.945463574	1.152415541
H	-4.687626541	4.679895876	2.366567244
H	4.501799811	-4.821726247	1.784389225
H	2.098106037	5.142686482	0.073504956
H	-3.105096934	-5.050376884	0.703669214
H	-7.364416408	1.562822519	1.979682294
H	7.383859673	-1.478916896	2.005065502
H	4.27138011	3.09061285	0.976204579
H	-4.705694232	-2.401816526	1.165434561
H	-1.097703461	4.457810581	2.66746985
H	0.934414602	-4.31351267	2.377974942
C	-4.664730077	1.580319867	2.182396149
C	4.703441978	-1.650930325	2.119136939
H	-3.021599998	5.300859027	2.388004289
H	2.789533377	-5.296792212	1.783422571
H	-5.245471621	2.356214166	2.697918274
H	5.281171136	-2.515881581	2.473876535
H	-0.21243308	3.046781468	2.039483051
H	0.1724217	-2.744757967	2.017191864
C	-0.811267888	3.421739012	2.87826739
C	0.765165711	-3.298130173	2.755874505
H	-4.396960472	0.826801872	2.937529668
H	4.497585167	-1.026204001	3.00089102

C	-2.021223327	2.508417005	3.163789987
C	2.077295871	-2.5627272	3.094486665
H	-0.840217002	0.681246236	2.878616364
H	1.070275247	-0.613877572	3.151889261
H	-0.154834089	3.436402006	3.76015787
H	0.151827539	-3.38667488	3.664571946
C	-1.461862041	1.155413573	3.6437557
C	1.705151532	-1.236269339	3.788878024
H	-3.2922653	4.075026497	4.094416863
H	3.220252364	-4.369293777	3.677100185
H	-3.68725663	2.422028386	4.602292445
H	3.814743582	-2.867595724	4.410895303
C	-2.867297612	3.092703331	4.315331669
C	2.913830342	-3.400860214	4.082232781
H	-0.836348009	1.327559443	4.53204118
H	1.15441068	-1.460269973	4.715124775
H	-2.24986169	0.451036669	3.930476827
H	2.583104679	-0.639228259	4.062190715
H	-2.223620239	3.207091963	5.200748716
H	2.311880695	-3.597700341	4.98273522
N	5.738326139	-1.588227478	-3.562863119
H	6.431825385	-1.446853034	-2.826341683
H	6.2432537	-1.546968428	-4.449537206
H	5.410789713	-2.549688133	-3.461486605

Supplementary Table 56 | Cartesian coordinate of the reactant complex for coordination of dinitrogen to XVIII. Units are presented in Å.

SCF energy (in toluene) = -3962.54624778 hartree

ZPE = 1.279297 hartree

Atom	X	Y	Z
Mo	-2.569491425	0.015759051	-0.025604302
Mo	2.665364896	-0.256928488	-0.267710335
P	3.173001668	1.796826612	-1.546120374
P	-3.148728274	-1.970577322	-1.512868268
N	-4.804146263	0.109582542	-0.071052502
N	4.910244065	-0.311762461	-0.31178691
P	3.104118882	-2.11623662	1.288443214
P	-3.03878986	2.034661691	1.437834707
N	-0.508206622	-0.07853243	-0.047687047
N	0.647403364	-0.138387035	-0.127958946
N	-2.488768716	1.838967353	-2.616185552
N	-2.504953917	1.177867588	-1.690233886
N	-2.651367338	-1.152247241	1.632892129
N	-2.722923561	-1.818143309	2.553624124
N	2.641225143	-2.017519677	-2.806253865
N	2.653958336	-1.353891252	-1.868809524
N	3.529336901	2.839731891	4.299313224
N	2.598398304	3.292742638	4.693324644
H	2.568366143	3.368848346	-5.061496226
H	-2.475633742	-3.045028989	-5.20201285
H	-3.848886382	-2.116148197	-4.593695441
H	4.01716115	2.5263859	-4.507039812
C	3.166199111	3.130418823	-4.168370289
C	-3.104803493	-2.873419767	-4.315493359
H	-2.198840513	-0.323987916	-3.91544483
H	2.586239594	0.403572248	-4.133923929
H	-3.631344588	-3.807499395	-4.103681397
H	3.553875731	4.074672115	-3.775152793
H	-0.874317681	-1.343410127	-4.497760101
H	1.234101792	1.356463151	-4.768499036
C	1.773710862	1.082438452	-3.84930992
C	-1.494525035	-1.108875362	-3.620170585
H	0.431032055	3.371053061	-3.70275502
H	-0.445263601	-3.517327474	-3.744066466

C	2.277913428	2.368442722	-3.163225824
C	-2.204022287	-2.395465177	-3.156748998
H	-4.389597153	-0.458092637	-2.91353395
H	4.56711697	0.64661428	-3.116795758
H	1.090669216	0.524020579	-3.203030281
H	-0.840345502	-0.70705823	-2.840799463
H	5.473309168	2.067095754	-2.591644411
H	-5.395847798	-1.892340738	-2.696365319
C	1.045891473	3.221633516	-2.803259088
C	-1.107760121	-3.442336315	-2.870045617
H	3.291499356	5.0288456	-1.876798308
H	-3.523329431	-5.069058407	-2.399697642
H	0.414311885	2.722883704	-2.057984913
H	-0.482161545	-3.150506023	-2.0175017
C	4.836856476	1.245799461	-2.234625939
C	-4.738136442	-1.188953257	-2.169668293
H	7.495683364	0.848911689	-2.178263862
H	-7.42280959	-0.885770993	-1.977628765
H	1.31668054	4.214557878	-2.424800781
H	-1.513636964	-4.441824634	-2.679450466
H	4.943630755	4.374380606	-1.884378576
H	-5.100027528	-4.247685754	-2.391892778
H	-4.377586883	2.796631692	-1.12449701
H	4.158430484	-3.374212292	-1.134151108
C	4.076836622	4.595576307	-1.247854897
C	-4.309145637	-4.664110833	-1.754723006
H	-2.48927203	5.201334597	-0.650265076
H	1.880218387	-5.365561097	-0.282791012
C	6.996517697	0.283666625	-1.393865362
C	-6.905636802	-0.412975857	-1.145614682
H	4.387230408	5.374061514	-0.53344353
H	-4.746485898	-5.511659921	-1.205263695
C	5.609033689	0.375725198	-1.273939812
C	-5.511517763	-0.470449424	-1.091186153
H	-4.764025876	4.50826184	-0.908810717
H	4.287945131	-5.094068193	-0.729341748
H	-1.857306141	3.558286184	-0.844916277
H	1.649781998	-3.679954435	-0.792163022
C	-4.604249504	3.558896549	-0.375955924
C	4.299779959	-4.077457068	-0.307880283
C	-2.220131064	4.281058588	-0.109539892
C	1.83949963	-4.324327173	0.072628857
H	-8.704713636	0.288090928	-0.16622826
H	8.804436377	-0.626822524	-0.620494456
C	3.596094781	3.355139585	-0.473833753
C	-3.774151482	-3.637152519	-0.737375989
H	-5.550500472	3.294765479	0.110508161

H	5.296434998	-3.914404537	0.121161708
C	-7.618204093	0.238729787	-0.139747375
C	7.724493459	-0.532853868	-0.529143941
H	-1.392631924	4.527068629	0.563362908
H	0.987776636	-4.240935249	0.757429232
H	1.509687621	4.054108116	-0.253721861
H	-1.784691979	-4.582847393	-0.558981361
H	5.665113392	2.744069285	0.001538512
H	-5.836763993	-2.9937592	-0.298615126
C	-3.461589251	3.758093299	0.646132417
C	3.178775235	-3.964907348	0.750009263
C	2.349151743	3.716465262	0.362733291
C	-2.626462092	-4.282321812	0.072039644
C	4.71402525	2.944307003	0.509181971
C	-4.9343736	-3.317738529	0.233017988
H	4.886254537	3.762436864	1.224921228
H	-5.191783733	-4.234089294	0.785433616
H	-4.245181182	5.728463038	1.074941002
H	3.611646876	-5.969193782	1.447182737
C	7.022744732	-1.216811629	0.463388414
C	-6.899093924	0.825704516	0.900787425
H	2.001172729	2.858080908	0.953377332
H	-2.246529986	-3.613489324	0.848994199
C	5.638611223	-1.078658853	0.564759887
C	-5.504787318	0.755206436	0.913438073
C	-3.936056283	4.836312067	1.640889947
C	3.47326433	-4.966663545	1.880571026
H	-4.805210367	4.510869186	2.227206604
H	4.391721726	-4.720938976	2.429793965
H	2.597077108	4.528948388	1.063120767
H	-3.005310072	-5.18837998	0.569503763
H	-7.410543638	1.346331271	1.707479786
H	7.543811258	-1.848728921	1.179586952
H	4.44657993	2.046678252	1.081083599
H	-4.675585652	-2.551110291	0.966823434
H	-1.234181193	4.342046537	2.693350204
H	0.885392762	-3.646144635	2.940652963
C	-4.720837642	1.406295543	2.02565275
C	4.900838506	-1.719310461	1.711552713
H	-3.150188171	5.152553797	2.333963749
H	2.650251772	-5.036315776	2.600628836
H	-5.335882148	2.167794545	2.522523837
H	5.466655826	-2.574172818	2.104965565
H	-0.304252444	2.955660155	2.084890327
H	0.295008316	-2.176070451	2.133563507
C	-0.939299562	3.309314639	2.907521317
C	0.869107931	-2.550734305	2.989184798

H	-4.471596289	0.650473349	2.784735288
H	4.845689749	-0.983723131	2.528544678
C	-2.143453566	2.372368665	3.133586199
C	2.287739887	-1.950775465	3.032020771
H	-0.931289228	0.557506229	2.887722532
H	1.548691125	0.042806883	2.476628024
H	-0.321014915	3.331698246	3.817455752
H	0.319094356	-2.274229958	3.900910557
C	-1.582966898	1.026115194	3.631821238
C	2.157605175	-0.427475521	3.260332513
H	-3.481623107	3.910447252	4.01181062
H	3.165785045	-3.637971598	4.156574336
H	-3.871178433	2.247838635	4.490423594
H	4.085889567	-2.129302851	4.298326835
C	-3.050493029	2.934780029	4.248642175
C	3.075342557	-2.549532047	4.214753608
H	-0.99523727	1.204296932	4.545336464
H	1.662051553	-0.237663337	4.224580106
H	-2.37128864	0.310733283	3.887795564
H	3.132856928	0.078156629	3.292610272
H	-2.4526975	3.054732048	5.165216449
H	2.550471044	-2.315487098	5.153905244

Supplementary Table 57 | Cartesian coordinate of the transition state for coordination of dinitrogen to XVIII. Units are presented in Å.

SCF energy (in toluene) = -3962.53971198 hartree

ZPE = 1.281230 hartree

imaginary frequency: 64*i*

Atom	X	Y	Z
Mo	-2.592612699	0.105627233	0.035617467
Mo	2.649526078	-0.176206421	-0.1251175
P	3.223020507	1.969125262	-1.297632057
P	-3.191539086	-2.033983317	-1.198971721
N	-4.83303084	0.179464493	0.027468642
N	4.872901856	-0.217137021	-0.050575836
P	3.07038783	-2.296429285	1.137488114
P	-3.056870872	2.276368472	1.267329669
N	-0.533389453	0.014049	0.008763732
N	0.621712063	-0.052356284	-0.042758633
N	-2.531876371	1.632843098	-2.74224589
N	-2.549808938	1.074089836	-1.751807107
N	-2.604819357	-0.861019432	1.819023546
N	-2.614475518	-1.417714416	2.812031109
N	2.714453328	-1.64376359	-2.838191493
N	2.679030712	-1.087652789	-1.832583523
N	2.553729741	1.610279071	2.922644821
N	2.469502998	2.192376208	3.862287186
H	2.708225661	3.503938698	-4.844983344
H	-2.569020542	-3.517955801	-4.75367097
H	-3.942746955	-2.54012044	-4.232693002
H	4.064804899	2.517329689	-4.294923133
C	3.294054779	3.221011445	-3.95703397
C	-3.184237471	-3.25086756	-3.881053292
H	-2.297775942	-0.656554706	-3.786642467
H	2.418148869	0.600409818	-3.893730896
H	-3.694543577	-4.16026697	-3.552978777
H	3.79219022	4.125383159	-3.595287786
H	-0.993601339	-1.736113749	-4.299716247
H	1.140791699	1.682531023	-4.472864381
C	1.688634035	1.3525261	-3.577064796
C	-1.580598915	-1.403569427	-3.430746492

H	0.600934927	3.742849556	-3.464681903
H	-0.515761499	-3.802875499	-3.29527235
C	2.346078957	2.582717706	-2.919696693
C	-2.269307876	-2.631896964	-2.802880866
H	-4.47304503	-0.703580984	-2.738386445
H	4.580985959	0.699470013	-2.812189531
H	0.977717528	0.864673152	-2.902543216
H	-0.896139276	-0.915870247	-2.72990642
H	5.528895993	2.124392239	-2.364691621
H	-5.464105181	-2.110291705	-2.340514317
C	1.216515831	3.572206437	-2.569467579
C	-1.158486635	-3.630299448	-2.419967251
H	3.597528207	5.172783919	-1.755330369
H	-3.565757957	-5.217077404	-1.725519729
H	0.554027052	3.17371454	-1.790655004
H	-0.518189984	-3.236679501	-1.620828887
C	4.872613422	1.339380433	-1.966397822
C	-4.802324677	-1.347704168	-1.90979083
H	7.550071805	1.075835449	-1.685431781
H	-7.484832711	-1.048117752	-1.688772283
H	1.59239331	4.548328613	-2.243109208
H	-1.55085388	-4.60391776	-2.106783823
H	5.193298759	4.393411135	-1.688059149
H	-5.152466488	-4.415776595	-1.756410725
H	-4.466901336	2.662998652	-1.300367703
H	4.535713195	-2.778734471	-1.377500313
C	4.328900155	4.705446282	-1.087592408
C	-4.334316516	-4.746834709	-1.10344158
H	-2.61595707	5.22350496	-1.150133764
H	2.564475797	-5.249535911	-1.248447653
C	7.010714211	0.485420869	-0.947690882
C	-6.953910554	-0.47965768	-0.928336041
H	4.680816666	5.486733748	-0.395712899
H	-4.74312632	-5.5314509	-0.448298277
C	5.616078245	0.506786049	-0.952740162
C	-5.558385456	-0.518066599	-0.90249674
H	-4.851375384	4.389528726	-1.354345499
H	4.868634359	-4.516972133	-1.379887666
H	-1.984756793	3.572699216	-1.239612973
H	2.05049669	-3.562417285	-1.405803509
C	-4.686606909	3.535061883	-0.680767008
C	4.706128437	-3.642997494	-0.73100971
C	-2.322872382	4.349412067	-0.548453667
C	2.302640271	-4.342144521	-0.68211966
H	-8.737312988	0.312138627	0.010074448
H	8.78606918	-0.314785714	0.003535272
C	3.734747653	3.539912578	-0.274456441

C	-3.780317131	-3.607279219	-0.224887683
H	-5.628492279	3.352057859	-0.150791697
H	5.632484373	-3.479367242	-0.167384933
C	-7.650012204	0.275135874	0.01514261
C	7.698697803	-0.286846406	-0.010362335
H	-1.47278621	4.649533386	0.071828831
H	1.409717225	-4.571930406	-0.091011358
H	1.759113653	4.515683068	-0.13716053
H	-1.76838336	-4.491730093	0.012410346
H	5.761001036	2.854641758	0.261681466
H	-5.833880033	-2.922701227	0.201651536
C	-3.535332847	3.886346977	0.288991305
C	3.505281869	-3.935815016	0.196729024
C	2.502835566	4.039986559	0.509171863
C	-2.609850007	-4.151394569	0.623233226
C	4.807363405	3.095753066	0.744621959
C	-4.913676789	-3.184482416	0.736959461
H	4.994869312	3.917080951	1.452984418
H	-5.149190225	-4.033479421	1.396360594
H	-4.378790933	5.867195418	0.497746042
H	4.242529624	-5.951842185	0.460411698
C	6.948252503	-1.025427639	0.904529665
C	-6.912919418	0.980620261	0.965634559
H	2.004866533	3.219771464	1.037312779
H	-2.238515713	-3.406396987	1.331794879
C	5.554404771	-0.977915372	0.86800881
C	-5.518037613	0.923424662	0.951693171
C	-4.025456504	5.061069318	1.158822975
C	3.896128314	-5.123207279	1.096957632
H	-4.868772686	4.78217801	1.804108964
H	4.717265127	-4.878253866	1.783389641
H	2.815151197	4.785620609	1.256542996
H	-2.963753451	-5.015418399	1.206515469
H	-7.410584552	1.583630007	1.722039811
H	7.43594899	-1.645036297	1.654277686
H	4.495733175	2.217708482	1.320004373
H	-4.633805207	-2.340554988	1.371865196
H	-1.293179964	4.75987209	2.21298891
H	1.071063045	-4.57874058	2.051083598
C	-4.715484743	1.697737339	1.967062243
C	4.738363128	-1.764708768	1.860956572
H	-3.236375653	5.485708479	1.787798375
H	3.053559306	-5.500868479	1.686071041
H	-5.326673608	2.503274437	2.394205919
H	5.333898426	-2.590249244	2.272824799
H	-0.329814313	3.345041838	1.734261434
H	0.277568615	-3.065965039	1.554763911

C	-0.959158634	3.765841514	2.528517652
C	0.833603065	-3.555547714	2.363735085
H	-4.437198135	1.029227599	2.795136189
H	4.470842695	-1.113117915	2.70759967
C	-2.126329613	2.824585259	2.887617147
C	2.0903206	-2.750502964	2.748240764
H	-0.864148611	1.028192359	2.811633015
H	1.049513039	-0.821465694	2.627720833
H	-0.324496755	3.903788575	3.416650503
H	0.161706998	-3.624385245	3.231992087
C	-1.51340212	1.557793962	3.516713203
C	1.620662806	-1.413321514	3.351846591
H	-3.479675287	4.422049333	3.618791388
H	3.224422961	-4.489956965	3.519044015
H	-3.819115311	2.818596385	4.297278238
H	3.768112775	-2.930656226	4.164242849
C	-3.020650998	3.488855422	3.955614848
C	2.888776501	-3.499119832	3.834837208
H	-0.911553727	1.851428158	4.390487821
H	0.972665759	-1.610354599	4.219026529
H	-2.273494762	0.852483158	3.867399894
H	2.464489074	-0.809690794	3.707654928
H	-2.40492936	3.726058308	4.836884499
H	2.247174374	-3.63760415	4.718827268

Supplementary Table 58 | Cartesian coordinate of the reactant complex for coordination of dinirogen to XVI. Units are presented in Å.

SCF energy (in toluene) = -1983.03005450 hartree

ZPE = 0.670536 hartree

Atom	Coordinates (Angstroms) X	Y	Z
Mo	0.421011816	-0.172212312	0.086856761
P	0.279975163	2.008794371	1.155932205
N	-1.547014002	-0.319852552	1.082737875
P	-0.427752961	-2.14991007	-1.011812183
N	2.299759907	-0.185474602	-0.583161413
N	3.407398453	-0.240153754	-0.886725898
H	2.188014266	4.20354791	3.793076154
H	0.710764301	3.268296464	4.048411865
C	1.289190755	3.812380518	3.290444432
H	2.056543259	1.173072471	3.447709577
H	0.697146229	4.673306972	2.969354581
H	3.462648559	2.215285658	3.22389607
C	2.598943764	1.774806322	2.702879409
H	3.50866541	4.024439475	1.619090665
C	1.715992917	2.900465075	2.122094253
H	-0.205273797	1.202188661	3.37354324
H	2.978588346	1.118143495	1.911301594
H	-1.439443325	2.395952127	3.008169797
C	2.583259484	3.692524593	1.12389559
H	0.008485363	5.271278406	1.044735135
H	2.869545897	3.074002601	0.263626409
C	-0.879618308	1.552162544	2.576713424
H	-3.15687215	0.861474371	3.833763391
H	2.075317938	4.589231467	0.751407366
H	-1.385374623	4.585655935	1.910139583
H	-0.153684215	-3.580531614	1.5266373
C	-0.926991677	4.714646149	0.920584262
H	1.65808964	-5.280906118	-0.311268405
C	-2.984323137	0.230946914	2.963425292
H	-1.604324542	5.352019395	0.330703303
C	-1.838425241	0.444638061	2.193692525
H	-0.32613159	-5.296977009	1.118426312
H	1.963858167	-3.553804653	-0.012973232
C	-0.598434442	-4.277375325	0.805393104

C	1.429405074	-4.261897091	-0.660135772
H	-4.775972526	-0.961830878	3.223012392
C	-0.725432716	3.37333134	0.193162084
H	-1.687869195	-4.186549963	0.891396407
C	-3.886842256	-0.776905781	2.624288664
H	1.848760201	-4.153907167	-1.666237626
H	0.958423376	4.079212326	-1.052782039
H	-2.718795701	2.648635001	0.812697448
C	-0.09522697	-4.026549869	-0.63283604
C	-0.028711243	3.617310772	-1.161674394
C	-2.1230755	2.783203369	-0.098611445
H	-2.675674365	3.47217264	-0.755565014
H	-0.628533717	-6.059202849	-1.192451612
C	-3.621237575	-1.524849052	1.473129152
H	0.105345989	2.679406853	-1.71562562
C	-2.480225113	-1.270088774	0.716852704
C	-0.783535379	-5.035329643	-1.568811304
H	-1.867586748	-4.872655942	-1.632187967
H	-0.643227426	4.289430452	-1.780514463
H	-4.312965393	-2.296365233	1.140695929
H	-2.059408086	1.810897978	-0.602139012
H	1.028160057	-3.372099724	-3.562048842
C	-2.265271145	-1.971454636	-0.600957064
H	-0.373105177	-5.000214241	-2.584414547
H	-2.836767097	-2.908477614	-0.640010532
H	1.750102703	-1.868203634	-2.940446591
C	0.914681091	-2.281563652	-3.519081554
H	-2.678636487	-1.327594618	-1.391424133
C	-0.446528355	-1.865742467	-2.928218632
H	0.232471276	0.186119082	-2.511020971
H	1.002200958	-1.907655782	-4.550917063
C	-0.578451666	-0.329536278	-3.055995161
H	-1.515148553	-3.616850333	-3.742063192
H	-2.577537323	-2.253599867	-3.349199937
C	-1.584565815	-2.52471038	-3.730303454
H	-0.488474304	-0.027749012	-4.111154838
H	-1.546596748	0.04089027	-2.694760483
H	-1.538718021	-2.184144778	-4.77683016
H	1.898543176	-0.681387419	2.459343395
N	1.208358182	-1.223979337	1.936441696
H	1.660997406	-2.101945743	1.677907112
H	0.450883609	-1.458298711	2.583745862
N	2.524016653	2.073817502	-3.455317036
N	3.597920113	2.028093967	-3.185851039

Supplementary Table 59 | Cartesian coordinate of the transition state for coordination of dinirogen to XVI. Units are presented in Å.

SCF energy (in toluene) = -1983.02333356 hartree

ZPE = 0.672276 hartree

imaginary frequency: 94*i*

Atom	X	Y	Z
Mo	-0.159339036	-1.67018422	-0.546120201
P	-0.293237701	0.491081506	0.644161216
N	-2.164276933	-1.811162776	0.414485239
P	-1.046224076	-3.661243359	-1.673843903
N	1.773117526	-1.834372114	-0.985546098
N	2.905677563	-1.991853244	-1.097506526
H	1.660832414	2.426408584	3.445900695
H	0.20563844	1.443773192	3.640906858
C	0.752460529	2.077025391	2.930388196
H	1.591413673	-0.518971851	2.815965419
H	0.139401887	2.958109224	2.723992856
H	2.967040154	0.559806424	2.608320711
C	2.085618894	0.148315483	2.093427407
H	2.909210982	2.524490971	1.241270602
C	1.159662854	1.305493499	1.658382477
H	-0.742055826	-0.51379947	2.785078631
H	2.437836025	-0.438091835	1.237002061
H	-1.958455929	0.729798238	2.565945339
C	1.980342613	2.225017286	0.732468981
H	-0.683721822	3.726842289	0.960589522
H	2.262244729	1.71569676	-0.198063241
C	-1.42787267	-0.078811923	2.041787599
H	-3.703507569	-0.791127046	3.270134627
H	1.441993272	3.143818066	0.474129455
H	-2.011628846	2.853332312	1.757789259
H	-1.024244604	-4.75408791	1.008699214
C	-1.594584082	3.14398791	0.784902615
H	1.054657543	-6.621619313	-0.472924183
C	-3.559699677	-1.364937582	2.356444846
H	-2.321716352	3.82147555	0.310650975
C	-2.420433587	-1.124582463	1.582906065
H	-0.957444548	-6.520251676	0.842636126

H	1.308151083	-4.856888703	-0.444683013
C	-1.308795145	-5.592618846	0.365546896
C	0.814608202	-5.675431274	-0.98229345
H	-5.379481414	-2.524178785	2.558663385
C	-1.3518128	1.93697964	-0.139627592
H	-2.404946895	-5.627532736	0.350237856
C	-4.490893622	-2.324178384	1.964014204
H	1.273169306	-5.71232633	-1.977143367
H	0.292841642	2.868215319	-1.292924768
H	-3.314514417	1.046478354	0.361731462
C	-0.715323085	-5.48575671	-1.056799959
C	-0.69814506	2.428307101	-1.44656658
C	-2.727304194	1.341326361	-0.515847562
H	-3.308077291	2.101007435	-1.061047891
H	-1.149810971	-7.590166204	-1.35280609
C	-4.24701379	-3.012558536	0.772107466
H	-0.596661144	1.614621827	-2.171378337
C	-3.106612622	-2.738695397	0.021223622
C	-1.316174457	-6.637931892	-1.88175879
H	-2.399242619	-6.529483201	-2.021422785
H	-1.335599602	3.2022448	-1.901252924
H	-4.949606099	-3.759661911	0.408773963
H	-2.625898878	0.462535875	-1.16422654
H	0.446280917	-5.212504498	-3.96463044
C	-2.884099518	-3.429457509	-1.296982738
H	-0.848869211	-6.734708759	-2.867690541
H	-3.475218576	-4.353487536	-1.351483442
H	1.07557244	-3.568414024	-3.715729767
C	0.256370296	-4.149714002	-4.157965251
H	-3.258422175	-2.772054348	-2.095921852
C	-1.113475427	-3.694437591	-3.618572732
H	-0.563066019	-1.570257026	-3.667230555
H	0.293407932	-4.006960128	-5.248687118
C	-1.337636022	-2.233337036	-4.061123381
H	-2.114039947	-5.615030523	-4.096294447
H	-3.230294207	-4.255496809	-3.880350055
C	-2.237692801	-4.540871233	-4.251673998
H	-1.304227922	-2.175214767	-5.160083721
H	-2.315457668	-1.842671445	-3.748279917
H	-2.242429504	-4.370195	-5.33960547
H	1.219623315	-2.241479633	1.826475999
N	0.475703583	-2.754403933	1.350170192
H	0.846652482	-3.686977188	1.160716961
H	-0.28876905	-2.872985724	2.020622347
N	0.99802532	-0.022426159	-2.947895534
N	2.015649555	0.210281206	-3.336060334

Supplementary Table 60 | Cartesian coordinate of the transition state for elimination of the ammonia ligand from XX. Units are presented in Å.

SCF energy (in toluene) = -1983.06187392 hartree

ZPE = 0.673637 hartree

imaginary frequency: 50*i*

Atom	X	Y	Z
Mo	-0.291584638	-1.415184887	-0.836062705
P	-0.43524808	0.590889669	0.65135552
N	-2.298051447	-1.701446266	0.113342805
P	-1.00468225	-3.599856001	-1.844233151
N	1.577434974	-1.267625864	-1.531131887
N	2.678156405	-1.242440973	-1.84475295
H	1.552945281	1.89214834	3.755499048
H	0.115166176	0.866675483	3.774010536
C	0.640845049	1.636079132	3.1945581
H	1.34186102	-0.975743834	2.370218158
H	0.011579366	2.529494715	3.16968293
H	2.733110413	0.090151748	2.660027724
C	1.907997345	-0.127585911	1.963467103
H	2.842281805	2.327379912	1.629786704
C	1.038694373	1.132858346	1.791557983
H	-0.930798535	-0.830810294	2.542757549
H	2.338351742	-0.437748117	1.006021554
H	-2.064384308	0.518622179	2.611117052
C	1.90071175	2.191898467	1.076239601
H	-0.608751982	3.711895984	1.492745974
H	2.158600671	1.879967745	0.055915271
C	-1.591571651	-0.199685129	1.928714943
H	-4.111364204	-0.712121307	2.800116464
H	1.411043239	3.170430813	1.025062153
H	-2.035022133	2.853923257	2.118712346
H	-0.703584822	-4.074606791	1.072970443
C	-1.560624853	3.249492617	1.210810892
H	0.739356453	-6.446125737	0.034172304
C	-3.890358606	-1.233176122	1.871287125
H	-2.209459621	4.053388543	0.831200043
C	-2.635167301	-1.07144058	1.280549446
H	-1.161744121	-5.774336614	1.275701426

H	1.247716509	-4.795782435	-0.376323837
C	-1.302998424	-4.885770744	0.641439365
C	0.635545247	-5.639580876	-0.708370504
H	-5.823862945	-2.192879188	1.716226608
C	-1.404563201	2.178690225	0.112999259
H	-2.361291642	-4.607881121	0.697464278
C	-4.840568834	-2.05731453	1.270544305
H	1.034749005	-6.014865034	-1.654348748
H	0.311236299	3.159352407	-0.877081577
H	-3.42213405	1.384587335	0.527527332
C	-0.8486145	-5.225150286	-0.796597869
C	-0.696294596	2.800770398	-1.1112268
C	-2.825382565	1.749017779	-0.317473995
H	-3.345405129	2.627506813	-0.728255087
H	-1.613362853	-7.242450086	-0.584241849
C	-4.498172282	-2.703119205	0.082908967
H	-0.625166455	2.091424372	-1.940579189
C	-3.231491349	-2.513840694	-0.474034334
C	-1.685617209	-6.41303707	-1.30482295
H	-2.750522743	-6.165839573	-1.406256514
H	-1.280518385	3.665866952	-1.460237087
H	-5.207618237	-3.354543572	-0.42230003
H	-2.815028766	0.980715675	-1.094643057
H	0.331833018	-5.943414908	-3.595430958
C	-2.852730789	-3.20486191	-1.757655617
H	-1.329051712	-6.792669387	-2.2685983
H	-3.510196249	-4.066726827	-1.929699548
H	1.332695869	-4.474930354	-3.478251811
C	0.446806741	-4.914480931	-3.954069782
H	-3.00661762	-2.508991647	-2.595246438
C	-0.815578525	-4.059479601	-3.72455484
H	0.251020622	-2.18271143	-4.136672686
H	0.647047887	-4.969154904	-5.034472851
C	-0.623569105	-2.736948206	-4.493604072
H	-2.270498974	-5.730821937	-3.821825458
H	-2.939699597	-4.158391057	-4.297941439
C	-2.039995051	-4.78501627	-4.321901142
H	-0.476830601	-2.966677384	-5.560030343
H	-1.489834965	-2.072391652	-4.416526201
H	-1.837536187	-5.013073587	-5.37954741
H	2.788929247	-2.808640875	0.185504987
N	2.373498798	-3.203208005	1.0320066
H	2.733558292	-4.15582624	1.111867675
H	2.774579519	-2.680506741	1.811447834
N	-1.079645977	-0.455390455	-2.32900385
N	-1.579405287	0.113954283	-3.190925296

Supplementary Table 61 | Cartesian coordinate of the product complex for elimination of the ammonia ligand from XX. Units are presented in Å.

SCF energy (in toluene) = -1983.06752402 hartree

ZPE = 0.671638 hartree

Atom	X	Y	Z
Mo	-0.568818136	-1.327413488	-0.890349162
P	-0.662912932	0.574765171	0.666283436
N	-2.592810543	-1.666964014	0.035610161
P	-1.112554855	-3.558400974	-1.793993879
N	1.244467134	-1.029102405	-1.669123381
N	2.299588878	-0.870383478	-2.085393539
H	1.39943353	1.224752065	3.919691119
H	-0.311426233	0.803190034	3.800147431
C	0.509258134	1.311538558	3.277717671
H	0.465899447	-1.387910326	2.623078566
H	0.26005059	2.374285876	3.210423569
H	2.124671752	-0.813281823	2.796846753
C	1.246580578	-0.788376925	2.133757564
H	2.882180698	1.337914832	1.861287052
C	0.813113789	0.677656271	1.905447376
H	-1.533899776	-0.635157043	2.578528662
H	1.539280525	-1.290389924	1.202072386
H	-2.562688594	0.768293741	2.340128839
C	1.982504024	1.424799149	1.233244457
H	-0.191807303	3.646072237	1.559524435
H	2.220637225	1.006169806	0.247372717
C	-2.040580981	-0.03719626	1.80727077
H	-4.639226341	-0.434974242	2.440218703
H	1.77326751	2.49432572	1.112431134
H	-1.777130717	3.092253598	2.146672539
H	-0.938733727	-3.751939654	1.153489111
C	-1.214553277	3.397252324	1.254207198
H	1.04301682	-5.895993376	0.325393349
C	-4.333908727	-1.04797989	1.595234732
H	-1.675526633	4.324536186	0.880646514
C	-3.031170848	-0.934174066	1.106085999
H	-1.030137513	-5.491444149	1.478386263
H	1.267179971	-4.19582646	-0.125173632
C	-1.315914157	-4.705915532	0.762904557

C	0.816478782	-5.144228882	-0.446484179
H	-6.235607472	-2.060079439	1.386215063
C	-1.252220036	2.332754537	0.143774841
H	-2.411232026	-4.655014093	0.747946427
C	-5.218475527	-1.955078183	1.014229404
H	1.321848777	-5.45972745	-1.365239047
H	0.668566446	2.932240791	-0.763066914
H	-3.401194326	1.925896804	0.463958569
C	-0.715400448	-5.044169739	-0.620420494
C	-0.380686498	2.788682317	-1.045823458
C	-2.711209325	2.191806867	-0.347050082
H	-3.041756983	3.160637145	-0.750539237
H	-1.094620135	-7.149956942	-0.268435464
C	-4.763169218	-2.728711436	-0.051426359
H	-0.415551857	2.063601227	-1.865379606
C	-3.459168923	-2.56558666	-0.526965218
C	-1.277866456	-6.407859021	-1.06080568
H	-2.362123918	-6.377435349	-1.232315132
H	-0.7564966	3.751111037	-1.425008397
H	-5.415679055	-3.454156167	-0.532046126
H	-2.81195332	1.45158358	-1.146423106
H	0.620831354	-5.716446963	-3.375085513
C	-2.98989908	-3.380675183	-1.709296486
H	-0.797110509	-6.78441803	-1.970365187
H	-3.542400251	-4.329556438	-1.750612694
H	1.315609333	-4.077028117	-3.401985873
C	0.531814405	-4.719958349	-3.822620397
H	-3.238076322	-2.830987095	-2.629464812
C	-0.870502866	-4.108518567	-3.63306771
H	-0.1509413	-2.111429618	-4.20325298
H	0.73368299	-4.827624575	-4.898699852
C	-0.936933625	-2.821036631	-4.482587454
H	-1.9665751	-6.031688433	-3.600147041
H	-2.946448751	-4.650768579	-4.129555224
C	-1.941012249	-5.088892188	-4.154535018
H	-0.80260637	-3.086650183	-5.542338928
H	-1.897529664	-2.300876224	-4.393945515
H	-1.723515166	-5.331019829	-5.206045425
H	3.44126741	-2.20339538	-0.603128558
N	3.588476838	-2.825552915	0.195165083
H	4.025351138	-3.6690848	-0.18046725
H	4.298388296	-2.377723041	0.77751622
N	-1.446542726	-0.409362175	-2.367332611
N	-1.989989222	0.132280873	-3.218305805

Supplementary Table 62 | Cartesian coordinate of the reactant complex for coordination of dinitrogen to XIX'. Units are presented in Å.

SCF energy (in toluene) = -2036.01364113 hartree

ZPE = 0.640838 hartree

Atom	X	Y	Z
Mo	2.897435293	-0.190280519	-0.259172779
P	3.431585504	-2.227810116	0.998045567
N	5.140787208	-0.093022779	-0.283616928
P	3.248497387	2.063312315	-1.190648159
N	-0.249202745	-0.350957241	-0.163627671
N	0.889900355	-0.291853159	-0.210922777
N	-1.515602513	2.214990789	2.001332891
N	-0.656535393	1.713906732	2.488729382
N	2.976080795	-1.015407686	-2.021787137
N	3.058913729	-1.505991966	-3.053850624
H	2.85513776	-3.01693181	4.780095008
H	4.388334763	-2.628215063	3.993505539
C	3.400971466	-3.07685873	3.825828187
H	3.364163169	-0.334252847	3.283848795
H	3.546036723	-4.137006538	3.599605725
H	1.914437136	-0.83784463	4.160141699
C	2.409730178	-0.869124634	3.177867134
H	0.652707528	-2.87516907	3.536439442
C	2.600037762	-2.338941608	2.735397494
H	5.130754744	-1.227094287	2.411042251
H	1.771893214	-0.315232673	2.475044015
H	5.827434501	-2.689167222	1.728558559
C	1.203861679	-2.977410265	2.589429964
H	3.099598295	-5.326362106	1.857598143
H	0.61308076	-2.488780646	1.804536404
C	5.214426072	-1.811875322	1.483170133
H	7.865188359	-1.700421545	0.935199688
H	1.262797061	-4.048262445	2.361169165
H	4.82755132	-4.921589385	1.738806056
H	4.48630062	1.991313649	1.486464683
C	3.91658001	-5.117016555	1.157565494
H	2.517945766	4.331483512	1.796318188
C	7.299333427	-0.993030208	0.333156651
H	4.09087517	-6.036852701	0.57864131

C	5.90980464	-0.949929333	0.458614349
H	4.842008707	3.689450039	1.859644054
H	2.004536031	2.671394498	1.428344477
C	4.71293482	2.967699408	1.039198031
C	2.312216769	3.624608157	0.978093054
H	9.028790146	-0.139567339	-0.649821703
C	3.580006315	-3.968093894	0.190368201
H	5.674811437	2.882283846	0.520077208
C	7.946237081	-0.121662562	-0.542086586
H	1.459675258	4.01328221	0.412446476
H	1.408957129	-4.367541283	0.154439226
H	5.692606941	-3.734094961	-0.412421235
C	3.579269611	3.457194282	0.110623997
C	2.252885673	-4.271269183	-0.538242892
C	4.703393338	-3.871866268	-0.86687388
H	4.734588359	-4.813161546	-1.435776605
H	4.263399804	5.502029014	0.358321049
C	7.169652678	0.778595435	-1.26904554
H	2.008689666	-3.490002368	-1.265814143
C	5.77915771	0.77345097	-1.131487716
C	3.997951372	4.82234862	-0.466203205
H	4.878024673	4.743807296	-1.11810974
H	2.346548335	-5.224196377	-1.08077292
H	7.631232802	1.482661057	-1.957808801
H	4.53242598	-3.060343626	-1.580934675
H	1.27083624	4.4941808	-1.698490029
C	4.944456337	1.7319574	-1.949121849
H	3.193967599	5.303290573	-1.033752845
H	5.526668146	2.635706931	-2.176816392
H	0.430363611	2.931223691	-1.543413469
C	1.036031201	3.555192107	-2.213030585
H	4.710516493	1.257132215	-2.913272024
C	2.294929648	2.809917783	-2.699691182
H	1.174356924	0.940366671	-2.973726235
H	0.410761584	3.808869234	-3.081921848
C	1.823823068	1.609225869	-3.548208307
H	3.508944057	4.625226606	-3.075289741
H	3.997301929	3.216127284	-4.036451212
C	3.1378708	3.738225328	-3.597809848
H	1.255751273	1.985692283	-4.412481267
H	2.654255055	1.009342711	-3.937585519
H	2.514523665	4.087602802	-4.434920386

Supplementary Table 63 | Cartesian coordinate of the transition state for coordination of dinitrogen to XIX'. Units are presented in Å.

SCF energy (in toluene) = -2036.00646998 hartree

ZPE = 0.643386 hartree

imaginary frequency: 67*i*

Atom	X	Y	Z
Mo	2.7152904	-0.080353185	-0.077384972
P	3.266365356	-2.211728179	1.100749262
N	4.955788968	-0.031272329	-0.085684379
P	3.159868234	2.148604965	-1.163361172
N	-0.439130874	-0.134069408	0.009503869
N	0.700359877	-0.116769364	-0.027136369
N	0.275814525	1.878339381	2.903685239
N	1.284899412	1.621139104	2.522092138
N	2.732920037	-0.924478411	-1.825057814
N	2.783892233	-1.42514229	-2.855858999
H	2.692538878	-3.61215703	4.705177984
H	4.123873551	-2.762785362	4.118245717
C	3.28990522	-3.403211742	3.804471638
H	2.670155355	-0.668069524	3.550265263
H	3.704358814	-4.354909055	3.461716446
H	1.395285002	-1.60744145	4.341462623
C	1.869328325	-1.398959068	3.370160488
H	0.557877372	-3.698703758	3.37247459
C	2.390281208	-2.713027238	2.757708275
H	4.716931189	-1.04893924	2.644827085
H	1.122267717	-0.935837494	2.719951072
H	5.59067694	-2.482601841	2.106671903
C	1.16164674	-3.595822206	2.458237483
H	3.359750855	-5.401374325	1.625163541
H	0.520775444	-3.149151238	1.687315754
C	4.954379023	-1.658169623	1.758983433
H	7.639460683	-1.450705028	1.420459709
H	1.436140174	-4.606357735	2.136100194
H	5.018423594	-4.761701142	1.59280402
H	4.224742988	2.326398307	1.575912554
C	4.148001381	-5.012025035	0.972031227
H	2.816124419	4.980096119	1.415313429

C	7.093914517	-0.816227961	0.725620185
H	4.45135583	-5.834307211	0.306335864
C	5.69790486	-0.808182256	0.762043197
H	4.903994917	3.961284524	1.693324573
H	1.895105562	3.487636599	1.16872733
C	4.664536864	3.146317672	0.993883305
C	2.468603799	4.264175786	0.655135088
H	8.856889924	-0.010838036	-0.237119695
C	3.679487316	-3.822990866	0.111916546
H	5.608873709	2.789912415	0.568193304
C	7.769733649	-0.015841661	-0.1939916
H	1.787123086	4.801407801	-0.00936721
H	1.589923295	-4.520304352	-0.055546974
H	5.760812471	-3.266995608	-0.373308589
C	3.690203413	3.676359339	-0.082678535
C	2.42950777	-4.233484531	-0.69727344
C	4.815926472	-3.493397858	-0.882507737
H	4.991250499	-4.375779082	-1.51628383
H	4.756455544	5.563355121	-0.145166217
C	7.015886757	0.77641733	-1.059016948
H	2.095689451	-3.430013001	-1.360088574
C	5.6209401	0.753058459	-0.989970292
C	4.394752522	4.805715547	-0.857548517
H	5.266820723	4.452173299	-1.422588568
H	2.677470552	-5.103483192	-1.323996725
H	7.499999037	1.413500564	-1.795635469
H	4.567048218	-2.659882951	-1.544000935
H	1.488722381	4.762092889	-2.023746125
C	4.798542501	1.596082941	-1.929176209
H	3.719237708	5.315239152	-1.553124586
H	5.408478795	2.415137385	-2.330674389
H	0.473447923	3.38718193	-1.52136649
C	1.100193638	3.784574151	-2.329958846
H	4.489601223	0.97770878	-2.784280965
C	2.217362616	2.801651629	-2.733213674
H	0.85661501	1.072993738	-2.704658797
H	0.446809954	3.95435244	-3.198325268
C	1.54247102	1.580339779	-3.39162641
H	3.656785378	4.348471655	-3.408400925
H	3.86845912	2.770684643	-4.192656206
C	3.126358091	3.469042369	-3.787310693
H	0.965371596	1.925322299	-4.263169639
H	2.263755445	0.838591062	-3.748804538
H	2.504783708	3.800731311	-4.632897774

Supplementary Table 64 | Cartesian coordinate of the transition state for protonation of the bridging dinitrogen ligand in 1 by LutH+ [TS(BR)]. Units are presented in Å.

SCF energy (in toluene) = -4289.74738262 hartree

ZPE = 1.440997 hartree

imaginary frequency: 1040*i*

Atom	X	Y	Z
Mo	2.700799535	0.204071548	-0.131785265
Mo	-2.634300315	-0.145041435	-0.258835441
P	-4.007879695	1.879560104	0.909538014
P	3.910625461	-2.080975256	0.532455069
N	4.991898665	0.609248018	-0.41999196
N	-4.747129207	-0.232206945	-1.180515939
P	-2.57699827	-2.175214857	-1.851704631
P	2.817364429	2.665490096	-0.987967022
N	0.767612254	-0.073181483	0.125552058
N	-0.417979133	-0.240025297	0.352393235
N	3.341408566	1.125646766	2.830853111
N	3.028312252	0.817206701	1.785645085
N	2.715520768	-0.425754993	-2.077879067
N	2.858482857	-0.740519672	-3.155019205
N	-3.735020531	-1.89102989	2.124558243
N	-3.283392961	-1.273202897	1.277811262
N	-2.139091857	1.033395095	-1.824265042
N	-1.964661395	1.691402797	-2.735714356
H	-5.451004726	2.89564964	4.404313744
H	4.25432866	-4.275337018	3.73088798
H	5.352050957	-3.002781831	3.203342629
H	-6.215714973	1.822891921	3.235545306
C	-5.523474402	2.667038317	3.330460317
C	4.640936116	-3.755143546	2.842217404
H	3.453400237	-1.391603979	3.463673323
H	-4.351174274	0.242170942	3.478989356
H	5.189919795	-4.491116956	2.251864381
H	-5.971417768	3.536593025	2.845106074
H	2.405832846	-2.724015714	3.954661859
H	-3.586835815	1.314885135	4.657637533
C	-3.647287903	1.074640382	3.585767511
C	2.763363219	-2.153858037	3.085118378

H	-3.078801682	3.586252614	4.245143878
H	2.054307159	-4.693887438	2.620039685
C	-4.107407011	2.326048996	2.812584253
C	3.4455876	-3.118843893	2.098338069
H	5.211948317	-0.82418671	2.130958362
H	-5.596029918	0.197782945	1.568190346
H	-2.669405718	0.716904633	3.260415489
H	1.90834149	-1.64365435	2.635402499
H	-6.497570658	1.603395223	0.999208242
H	6.288797325	-1.945974958	1.30479301
C	-3.168900473	3.502633853	3.151934403
C	2.437138166	-4.216395628	1.706546895
H	-5.038662419	4.919327232	1.47442613
H	4.832431123	-5.182352091	0.288859709
H	-2.160362751	3.371125788	2.748621469
H	1.578401272	-3.809261088	1.158334367
C	-5.638615197	0.959981838	0.778009636
C	5.469828539	-1.23491473	1.144678142
H	-7.948130083	0.686420463	-0.548655244
H	8.007477138	-0.478267545	0.670079005
H	-3.552653293	4.461190578	2.788123968
H	2.88844571	-5.006574561	1.098329016
H	-6.342960839	3.996331167	0.694535739
H	6.267829997	-4.131453954	0.311478575
H	4.612587412	2.806895759	1.399937845
H	-4.89883992	-2.794965392	-0.066218674
C	-5.369895194	4.476204518	0.530606571
C	5.417465868	-4.465571455	-0.295665049
H	2.398038708	5.063210099	1.971727994
H	-3.114139043	-5.407033958	0.144608836
C	-7.119072296	0.257303523	-1.106074198
C	7.299955184	0.128425727	0.110340058
H	-5.536567164	5.30597121	-0.172111603
H	5.831991546	-5.014189205	-1.153890356
C	-5.836751881	0.296671478	-0.550418893
C	5.931661667	-0.122657948	0.244086723
H	4.763587845	4.537105295	1.718196019
H	-5.268637187	-4.489002288	-0.412875779
H	2.1013474	3.32176126	1.915115481
H	-2.64368595	-3.799817854	0.712600072
C	4.598478539	3.791293211	0.927274077
C	-4.857916837	-3.563267066	-0.842290463
C	2.158910794	4.224156914	1.302064162
C	-2.634793022	-4.462803045	-0.154541082
H	8.801215612	1.343924789	-0.862087524
H	-8.306822488	-0.336358031	-2.814710346
C	-4.336422223	3.513601892	-0.090004459

C	4.576422453	-3.289783592	-0.833158023
H	5.449346743	3.857841864	0.239757594
H	-5.521348635	-3.266246548	-1.662059833
C	7.74003508	1.139363709	-0.738979729
C	-7.318676016	-0.308658222	-2.360921542
H	1.16848997	4.414472718	0.87598142
H	-1.593885492	-4.690788488	-0.407242868
H	-2.551281833	4.541695074	0.70931444
H	2.701528357	-4.443402638	-0.997041121
H	-5.89291987	2.774624005	-1.46247642
H	6.439376705	-2.225684138	-1.341845296
C	3.259428169	4.113458813	0.226043082
C	-3.419744864	-3.849820306	-1.331734716
C	-2.989213997	4.247177915	-0.250937091
C	3.376307044	-3.849693982	-1.622102007
C	-4.866068844	3.155058606	-1.497535412
C	5.485771983	-2.503610601	-1.805136738
H	-4.88015096	4.072092686	-2.104879959
H	5.718895008	-3.153891918	-2.660236016
H	3.796069005	6.201417323	0.298316537
H	-4.125568367	-5.74284461	-2.096059535
C	-6.211789046	-0.840777752	-3.01396944
C	6.783663309	1.880780562	-1.42648682
H	-2.263386717	3.629980485	-0.787592192
H	2.792751526	-3.054964894	-2.091441184
C	-4.955190214	-0.806894416	-2.403043222
C	5.425613356	1.606186444	-1.244098315
C	3.431929882	5.486723329	-0.454150466
C	-3.527082454	-4.89501639	-2.460755485
H	4.173029592	5.466210069	-1.262493695
H	-4.034302558	-4.504698821	-3.351948273
H	-3.143753473	5.165610823	-0.836796653
H	3.747178705	-4.507587954	-2.421635886
H	7.07647402	2.679895256	-2.102906959
H	-6.308570886	-1.301958135	-3.993818881
H	-4.245425387	2.425725017	-2.020690209
H	5.019350219	-1.599681514	-2.201657906
H	0.609224396	4.892285196	-1.195964902
H	-0.580663593	-4.582833449	-2.287901657
C	4.398630285	2.429635497	-1.969821269
C	-3.789971394	-1.457497764	-3.086745154
H	2.494355902	5.888272613	-0.850626392
H	-2.555338683	-5.297325446	-2.764510885
H	4.838258958	3.383385466	-2.284508469
H	-4.149849728	-2.192846289	-3.816131377
H	-0.059321381	3.256245991	-0.987630297
H	0.176490016	-3.231964746	-1.411162495

C	0.395146995	3.947072782	-1.707227351
C	-0.150306094	-3.581998006	-2.398873948
H	4.091180227	1.906558727	-2.88616744
H	-3.217003352	-0.705460239	-3.646665685
C	1.64425449	3.335237856	-2.367416026
C	-1.119899825	-2.587648114	-3.061181907
H	0.734873851	1.349662406	-2.619057773
H	-0.024022901	-0.833211076	-2.346894948
H	-0.356788263	4.153189017	-2.480273832
H	0.744256851	-3.682797964	-3.028940953
C	1.205411805	2.130004911	-3.220231738
C	-0.370712386	-1.264775974	-3.289928499
H	2.585213324	5.292530968	-2.853682057
H	-2.073907414	-4.075988334	-4.408037518
H	3.154422602	3.944583059	-3.858053498
H	-2.159277885	-2.405982738	-4.998137756
C	2.285458136	4.358327949	-3.331962967
C	-1.54062318	-3.124066856	-4.447428
H	0.466307918	2.472089638	-3.956535376
H	0.504488484	-1.453044463	-3.926656936
H	2.03947453	1.687942187	-3.777374805
H	-0.9865124	-0.516607919	-3.801198392
H	1.541866113	4.61168536	-4.101360248
H	-0.631695138	-3.283005778	-5.046691304
H	-0.544134685	-0.597836299	1.51092636
C	-0.132347848	0.343849791	5.052443942
C	-0.343698984	-0.857577695	5.730042301
C	-0.615556429	-2.007898307	4.996787835
C	-0.660138672	-1.940473871	3.598580488
N	-0.462588853	-0.7673833	2.959088488
C	-0.209059425	0.367799343	3.659356076
H	0.081310414	1.262846489	5.592011856
H	-0.296578671	-0.893384036	6.816711888
H	-0.786252218	-2.962104005	5.489804819
C	-0.907631775	-3.174751904	2.779240179
H	-0.225060942	-3.97964271	3.077101751
H	-1.932636018	-3.532341488	2.932084138
H	-0.775297858	-2.973978764	1.712880309
C	-0.044703451	1.636138365	2.875448844
H	0.802119272	1.572667083	2.189879242
H	-0.926527319	1.810748771	2.24884147
H	0.102161698	2.491455805	3.542269813

Supplementary Table 65 | Cartesian coordinate of the product complex for protonation of the bridging dinitrogen ligand in 1 by LutH+ [II-Lut(BR)]. Units are presented in Å.

SCF energy (in toluene) = -4289.79412988 hartree

ZPE = 1.442022 hartree

Atom	X	Y	Z
Mo	2.590250588	-0.450952706	0.084540188
Mo	-2.475131633	-0.792813423	0.390298689
P	-3.127700808	-0.91979797	-2.100287945
P	3.078677683	1.960376976	0.948044322
N	4.895584048	-0.228076257	-0.114872935
N	-4.628436304	-1.506728999	0.476563923
P	-2.901047841	-0.994441756	2.907935941
P	3.368249838	-2.78643514	-0.902000557
N	0.721567174	-0.355281395	0.296392812
N	-0.496723315	-0.042314692	0.403662612
N	2.283367976	0.660670779	-2.874733902
N	2.389187194	0.268983397	-1.820365417
N	2.831324809	-1.196375522	1.973911328
N	2.984273546	-1.613852753	3.013000529
N	-3.264853619	2.26122789	0.457658704
N	-3.030263025	1.145515423	0.422175099
N	-1.791763151	-2.689766688	0.351450792
N	-1.404647113	-3.760146188	0.320973226
H	-3.497228903	1.551543664	-5.089803732
H	1.94152665	5.506706854	-0.102534342
H	3.449397336	4.695192276	-0.535094463
H	-4.743131467	1.098334379	-3.926254847
C	-3.827428381	0.746344518	-4.416900033
C	2.675584481	4.763769578	0.239832946
H	1.99688853	2.990413054	-1.726547167
H	-3.317201078	2.136534337	-2.103434532
H	3.136569562	5.144761405	1.153824687
H	-4.082351768	-0.113881064	-5.040326173
H	0.586880077	3.904285312	-1.179511457
H	-2.133084002	2.546127744	-3.348678231
C	-2.431827373	1.767744294	-2.631241326
C	1.265753749	3.081357756	-0.915154229
H	-1.081672156	0.957158997	-4.751589891
H	0.071066998	4.310461251	1.084896492

C	-2.699725375	0.460583451	-3.401230039
C	1.92934862	3.42431756	0.433821182
H	4.202342336	2.435185271	-1.124077439
H	-4.996912755	0.512115571	-1.597858605
H	-1.624028852	1.651752467	-1.901380758
H	0.688996728	2.152208986	-0.877545566
H	-5.592836558	-0.774369045	-2.648357372
H	5.176421655	3.097956228	0.187611111
C	-1.402879696	0.093902417	-4.150879144
C	0.812881711	3.607367272	1.483807257
H	-3.208688028	-2.038835413	-5.148528942
H	3.256233001	4.354478704	3.097218311
H	-0.585650054	-0.145427802	-3.458585089
H	0.290170397	2.668186587	1.711003114
C	-4.952123273	-0.571739967	-1.781250404
C	4.593225155	2.221334951	-0.118298082
H	-7.450053909	-1.539959174	-1.394570747
H	7.283158822	2.158000541	-0.368055119
H	-1.532378137	-0.747516569	-4.839262092
H	1.183234368	4.022637018	2.427079239
H	-4.815354698	-2.201242196	-4.404683234
H	4.848904686	4.07072961	2.357889316
H	4.401054294	-0.826621446	-2.888822159
H	-5.0027343	0.896412556	2.007642616
C	-3.795369969	-2.605548547	-4.418594678
C	4.112953766	3.674134422	3.068664745
H	2.670592087	-2.765786285	-4.658866276
H	-3.623272826	2.483522217	4.361472799
C	-6.80740685	-1.733472252	-0.538997361
C	6.862339671	1.157002509	-0.314003988
H	-3.864636096	-3.637913	-4.792466881
H	4.57903153	3.699054198	4.064144125
C	-5.479860827	-1.2983335	-0.569982345
C	5.481222986	1.003319464	-0.179986106
H	4.828402009	-1.819938832	-4.285369033
H	-5.668497927	1.559254964	3.507092038
H	1.938423979	-1.822158485	-3.353671277
H	-2.789816825	2.044434535	2.866668289
C	4.73401222	-1.822221991	-3.190357458
C	-5.180097979	0.679149803	3.063878852
C	2.436797915	-2.767706317	-3.584563949
C	-3.036320342	1.692042792	3.872188358
H	8.760677923	0.133255018	-0.477426369
H	-8.319769169	-2.758511437	0.618870171
C	-3.152315784	-2.629514583	-3.018302545
C	3.707966624	2.219445903	2.755251975
H	5.736385768	-1.990261966	-2.780193329

H	-5.890200759	-0.153089103	3.131069747
C	7.68252664	0.032667138	-0.376591122
C	-7.290499901	-2.408879713	0.579268726
H	1.72526321	-3.578784548	-3.405912978
H	-2.106638105	1.580614936	4.438386776
H	-1.073875828	-2.521174838	-3.761718785
H	1.725484938	2.4128283	3.708142941
H	-5.036086148	-3.393085956	-2.164541645
H	5.821338235	1.702504149	2.392845648
C	3.749810481	-2.945048277	-2.789491602
C	-3.876639526	0.395117655	3.844767873
C	-1.706094064	-3.158069496	-3.135456062
C	2.613928334	1.776459382	3.752300569
C	-3.965323205	-3.626783391	-2.161634447
C	4.960423845	1.337553363	2.964913758
H	-3.85075386	-4.631818379	-2.593255569
H	5.236952568	1.37596703	4.027871306
H	4.678726445	-4.233353262	-4.251071679
H	-4.936266112	0.821005074	5.680273762
C	-6.421434655	-2.622986846	1.646870276
C	7.085305138	-1.223912235	-0.305504224
H	-1.232063821	-3.255950139	-2.155251928
H	2.300712215	0.740797719	3.597426395
C	-5.104752788	-2.161257238	1.57656653
C	5.698445474	-1.329881767	-0.17923349
C	4.408920237	-4.282172897	-3.186248931
C	-4.280440004	0.030180312	5.287418791
H	5.334851042	-4.47610608	-2.629756068
H	-4.842691183	-0.910832352	5.341449366
H	-1.727414662	-4.157137311	-3.596129111
H	3.016854991	1.849878316	4.772539627
H	7.685520043	-2.129481132	-0.345792245
H	-6.754844083	-3.144011099	2.541203185
H	-3.625709646	-3.672413194	-1.124110414
H	4.796193194	0.288336207	2.706987951
H	1.869297698	-5.296241646	-2.116649137
H	-1.297801027	-0.06369919	5.497603129
C	5.057871246	-2.692904491	-0.09351153
C	-4.166341823	-2.377950602	2.738219656
H	3.74278914	-5.140692468	-3.062314155
H	-3.422875737	-0.040851935	5.964176395
H	5.745373292	-3.451083738	-0.486428425
H	-4.741647585	-2.560815606	3.654202077
H	0.756210224	-4.193951238	-1.264953407
H	-0.318188854	-0.018699195	4.013109164
C	1.528505873	-4.959228472	-1.13274876
C	-0.752065496	-0.679619179	4.775358429

H	4.882909741	-2.943128823	0.963228139
H	-3.565069982	-3.280103305	2.554303511
C	2.681611998	-4.467059779	-0.236538346
C	-1.626617169	-1.781921371	4.144738485
H	1.312602985	-3.476217637	1.168475139
H	-0.184564163	-2.142217183	2.529416226
H	1.046206538	-5.816262964	-0.643243463
H	0.081794256	-1.151753484	5.314862946
C	2.113368233	-4.221181565	1.174022886
C	-0.695155392	-2.694200211	3.32440002
H	4.240895849	-5.808457605	-1.078122327
H	-2.912161806	-2.079667359	5.929135614
H	4.551516414	-5.295238897	0.593094715
H	-2.836349029	-3.485202563	4.850373149
C	3.766415044	-5.561151809	-0.12515381
C	-2.261281894	-2.64451662	5.257617912
H	1.688012224	-5.163858462	1.545702716
H	0.069078373	-3.113068521	3.995733275
H	2.882687414	-3.909781013	1.888618263
H	-1.224873729	-3.536731207	2.867839778
H	3.290246247	-6.478283936	0.2499455
H	-1.455773038	-3.076668159	5.870070278
H	-0.564696022	0.9658088	0.622041161
C	-0.852633952	7.772790063	-3.533314328
C	-2.095895362	8.159214856	-3.03508955
C	-2.535558955	7.617710441	-1.82868177
C	-1.714444533	6.701988271	-1.155927395
N	-0.513279786	6.329484512	-1.638732756
C	-0.087143734	6.852982281	-2.80446584
H	-0.475165769	8.176586207	-4.470257648
H	-2.712002241	8.873891434	-3.578264884
H	-3.498709657	7.897488724	-1.407447733
C	-2.14484369	6.084972595	0.152718456
H	-1.385449072	6.258460005	0.926737872
H	-3.093883814	6.505991722	0.503381024
H	-2.273537302	4.998292001	0.053048037
C	1.268300044	6.403505851	-3.295648503
H	2.047810718	6.654842716	-2.563959662
H	1.29047617	5.313942501	-3.431159939
H	1.528093838	6.875199915	-4.25005274

Supplementary Table 66 | Cartesian coordinate of the transition state for protonation of the pyridine nitrogen atom in 1 by LutH⁺ [TS(PY)]. Units are presented in Å.

SCF energy (in toluene) = -4289.74661785 hartree

ZPE = 1.437743 hartree

imaginary frequency: 1314*i*

Atom	X	Y	Z
Mo	-2.008275243	-0.351635214	0.15699937
Mo	3.172117341	0.178217323	-0.136480457
P	3.955004592	-2.120868773	0.721394614
P	-2.408588027	0.819088555	2.511765679
N	-4.517466253	-0.690967679	0.2044093
N	5.43606197	0.405128314	-0.068823967
P	3.526194956	2.577304343	-0.988886663
P	-2.482179163	-1.412205276	-2.200741146
N	0.019660785	-0.174012001	-0.001017612
N	1.162944422	-0.026912678	-0.105445805
N	-1.473610607	-3.238301834	1.350053248
N	-1.686595535	-2.208839815	0.925492339
N	-1.945716143	1.526256918	-0.624479636
N	-1.702440701	2.549883272	-1.052190737
N	2.906498244	1.197435365	2.850745194
N	3.02943072	0.833422744	1.78135311
N	3.210847635	-0.507207599	-2.053574569
N	3.199973588	-0.907114985	-3.11619715
H	3.487544468	-4.262532966	3.940262779
H	-2.389953238	-0.228827529	6.268385088
H	-3.747355622	-0.413876968	5.161484983
H	4.731389932	-3.043998309	3.659149503
C	4.07217028	-3.760961554	3.154732769
C	-2.839828707	0.174726037	5.349464806
H	-2.423610105	-2.0099256	3.624998715
H	2.931984821	-1.279001424	3.461360965
H	-3.14020741	1.203609396	5.559439796
H	4.695309403	-4.520890557	2.6772556
H	-1.259819615	-1.90429548	4.952165688
H	1.743804832	-2.546994379	3.783821409
C	2.292141056	-2.028306006	2.983672172
C	-1.55025666	-1.461473701	3.987648271

H	1.479387186	-4.480258188	2.454805781
H	-0.059952227	0.089968028	5.456806174
C	3.096305652	-3.067841647	2.180035394
C	-1.818112567	0.040408683	4.201186486
H	-4.744645584	0.345460621	3.392591066
H	5.111706801	-0.970374563	2.494876638
H	1.566980779	-1.503846045	2.352677236
H	-0.728835588	-1.627355759	3.287052511
H	6.203473093	-2.210059011	1.877192196
H	-4.740775928	1.296606286	1.903635378
C	2.095524691	-4.102460276	1.626457527
C	-0.464909713	0.663763792	4.610680821
H	4.655287727	-5.291604543	0.689569761
H	-2.019973084	2.976195483	4.929248618
H	1.412985459	-3.661216268	0.889858118
H	0.274510584	0.611238786	3.802298658
C	5.489671327	-1.434786791	1.572604194
C	-4.262280009	0.454406836	2.413972447
H	8.140722774	-0.903329635	1.49191125
H	-4.637711663	-2.056667969	3.3325544
H	2.588629257	-4.966972233	1.16988098
H	-0.541229916	1.704039116	4.937242519
H	6.143616961	-4.339099314	0.884351238
H	-3.727090899	2.983730616	4.436804927
H	-2.360871354	-4.007294871	-0.703476386
H	4.805917735	2.616316564	1.708661293
C	5.365192128	-4.639190289	0.171126191
C	-2.71294105	3.283019212	4.142330524
H	0.382936085	-3.913605698	-2.355493238
H	2.787566415	4.981283631	1.894673386
C	7.580299602	-0.251948969	0.825580005
C	-4.641988495	-2.035283243	2.24576767
H	5.855971472	-5.248007792	-0.602530036
H	-2.69818722	4.382890593	4.114130493
C	6.192051312	-0.388980993	0.744953092
C	-4.41371787	-0.808951188	1.596192978
H	-1.646635657	-5.145838754	-1.843990138
H	5.088146373	4.332783442	2.02002462
H	-0.039455081	-2.699020764	-1.130125712
H	2.247319041	3.319449113	1.609530374
C	-2.272013233	-4.244938066	-1.762700376
C	4.992471418	3.578162191	1.225622622
C	-0.143950752	-2.963295918	-2.185846139
C	2.575699779	4.236538151	1.112953671
H	-5.083123346	-4.135265433	2.014706934
H	9.309365031	0.825072347	0.099104068
C	4.686170337	-3.432978801	-0.508241329

C	-2.338035953	2.757471563	2.74087783
H	-3.264265637	-4.513613395	-2.148488198
H	5.959453573	3.525268367	0.712101858
C	-4.878857663	-3.19028918	1.517845245
C	8.228822479	0.70827984	0.052668369
H	0.37183164	-2.193537405	-2.772574887
H	1.742311648	4.61798457	0.514910917
H	2.779881408	-4.479339309	-0.901216315
H	-0.14977304	2.805907377	3.052677093
H	6.660144474	-2.495363725	-0.801051459
H	-4.36997634	3.319824942	2.101487246
C	-1.621797105	-3.125710289	-2.602692563
C	3.85298742	4.011281624	0.274485542
C	3.567003235	-3.945585604	-1.441513791
C	-0.907145069	3.213057734	2.376212755
C	5.757938349	-2.734444002	-1.375469073
C	-3.3300067	3.428424683	1.766031383
H	6.057779786	-3.419799773	-2.181613421
H	-3.116124281	4.506759992	1.73965338
H	-1.166783274	-4.602511535	-4.09713969
H	4.542837265	6.052956965	0.444288333
C	7.458392855	1.514913788	-0.781593813
C	-4.81463021	-3.113083164	0.112167139
H	3.104029386	-3.135260702	-2.010627254
H	-0.635811101	2.923665904	1.356062074
C	6.071298084	1.350703082	-0.820724919
C	-4.587204803	-1.902239789	-0.511882967
C	-1.659418635	-3.619578184	-4.062218949
C	4.285487427	5.348062121	-0.360278095
H	-2.681272442	-3.759789307	-4.433125322
H	5.177932174	5.242980775	-0.990543492
H	4.004388405	-4.649225637	-2.165666168
H	-0.850202318	4.309733893	2.440386161
H	-4.904263834	-4.010154552	-0.493072159
H	7.921385951	2.277057626	-1.403746792
H	5.39329985	-1.815501052	-1.841261026
H	-3.246632411	3.057115985	0.745893623
H	-0.499437768	-0.957820081	-4.515462195
H	1.629234173	5.05551842	-1.578401178
C	-4.342665002	-1.805590965	-1.985218668
C	5.231091091	2.227912005	-1.711931778
H	-1.11579631	-2.969491599	-4.754189033
H	3.494670085	5.815546086	-0.955124229
H	-4.647502071	-2.720890763	-2.503161745
H	5.786957657	3.135256177	-1.978778548
H	-0.549627858	0.471807203	-3.468692132
H	0.722715922	3.531243699	-1.433023828

C	-1.078749984	-0.062664636	-4.26644265
C	1.375501908	4.120431456	-2.088005315
H	-4.894344757	-0.966783112	-2.427270479
H	5.024705762	1.693559913	-2.651089773
C	-2.528519573	-0.386133744	-3.857524352
C	2.619253547	3.327181192	-2.534415047
H	-2.77801342	1.562019652	-2.851362692
H	1.489820522	1.466833281	-2.827825085
H	-1.081883934	0.583220249	-5.156720549
H	0.787126776	4.387793736	-2.97794447
C	-3.262532308	0.945310199	-3.605213992
C	2.130438981	2.147653076	-3.397444884
H	-2.75156608	-1.916156814	-5.452955718
H	3.891668029	5.10602906	-2.919450281
H	-4.284526298	-1.416102987	-4.719245984
H	4.363368021	3.664803129	-3.841297768
C	-3.285432541	-1.072548166	-5.018724307
C	3.510455011	4.217168481	-3.428564051
H	-3.269569159	1.518793599	-4.543745493
H	1.548002564	2.544728178	-4.242204992
H	-4.311888622	0.795553014	-3.321214054
H	2.955288867	1.563846268	-3.819760522
H	-3.429249284	-0.335732155	-5.822381027
H	2.911680946	4.56335313	-4.284210543
H	-5.52407758	0.15099868	-0.161470683
C	-8.986672738	2.115345813	-0.943808229
C	-9.003609756	0.846658544	-0.363629133
C	-7.803663073	0.185126365	-0.105415388
N	-6.617267165	0.780124038	-0.415665842
C	-6.578073379	2.016469958	-0.97112513
C	-7.764238697	2.703021837	-1.249181408
H	-9.918179482	2.637483439	-1.152269561
H	-9.941667342	0.36073191	-0.109806289
H	-7.710778697	3.691311358	-1.698881833
C	-5.242801128	2.634324006	-1.262270268
H	-5.092139953	3.524169505	-0.637652093
H	-4.425460873	1.937583938	-1.073337355
H	-5.187003377	2.961951362	-2.307568133
C	-7.788988239	-1.18504005	0.509961465
H	-7.204697861	-1.205092282	1.437773181
H	-8.80911646	-1.507107915	0.739496547
H	-7.339620705	-1.921881835	-0.166858851

Supplementary Table 67 | Cartesian coordinate of the product complex for protonation of the pyridine nitrogen atom in 1 by $LutH^+$ [II-Lut(PY)]. Units are presented in Å.

SCF energy (in toluene) = -4289.76769226 hartree

ZPE = 1.441601 hartree

Atom	X	Y	Z
Mo	-2.042672051	-0.591944169	0.205448605
Mo	3.125715073	0.2184401	-0.076894958
P	4.04835294	-2.038031725	0.760056994
P	-2.337587734	0.654659753	2.628047179
N	-4.292557075	-0.747668009	-0.021783706
N	5.379445205	0.559020699	-0.109471295
P	3.321312824	2.641963239	-0.922070289
P	-2.372036119	-1.660032687	-2.267604159
N	0.02768783	-0.283023791	0.051669931
N	1.167241197	-0.09038185	-0.019268718
N	-1.263144392	-3.482539033	1.262026528
N	-1.573800135	-2.466703494	0.878013084
N	-2.230503418	1.321280176	-0.458711943
N	-2.202071625	2.389395188	-0.834282414
N	2.916742244	1.248901094	2.916976921
N	3.01407523	0.879848316	1.848813181
N	3.167783975	-0.455263928	-2.003152105
N	3.166770038	-0.839714584	-3.070135586
H	3.827498394	-4.122084923	4.040076766
H	-2.472316777	-0.623920652	6.295334093
H	-3.807811411	-0.541288152	5.146415674
H	5.019717571	-2.884195118	3.641191664
C	4.348756439	-3.633459032	3.203719524
C	-2.84561651	-0.084404582	5.412614792
H	-2.50447972	-2.215192609	3.612803122
H	3.114948142	-1.214317682	3.561679676
H	-3.03208079	0.946983171	5.717981579
H	4.959480425	-4.395259144	2.713056809
H	-1.344002639	-2.216229768	4.944072272
H	1.985051244	-2.522728682	3.92224883
C	2.485627605	-1.984753708	3.103827617
C	-1.60860584	-1.720705426	3.998352053
H	1.768517112	-4.484060527	2.641362808
H	-0.065444514	-0.288112923	5.553317269

C	3.29633326	-2.996478065	2.271257481
C	-1.811265382	-0.21841423	4.27645954
H	-4.744334223	0.044841978	3.208635531
H	5.245897736	-0.820340864	2.462089293
H	1.7187955	-1.486473582	2.501035304
H	-0.790500432	-1.89500088	3.295303085
H	6.354445953	-2.018141072	1.795606394
H	-4.597088415	1.097093448	1.801037319
C	2.315025155	-4.079108714	1.777739431
C	-0.43307643	0.325609563	4.718390794
H	4.843630409	-5.186244698	0.721698475
H	-1.804172549	2.706437444	5.094351599
H	1.568225285	-3.672718575	1.084781909
H	0.308621517	0.257242405	3.912922666
C	5.595060675	-1.275171624	1.523758952
C	-4.137966478	0.244418883	2.314597282
H	8.220007546	-0.641506066	1.283373734
H	-4.53645251	-2.336706867	2.998622729
H	2.818467398	-4.920905601	1.291871072
H	-0.45734465	1.360524601	5.068782433
H	6.312523492	-4.191429689	0.838089805
H	-3.542752509	2.767219213	4.729129923
H	-2.643145866	-4.172089892	-0.639971628
H	4.741517421	2.74211407	1.700230408
C	5.508798407	-4.51794137	0.165685839
C	-2.546308277	3.054722065	4.372856712
H	-0.015605458	-4.655795879	-2.385227226
H	2.654606371	5.064119375	1.970999122
C	7.59383377	-0.014008061	0.653873854
C	-4.575654498	-2.212604154	1.919294689
H	5.978864366	-5.118929013	-0.626661394
H	-2.508719117	4.154102718	4.382878801
C	6.209469764	-0.205896038	0.656171092
C	-4.085887649	-0.961859457	1.379011289
H	-2.174882734	-5.455979888	-1.756958504
H	4.978453617	4.468931272	1.991339823
H	-0.139734198	-3.341523343	-1.202953582
H	2.183557888	3.369598063	1.779354618
C	-2.634644475	-4.459255882	-1.69129075
C	4.867995103	3.707904189	1.20528533
C	-0.335570294	-3.613011601	-2.244515163
C	2.430657886	4.282224652	1.230318566
H	-5.562130086	-4.076358429	1.579833709
H	9.230075454	1.132059187	-0.172358432
C	4.766555107	-3.33537414	-0.489627141
C	-2.290254032	2.584377487	2.924283771
H	-3.67022642	-4.563181061	-2.033558579

H	5.80736987	3.687794196	0.640967433
C	-5.15019563	-3.170806393	1.141899998
C	8.154188267	0.972099153	-0.154689997
H	0.29814762	-2.987273622	-2.880377225
H	1.541243241	4.600269667	0.678741401
H	2.873962644	-4.435607139	-0.792916766
H	-0.089727467	2.612911121	3.059066549
H	6.701754277	-2.351323069	-0.866579126
H	-4.383100371	3.112397441	2.494302469
C	-1.835833261	-3.500120227	-2.599597339
C	3.662084114	4.092663982	0.317872094
C	3.620640116	-3.882333422	-1.369836473
C	-0.895381969	3.06304683	2.467798417
C	5.781990767	-2.612099897	-1.402523241
C	-3.381261854	3.283712281	2.080639323
H	6.063517674	-3.290941073	-2.220341165
H	-3.201995547	4.36776237	2.119943687
H	-1.791200198	-5.073987836	-4.071629394
H	4.291664996	6.157171147	0.429011006
C	7.30556626	1.748481919	-0.940353649
C	-5.159765423	-2.995253364	-0.284797752
H	3.108556006	-3.086607479	-1.917920897
H	-0.710134698	2.827964527	1.414467591
C	5.926048473	1.528739273	-0.897494008
C	-4.691715637	-1.855792526	-0.850858792
C	-2.058732191	-4.007628218	-4.040180079
C	4.006149012	5.43791556	-0.352738105
H	-3.108830051	-3.932738286	-4.349786418
H	4.857157343	5.359559293	-1.041492469
H	4.042895117	-4.575851596	-2.11208184
H	-0.823445254	4.153846996	2.587969253
H	-5.460305224	-3.81714011	-0.928073868
H	7.701488365	2.527921036	-1.586785544
H	5.377044891	-1.702758164	-1.852969856
H	-3.385681974	3.000185766	1.028534874
H	-0.306241132	-1.86278518	-4.701348008
H	1.269803086	5.027333548	-1.39217853
C	-4.289585096	-1.68558774	-2.275512163
C	4.998518486	2.368994144	-1.738742062
H	-1.438948492	-3.502214242	-4.785056007
H	3.161035116	5.87364878	-0.894812197
H	-4.700176043	-2.473254073	-2.91578752
H	5.498757327	3.30144844	-2.027791125
H	0.188167212	-0.671413167	-3.483877662
H	0.457032546	3.461022227	-1.167570289
C	-0.508429426	-0.862633581	-4.308628747
C	1.029549627	4.07462803	-1.874488803

H	-4.610659675	-0.713787525	-2.675036393
H	4.769673327	1.830088683	-2.67006316
C	-1.978242738	-0.657655784	-3.886792461
C	2.27706648	3.33877901	-2.403113861
H	-1.393899666	1.189216624	-2.865478537
H	1.218050966	1.428454649	-2.602362689
H	-0.269041166	-0.148102289	-5.10936469
H	0.364994983	4.30088481	-2.720791125
C	-2.152135972	0.837311044	-3.567584997
C	1.78515563	2.130418766	-3.222345531
H	-2.869331466	-2.031364637	-5.390933777
H	3.419792799	5.179523343	-2.885518165
H	-3.969733024	-0.761070074	-4.830031118
H	3.919521176	3.752566518	-3.814680272
C	-2.922866247	-0.992074137	-5.062786351
C	3.06157243	4.262494922	-3.359982978
H	-2.039241298	1.413071235	-4.497687602
H	1.126353208	2.491401407	-4.025820695
H	-3.140167159	1.076335552	-3.157817107
H	2.60437615	1.581003247	-3.698077918
H	-2.641781233	-0.364541786	-5.921021422
H	2.395704379	4.560908591	-4.183262266
H	-4.793199413	0.124380653	-0.278542667
C	-8.994510301	2.853000001	-0.835004935
C	-8.934959985	1.732257634	-0.010658696
C	-7.740266729	1.004971185	0.070420612
N	-6.637566238	1.356004912	-0.628309176
C	-6.69971547	2.435934071	-1.43823322
C	-7.860926597	3.209012491	-1.561832518
H	-9.910291644	3.435968818	-0.914116733
H	-9.800448767	1.415734935	0.566682448
H	-7.868224414	4.071924806	-2.223518832
C	-5.468499176	2.774115179	-2.239018816
H	-5.593569333	3.715425282	-2.785223153
H	-4.583654576	2.864394885	-1.598463646
H	-5.26078367	1.986208472	-2.976796189
C	-7.64880436	-0.212198453	0.956521976
H	-6.991489002	-0.026587981	1.817070648
H	-8.633827906	-0.492009271	1.34581122
H	-7.237458167	-1.068303041	0.408814819

Supplementary Table 68 | Cartesian coordinate of the reactant complex for coordination of dinitrogen to XXII. Units are presented in Å.

SCF energy (in toluene) = -2833.52870426 hartree

ZPE = 0.670416 hartree

Atom	X	Y	Z
Mo	-0.166437016	0.495192316	0.319616191
P	0.401533206	-0.965165018	2.397683631
N	1.886034836	1.13740604	0.806901809
P	0.368551253	1.996082139	-1.684918994
N	-1.31748157	1.490038416	1.129143317
H	-0.939310269	-1.940819456	5.876810026
H	0.610550112	-1.201786857	5.462383292
C	-0.183904798	-1.857432111	5.082894672
H	-0.886535701	0.741558751	4.689852214
H	0.232736032	-2.857845756	4.929879572
H	-2.367826877	-0.191703436	4.897138879
C	-1.557040893	0.031759696	4.190564461
H	-2.672666841	-2.428570629	4.148041114
C	-0.867597979	-1.297583205	3.817196604
H	0.684236574	1.20695687	3.412088597
H	-2.005339018	0.522143615	3.320564064
H	1.981919352	0.173291686	4.019450127
C	-1.957264306	-2.272747182	3.328252354
H	2.329841135	-2.490684008	4.305886879
H	-2.500653967	-1.877355722	2.465563127
C	1.423140324	0.450436435	3.119357154
H	3.955432787	1.404843863	3.467688342
H	-1.560777157	-3.249708056	3.042806914
H	3.384465987	-1.522833065	3.250189887
H	-0.196154983	3.673085418	0.795630583
C	2.74412401	-2.412585284	3.296367188
H	-1.347427987	5.387345775	-1.382455058
C	3.62458281	1.481347773	2.435574383
H	3.396844858	-3.283283036	3.143103101
C	2.343250873	1.041836218	2.093053316
H	0.383976524	5.270996096	0.286650056
H	-1.966363994	3.748117802	-1.123633705
C	0.498441571	4.189669928	0.127630871
C	-1.22548015	4.336649662	-1.679916414

H	5.460146298	2.352416752	1.705661826
C	1.666111331	-2.412640336	2.193544081
H	1.522311183	3.928312714	0.418632678
C	4.459864469	2.007231867	1.453515768
H	-1.46336639	4.271607257	-2.746698715
H	0.526575718	-4.037143728	3.159950231
H	2.942178596	-1.297693325	0.771746156
C	0.217744989	3.892705286	-1.361734978
C	0.931859578	-3.770413255	2.178269937
C	2.368239149	-2.230264726	0.827146626
H	3.081667747	-3.055037081	0.690206603
H	1.062374803	5.780995504	-1.976377088
C	3.999561039	2.073980497	0.138907526
H	0.122204051	-3.801845094	1.442405464
C	2.712208521	1.632837197	-0.166818252
C	1.22514743	4.716071175	-2.193504595
H	2.264051659	4.493633341	-1.91941815
H	1.656275272	-4.54961927	1.903761876
H	4.632921081	2.461446625	-0.654537719
H	1.664718414	-2.281097823	-0.012048629
H	-2.211539456	2.256024064	-3.248706465
C	2.212522009	1.643804373	-1.587870529
H	1.116066683	4.583562003	-3.271525622
H	2.816263991	2.321447284	-2.201978479
H	-1.989847465	0.562328681	-2.758411612
C	-1.675851167	1.32727694	-3.473846212
H	2.340944688	0.638286583	-2.012929355
C	-0.14222532	1.520353089	-3.479434577
H	0.391967566	-0.590763363	-3.069346882
H	-1.99382257	1.001246709	-4.473857966
C	0.538093481	0.177419449	-3.833505806
H	-0.291506991	3.49630546	-4.459076625
H	1.32275138	2.75159954	-4.582307066
C	0.24499264	2.547267179	-4.560985228
H	0.089129156	-0.199979101	-4.762341591
H	1.611048852	0.299829947	-4.028191512
H	-0.023629777	2.130076753	-5.541713902
S	-1.217834038	-2.438970717	-0.96159332
O	-0.123841572	-3.314752271	-1.390088596
O	-1.9508965	-2.727935039	0.274958494
O	-0.77440974	-0.9234811	-1.056252527
C	-2.48644718	-2.429377482	-2.335122533
F	-3.425704471	-1.51194419	-2.068146939
F	-1.898463454	-2.130401773	-3.498474074
F	-3.045495702	-3.633480791	-2.409118804
N	-5.591815474	2.087873758	1.201388853
N	-4.491273542	2.089619161	1.320142287

```
 H    -2.268086065    1.807945783    1.344789289
---------------------------------------------------
```

Supplementary Table 69 | Cartesian coordinate of the transition state for coordination of dinitrogen to XXII. Units are presented in Å.

SCF energy (in toluene) = 2833.52111384 hartree

ZPE = 0.671330 hartree

Imaginary frequency: 73*i*

Atom	Coordinates (Angstroms) X	Y	Z
Mo	-0.179897479	0.598195977	0.369873802
P	0.389240015	-0.923617925	2.439437557
N	1.902994268	1.068236769	0.742476868
P	0.363182843	2.070693848	-1.679404289
N	-0.96571857	1.785008657	1.358886644
H	-0.998043382	-1.764517736	5.942828017
H	0.55181017	-1.024447763	5.526822561
C	-0.228081347	-1.704937981	5.160856729
H	-0.937555865	0.90513188	4.536327974
H	0.202287523	-2.705699548	5.056815791
H	-2.36104106	-0.058306523	4.931454078
C	-1.606753234	0.124602107	4.154153018
H	-2.71984976	-2.306806584	4.178444007
C	-0.889336527	-1.20929288	3.85833174
H	0.765181639	1.281365248	3.365737534
H	-2.133576111	0.504403662	3.27289057
H	2.035909346	0.217003787	3.990555827
C	-1.959576628	-2.217810728	3.389652613
H	2.239298779	-2.415872916	4.437675802
H	-2.454250579	-1.891067692	2.470380001
C	1.46630867	0.482442387	3.094349297
H	4.0601848	1.282194178	3.335868515
H	-1.554918136	-3.215529297	3.206327818
H	3.35444479	-1.519144995	3.381917137
H	0.284292844	3.727036432	0.846124163
C	2.684030854	-2.385382648	3.439156503
H	-1.06781375	5.566652374	-1.222276817
C	3.705302493	1.347244533	2.311001168
H	3.31375852	-3.280048536	3.335966802
C	2.388705899	0.983171656	2.020650119
H	0.798360802	5.327298322	0.278082337
H	-1.741519447	3.979606978	-0.813083415

C	0.880554964	4.247249273	0.092174271
C	-1.049732274	4.500680075	-1.487596123
H	5.576948029	2.056175654	1.500560203
C	1.644135358	-2.397115394	2.297679642
H	1.934205929	3.977929307	0.231350214
C	4.547163607	1.777485387	1.288186946
H	-1.43500201	4.413655985	-2.508993795
H	0.473439868	-3.985932691	3.280462595
H	3.009920626	-1.348400591	0.910980697
C	0.393108629	3.971789026	-1.347806215
C	0.894997547	-3.746508271	2.298299048
C	2.409779126	-2.263880088	0.960949512
H	3.108395036	-3.108826123	0.883560955
H	1.350388403	5.793163842	-1.994741064
C	4.051545124	1.840787487	-0.012819236
H	0.096456239	-3.785844492	1.551450018
C	2.724952718	1.490700982	-0.267539616
C	1.342016703	4.736597119	-2.296839534
H	2.37717336	4.379253226	-2.223408893
H	1.615432109	-4.538977904	2.053429022
H	4.683358152	2.163754593	-0.835725837
H	1.751305304	-2.317591475	0.089420893
H	-2.313619471	2.354307789	-3.002791117
C	2.168152879	1.543357819	-1.664357594
H	1.03792309	4.701240388	-3.343497616
H	2.806661277	2.158939007	-2.308027854
H	-2.046020224	0.633579118	-2.649570532
C	-1.796024902	1.446617145	-3.336370411
H	2.175824906	0.529368992	-2.086720837
C	-0.269642528	1.652489753	-3.455684277
H	0.318152017	-0.463115586	-3.177192187
H	-2.198412551	1.186658836	-4.325267018
C	0.396033001	0.33628007	-3.917275074
H	-0.513146368	3.659957676	-4.356562918
H	1.085981126	2.926183028	-4.646406401
C	0.015756295	2.717556946	-4.532252002
H	-0.116153509	-0.007303502	-4.826008279
H	1.450904077	0.484124634	-4.181526549
H	-0.34104273	2.32620791	-5.495438937
S	-0.960021492	-2.4326099	-0.971404531
O	0.136858196	-3.334160534	-1.328761073
O	-1.768682346	-2.693210192	0.225801811
O	-0.490502063	-0.933917615	-1.036044788
C	-2.157046893	-2.474209058	-2.408129534
F	-3.127201463	-1.565324079	-2.22134658
F	-1.52062678	-2.203374501	-3.55172035
F	-2.695098811	-3.688213496	-2.469433896

N	-3.118615827	0.165043661	0.164969935
N	-4.216952629	0.11818306	0.299266111
H	-1.749265864	2.280649503	1.788778385

Supplementary Table 70 | Cartesian coordinate of the reactant complex for coordination of dinitrogen to XI. Units are presented in Å.

SCF energy (in toluene) = -2833.09593003 hartree

ZPE = 0.658862 hartree

Atom	X	Y	Z
Mo	-0.090846858	0.515515465	0.350721653
P	0.503696831	-0.938863679	2.368875807
N	1.984347419	1.161492734	0.808302074
P	0.428787188	2.013627905	-1.62789221
N	-1.037481938	1.511163978	1.28777066
H	-0.793426309	-1.953547806	5.868777711
H	0.732294809	-1.16188553	5.453877486
C	-0.039387027	-1.845614907	5.074916499
H	-0.802500982	0.717493096	4.696219142
H	0.416816907	-2.830360404	4.927255188
H	-2.281580729	-0.234900808	4.852946906
C	-1.454708003	0.00827203	4.170147397
H	-2.522085709	-2.473632006	4.111684295
C	-0.728733573	-1.305857322	3.805104382
H	0.766248175	1.214525813	3.391244484
H	-1.872106712	0.508748158	3.290609089
H	2.085825759	0.212208835	4.025549191
C	-1.793996543	-2.3042444	3.304313659
H	2.564897853	-2.363516577	4.226739793
H	-2.33842605	-1.919190484	2.435554256
C	1.531141142	0.4769902	3.117824516
H	4.051507904	1.472039844	3.470237439
H	-1.374006053	-3.275763678	3.03009089
H	3.532487952	-1.407995054	3.0800297
H	-0.226726527	3.703987153	0.78270022
C	2.924815392	-2.314376261	3.192745549
H	-1.249255618	5.434094009	-1.406047691
C	3.721711726	1.540794262	2.436593293
H	3.594206743	-3.173413451	3.036861784
C	2.445213228	1.081986621	2.095940322
H	0.415083907	5.298369179	0.308945183
H	-1.895390442	3.793856495	-1.213240584
C	0.510238538	4.213010312	0.155374077
C	-1.125578402	4.387679935	-1.72124742

H	5.548226846	2.434448486	1.706009568
C	1.780798442	-2.379660834	2.161854846
H	1.511436064	3.921209843	0.49405193
C	4.553308267	2.073010126	1.454461828
H	-1.31057369	4.34881412	-2.800711609
H	0.732268025	-3.996894023	3.244520969
H	2.904655784	-1.296947585	0.594100538
C	0.295014588	3.917783004	-1.345138032
C	1.096018901	-3.760718987	2.238859645
C	2.376157945	-2.246277466	0.740333527
H	3.104944923	-3.05509995	0.580087822
H	1.18417939	5.79425717	-1.945351552
C	4.090387464	2.124585688	0.138184994
H	0.268238702	-3.841872017	1.527706098
C	2.80999616	1.661832481	-0.162862828
C	1.346984502	4.723354188	-2.136207997
H	2.368529217	4.494133404	-1.806743321
H	1.840294715	-4.52800007	1.976947808
H	4.714947128	2.521442514	-0.658360341
H	1.595376581	-2.350143496	-0.023390534
H	-2.092235224	2.405673128	-3.296003214
C	2.289263806	1.670312297	-1.574914932
H	1.292041632	4.571872977	-3.217483492
H	2.888631653	2.335516178	-2.207368248
H	-1.982986646	0.719876408	-2.749404489
C	-1.604194357	1.443098014	-3.478516515
H	2.39391976	0.659060628	-1.991594698
C	-0.061587757	1.538594427	-3.431380372
H	0.242220839	-0.588360044	-2.92316341
H	-1.906635893	1.105878997	-4.481241211
C	0.506002552	0.127646158	-3.708811703
H	-0.023631502	3.479626999	-4.477537855
H	1.532613158	2.616412548	-4.511654085
C	0.44181156	2.489296623	-4.532836451
H	0.065652637	-0.247317354	-4.643866636
H	1.594429476	0.133851832	-3.8505577
H	0.182891786	2.065001165	-5.514499989
S	-1.926991501	-2.13705718	-0.644058947
O	-0.943064314	-3.160586562	-0.239920235
O	-3.175783088	-2.027786108	0.122087024
O	-1.278860205	-0.778542643	-0.961384036
C	-2.43743701	-2.615427015	-2.371816105
F	-3.277134075	-1.704002024	-2.885386897
F	-1.359887025	-2.703018362	-3.172451406
F	-3.050736389	-3.804594342	-2.348887171
N	-4.169274275	1.451549002	-0.293987996
N	-4.472260442	1.023067971	0.68092562

Supplementary Table 71 | Cartesian coordinate of the transition state for coordination of dinitrogen to XI. Units are presented in Å.

SCF energy (in toluene) = -2833.08497954 hartree

ZPE = 0.659524 hartree

Imaginary frequency: 107*i*

Atom	X	Y	Z
Mo	2.492520234	0.539474458	0.38796536
P	3.076442863	-0.895522673	2.443344282
N	4.596948531	1.081767523	0.760714246
P	3.003995907	2.012261367	-1.6275705
N	1.721135995	1.641960782	1.378025992
H	1.661204676	-1.8424376	5.929188772
H	3.269089656	-1.213884577	5.545593638
C	2.432029329	-1.801055983	5.145222765
H	1.960349161	0.829303868	4.834681251
H	2.777327728	-2.826247598	4.976908097
H	0.382293092	0.044840674	4.95201009
C	1.237559389	0.214736792	4.281984495
H	-0.093264165	-2.137273464	4.13854386
C	1.821265828	-1.159721431	3.884031015
H	3.479752224	1.275721314	3.378272492
H	0.888618676	0.787227881	3.416002356
H	4.765096859	0.219653164	4.004003445
C	0.666024623	-2.032901346	3.349043723
H	4.91068307	-2.50241632	4.405911562
H	0.192032035	-1.580348936	2.472489043
C	4.191162854	0.485128232	3.108548537
H	6.77737336	1.314011504	3.337478938
H	0.988505814	-3.036302692	3.058156176
H	6.019748953	-1.593076547	3.355988455
H	2.666640005	3.69158841	0.853281412
C	5.339016624	-2.452779403	3.399945563
H	1.471697993	5.480957187	-1.264988678
C	6.413822676	1.384180853	2.315472445
H	5.952826823	-3.354766011	3.259600921
C	5.099472065	1.001681699	2.032339413
H	3.262494935	5.285267134	0.32515701
H	0.813125131	3.869275959	-0.929979788

C	3.338919895	4.200460166	0.157334016
C	1.532151625	4.420148438	-1.549184789
H	8.265679526	2.140229405	1.498037011
C	4.273661443	-2.404079919	2.285014164
H	4.369736175	3.907621284	0.390470257
C	7.239886561	1.841791769	1.291723838
H	1.218870622	4.344980823	-2.596428903
H	3.039731061	-3.962238234	3.244735647
H	5.629280855	-1.37489881	0.876345044
C	2.97260225	3.916711683	-1.316495856
C	3.477753302	-3.72663086	2.268557717
C	5.00839925	-2.276852405	0.929551299
H	5.678195125	-3.141794867	0.813544236
H	3.899564202	5.769416031	-1.931801318
C	6.729474963	1.903855668	-0.004433249
H	2.680739351	-3.723418681	1.518393558
C	5.40961522	1.520884158	-0.248501451
C	3.967922209	4.703689669	-2.195184038
H	5.005762253	4.397472395	-2.009745573
H	4.166986472	-4.54474562	2.013872805
H	7.344803384	2.247771995	-0.831959347
H	4.316794319	-2.289860929	0.081386947
H	0.371297541	2.291518364	-3.020763744
C	4.837741533	1.558258373	-1.638902275
H	3.771442185	4.619717637	-3.266019849
H	5.453813038	2.184325898	-2.295389758
H	0.637284009	0.5633425	-2.709635827
C	0.900886205	1.396265661	-3.367778434
H	4.859950259	0.541424202	-2.053762098
C	2.431036353	1.600794503	-3.432388629
H	2.968637239	-0.514531929	-3.100072048
H	0.527274811	1.161138434	-4.375291493
C	3.084194185	0.266111218	-3.85744831
H	2.242459666	3.600489557	-4.358878515
H	3.839945765	2.846727539	-4.585955665
C	2.763200106	2.648840001	-4.511037226
H	2.586943725	-0.088471628	-4.771531648
H	4.148525298	0.388896022	-4.09766509
H	2.437990362	2.2567766	-5.486343439
S	1.60571777	-2.613241158	-1.033667952
O	2.648543907	-3.602079896	-1.351522801
O	0.743539798	-2.866293117	0.135547933
O	2.119660394	-1.176257384	-1.107593106
C	0.447740226	-2.646089289	-2.495657317
F	-0.516955778	-1.716664802	-2.355221636
F	1.114205261	-2.390573333	-3.634774437
F	-0.130567518	-3.848220864	-2.595805102

N	-0.30274668	0.126739695	0.05701126
N	-1.398103237	0.259662925	0.163431744

Supplementary Table 72 | Cartesian coordinate of the reactant complex for protonation of XXIV by LutH$^+$. Units are presented in Å.

SCF energy (in toluene) = -3160.33583475 hartree

ZPE = 0.819388 hartree

Atom	X	Y	Z
Mo	-0.668729425	0.76408586	0.649305018
P	0.17206536	-0.723862687	2.669594978
N	1.479063577	1.338569209	0.925011559
P	-0.220211709	2.286539423	-1.405337272
N	-1.38343517	1.915908552	1.674620274
H	-1.23111363	-1.812113895	6.119427867
H	0.312569987	-1.014167378	5.788849624
C	-0.443110618	-1.686934099	5.362786774
H	-1.176360017	0.908428792	4.94663945
H	0.013130099	-2.669399522	5.210416941
H	-2.618660719	-0.08595654	5.179290811
C	-1.830387923	0.173088635	4.458206827
H	-2.889808569	-2.293603767	4.268839877
C	-1.078713713	-1.119545677	4.077912605
H	0.422706644	1.509975818	3.568025467
H	-2.301346644	0.643291292	3.589357945
H	1.780884076	0.54234306	4.176038675
C	-2.111272563	-2.120946618	3.511573132
H	2.038256342	-2.136501257	4.719792374
H	-2.589694283	-1.742368441	2.602327946
C	1.169059667	0.754890503	3.292985354
H	3.740123947	1.684101145	3.413757963
H	-1.668830192	-3.088712577	3.262638531
H	3.136577223	-1.151047476	3.728408586
H	-0.178371144	3.832780191	1.147374867
C	2.518023886	-2.057064007	3.740874854
H	-1.687175409	5.752298915	-0.81655162
C	3.333926095	1.722926467	2.406470932
H	3.206106999	-2.908792349	3.645479361
C	2.023776953	1.294793384	2.17615696
H	0.210987542	5.496245825	0.633129045
H	-2.287719979	4.150116558	-0.342541201
C	0.345215567	4.430255881	0.395781919
C	-1.662887482	4.683018187	-1.071307434

H	5.133088814	2.503390537	1.501555373
C	1.530553624	-2.109289436	2.552889451
H	1.418184165	4.218584901	0.466779343
C	4.107675918	2.177553167	1.34014071
H	-2.121012683	4.573061951	-2.059771045
H	0.423506286	-3.794838567	3.448904148
H	2.909075114	-0.953873847	1.271792335
C	-0.204016016	4.182378085	-1.027368029
C	0.872359691	-3.504387529	2.492368615
C	2.356386089	-1.899389895	1.262862485
H	3.101103938	-2.705903217	1.206626745
H	0.697721139	6.048380767	-1.635913876
C	3.55130584	2.20146539	0.062984063
H	0.11344583	-3.576385194	1.708006219
C	2.23030621	1.783489197	-0.120697751
C	0.677747883	5.007627843	-1.990027041
H	1.717303245	4.656065796	-1.997864361
H	1.65428886	-4.242310723	2.265746509
H	4.129424264	2.540298265	-0.79251737
H	1.756459618	-1.958264413	0.353483019
H	-2.967392524	2.436526945	-2.541584292
C	1.594816961	1.792721872	-1.484988146
H	0.311876614	5.022699756	-3.016858585
H	2.18415419	2.396594106	-2.184416999
H	-2.554605752	0.707633015	-2.428762591
C	-2.408340751	1.618792682	-3.015196866
H	1.583047177	0.763024463	-1.867324994
C	-0.907825272	1.945411908	-3.183598044
H	-0.172071557	-0.133855482	-3.07002501
H	-2.850556068	1.451674033	-4.007389826
C	-0.186890468	0.709402503	-3.762436301
H	-1.312533891	3.989314719	-3.946453739
H	0.300910466	3.360018736	-4.370115947
C	-0.748125679	3.087460426	-4.206270109
H	-0.722759402	0.388294633	-4.666159763
H	0.84213218	0.944302753	-4.06344354
H	-1.141184839	2.731456458	-5.169105631
S	-0.65236241	-2.446209663	-0.921823905
O	0.520712251	-3.285204579	-1.196932589
O	-1.528599668	-2.808812004	0.209859514
O	-0.313460144	-0.947726714	-0.933741955
C	-1.741504034	-2.630106463	-2.428810402
F	-2.776552289	-1.766877127	-2.370648087
F	-1.045006393	-2.380730104	-3.547478606
F	-2.221119773	-3.872347755	-2.479812314
N	-2.627940924	0.010282803	0.263132999
N	-3.674169065	-0.35546906	0.108948682

H	-2.476093069	2.930681005	2.5010196
C	-2.705592502	4.415670054	3.964854992
N	-3.177832951	3.584166547	2.999112131
C	-4.479203271	3.532603998	2.613456065
C	-5.390261165	4.382279874	3.23950865
C	-4.949872125	5.251525131	4.237551175
C	-3.601817926	5.269457214	4.602281117
H	-3.238711272	5.940291276	5.37548072
H	-5.655416479	5.917121625	4.73021045
H	-6.433708958	4.354191196	2.939170582
C	-1.241606197	4.353442165	4.288662917
H	-0.977877208	5.127606984	5.014925917
H	-0.982480552	3.375735429	4.715794165
H	-0.630546711	4.491597568	3.388334988
C	-4.858101837	2.548649338	1.547371855
H	-5.849092379	2.780866456	1.146364631
H	-4.130890535	2.547328725	0.728072846
H	-4.888488262	1.529048058	1.955198224

Supplementary Table 73 | Cartesian coordinate of the transition state for protonation of XXIV by LutH$^+$. Units are presented in Å.

SCF energy (in toluene) = -3160.33093009 hartree

ZPE = 0.815146 hartree

Imaginary frequency: 1103*i*

Atom	X	Y	Z
Mo	-0.39555315	0.497911238	0.447894075
P	0.403464816	-0.981896572	2.49648311
N	1.737689151	1.074122439	0.762270434
P	0.09055375	2.017205525	-1.609439228
N	-1.139805493	1.66902953	1.452502621
H	-1.071890718	-2.056730301	5.914846763
H	0.467957967	-1.240473923	5.615037601
C	-0.267753024	-1.927104168	5.176514348
H	-1.019858604	0.659271222	4.713680998
H	0.204875224	-2.904393855	5.042314344
H	-2.451538511	-0.350086582	4.934756119
C	-1.654822526	-0.090585074	4.223673967
H	-2.682520092	-2.569160916	4.0353839
C	-0.88203168	-1.378454408	3.873067863
H	0.623427591	1.262367939	3.383834088
H	-2.123422442	0.363898786	3.34553403
H	1.969605247	0.302720553	4.025513461
C	-1.889505802	-2.397518841	3.293478343
H	2.22996746	-2.363161048	4.598453787
H	-2.353221777	-2.034105046	2.370715143
C	1.376354197	0.507416238	3.128337152
H	3.941053612	1.446142968	3.297833429
H	-1.431756417	-3.363502613	3.066095491
H	3.346667699	-1.378772483	3.626422925
H	0.111169841	3.576166935	0.936587895
C	2.733072414	-2.288110773	3.631049062
H	-1.377952181	5.487225326	-1.058751872
C	3.557562831	1.474144806	2.281410195
H	3.428055646	-3.136366578	3.559010737
C	2.25396373	1.040917961	2.026295802
H	0.498806138	5.234196919	0.419149487
H	-1.987459114	3.894457326	-0.57320895

C	0.639992092	4.168789609	0.185826292
C	-1.354233499	4.415707494	-1.302588445
H	5.375335312	2.248423742	1.40957366
C	1.776472093	-2.35723435	2.418156646
H	1.712966125	3.961074308	0.267370832
C	4.354280387	1.919534936	1.228179935
H	-1.803329841	4.297164143	-2.294175589
H	0.659061974	-4.043693222	3.299353122
H	3.178200099	-1.200192015	1.162639919
C	0.103473445	3.913773352	-1.24022459
C	1.132236857	-3.758639039	2.353226431
C	2.634948648	-2.150968775	1.149178222
H	3.38919087	-2.950050715	1.122926558
H	1.02029964	5.771229759	-1.846800371
C	3.826913782	1.931393568	-0.061415524
H	0.396137266	-3.845473074	1.549269338
C	2.510377095	1.512332493	-0.271095092
C	0.997492274	4.730289701	-2.199536935
H	2.035382383	4.373569497	-2.199512385
H	1.927157796	-4.491149813	2.15652451
H	4.423712253	2.263852567	-0.906455063
H	2.061999661	-2.227369783	0.223941273
H	-2.640492965	2.153570933	-2.784245445
C	1.903496871	1.515190999	-1.64851569
H	0.638324041	4.745934265	-3.228595419
H	2.507766944	2.116092331	-2.337514345
H	-2.227633223	0.426702297	-2.652252077
C	-2.073659299	1.333227342	-3.243617438
H	1.897870596	0.484541427	-2.027936537
C	-0.571919907	1.662366115	-3.393941797
H	0.172391196	-0.412947322	-3.264234755
H	-2.501766871	1.157902187	-4.240454034
C	0.164611715	0.428928679	-3.957757579
H	-0.97381301	3.7020031	-4.169289338
H	0.647158092	3.076757349	-4.570635858
C	-0.402815078	2.801569391	-4.418489898
H	-0.354050978	0.103292819	-4.869756635
H	1.197434845	0.668209403	-4.241655658
H	-0.782891194	2.439883237	-5.384325839
S	-0.338390531	-2.682309849	-1.081870301
O	0.855803144	-3.494067959	-1.340557149
O	-1.225073402	-3.052412803	0.036859388
O	-0.018939304	-1.171633651	-1.085715226
C	-1.400062641	-2.884067394	-2.607543921
F	-2.440217534	-2.027668786	-2.574656767
F	-0.683872169	-2.643096452	-3.713909173
F	-1.867722217	-4.130042125	-2.648812952

N	-2.356778843	-0.256458044	0.043369104
N	-3.394955573	-0.634540776	-0.12411686
H	-1.96574477	2.467333742	2.061218232
C	-2.3244737	4.115889753	3.613262969
N	-2.785232731	3.295538319	2.637860809
C	-4.075331656	3.338789538	2.225685058
C	-4.964490154	4.246659847	2.805472153
C	-4.514348329	5.1015441	3.80997272
C	-3.182240605	5.036003338	4.217957958
H	-2.802201463	5.689789834	4.99832478
H	-5.195163486	5.814297228	4.270655718
H	-5.996423067	4.274323231	2.466192292
C	-0.881111446	3.985999667	4.020038608
H	-0.606546944	4.775805709	4.725979021
H	-0.702488872	3.018553112	4.508009311
H	-0.213345689	4.048768373	3.152584852
C	-4.504363822	2.377416896	1.15173871
H	-5.511552689	2.617463866	0.797671404
H	-3.819397648	2.40043758	0.296041383
H	-4.517753684	1.347702792	1.53233633

Supplementary Table 74 | Cartesian coordinate of the product complex for protonation of XXIV by LutH⁺. Units are presented in Å.

SCF energy (in toluene) = -3160.33535581 hartree

ZPE = 0.818313 hartree

Atom	X	Y	Z
Mo	-0.712471133	0.726910071	0.704379023
P	0.136302394	-0.767925682	2.726204525
N	1.395012816	1.358607307	0.996830048
P	-0.3316328	2.340549388	-1.299352492
N	-1.460614769	1.867570067	1.763745194
H	-1.275841956	-1.897973745	6.147024136
H	0.246726151	-1.056936705	5.831867792
C	-0.484700319	-1.751058453	5.398267828
H	-1.295941503	0.837146158	4.951588735
H	-0.000942734	-2.721027733	5.249953083
H	-2.68865594	-0.212652477	5.218485557
C	-1.922183166	0.067397961	4.482773294
H	-2.905839982	-2.4233774	4.280330281
C	-1.128747199	-1.20145637	4.109158937
H	0.331513564	1.467055299	3.650879107
H	-2.436318615	0.508234262	3.623830765
H	1.707704663	0.519487508	4.237992045
C	-2.12264792	-2.235170469	3.532397679
H	2.008709715	-2.161030535	4.777725407
H	-2.601851806	-1.873885337	2.616743759
C	1.090354386	0.730600417	3.359035006
H	3.628051179	1.732713941	3.505489883
H	-1.65080354	-3.192613905	3.296769487
H	3.096655877	-1.133251909	3.818158337
H	-0.298283381	3.827846758	1.32517576
C	2.50060767	-2.05402189	3.807814866
H	-1.916405661	5.721828347	-0.561645598
C	3.226625383	1.775540498	2.49663966
H	3.210843201	-2.886802678	3.709027143
C	1.932979576	1.308296101	2.252050726
H	0.035086201	5.500138487	0.833835499
H	-2.451058726	4.092464307	-0.118271288
C	0.193317553	4.444946484	0.569205723
C	-1.859712803	4.662037044	-0.846500166

H	5.002553018	2.6338134	1.618014642
C	1.532224009	-2.114952249	2.604191081
H	1.273069251	4.261493046	0.612270272
C	3.989384298	2.277747388	1.444112445
H	-2.333476407	4.563531405	-1.828964705
H	0.453722654	-3.834462881	3.465438941
H	2.909430574	-0.908543407	1.37044587
C	-0.381717292	4.218371717	-0.846774273
C	0.911242285	-3.525421681	2.519277614
C	2.370476602	-1.861214095	1.330559341
H	3.127996172	-2.654646616	1.263142391
H	0.444668277	6.128582818	-1.41347849
C	3.436899873	2.316207705	0.16564169
H	0.16649566	-3.610393779	1.723430621
C	2.131257241	1.861669332	-0.03466851
C	0.449371825	5.10144203	-1.804483374
H	1.499577673	4.78605915	-1.852262485
H	1.715712675	-4.24098628	2.300621186
H	4.005158714	2.699309926	-0.677563949
H	1.783858175	-1.90506788	0.41186117
H	-3.09740858	2.435084863	-2.388534622
C	1.495895683	1.902286557	-1.398851579
H	0.054261275	5.140959586	-2.81939898
H	2.062937081	2.557023118	-2.070141304
H	-2.632060458	0.718660793	-2.328588436
C	-2.521239568	1.648597295	-2.892087054
H	1.521609261	0.891243566	-1.826687233
C	-1.034388132	2.025941963	-3.072358161
H	-0.223867735	-0.030610564	-3.035440584
H	-2.972403583	1.4943822	-3.882090166
C	-0.277757118	0.834717632	-3.69764228
H	-1.524312684	4.073655804	-3.766236519
H	0.108600068	3.52279217	-4.225090747
C	-0.927113245	3.204381171	-4.060229262
H	-0.812797913	0.523791011	-4.605191273
H	0.738758433	1.114383164	-4.0023739
H	-1.316587497	2.862559664	-5.029452942
S	-0.644165442	-2.373549204	-0.856088629
O	0.566301481	-3.137741987	-1.16671514
O	-1.498991397	-2.789867187	0.267993402
O	-0.349699199	-0.84660264	-0.814071458
C	-1.733414913	-2.541763254	-2.367811463
F	-2.788395488	-1.710335934	-2.280077089
F	-1.044792338	-2.24960292	-3.477288481
F	-2.171801148	-3.795872506	-2.435838195
N	-2.700566833	-0.014167814	0.350099092
N	-3.748042469	-0.373383867	0.212443574

H	-2.163983092	2.456974761	2.311854839
C	-2.875667843	4.320048424	4.205668809
N	-3.322644017	3.504767083	3.226067788
C	-4.639894306	3.47717678	2.929571053
C	-5.560853669	4.278492672	3.614257361
C	-5.106741349	5.121965471	4.626184114
C	-3.745653181	5.144413554	4.92711536
H	-3.355931585	5.790319347	5.710072257
H	-5.803707153	5.754211328	5.172797025
H	-6.615149369	4.235133498	3.352437527
C	-1.394664518	4.304128303	4.494096477
H	-1.136518795	5.011920552	5.288722336
H	-1.070965586	3.303892986	4.813547584
H	-0.815776599	4.569042776	3.599420628
C	-5.081355914	2.537457977	1.836022074
H	-6.142283676	2.670656085	1.600381498
H	-4.502971028	2.701416185	0.917485895
H	-4.933444537	1.491039406	2.135775692

Supplementary Methods

X-ray Crystallography. Crystallographic data of **2**·7/3C_4H_8O, **3**·1/6C_6H_{14}, **4,** and **5**·C_4H_8O are summarized in Supplementary Tables 1 and 2. Selected bond lengths and angles are summarized in Supplementary Tables 3–6, and their ORTEP drawings are shown in Supplementary Figures 1–4. A preliminary X-ray analysis of **2**·7/3C_4H_8O has shown that the chroride (Cl(2) and Cl (3)) and nitride (N(2) and N(3)) atoms bonded to molybdenum atom trans to each other are disordered between two positions in a ratio of 0.75:0.25. A preliminary X-ray analysis of **3**·1/6C_6H_{14} has shown that there is a two-fold screw axis running through the Cl(1), N(1) and C(1) atoms and that the molybdenum nitride group (Mo(1)-N(2)) is crystallographically disordered between two symmetrical positions with one-half atomic occupancies. Thus, superficial but false elongation of the Mo≡N bonds are unavoidable for the crystallographic data of **2**·7/3C_4H_8O and **3**·1/6C_6H_{14}, and their metric features around the molybdenum atoms are indiscussible. Diffraction data were collected at −75 °C (**2**·7/3C_4H_8O, **3**·1/6C_6H_{14}, and **5**·C_4H_8O) or −90 °C (**4**) on a Rigaku RAXIS RAPID imaging plate area detector with graphite-monochromated Mo Kα radiation (λ = 0.71075 Å). Reflections were collected for the 2θ range of 5° to 55°. Intensity data were corrected for Lorenz-polarization effects and for empirical absorption (ABSCOR for **2**·7/3C_4H_8O, **4**, and **5**·C_4H_8O) or numerical absorption (NUMABS for **3**·1/6C_6H_{14}). The structure solution and refinements were carried out by using the *CrystalStructure* crystallographic software package.[1] The positions of the non-hydrogen atoms were determined by direct methods (SHELX-97[2]) and subsequent Fourier syntheses (DIRDIF-99[3]), and were refined F_o^2 using all unique reflections by full-matrix least-squares with anisotropic thermal parameters except for all the THF molecules in **2**·7/3C_4H_8O, and one methyl carbon atom (C(4)) and hexane carbon atoms (C(13) and C(14)) in **3**·1/6C_6H_{14}. Location of the imide hydrogen atom (H49) of **5**·C_4H_8O was found in a difference Fourier map and was refined isotropically, while all the other hydrogen atoms except for those in THF and hexane molecules in **2**·7/3C_4H_8O and **3**·1/6C_6H_{14} were placed at the calculated positions with fixed isotropic parameters.

For the crystallographic data of **2**·7/3C_4H_8O, one THF molecule is disordered between two positions (O(2)-C(28)-C(29)-C(30)-C(31) and O(3)-C(32)-C(33)-C(34)-C(35)) and were refined

with one-half occupancies. Other two THF molecules containing O(4) and O(5) atoms are disordered around two three-fold rotoinversion axes, where one-sixth moiety of five non-hydrogen atoms were solved with one oxygen atom, where its atomic occupancy is given as two thirds (32 electrons for non-hydrogen atoms in THF, divided by 6 asymmetric units, divided by 8 electrons for one oxygen atom). The total number of THF molecules per complex **2** is calculated as seven thirds (one for O(1), two halves for those containing O(2) and O(3) , and two sixths for those containing O(4) and O(5)). Consequently, the total void space is filled with the THF molecules.

For the crystallographic data of **3**·$1/6C_6H_{14}$, one methyl carbon atom (C(4)) was disordered and solved with isotropic thermal parameters. Thus, the alert level B errors (PLAT201_ALERT_2_B Isotropic non-H Atoms in Main Residue(s) .... 1) is not avoidable. A hexane molecule consisting of C(13) and C(14) atoms appears as a disorder to form a linearly extended carbon chains along the *c*-axis in **3**·$1/6C_6H_{14}$, where their atomic occupancies were given as one third, corresponding to one-sixth hexane molecule per complex **3**.

For the crystallographic data of **4**, the maximum residual peak exists very near to the molybdenum atom (0.910 Å), thus the alert level B errors (DIFMX01_ALERT_2_B The maximum difference density is > 0.1*ZMAX*1.00_refine_diff_density_max given = 4.700 and PLAT097_ALERT_2_B Large Reported Max. (Positive) Residual Density 4.70 e$Å^{-3}$) are not avoidable.

Computational details

Three possible reaction pathways for formation of the six-coordinate imide complex XIII.

Because the formation of the six-coordinate imide complex **XIII** involves three elementary steps, protonation, reduction, and N_2 coordination, there are three reaction pathways, Paths E, F, and G, to be considered (Supplementary Figure 5). Details in Path E are discussed in the main text. In Path F, protonation of the nitride complex **XI** gives a cationic five-coordinate imide complex **XXII**. Dinitrogen attacks the vacant coordination site in **XXII** to give a cationic six-coordinate imide complex **XXIII**, and then the generated **XXIII** is reduced to give **XIII**. In Path G, dinitrogen first coordinates to **XI** to afford a six-coordinate nitride complex **XXIV** and then protonated. In all the pathways, the protonation step is found to be slightly endothermic, followed by a highly exothermic reduction step. The coordination of dinitrogen is also possible at room temperature.

Supplementary References

(1) *CrystalStructure 3.80: Single Crystal Structure Analysis Software*; Rigaku Corp: Tokyo, Japan, and MSC: The Woodlands, TX, pp 2000–2007.

(2) Sheldrick, G. M. *SHELX-97: Program for the Refinement of Crystal Structure*; University of Göttingen: Göttingen, Germany, 1997.

(3) Beurskens, P. T., Beurskens, G., de Gelder, R., García-Granda, S., Gould, R. O., Israël, R. & Smits, J. M. M. *The DIRDIF-99 Program System*; Crystallography Laboratory, University of Nijmegen: Nijmegen, The Netherlands, 1999.
